# Supplementary material for: Exploring non-retention in clinical trials: a meta-ethnographic synthesis of studies reporting participant reasons for drop out
Source: BMJ Open. 2019 Jun 3;9(6):e021959. doi: 10.1136/bmjopen-2018-021959 (PMC6561611; doi:10.1136/bmjopen-2018-021959)
Supplement: Supplementary data [file bmjopen-2018-021959supp001.pdf]

Enhancing transparency in reporting the synthesis of qualitative research: ENTREQ

ENTREQ Statement: content and rationale

The ENTREQ statement consists of 21 items grouped into five main domains: introduction, methods and methodology, literature search and selection, appraisal, and synthesis of findings (Table 1). For each item, a descriptor and examples are provided. Below we present a rationale for each domain and its associated items.

Table 1

Enhancing transparency in reporting the synthesis of qualitative research: the ENTREQ statement

No	Item	Guide and description	
**1**	Aim	State the research question the synthesis addresses.	See Page 3
**2**	Synthesis methodology	Identify the synthesis methodology or theoretical framework which underpins the synthesis, and describe the rationale for choice of methodology *(e.g. meta-ethnography, thematic synthesis, critical interpretive synthesis, grounded theory synthesis, realist synthesis, meta-aggregation, meta-study, framework synthesis).*	See Pages 3-4 and S1 Box
**3**	Approach to searching	Indicate whether the search was pre-planned (*comprehensive search strategies to seek all available studies)* or iterative (*to seek all available concepts until they theoretical saturation is achieved)*.	See Page 3
**4**	Inclusion criteria	Specify the inclusion/exclusion criteria *(e.g. in terms of population, language, year limits, type of publication, study type).*	See Page 3-4
**5**	Data sources	Describe the information sources used (e.g. *electronic databases (MEDLINE, EMBASE, CINAHL, psycINFO, Econlit), grey literature databases (digital thesis, policy reports), relevant organisational websites,*	See Page 3

No	Item	Guide and description	
		experts, information specialists, generic web searches (Google Scholar) hand searching, reference lists) and when the searches conducted; provide the rationale for using the data sources.	
**6**	Electronic Search strategy	Describe the literature search *(e.g. provide electronic search strategies with population terms, clinical or health topic terms, experiential or social phenomena related terms, filters for qualitative research, and search limits)*.	See Page 3 and Appendix 1
**7**	Study screening methods	Describe the process of study screening and sifting *(e.g. title, abstract and full text review, number of independent reviewers who screened studies).*	See Page 3-4
**8**	Study characteristics	Present the characteristics of the included studies *(e.g. year of publication, country, population, number of participants, data collection, methodology, analysis, research questions).*	See Page 4-5
**9**	Study selection results	Identify the number of studies screened and provide reasons for study exclusion *(e,g, for comprehensive searching, provide numbers of studies screened and reasons for exclusion indicated in a figure/flowchart; for iterative searching describe reasons for study exclusion and inclusion based on modifications t the research question and/or contribution to theory development).*	See Page 4 and Figure 1 and 2
**10**	Rationale for appraisal	Describe the rationale and approach used to appraise the included studies or selected findings *(e.g. assessment of conduct (validity and robustness), assessment of reporting (transparency), assessment of content and utility of the findings).*	See Page 3-4

No	Item	Guide and description	
**11**	Appraisal items	State the tools, frameworks and criteria used to appraise the studies or selected findings *(e.g. Existing tools: CASP, QARI, COREQ, Mays and Pope* [25]*; reviewer developed tools; describe the domains assessed: research team, study design, data analysis and interpretations, reporting).*	See Page 4 and S1 CASP checklist
**12**	Appraisal process	Indicate whether the appraisal was conducted independently by more than one reviewer and if consensus was required.	See Page 4. 1 reviewer (the main author) initially assessed quality of included studies using the CASP criteria and noted any critical aspects of quality with the study team. During subsequent group discussions we continued to discuss and reflect on key aspects of quality.<br>Due to the small number of eligible studies we decided to include all (please see discussion section and also S1 CASP checklist)
**13**	Appraisal results	Present results of the quality assessment and indicate which articles, if any, were weighted/excluded based on the assessment and give the rationale.	Please see discussion section and S1 CASP checklist
**14**	Data extraction	Indicate which sections of the primary studies were analysed and how were the data extracted from the primary studies? *(e.g. all text under the headings "results /conclusions" were extracted electronically and entered into a computer software).*	See Page 4 and S1 Box
**15**	Software	State the computer software used, if any.	N/A
**16**	Number of reviewers	Identify who was involved in coding and analysis.	See Pages 4
**17**	Coding	Describe the process for coding of data *(e.g. line by line coding to search for concepts).*	See Page 4

No	Item	Guide and description	
**18**	Study comparison	Describe how were comparisons made within and across studies *(e.g. subsequent studies were coded into pre-existing concepts, and new concepts were created when deemed necessary).*	See Page 4, S1 Box, S3 Table
**19**	Derivation of themes	Explain whether the process of deriving the themes or constructs was inductive or deductive.	See Page 4,S1 Box, S3 Table
**20**	Quotations	Provide quotations from the primary studies to illustrate themes/constructs, and identify whether the quotations were participant quotations of the author's interpretation.	See Results section and S2 Table
**21**	Synthesis output	Present rich, compelling and useful results that go beyond a summary of the primary studies (e.g. *new interpretation, models of evidence, conceptual models, analytical framework, development of a new theory or construct).*	See Results and discussion section. Also see our conceptual model illustrating our 'line of argument' (S3 Figure).
