# Supplementary material for: Exploring non-retention in clinical trials: a meta-ethnographic synthesis of studies reporting participant reasons for drop out
Source: BMJ Open. 2019 Jun 3;9(6):e021959. doi: 10.1136/bmjopen-2018-021959 (PMC6561611; doi:10.1136/bmjopen-2018-021959)
Supplement: Supplementary data [file bmjopen-2018-021959supp003.pdf]

S1 Box. Our analytical approach

1) Getting started

This stage involved us generating a research question specific to our area of interest that we believed could be usefully addressed by referring to qualitative research. In our case this was the question of what influences non-retention within clinical trials from the perspectives of trial withdrawers?

2) Describing what is relevant to initial interest

This stage involved making a series of decisions relating to deciding what was relevant to our initial area of interest, deciding on the searching process, inclusion/exclusion decisions and quality assessment. In our case we were interested in any study that reported the use of qualitative methods (for collection and analysis of data) to explore the reasons why individual participants withdraw from clinical trials. We were interested in any reports made by participants themselves or by trial staff, but this had to be specifically in relation to why participants withdraw. We defined withdrawal or non-retention as covering any aspect of attrition recognising that this might cover activities such as cessation of, or withdrawal from the intervention(s), non-attendance at clinic visits, through to non-response to some or all follow up questionnaires etc. We decided that we would exclude studies that did not use qualitative means to collect or analyse their data and also studies reporting findings from trial withdrawers who were not patient participants e.g. GPs in a primary care cluster trial. Following these decisions, a systematic search across a range of databases was conducted with assistance from an information specialist (See S1 Appendix) and all titles and abstracts were screened for inclusion (see S1 and S2 Figures). Applying quality criteria to qualitative research remains a contentious issue and there is no consensus regarding whether and how this should be done (Mays 2000; McEwan 2004). However, one author (ZS) undertook a quality assessment of each of the 11 papers that were identified as being eligible for inclusion in the synthesis. Whilst authors of some qualitative evidence syntheses have chosen to exclude what they deem to be poor quality papers, we made the decision not to exclude any of the identified papers. Although all papers had study aims that were amenable to investigation via qualitative means and all included qualitative data, as a team we deemed some as being richer than others in terms of data and insights (i.e. first and second order constructs). Despite this variation in the overall level of quality, due to the small number of identified studies we considered it more important to retain any relevant findings than disregard based on study quality. In doing so, we would argue that all 11 papers contributed useful elements to the collective whole and enabled us to develop our line of argument in terms of the issues of importance regarding trial non-retention.

3) Reading the studies

At this stage, we aimed to become as familiar as possible with the content of all the identified papers with each author independently reading through all of the data provided and making detailed notes of their observations including identification of preliminary themes. After sharing notes, we met to discuss our findings as a team, comparing and contrasting our preliminary observations etc. We repeated this process for the 3 papers that we identified in our updated database search.

4) Determining how the studies are related

In describing this phase, Noblit and Hare 1988 state that “In doing a synthesis, the various studies must be ‘put together’. This requires determining the relationships between the studies to be

synthesized. We think it makes sense to create a list of the key metaphors, phrases, ideas, and/or concepts (and their relations) used in each account and to juxtapose them…" During this next stage (which in practice we found very much related to activities undertaken as part of stage 3), data was extracted initially from all 8 papers (retrieved from the 1st database search) using a standard form which summarised the main phrases, themes and ideas, along with, information regarding methods, and any other important information relating to the context of the research (some of this data is illustrated in S1 Table). During this stage, we focussed on both 1st order constructs within included papers (meaning study participant quotations found in the results section of papers) along with 2nd order constructs (meaning the interpretations made by the papers' authors, usually found in the discussion and conclusion sections of papers but also sometimes within the results). Using the standard form, the papers were initially organised in chronological order (but as inductive analysis progressed papers were grouped according to emerging themes) and we focused on the findings, concepts and themes used by the papers' authors generating a list of key categories. This document (along with our other written notes and observations) facilitated discussions at a series of subsequent team meetings and were very useful for consideration of how identified themes from one paper might relate to the others. We added similar data from the additionally identified 3 papers to this form, to allow us to compare and contrast findings with the earlier 8 papers.

5) Translating the studies into one another

Noblit and Hare 1988 state that "In its simplest form, translation involves treating the accounts as analogies: One program is like another except….It also compares both the metaphors or concepts and their interactions in one account with the metaphors and their interactions in the other accounts."

At this key stage (which again in practice we found inter-related to stage 4), following this process, we sought to consider the extent to which themes and concepts seemed common or distinct across the papers. Our initial grouping of 1st and 2nd order constructs across the 8 papers resulted in 14 sub-themes. These were issues/ideas that we each considered important in terms of things that might make people withdraw from trials. During the process of translating themes/concepts from each of the individual studies into those of the others (i.e. comparing and contrasting across studies), following further team discussion these were then grouped and categorised into 5 broad key themes (as it became apparent that some of sub-themes were related or overlapped). We interpreted our 5 key themes as characterising the main considerations and features that appeared to influence non-retention in the trials under investigation (See S2 Table). For the 3 subsequently identified papers, we repeated this stage by comparing and contrasting concepts and their interactions in these 3 accounts with the concepts identified in the original 8 accounts– in essence comparing for 'fit' and checking for any additional themes (Lang 2013). During this process, we were confident that concepts identified in the later 3 papers supported and complemented our originally identified 5 key themes with no new themes emerging.

6) Synthesizing the translations

Noblit and Hare 1988 state that "Synthesis refers to making a whole into something more than the parts alone imply." For our synthesis, what we were attempting to do at this stage was to move towards an explanatory analysis. We considered and discussed how the various translations compared in an attempt to develop a more nuanced and collective understanding of factors influencing trial non-retention (in doing so, developing our 'line of argument' synthesis). As before, we did this through a process of reflection and team discussions, in an attempt to produce overarching insights into the factors that appear to influence non-retention.

7) Expressing the synthesis

As is common with other meta-ethnographies we sought to express our collective insights in both textual and diagrammatic format within our paper. In doing so, we expressed our synthesis both within our paper as our ‘line of argument’ (with supportive illustrative data from across the studies) and also as a conceptual diagram (see S3 Figure).
