# Supplementary material for: Exploring non-retention in clinical trials: a meta-ethnographic synthesis of studies reporting participant reasons for drop out
Source: BMJ Open. 2019 Jun 3;9(6):e021959. doi: 10.1136/bmjopen-2018-021959 (PMC6561611; doi:10.1136/bmjopen-2018-021959)
Supplement: Supplementary data [file bmjopen-2018-021959supp006.pdf]

S3 Table

	Inter-relationship between self and trial processes/procedures														
**Themes**	**1. Perceptions of current health state in relation to specific aspects of the trial**		**2. The ‘fit of aspects of the trial with individual preferences for care and support**						**3. The compatibility of aspects of trial processes with individual capabilities**		**4. Concerns about or experiences of trial medication**			**5. Considerations around the extent to which trial participation could be appropriately accommodated into individuals’ broader lives**
14 Sub-themes	Perception of being too well	Perception of being too ill	Not individual /tailored/ personalised enough	Too basic	Inflexible	Too technical	Too intensive	Too stressful	Not tailored to individual capabilities	Cognitively not pitched at the individual	Medication not necessary	Potentially dangerous	Contraindications or side effects	Aspects of life getting in the way
Nakash 2008	*									*				*
Nicholas 2010	*	*	*	*	*									*
Postel 2010	*						*	*						*
Eborall 2011	*										*		*	*
Wells 2011	*	*								*				
Shilling 2011												*	*	
Sanders 2012	*			*	*	*								
Johansson 2015	*	*	*		*				*	*				*
Sari 2017	*						*							*
Fernandez-Alvarez 2017		*	*	*										*
Henshall 2018		*												*

*Denotes presence of themes across papers
