# Supplementary figures and images for: Exploring non-retention in clinical trials: a meta-ethnographic synthesis of studies reporting participant reasons for drop out
Source: BMJ Open. 2019 Jun 3;9(6):e021959. doi: 10.1136/bmjopen-2018-021959 (PMC6561611; doi:10.1136/bmjopen-2018-021959)

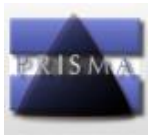

## PRISMA Flow Diagram: Retention Review

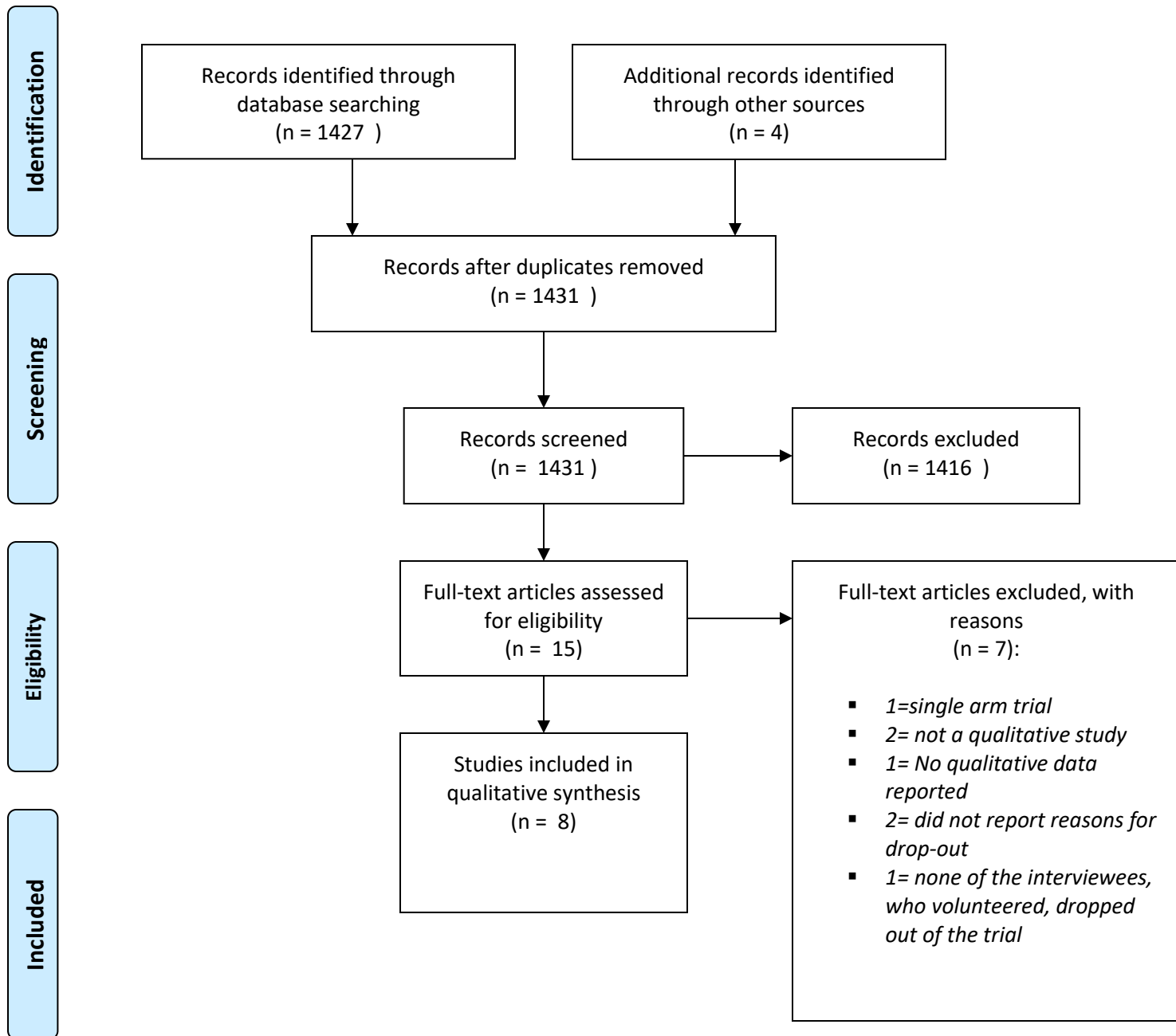

Supplement: Supplementary data [file bmjopen-2018-021959supp008.pdf]

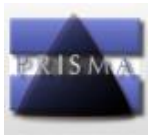

## PRISMA Flow Diagram: Retention Review (search update)

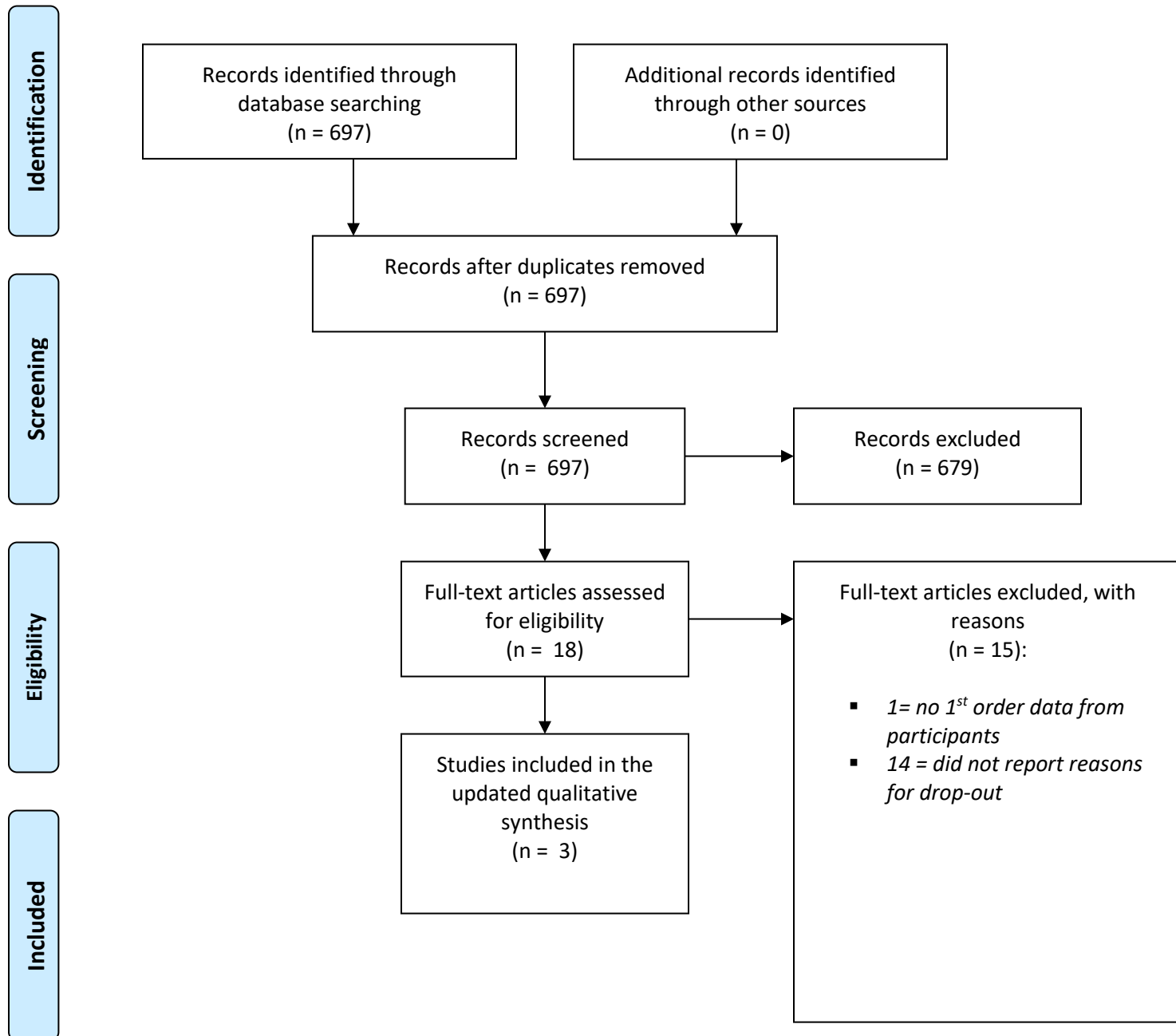

Supplement: Supplementary data [file bmjopen-2018-021959supp009.pdf]

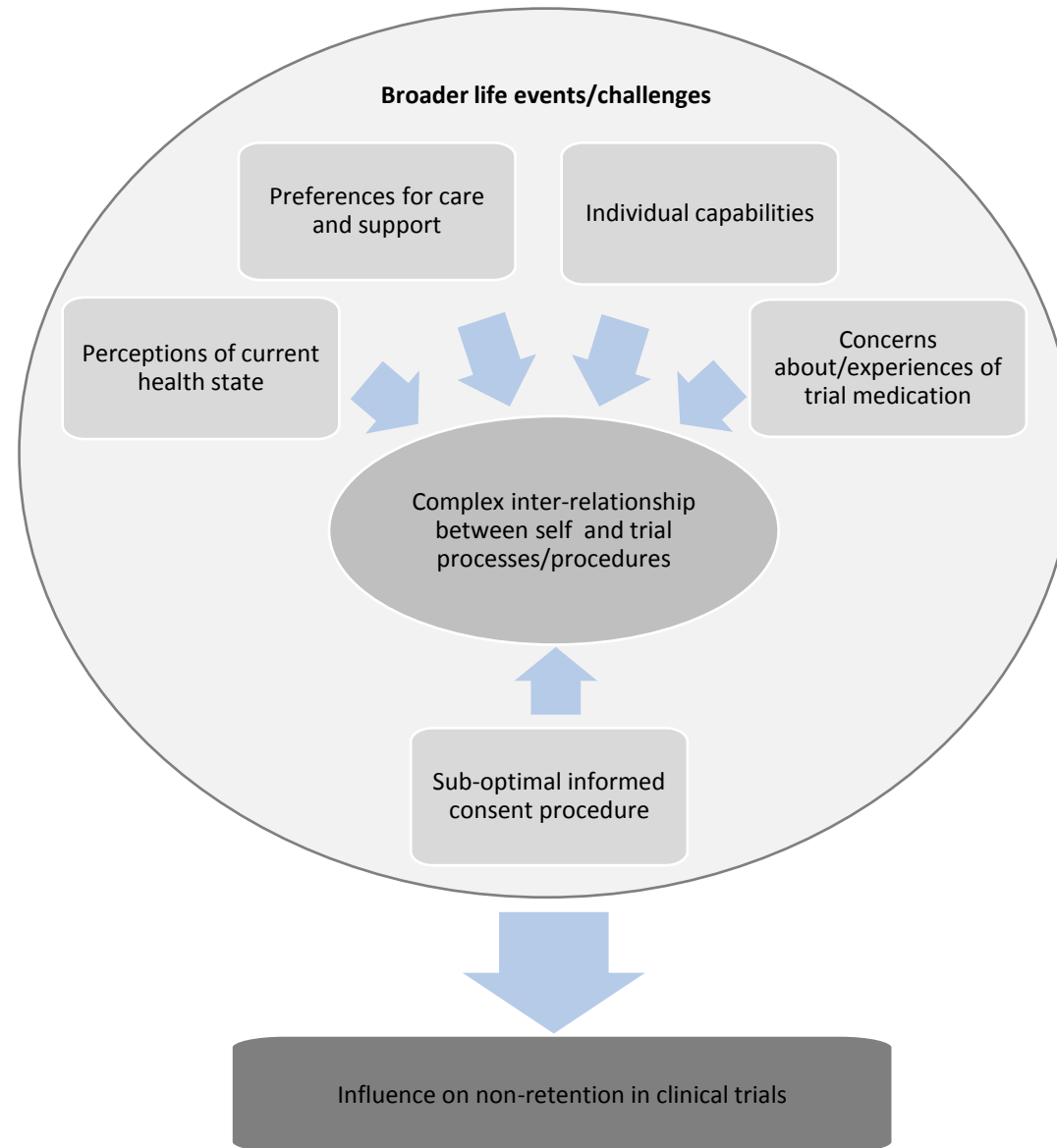

Supplement: Supplementary data [file bmjopen-2018-021959supp010.pdf]
