# Supplementary material for: Exploring non-retention in clinical trials: a meta-ethnographic synthesis of studies reporting participant reasons for drop out
Source: BMJ Open. 2019 Jun 3;9(6):e021959. doi: 10.1136/bmjopen-2018-021959 (PMC6561611; doi:10.1136/bmjopen-2018-021959)
Supplement: Author's manuscript [file bmjopen-2018-021959.draft_revisions.pdf]

BMJ Open

BMJ Open is committed to open peer review. As part of this commitment we make the peer review history of every article we publish publicly available.

When an article is published we post the peer reviewers' comments and the authors' responses online. We also post the versions of the paper that were used during peer review. These are the versions that the peer review comments apply to.

The versions of the paper that follow are the versions that were submitted during the peer review process. They are not the versions of record or the final published versions. They should not be cited or distributed as the published version of this manuscript.

BMJ Open is an open access journal and the full, final, typeset and author-corrected version of record of the manuscript is available on our site with no access controls, subscription charges or pay-per-view fees (http://bmjopen.bmj.com).

If you have any questions on BMJ Open's open peer review process please email info.bmjopen@bmj.com

BMJ Open

Exploring non-retention in clinical trials: A meta-ethnographic synthesis of studies reporting participant reasons for drop out

Journal:	*BMJ Open*
Manuscript ID	bmjopen-2018-021959
Article Type:	Research
Date Submitted by the Author:	06-Feb-2018

Complete List of Authors:	Skea, Zoë; Universit of Aberdeen, Health Services Research Unit<br>Newlands, Rumana ; University of Aberdeen College of Life Sciences and Medicine, Health Services Research Unit<br>Gillies, Katie; University of Aberdeen, Health Services Research Unit

Keywords:	QUALITATIVE RESEARCH, Clinical trials < THERAPEUTICS, Retention

SCHOLARONE™
Manuscripts

Exploring non-retention in clinical trials: A meta-ethnographic synthesis of studies reporting participant reasons for drop out

Author: Dr Zoë Christina Skea,

Co-authors: Dr Rumana Newlands, Dr Katie Gillies.

Corresponding author: Dr Zoë Christina Skea, Health Services Research Unit, 3rd Floor, Health Sciences Building, University of Aberdeen, Aberdeen, AB25 2ZD, Tel: +44 (0) 1224 438153. email:z.skea@abdn.ac.uk. Fax: +44 (0) 1224 438165.

Co-authors. Dr Rumana Newlands, Health Services Research Unit, University of Aberdeen, Aberdeen. Dr Katie Gillies, Health Services Research Unit, University of Aberdeen, Aberdeen.

Key words: Qualitative research; Clinical Trials; Retention.

Word count: 6355

For peer review only

For peer review only - http://bmjopen.bmj.com/site/about/guidelines.xhtml

Abstract

Objectives

To undertake a meta-ethnographic synthesis of findings from primary qualitative studies that have explored participant reported factors influencing non-retention within a clinical trial context.

Design

A systematic search and meta-ethnography was conducted for published papers that contained qualitative data from trial non-retainers.

Participants

We identified 8 qualitative studies reporting data from 9 trials. The studies were undertaken between 2008 and 2015. Each study included between 3 and 40 people who had dropped out from a trial, with findings from 137 people in total reported across the papers.

Results

Emergent from our synthesis was the significance of trial non-retainers' perceptions around the personal 'fit' of key aspects of the trial with their personal beliefs, preferences, capabilities or life circumstances. These related to their own health state; preferences for receiving trial 'care'; individual capabilities; beliefs about or experiences of trial medication; and considerations whether trial participation could be accommodated into their broader lives. All these factors raise important issues around the extent to which initial decisions to participate were fully informed.

Conclusions

To improve retention in clinical trials, researchers should work to reduce the burden on trial participants both through the design of the intervention itself as well as through simplified data collection processes. Providing more detail on the nature of the trial interventions and what can be expected by 'participation' at the consenting stage may prove helpful in order to manage expectations. Early and meaningful patient/public involvement could be particularly important for accommodating future trial participants' preferences and capabilities.

Strengths and limitations of this study

- Trial retention has recently been identified as one of the top three priorities for methodological research by UK trialists.

- Within the context of clinical trials, issues around retention have not received equal scrutiny compared to methodological questions about trial recruitment despite being arguably just as important for trial validity.

For peer review only - http://bmjopen.bmj.com/site/about/guidelines.xhtml

For peer review only

- Understanding the complex reasons why trial participants leave a trial after initially consenting is important if trialists are to be able to design effective intervention strategies to address the problem.

- To our knowledge this is the first synthesis of key findings from qualitative studies exploring participants' perspectives of trial non-retention which provides learning across their collective contributions.

- Our synthesis included 8 eligible papers reporting findings across 9 trials, 4 of which were set within a mental health context and all of which were conducted in high-income countries.

Introduction

Randomised controlled trials are integral for evidenced based clinical decision making. Within the context of clinical trials, the focus of much methodological research in recent years has been on issues specifically relating to trial recruitment, including significant investigation into how to increase the numbers of prospective participants recruited [1,2]. A key focus of much of this research has been on trial participants' perspectives and experiences particularly around why they do or do not choose to consent to participate in clinical trials [3,4,5,6,7,]. Whilst issues relating to trial recruitment are undoubtedly important, issues around retention (i.e. ensuring that trial participants remain in the trial to provide primary outcome data) have not received equal scrutiny in the literature despite being arguably just as important for trials in terms of ensuring that research questions are adequately answered [2].

Trial retention was recently identified in the top three priorities for methodological research by UK trialists [8]. Most trials experience the issue of missing data often referred to as a 'loss to follow-up', 'attrition' or 'drop out' and this can bias the findings of a trial. Some recent quantitative surveys have identified participant characteristics (e.g. age, gender, physical or mental health) or trial processes (e.g. study duration or length and relevance of outcome measures) as being potential predictors of trial retention [9,10,11]. However, these studies are small in size, often limited to a particular clinical context, and the items included in the surveys are often identified by researchers rather than asking participants what items should be included. In addition, they lack any in depth exploration of the relevant issues affecting why participants withdraw, as reported by participants.

Understanding the complex reasons why trial participants leave a trial (either actively (e.g. by requesting no further follow up or purposefully not returning data) or passively (e.g. forgetting to return a questionnaire or attend a clinic visit)) after initially consenting to participation is important especially if those reasons are modifiable. This understanding of participant perspectives then becomes crucial if trialists are to be able to design effective intervention strategies to address the problem.

For peer review only - http://bmjopen.bmj.com/site/about/guidelines.xhtml

The approach of conducting in-depth qualitative research within the context of clinical trials is considered particularly useful for improving the evidence base for how trialists conduct them [12]. Indeed this approach has been used widely to explore perspectives on trial recruitment both in terms of primary qualitative studies and secondary syntheses. To our knowledge this is the first synthesis of key findings from studies exploring participants' perspectives of trial non-retention which provides learning across their collective contributions. Our aim was to undertake a meta-ethnographic synthesis of findings from primary qualitative studies and our specific research question was 'what influences non-retention in clinical trials'?

Methods

A systematic literature search and meta-ethnography was conducted (See S1 ENTREQ Checklist). Meta-ethnography essentially involves an 'interpretive and inductive' approach to synthesising studies [13,14]. Essentially meta-ethnography involves the process of 'translating' the findings of individual qualitative studies so that they can be considered in relation to one another with the aim of identifying and building new conceptual knowledge on a particular topic [13,14]. The process of 'translating' findings across studies can be either 'reciprocal' or 'refutational' depending on how individual studies relate to each other [13].

Searching and identification of relevant studies

A systematic search was conducted for published papers that contained qualitative data about trial participants' reasons for not completing some or all of the processes involved in a clinical trial after initially consenting to take part (which we describe as constituting non-retention). Search strategies were informed by previous studies [12] and are provided in Appendix 1. Seven electronic databases were searched by an information specialist: Embase, Ovid MEDLINE, PsycINFO, Cochrane Central Register of Controlled Trials, The Social Sciences Citation Index (SSCI), Cumulative Index of Nursing & Allied Health Literature, and Applied Social Sciences Index and Abstracts (ASSIA) and covered papers published from 1946 to August, 2016. Google Scholar and bibliographies of identified publications were also searched manually for additional potentially eligible papers.

One author (RN) screened all titles and abstracts with a second author (KG) screening a random 10% sample. Eligible studies included those that used qualitative methods and contained qualitative data exploring any aspect of non-retention from the perspective of patient participants (recognising that non-retention might cover activities such as cessation of or withdrawal from the intervention(s), non-attendance at clinic visits, through to non-response to some or all follow up questionnaires etc).

Analysis and synthesis

In order to collate and synthesise the available primary research, the seven steps of meta-ethnography as listed in S1 Box were followed. In summary, the three authors (ZS, RN, KG) each

For peer review only - http://bmjopen.bmj.com/site/about/guidelines.xhtml

For peer review only

read and systematically extracted data from the included papers, shared notes and discussed study findings and interpretations during a series of group meetings. The papers were initially organised in chronological order (but as inductive analysis progressed papers were grouped according to emerging themes) and we focused on the findings, concepts and themes used by the papers' authors generating a list of key categories. We used a standard form which summarised the main themes, information regarding methods, and any other important information relating to the context of the research within each study (some of this data is illustrated in S1 Table). Although we initially organised papers chronologically in this table, we used it to facilitate a series of further group discussions around emerging issues and as inductive analysis progressed we grouped and discussed our data according to the 5 key emerging themes**.** In line with the process of undertaking a meta-ethnography, primary data or 'first order constructs' (quotations from study participants who had not completed any or some of the various trial processes) and authors' interpretations of these data ('second order constructs') were extracted, compared and contrasted between studies (enabling us to produce a 'reciprocal translation'), and organised into themes to facilitate the development of new insights or a 'line of argument' [13].

Study Quality

One author (ZS) undertook a brief quality assessment of each of the papers included in the synthesis. This was based on the Critical Appraisal Skills Programme (CASP) criteria [15] which was used to appraise the identified primary studies and consider their inclusion into the synthesis. Questions developed by the CASP have been used previously for appraising the quality of studies for inclusion in meta-ethnography [16,17,18,19,20].

Results

Description of Studies

The database search produced 1431 abstracts (see S1 Figure for details). We only included studies that provided identifiable data about reasons for non-retention from the included study participants and/or in the authors' reflections. In all, 8 papers met our inclusion criteria. The focus and key study characteristics for the 8 included papers are outlined in S1 Table. The identified papers were conducted in 5 countries (UK, USA, Australia, Sweden and The Netherlands) and discussed non-retention in 9 separate trials. Four of the papers focussed solely on reasons for non-retention [21,22,23,24], with the remaining 4 also considering reasons for consenting [25], non-consenting [26,27] and retention [28]. Each study included between 3 and 40 people who had dropped out from a trial, with findings from 137 people in total reported across the papers. As can be seen from S1 Table the setting of the trials in which the qualitative research was embedded included a range of clinical contexts such as: mental health problems [21,24]; mental health problems and cancer [23]; problem drinking [22]; diabetes, Chronic obstructive pulmonary disease, heart failure, or social care needs [26]; severe ankle sprains [28]; asymptomatic atherosclerosis [27]; neurodevelopment disorders [25], and osteopenia [25]. As expected, the clinical context differed so did the interventions under investigation and included: telehealth equipment or tele care devices [26]; web-based psycho-educational/cognitive therapy based support tools [21,22,24]; anti-depressant medication and/or cognitive behavioural therapy [23]; various mechanical ankle supports [28];

For peer review only - http://bmjopen.bmj.com/site/about/guidelines.xhtml

aspirin [27]; melatonin [25]; and bisphosphonate risedronate or vitamin D analogue 1 – alphahydroxychol ecalciferol [25].

Findings were presented from trial non-retainers both before outcome data had been collected (e.g. those who withdrew from the intervention) and/or during the follow up when outcome data was being collected (See S1 Table for a summary of non-retention behaviour i.e. non-adherence to intervention, non-return of questionnaires). For example, 7 studies reported aspects related to non-adherence to trial intervention: 3 of these reported cessation of trial medication [23,25 for both trials,27]; 4 reported cessation of treatment therapy sessions [21,22,23,24]; 1 reported cessation of use of telehealth equipment or tele health devices [26] and another reported non-completion of study workbooks [21]. Two studies reported non-return of follow-up questionnaires [22, 28]. Three of the 8 studies appeared to have included only the views of those who had dropped out of the active intervention arms of the trial [23,24,26]. For 3 studies it was unclear whether data was from intervention or control groups [25 for both trials, 27,28] and only 2 studies specifically stated that they included views of both those in the intervention and control groups [21,22].

Six of the 8 studies used semi-structured interviews to collect data from people who had withdrawn from the main trial [21,23,24,25,26, 28]; 1 used a combination of focus groups and interviews[27] and another distributed a questionnaire that contained various open ended response options [23] (NB: only the qualitative data are reported and referred to in this paper). Although some papers provided gender, age and/or demographic details for participants taking part in the trial in question, as can be seen from S1 Table, this information was less comprehensive for those who had dropped out of the trial. Where participant characteristic information was provided in the original studies we have included this at the end of the quotes presented to illustrate findings.

Key themes from the synthesis

Our initial grouping of first and second-order constructs across the 8 papers resulted in 14 sub-themes. During the process of translating themes from each of the individual studies (i.e. comparing and contrasting across studies) these sub-themes were then grouped and categorised into 5 broad key themes which characterised the main considerations and features that appeared to influence non-retention in the trials under investigation (See S2 Table). These themes were: 1) Perceptions of current health state in relation to specific aspects of the trial; 2) the 'fit' of aspects of the trial with individual preferences for care and support; 3) the compatibility of aspects of trial processes with individual capabilities; 4) concerns about or experiences of trial medication; and 5) considerations around the extent to which trial participation could be appropriately accommodated into individuals' broader lives.

As these theme labels suggest, within them they accommodate a spectrum of views or experiences.

The 5 broad key themes identified as influencing participants' non-retention in clinical trials are illustrated with example data in S2 Table. In S2 Table, primary study participant quotes illustrating first order constructs are displayed in italics, and primary study author interpretations illustrating second order constructs are presented in bold text. In the rest of this paper, primary study participant quotes are displayed in italics.

Influences on participant non-retention in clinical trials: a line of argument

For peer review only - http://bmjopen.bmj.com/site/about/guidelines.xhtml

Expressed below is our 'line of argument' which is organised into themes to facilitate the development of cumulative insights (S2 Figure conceptually illustrates the line of argument developed from the synthesis). Our argument emphasises the significance of trial participants' perceptions around the 'fit' of key aspects of the trial with their personal beliefs, preferences, capabilities or life circumstances. These factors related to their own health state, preferences for how they wanted to receive care, their individual capabilities, beliefs about or experiences of trial medication, and also considerations around the extent to which trial participation could be appropriately accommodated (or not) into their broader lives. Implicit within several of these identified factors is the suggestion that there may have been deficits within the initial consenting process which led to participants (who subsequently withdrew) not being fully informed or at least not realising what the trial expected of them and what they could expect of the trial. These are discussed in more detail below and arranged across 5 key themes

1) Perceptions of current health state in relation to specific aspects of the trial.

This theme describes how aspects of the trial might not be right for people as individuals. For example, across 7 of the 8 studies a key influence on decisions to discontinue trial participation appeared to relate to perceptions of either being 'too well' to warrant further engagement with the trial [21,22,23, 26, 27,28] or struggling with the compatibility of aspects of the trial, particularly the interventions or ways outcomes were assessed, with their personal sense of self [21,22,26,27]. Conversely, within the same set of studies, other participants described periods of feeling too unwell to be able to engage appropriately in trial processes.

a) Being too well to engage further with trial processes

Some participants cited a belief that they had suitably recovered part way through a particular trial as a reason for discontinuing trial medication and/or problem solving treatment exercises [21,22,23]:

"Things really improved for me…I just felt really good and didn't really feel like I had that much to offer in regard to finding out more about it" [21; Female, 30-39 years, Bipolar disorder, control group]

"I have been sufficiently helped" [22; No gender/age details, Problem drinker, Intervention group]

Participants also cited recovery as a reason for not completing and returning all the required follow-up outcome assessment questionnaires [28, Severe ankle sprains] perhaps highlighting here the importance at the consenting stage of making sure participants are fully informed about the value of sustained engagement throughout the duration of the trial (even if they feel they are no longer personally benefiting from that engagement).

b) Lack of compatibility with personal sense of self

Sometimes reasoning around trial withdrawal related to participants' struggle to accommodate aspects of the trial with their personal sense of self at the time [1,26,27], suggesting that the intervention challenged their sense of self somehow. Again perhaps indicating the importance for initial trial recruitment consultations to include adequate discussions about the nature of the study intervention and also what will be expected of participants in terms of engagement with them. For example, a belief that they could self-manage or cope well enough without the need to engage with

For peer review only - http://bmjopen.bmj.com/site/about/guidelines.xhtml

the trial support intervention [26; self –care intervention to facilitate support for self-management in aging populations]; a belief that they had adequately managed their condition thus far without the need for any medication [27; aspirin for asymptomatic atherosclerosis] and also non-acceptance of a diagnosis amongst those newly diagnosed [21,Nicholas; with bi-polar disorder] as a reason for not relating to (or seeing any value in) the study interventions:

[Discussing the need to keep active rather than monitoring his health indoors using tele-health equipment] *"You've got [to have] the will power…if you can't do it I am finished. If I wouldn't have that I'd be, I'd be stuck inside here you know, and looking through the window like…I throw myself in the garden and everything. Everything I do I'm working on, I cook myself dinners and everything.'* [26; Male; 85yers; COPD]

"I think if it had been medication that I needed to take, I would have taken it" [27; Male; 72; stopped taking aspirin medication]

"If you're taking a lot, it knocks the hell out of your stomach…Given the choice, I'd rather not take medication full stop" [27; Male; 55; stopped taking trial medication for asymptomatic atherosclerosis]

"I wasn't ready to accept the illness. At that stage after diagnosis I wasn't willing to change my life according to the program." [1; Male; 18-29 yrs; Bipolar disorder, control group]

c) *Being 'too ill' to be able to engage appropriately with trial interventions*

Conversely, within those papers focussing on interventions for mental health conditions, other participants described being 'too ill' to be able to engage appropriately in trial processes [21,23,24]. Reasons discussed in this context related to feeling either too depressed, too manic, or too emotional/stressed at certain times to be able to complete the required intervention tasks (e.g. e-health intervention and associated workbook activities; cognitive behavioural therapy) and also a concern that engagement with the intervention could act as a 'trigger' in terms of exacerbating anxiety symptoms:

"I did not cope with the exercises. I did them at the start but it gradually became more difficult to complete them…..particularly the breathing exercises. I got a bit dizzy and it increased my feelings of anxiety" [24, no gender or age details, generalised anxiety disorder]

"The biggest problem I have with my bipolar disorder is consistency; when I'm down I can't even brush my teeth or get up in the morning. So doing an education program with workbooks was beyond me" [21, Female, 18-29 yrs, Bipolar disorder, BEP group]

"I often go walking when having highs because I have to keep moving, so I didn't want to sit at a computer" [21, Male, 40-49 yrs, Bipolar disorder, BEP+IS group]

2) The 'fit' of aspects of the trial with individual preferences for care and support

Across 6 of the 8 studies another important influence in decisions to discontinue trial participation appeared to relate to the fit of aspects of the intervention with preferences for how participants wanted to receive care and support [21, 22,23,24,26,28], implicitly suggesting that the initial trial consenting process may have been sub-optimal in key ways. Participants in these trials discussed

For peer review only - http://bmjopen.bmj.com/site/about/guidelines.xhtml

how aspects of the design of the interventions were not individualised or tailored enough to be helpful and others commented on interventions being either too technical/ too intensive or conversely too basic:

"I would have liked to have more of a personal contact, it became a little distant everything, to do on the internet, because it is so heavy stuff, it's nice to meet a real person when you're working with heavy things like this" [24, no gender or age details, generalised anxiety disorder]

"I wanted something more about me specifically, as opposed to talking about general issues" (Nicholas, Male, 40-49 yrs, Bipolar disorder, BEP group)

"The information in the modules was too general and too limited" [21, Male, 18-29 yrs, Bipolar disorder, BEP group]

Some other participants simply indicated that they had been unhappy or dissatisfied or "not comfortable" with the treatment they had received although specific reasons were not provided within the included studies [22,23, 28].

3) The compatibility of aspects of trial processes with individual capabilities

Across 3 of the 8 studies [23,24,28] the extent to which aspects of the interventions were deemed to be appropriately 'pitched' at the individual emerged as being of importance. For example, participants cited attention problems and limited reading and writing skills as a reason for withdrawing from internet delivered cognitive behavioural therapy [24] or as a reason for non-response to follow-up questionnaires [28]. Communication and cultural issues were also cited as reasons for the discontinuation of problem solving treatments [23], suggesting that these issues would benefit from greater consideration and discussion at the consenting stage:

"I thought that it was too much to read, and I cannot read anything at all that I need to remember or learn. It goes in here and out there [pointing at the ears]" [24, no gender or age details, generalised anxiety disorder]

4) Concerns about or experiences of the trial medication

Across 2 of the 8 studies which were set in trials testing drug interventions, [25 – 2 trials; 27] concerns about the study medication were cited as reasons for discontinuing with trial participation. These included concerns that the trial drug(s) were not properly tested/licensed [25], concerns that the trial medication could negatively interact with other prescribed medication [27], through to citing a dislike of taking too much medication [27] or that the trial medication tasted offensive [25]. Constructs within this key theme again suggest potential issues with the informed consent process and highlight the importance of discussions about the purpose of any trial, the nature of trial medications and also the implication for participation of having certain co-morbidities:

"It just scared me when it said not to be given to children under 20…I didn't understand they weren't licensed for children…and that's what I thought it was, just to see if it worked, not to actually like so then it could be licensed" [25; Mother of child in trial for young people aged 4-18yrs with rheumatic diseases]

For peer review only - http://bmjopen.bmj.com/site/about/guidelines.xhtml

"again I found that I had stomach problems with the tablet so I assumed that it must be the aspirin…[27, Female, 63 yrs, stopped taking trial medication for asymptomatic atherosclerosis]

"..and they discovered I had heart fibrillation…After that I'd to go on warfarin you see, so that's why I had to drop out because warfarin and aspirin just don't agree" [27, Female, 77 yrs, stopped taking trial medication for asymptomatic atherosclerosis]

"I didn't think I really wanted to go on at the start but mum and dad persuaded me to. And so…when I was getting really fed up I just said 'No I don't want to' because I didn't like the taste [of the medicine] [25; POP trial; young person 11-14 yrs]

5) **Considerations around the extent to which trial participation could be appropriately accommodated into their broader lives**

Aside from issues relating to beliefs about current health state, individual capabilities, preferences for care and concerns about side effects, participants also discussed how decisions to discontinue with trial participation related to other life 'events' that tended to take priority over or made it hard for them to engage fully with the various demands of the trial [21, 22,23,24,28]. These factors appeared less directly related to the nature of the trial interventions themselves and more about the challenges of life in general (with one study [28] suggesting that these people could be classed more as 'happy' rather than 'unhappy' non-responders, in the sense that non-retention may be related to aspects out-with the trial itself). Reasoning here involved trading off trial participation with competing priorities and ranged from events such as work or family commitments, exams, pregnancies, postal strikes etc and more generally simply daily routines that got in the way. Within this theme participants also sometimes cited 'laziness' or 'forgetfulness' as reasons for why they had either not completed trial interventions or had not responded to follow-up questionnaires with some apparently being unaware that they were being considered as 'drop-outs' be study researchers:

[discussing cessation of therapy sessions/non-completion of study workbooks]*"I didn't have the time, and with everything else, it wasn't a priority"* [21, Female, 18-29 yrs, Bipolar disorder, control group]

[describing why they did not return a follow-up questionnaire] *"Do you know what…laziness I'm just gonna put it down to that"*

Researcher: *"OK and em it wasn't because you were disgruntled about part of the project?"*

"Definitely not no" [28, no gender/age details given, severe ankle sprains)

[discussing cessation of problem solving treatment sessions]"Did I drop out? No, I didn't dropout. I became busy and I figured I started missing calls." [23, Female, no age details, Cancer and depression)

Discussion

For peer review only - http://bmjopen.bmj.com/site/about/guidelines.xhtml

Principal findings

Our meta-ethnographic synthesis sought to explore factors that influence non-retention within clinical trial contexts. We identified 8 qualitative studies reporting data from 9 trials that explored participant reported reasons for not completing any or some of the various trial processes (after initially consenting to take part). What emerged from our analysis was the importance of trial participants' perceptions about the personal compatibility of key aspects of the trial with their personal beliefs, preferences, capabilities or life circumstances. These factors related to their own health state, preferences for how they wanted to receive care, their individual capabilities, beliefs about or experiences of trial medication, and also considerations around the extent to which trial participation could be appropriately accommodated or not into their broader lives (Conceptually illustrated in S2 Figure). Our synthesis has also highlighted that people's reasoning around dropping out of a trial can be described as being more or less 'active' in nature, with some people in our synthesis not even realising that they were being considered by the researchers as trial 'drop outs' [23]. All these factors raise important issues around the extent to which initial decisions to participate were fully informed and illustrate the importance for trial recruiters of ensuring that prospective participants are made aware of what the trial will entail and also what will be expected of them in terms of full participation.

Quantitative surveys have tended to investigate non-retention in the context of non-response to follow up questionnaires. These studies have identified either participant characteristics or trial processes as being potential predictors of trial retention [9,10,11].Whilst these studies have a place, it is arguably difficult to influence some of these previously identified factors influencing retention as they may not be modifiable e.g. age or study duration. Our synthesis of more in depth qualitative studies has usefully built on these findings and has enabled a more nuanced understanding of key issues of relevance (which are potentially modifiable) relating to non-adherence to interventions and non-return of follow-up questionnaires. Participant characteristics as well as trial processes are of importance but we have also demonstrated that there can be a complex inter-relationship between the two. For example, a perception that the nature of the intervention negatively affects one's mental health can be of importance as can perceptions about the nature of the intervention in relation to perceptions of self or in relation to personal preferences for care and support. Furthermore, the compatibility or otherwise of various trial processes with individual capabilities can have implications for retention. Reasons given for not completing various trial processes were not necessarily mutually exclusive, but were rather a synergistic combination of factors that could apparently work towards trial non-retention. Our findings also highlight that some participants' behaviour around leaving a trial could be described as being more or less 'active' in nature (e.g. stopping trial medication because of a concern around side effects (active) versus simply not remembering or being too busy to return a questionnaire (passive)). This is an important finding and one that has not been given due consideration in previous literature to date. Different types of trials are likely to present particular challenges in terms of their potential for non-retention. It could also be that certain types of reasoning might be more or less modifiable and easier to address particularly if they can be anticipated upfront during the trial design stage.

A recent study exploring reasons why people declined trial participation at the consent to recruitment stage has found that most declined at the outset because they judged themselves ineligible or not in need of the specific trial therapy in question [29]. The study authors suggest that

For peer review only - http://bmjopen.bmj.com/site/about/guidelines.xhtml

to improve recruitment to trials the most successful interventions are likely to be the ones that focus on patients' assessments of their own eligibility and their potential to benefit from the trial treatment, rather than reducing trial burden per se. In our synthesis we found that perceptions around eligibility and assessments regarding potential to benefit from the trial treatment were also considerations for people who had initially decided to join but who had subsequently ceased to engage. For example, this included those who felt that they had recovered such they did not need to engage further [21,22,23] and those who felt they could manage sufficiently well without engaging with the intervention [21,26,27]). However, in the context of non-retention, it is worth considering issues around trial burden (e.g. interventions that might be perceived to be too technical or too demanding given a person's health state) as well as issues around preference for particular styles of care and support and acknowledging that the specific intervention and, or, the ways outcomes are assessed has to be compatible within the context of trial participants' broader lives. In other words, issues around reducing trial burden is of importance, both in terms of the intervention itself and also the ways that follow up data is collected.

We know from previous syntheses of qualitative studies focusing on trial recruitment that people often choose to enter into trials in the hope of gaining some help for themselves from the intervention (even if they also state they are doing so for altruistic reasons – i.e. to benefit research more generally), so called 'conditional altruism' [7]. Some participants in our synthesis described perceptions around feeling too ill to continue taking part or feeling suitably better such that trial engagement was no longer warranted [21,22,23,24]. This perception of improvement in health would appear to resonate with the concept of conditional altruism in the sense that people might cease participation if they perceive their condition improves or conversely deteriorates, such that in effect their benefit for self has been realised and their continued participation is no longer warranted. Our finding here is perhaps exaggerated in trials with a mental health context (which applied to 4 of the 9 included trials), where diagnoses can adversely affect people's ability and inclination to initially take part in research [29,30,31]. We have shown that this issue also has relevance for retention in such trials as people's health states can be particularly vulnerable to fluctuation [29]. A recent meta-synthesis of factors affecting recruitment to depression trials [32] indicated that decisions can depend on issues relating to: perceptions of health at the time of invite; attitudes towards the research and trial interventions; and the demands of the trial. Our synthesis has shown that some of this reasoning might also have the potential to impact on non-retention in those who are successfully recruited. This comparable finding could suggest that the underlying beliefs, preferences and expectations about trial participation are not explored and unpacked fully during trial consenting discussions.

Strengths and limitations

Undertaking a meta-ethnographic synthesis of findings from qualitative studies that explored factors influencing non-retention within clinical trials has allowed us to gain important new shared insights into factors that seem to affect retention across a range of trial contexts - to our knowledge this is the first study to have synthesised these primary studies in this way. However, our systematic search identified only 8 eligible papers reporting findings across 9 trials, 4 of which had a mental health context and all of which were conducted in high-income countries. Whilst qualitative research does not usually intend to be generalizable, it is nevertheless important to consider the transferability of our findings to other clinical trial contexts and settings and one could argue that participants within

For peer review only - http://bmjopen.bmj.com/site/about/guidelines.xhtml

e.g. mental health trials, or trials that involve surrogate/proxy consent including those involving children [25]might face very different issues and challenges regarding retention. However, we were reassured that the key themes we identified had resonance across all of the 8 included papers to a greater or lesser extent and so are likely to be important considerations within a range of clinical trial contexts. Our original systematic literature search was undertaken in August 2016. Since undertaking and expressing the synthesis within this paper we have updated our search (to October 2017) and have identified 4 additional potentially relevant papers [33,34,35,36]. As this is a qualitative meta-synthesis the process of updating is not straightforward (as all included studies would have to be re-considered again both individually and collectively) and there is currently no guidance on how to do this for meta-ethnographies [7]. Although we may formally update our meta-ethnography in the future we have chosen not to integrate findings from the 4 newer identified studies within this particular synthesis. We have however read all 4 papers and are confident that the key 1st and 2nd order constructs presented across them would not significantly change the overall conclusions arising from this work.

We carried out a quality assessment of the 8 included papers. Although all papers had study aims that were amenable to investigation via qualitative means and all included qualitative data, some were deemed richer than others in terms of data and insights (i.e. first and second order constructs). Despite this variation in the overall level of quality, due to the small number of included studies we felt it was more important to retain any relevant findings rather than disregard based on study quality. In doing so, we would argue that all eight papers contributed useful elements to the collective whole and enabled us to develop our line of argument in terms of the issues of importance regarding trial non-retention.

Practice Implications

Some people in our synthesis appeared to be unaware that they were being considered as trial non-retainers by the study researchers. This raises the question of participants' understanding of the importance of remaining in a trial for its duration (i.e. completing the intervention and the outcome assessments) and its implications for the study in question.

A recent study of patient information documentation from UK NIHR funded trials has highlighted that withdrawal and retention are poorly described and that statements about the value of retention are infrequent [37]. The authors of this study also noted that there was an over-emphasis on a prospective participant's right to withdraw without corresponding information about the importance for research of sustained participation [37]. If trialists want to improve retention to clinical trials as well as recruitment then there is an argument for giving the latter more prominence in patient information materials (and also during any trial recruitment discussions). Trial decision aids have shown some promise here in terms of having the potential to support more informed decisions around trial participation including around issues of understanding, and also the extent of involvement and commitment to a trial over time [38].

Our synthesis also potentially highlights the issue of people's awareness or lack thereof of what the trial interventions would entail. For example, some participants were apparently not fully aware of what the intervention would entail before consenting and described after they had initially agreed to

For peer review only - http://bmjopen.bmj.com/site/about/guidelines.xhtml

take part that it was incompatible either with their sense of self (i.e. a perception that it would not help them or was not needed) or that it did not suit with their individual capabilities (i.e. being computer based and being perceived as too technical) [21,23,24,26,27,28]. If trialists want to improve retention then this suggests an argument for also providing more detail on the nature of the trial interventions at the consenting stage in order to manage expectations. We know from previous literature that patient/public involvement at the front end of trial design tends to be extremely limited if indeed it happens at all [39,40]. Given some of the key factors we found as being influential for non-retention, one could speculate that some early and meaningful patient/public involvement would be particularly useful (e.g. for ensuring that aspects of the trial are user-friendly and as compatible as possible with the target population's likely preferences and capabilities).

For peer review only

Implications for Research

A Cochrane review investigating interventions to improve retention in trials has highlighted that most strategies to improve retention have focussed on trying to improve follow-up questionnaire response [41]. Of these interventions, only monetary incentives have been shown to have a significant effect on return of questionnaires and the review highlighted that very few studies included trial participants in their design or development [41]. Our synthesis has demonstrated that there may be a range of issues relevant to trial participants that influence non-retention which may not be amenable to modification by 'incentives' or other interventions that fail to consider participants during development.

As mentioned previously, qualitative methods to improve recruitment to trials is now recognised as a well-established methodology built into the design and delivery of large publically funded clinical trials. The Qunitet Recruitment Intervention (QRI) is gathering momentum across a range of trials and Clinical Trials Units as a mechanism to unpack many of the nuances around how participants are recruited to RCTs [42]. Many of the approaches in the QRI are directly transferable to questions about retention. For example, how it is discussed in consultations and trial paperwork, what do stakeholders (trial participants and trial staff) report as the barriers and facilitators to retention, and work in this area could prove fruitful for minimising non-retention in ongoing RCTs. However, despite there being a clear need for more research in the context of trial retention, we also recognise the inherent challenges for researchers in obtaining the necessary ethical approvals for this type of research (particularly as current recruitment materials for trial participants tend to emphasise prospective participants' right to withdraw without given any reasons etc). Therefore, development of shareable resources to facilitate regulatory approvals may be an important contribution for the trials methodology community.

Finally, given that 4 of the 8 included studies focused on qualitative research within mental health trials there is certainly scope for further primary studies exploring the barriers and enablers to trial retention from a participant's perspective. Ideally, these studies would consider and explore all aspects of trial process relevant for retention but in particular focus on collection of primary outcome data. Interestingly, our search did not identify any studies that had explored reasons for trial participants' non-attendance at trial follow-up visits. This mirrors the findings of the Cochrane review on interventions to improve retention to trials which also found very little evidence with regard to interventions to improve attendance at follow-up visits [41].

For peer review only - http://bmjopen.bmj.com/site/about/guidelines.xhtml

Conclusions

Our systematic literature search and synthesis has highlighted that there is very little published qualitative literature exploring participant reported reasons for non-retention in clinical trials. Researchers have already called for 'a science of recruitment' in recognition that recruiting for science (e.g. trials) is not currently underpinned by an evidence base around the factors which might have the potential to impact on recruitment [1]. This is undoubtedly important but we would also argue that we need to develop a parallel focus on 'a science of retention' if we are to start to be able to tackle the very real issue of non-retention in clinical trials. Our qualitative synthesis (of albeit a small set of studies) feeds into this relatively undeveloped science and has shed some important light on the factors that might influence non-retention in clinical trials- factors that have implications both for practice and for further research. Taken together, the findings presented here and the subsequent implications for practice and research highlight the critical need to plan for retention as much as for recruitment during trial design and not treat it like the overlooked trial conduct 'Cinderella'.

Supporting Information

S1 ENTREQ Checklist

S1 Appendix. Database search strategies

S1 Box Steps of Meta-ethnography

S1 Figure. PRISMA flow diagram

S1 Table. Characteristics of included studies

S2 Table. Key themes of influence on decisions to withdraw from trial participation

S2 Figure. Conceptual diagram

Acknowledgements

We would like to thank Cynthia Fraser for her assistance in helping develop the database searching strategies.

Contributors

KG conceived the study idea. RN screened all titles and abstracts with KG screening a random 10% sample. ZCS, RN and KG conducted the data analysis and ZCS wrote the initial and subsequent manuscript drafts. All the authors contributed critically to discussions about interpretation of data and revisions of manuscript drafts. All the authors approved the final version.

Funding

For peer review only - http://bmjopen.bmj.com/site/about/guidelines.xhtml

ZCS was supported by a core grant from the CSO (reference CZU/3/3) and a Wellcome Trust Institutional Strategic Support Fund award (reference RG12724-18). RN was supported by a Wellcome Trust Institutional Strategic Support Fund award (reference RG12724-18). KG was supported by an MRC Methodology Research Fellowship (MR/L01193X/1).

Competing interests

None

Data sharing statement

No additional data are available.

References

1. Bower P, Wallace P, Ward E, Graffy J, Miller J, Delaney B, Kinmonth AL. Improving recruitment to health research in primary care. Fam Pract. 2009;26(5):391–7.
2. Adamson J, Hewitt CE, Torgerson DJ. Producing better evidence on how to improve randomised controlled trials. BMJ. 2015;351:h4923.
3. Blanch DC, Rudd RE, Wright E, et al. Predictors of refusal during a multi-step recruitment process for a randomized controlled trial of arthritis education. Patient Educ Couns 2008;73:280–5.
4. Madsen SM, Holm S, Davidsen B, et al. Ethical aspects of clinical trials: the attitudes of participants in two non-cancer trials. J Intern Med. 2000;248:463–74.
5. Verheggen FW, Nieman F, Jonkers R. Determinants of patient participation in clinical studies requiring informed consent: why patients enter a clinical trial. Patient Educ Couns 1998;35:111–25.
6. Locock L, Smith L. Personal benefit, or benefiting others? Deciding whether to take part in clinical trials. Clin Trials 2011;8:85–93.
7. McCann SK, Campbell MK, Entwistle VA. Reasons for participating in randomised controlled trials: conditional altruism and considerations for self. Trials 2010;11:31.
8. Smith CT, Hickey H, Clarke M, Blazeby J, Williamson P. The trials methodological research agenda: results from a priority setting exercise. Trials. 2014;15(1):32.
9. Applebaum AJ, Lichtenthal WG, Pessin HA, et al. Factors associated with attrition from a randomized controlled trial of meaning-centered group psychotherapy for patients with advanced cancer. Psycho-Oncology. 2012; Nov;21(11):1195-204. doi: 10.1002/pon.2013. Epub 2011 Jul 12.
10. McCambridge J, Kalaitzaki E, White IR, Khadjesari Z, Murray E, Linke S et al. Impact of length or relevance of questionnaires on attrition in online trials: randomized controlled

For peer review only - http://bmjopen.bmj.com/site/about/guidelines.xhtml

trial. Journal of Medical Internet Research. 2011 Oct;13(4):e96. e96. Available from, DOI: 10.2196/jmir.1733.

11. Hui D, Glitza I, Chisholm G et al. Attrition rates, reasons, and predictive factors in supportive care and palliative oncology clinical trials. Cancer. 2013:119;5:1098-1105.
12. O'Cathain A, Thomas K, Drabble Set al. The QUAlitative Research in Trials (QUART) study. Health Technology Assessment. 2014;18: No. 38 DOI: 10.3310/hta18380.
13. Noblit GW, Hare RD. Meta-ethnography: synthesizing qualitative studies. 1988. Newbury Park: Sage.
14. Britten N, Campbell R, Pope C et al. Using meta ethnography to synthesise qualitative research: a worked example. J Health Serv Res Policy. 2002;7:4:209–215.
15. Critical Appraisal Skills Programme (2017). CASP (Qualitative Research) Checklist. [online] Available at: http://docs.wixstatic.com/ugd/dded87_25658615020e427da194a325e7773d42.pdf Accessed: July 2017.
16. Campbell R, Pound P, Pope C et al. Evaluating meta-ethnography: a synthesis of qualitative research on lay experiences of diabetes and diabetes care. Social Science and Medicine. 2003;56:671-684.
17. Pound P, Britten N, Morgan M, et al. Resisting medicines: a synthesis of qualitative studies of medicine taking. Soc Sci Med. 2005. 61: 133-155. 10.1016/j.socscimed.2004.11.063.
18. Atkins S, Lewin S, Smith H, et al. Conducting a meta-ethnography of qualitative literature: Lessons learnt. BMC Medical Research Methodology. 2008, 8:21 doi:10.1186/1471-2288-8-21.
19. Schumm K, Skea Z, McKee L, N'Dow J. 'They're doing surgery on two people': a meta-ethnography of the influences on couples' treatment decision making for prostate cancer. Health Expectations. 2010:13, pp.335–349.
20. Black M, Entwistle VA, Bhattacharya S, et al. Vaginal birth after caesarean section: why is uptake so low? Insights from a meta-ethnographic synthesis of women's accounts of their birth choices. BMJ Open 2016;6: e008881. doi:10.1136/ bmjopen-2015-008881.
21. Nicholas J, Proudfoot J, Parker G, et al. "The Ins and Outs of an Online Bipolar Education Program: A Study of Program Attrition."J Med Internet Res 2010 (Dec 19); 12(5):e57.
22. Postel M, de Haan H, ter Huurne E, et al. Effectiveness of a Web-based Intervention for Problem Drinkers and Reasons for Dropout: Randomized Controlled Trial. J Med Internet Res 2010;12(4):e68.
23. Wells A, Palinkas L, Qiu X, et al. Cancer patients' perspectives on discontinuing depression treatment: the "drop out" phenomenon. Patient preference and Adherence. 2011:5;465-470.
24. Johansson O, Michel, T, Andersson G, et al. Experiences of non-adherence to Internet-delivered cognitive behavior therapy: A qualitative study. Internet Interventions. 2015:2;137-142.
25. Shilling, V, Williamson, P R, Hickey, H, Sowden, E, Smyth, R L and Young, B (2011) Processes in recruitment to randomised controlled trials of medicines for children (RECRUIT): a qualitative study. Project Report. 2011: 15. NIHR Journals Library.

For peer review only - http://bmjopen.bmj.com/site/about/guidelines.xhtml

26. Sanders C, Rogers A, Bowen R, et al. Exploring barriers to participation and adoption of telehealth and telecare within the Whole System Demonstrator trial: a qualitative study. BMC Health Services Research*.* 2012: **12**:220 https://doi.org/10.1186/1472-6963-12-220.
27. Eborall H, Stewart M, Cunningham-Burley S, et al. et al. Accrual and drop out in a primary prevention randomised controlled trial: qualitative study.Trials. 2011: 12:7 http://www.trialsjournal.com/content/12/1/7.
28. Nakash R, Hutton J, Lamb S, et al. Response and non-response to postal questionnaire follow-up in a clinical trial – a qualitative study of the patient's perspective. Journal of Evaluation in Clinical Practice. 2008:14;226-235.
29. Hughes-Morley A, Young B, Hempel R, Russell I, Waheed W, Bower P. What can we learn from trial decliners about improving recruitment? Qualitative study *Trials.*2016;**17**:494.
30. Halpern SD, Karlawish JHT, Berlin JA. The continuing unethical conduct of underpowered clinical trials. JAMA. 2002;288:358–62.
31. Drueke TB, Descamps-Latscha B, Locatelli F. Stopping a medical research project for financial reasons. Nephrol Dial Transplant. 2003;18(10):1982–3.
32. Hughes-Morley A, Young B, Waheed W, Small N, Bower P. Factors affecting recruitment into depression trials: systematic review, meta-synthesis and conceptual framework. J Affect Disord. 2015;172C:274–90.
33. Lee SMK, Leem J, Park JH, et al. Close look at the experiences of patients enrolled in a clinical trial of acupuncture treatment for atrial fibrillation in Korea: a qualitative study nested within a randomised controlled trial. BMJ Open 2017;7:e013180.
34. Fernandez-Alvarez J, Diaz-Garcia A, Gonzalez-Robles A, et al. Dropping out of a transdiagnostic online intervention: a qualitative analysis of client's experiences. Internet Interventions. 2017;10:29-38.
35. Sari S, Muller A, Roessler K. Exercising alcohol patients don't lack motivation but struggle with structures, emotions and social context – a qualitative dropout study. Family Practice. 2017; 18:45.
36. Close C, Sinclair M, McCullough J, et al. Factors affecting recruitment and attrition in ramdomised controlled trials of complementary and alternative medicine for pregnancy – related issues. Evidence-Based Complementary and Alternative Medicine. 2016 http://dx.doi.org/10.1155/2016/6495410.
37. Kearney A, Rosala-Hallas A, Bacon N, et al. Reducing attrition: the communication of retention and withdrawal within patient information sheets. Trials. 2017:18;(Suppl 1):P.489.
38. Gillies K, Skea ZC, Campbell MK. Decision aids for randomised controlled trials: a qualitative exploration of stakeholders' views. BMJ Open 2014;4:e005734. doi:10.1136/bmjopen-2014-005734.
39. Gamble C, Dudley L, Allam A, et al. Patient and public involvement in the early stages of clinical trial development: a systematic cohort investigation. BMJ Open 2014;4:e005234. doi:10.1136/bmjopen-2014- 005234.
40. Buck D, Gamble C, Dudley L, et al. From plans to actions in patient and public involvement: qualitative study of documented plans and the accounts of researchers and patients sampled from a cohort of clinical trials. BMJ Open 2014;4:e006400. doi:10.1136/bmjopen-2014- 006400.

For peer review only - http://bmjopen.bmj.com/site/about/guidelines.xhtml

41. Brueton VC, Tierney J, Stenning S, Harding S, Meredith S, Nazareth I, Rait G. Strategies to improve retention in randomised trials. Cochrane Database of Systematic Reviews 2013, Issue 12. Art. No.: MR000032. DOI: 10.1002/14651858.MR000032.pub2.
42. Donovan J, Rooshenas L, Jepson M et al. Optimising recruitment and informed consent in randomised controlled trials: the development and implementation of the Quintet Recruitment Intervention (QRI). Trials. 2016; 17:283. DOI 10.1186/s13063-016-1391-4.

For peer review only

For peer review only

For peer review only - http://bmjopen.bmj.com/site/about/guidelines.xhtml

S1 Appendix: Search strategies

Database: Embase <1996 to 2016 Week 32>, Ovid MEDLINE(R) In-Process & Other Non-Indexed Citations and Ovid MEDLINE(R) <1946 to Present>, Ovid MEDLINE(R) Epub Ahead of Print <August 12, 2016>
Search run 12th August 2 016
URL: https://ovidsp.ovid.com/

1 exp clinical trial/ (1703701)
2 randomi?ed control$ trial$.tw. (264645)
3 randomi?ed controlled trial?.kw. (16568)
4 clinical trial?.tw,kw. (607627)
5 controlled trial?.tw,kw. (348876)
6 controlled clinical trial?.tw,kw. (43965)
7 pragmatic trial?.tw,kw. (1460)
8 complex intervention?.tw,kw. (3707)
9 or/1-8 (2248337)
10 qualitative research/ (66175)
11 qualitative research.tw,kw. (24949)
12 (qualitative adj3 method$).tw. (40973)
13 (qualitative method? or qualitative methodology).kw. (1675)
14 (qualitative adj3 stud$).tw. (72849)
15 qualitative study.kw. (1485)
16 focus groups/ use ppez (20834)
17 focus group?.tw,kw. (65079)
18 grounded theory/ (3608)
19 grounded theory.tw,kw. (16970)
20 narrative analys?s.tw,kw. (1638)
21 process evaluation.tw,kw. (4300)
22 mixed method?.tw,kw. (18886)
23 mixed method$.mp. (19525)
24 mixed methodology.tw,kw. (527)
25 (in depth adj4 interview$).tw. (32578)
26 in depth interview?.kw. (99)
27 ((semi structured or semistructured) adj5 interview$).tw. (68020)
28 semi structured interview?.kw. (157)
29 qualitative interview$.tw. (13130)
30 qualitative interview?.kw. (273)
31 (interview$ and theme$).tw. (45139)
32 interview?.kw. (5082)
33 (interview$ and audio recorded).tw. (3208)
34 qualitative case stud$.tw. (1572)
35 descriptive case stud$.tw. (371)
36 qualitative case study.kw. (19)
37 descriptive case study.kw. (0)
38 qualitative exploration.tw,kw. (1371)
39 qualitative evaluation.tw,kw. (5109)
40 qualitative intervention.tw,kw. (20)
41 qualitative approach.tw,kw. (6196)
42 qualitative inquiry.tw,kw. (911)
43 qualitativ$ analys$.tw. (25050)

For peer review only

44 qualitative analysis.kw. (826)
45 (qualitative adj3 data).tw. (26159)
46 qualitative data.kw. (105)
47 discourse analysis.tw,kw. (2681)
48 discursive.tw,kw. (2686)
49 phenomenological.tw,kw. (24638)
50 thematic analysis.tw,kw. (16782)
51 ethnograph$.tw. (15000)
52 ethnography.kw. (1288)
53 action research.tw,kw. (6182)
54 ethno?methodology.tw,kw. (116)
55 social construction.tw,kw. (1443)
56 or/10-55 (336295)
57 phenomenological characteristics.tw,kw. (186)
58 phenomenological model.tw,kw. (1596)
59 action research arm test.tw,kw. (839)
60 protocol.ti. (60150)
61 or/57-60 (62747)
62 56 not 61 (331034)
63 9 and 62 (17965)
64 Patient Dropout/ use ppez (7280)
65 Patient Dropouts/ use emef (507)
66 Patient Recruitment/ use ppez (55744)
67 Research Subjects/ use emef (4237)
68 patient recruitment.kw. (144)
69 attrition.kw. (1063)
70 patient retention.kw. (25)
71 ((recruit$ or participat$ or take part or dropout$ or drop$ out$ or withdr?wl$ or barrier$ or retention or response$ or respond$ or attrition) adj10 trial?).tw. (113422)
72 or/64-71 (178421)
73 63 and 72 (2681)
74 limit 73 to english language (2656)
75 74 not abstract.pt. (2289)
76 exp animals/ not human/ (6693580)
77 exp nonhuman/ not humans/ (2882812)
78 75 not (76 or 77) (2284)
79 limit 78 to yr="2010 -Current" (1550)
80 remove duplicates from 79 (1001)

****
Database: PsycINFO <2002 to July Week 4 2016>
Search run 12th August 2016
URL: https://ovidsp.ovid.com/
----

1 clinical trials/ (9357)
2 randomi?ed control$ trial$.tw. (20733)
3 clinical trial?.tw. (23211)
4 controlled trial?.tw. (25131)
5 controlled clinical trial?.tw. (2000)

6 pragmatic trial?.tw. (151)
7 complex intervention?.tw. (481)
8 or/1-7 (47578)
9 qualitative research/ (6629)
10 qualitative research.tw. (13171)
11 (qualitative adj3 method$).tw. (16650)
12 (qualitative adj3 stud$).tw. (35238)
13 focus group?.tw. (22396)
14 grounded theory/ (2954)
15 grounded theory.tw. (10217)
16 narrative analys?s.tw. (1580)
17 process evaluation.tw. (924)
18 mixed method?.tw. (12748)
19 mixed methodology.tw. (604)
20 (in depth adj4 interview$).tw. (16870)
21 ((semi structured or semistructured) adj5 interview$).tw. (25412)
22 qualitative interview$.tw. (5962)
23 (interview$ and theme$).tw. (24528)
24 interview?.kw. (0)
25 (interview$ and audio recorded).tw. (811)
26 qualitative case stud$.tw. (3022)
27 descriptive case stud$.tw. (405)
28 qualitative exploration.tw. (698)
29 qualitative evaluation.tw. (586)
30 qualitative intervention.tw. (3)
31 qualitative approach.tw. (2572)
32 qualitative inquiry.tw. (1108)
33 qualitativ$ analys$.tw. (7974)
34 (qualitative adj3 data).tw. (13626)
35 discourse analysis/ (5598)
36 discursive.tw,kw. (6443)
37 phenomenological.tw. (15930)
38 thematic analysis.tw. (5668)
39 ethnograph$.tw. (17608)
40 action research.tw. (5273)
41 ethno?methodology.tw. (303)
42 social construction.tw. (2467)
43 or/9-42 (166675)
44 phenomenological characteristics.tw. (97)
45 phenomenological model.tw. (105)
46 action research arm test.tw. (90)
47 protocol.ti. (1818)
48 or/44-47 (2107)
49 43 not 48 (166225)
50 experimental attrition/ (269)
51 experimental recruitment/ (25)
52 experimental subjects/ (2201)
53 dropouts/ (318)
54 ((recruit$ or participat$ or take part or dropout$ or drop$ out$ or withdr?wl$ or barrier$ or retention or response$ or respond$ or attrition) adj10 trial?).tw. (11492)
55 or/50-54 (13921)

For peer review only

56 8 and 49 and 55 (382)
57 exp animals/ not human/ (154642)
58 56 not 57 (382)
59 limit 58 to (english language and yr="2010 -Current") (243)

****

Cochrane Central Register of Controlled Trials : Issue 7 of 12, June 2016
Search run 12th August 2016
URL: http://www.cochranelibrary.com/

#1 MeSH descriptor: [Qualitative Research] this term only
#2 qualitative NEXT research:ti,ab,kw or qualitative NEXT method:ti,ab,kw or qualitative NEXT study:ti,ab,kw (Word variations have been searched)
#3 MeSH descriptor: [Focus Groups] this term only
#4 MeSH descriptor: [Grounded Theory] this term only
#5 mixed NEXT method:ti,ab,kw or narrative NEXT analysis:ti,ab,kw (Word variations have been searched)
#6 interview:ti,ab,kw (Word variations have been searched)
#7 qualitative case study:ti,ab,kw or descriptive case study:ti,ab,kw (Word variations have been searched)
#8 qualitative NEXT exploration:ti,ab,kw or qualitative NEXT evaluation:ti,ab,kw or qualitative intervention:ti,ab,kw or qualitative approach:ti,ab,kw or qualitative analysis: ti,ab,kw (Word variations have been searched)
#9 qualitative data:ti,ab,kw (Word variations have been searched)
#10 discourse analysis:ti,ab,kw or discursive:ti,ab,kw (Word variations have been searched)
#11 social construction:ti,ab,kw or action research:ti,ab,kw or ethnography:ti,ab,kw or thematic analysis:ti,ab,kw or phenomenological:ti,ab,kw (Word variations have been searched)
#12 #1 or #2 or #3 or #4 or #5 or #6 or #7 or #8 or #9 or #10 or #11
#13 MeSH descriptor: [Patient Dropouts] this term only
#14 MeSH descriptor: [Patient Selection] this term only
#15 ((recruit$ or participat$ or take part or dropout$ or drop$ out$ or withdr?wl$ or barrier$ or retention or response$ or respond$ or attrition) near/10 trial?):ti,ab,kw (Word variations have been searched)
#16 #13 or #14 or #15
#17 #12 and #16 Publication Year from 2010 to 2016, in Trials
#18 abstract:pt (Word variations have been searched)
#19 #17 not #18 (282)

Social Sciences Citation Index
Search run: August 12th 2016
Web of Knowledge: URL http://wok.mimas.ac.uk/

#33 232 #21 AND #24 AND #31 Refined by: DOCUMENT TYPES: (ARTICLE OR REVIEW) Indexes=SSCI Timespan=2010-2016
32 233 #21 AND #24 AND #31
31 4,991 #25 OR #26 OR #27 OR #28 OR #29 OR #30
30 615 (TS=patient dropout) AND LANGUAGE: (English)
29 568 (TS=patient attrition) AND LANGUAGE: (English)
28 2,040 (TS=patient retention) AND LANGUAGE: (English)

For peer review only

27 1,923 (TS=((withdraw$ or barrier$ or retention or response$ or respond$ or attrition) NEAR/10 trial?)) AND LANGUAGE: (English)
26 70 (TS=(("take part" or dropout$ or "drop$ out") NEAR/10 trial?)) AND LANGUAGE: (English)
25 220 (TS=((recruit$ or participat$) NEAR/10 trial?)) AND LANGUAGE: (English)
24 42,063 #23 OR #22
23 42,671 (TS=randomised controlled trial) AND LANGUAGE: (English)
22 42,671 (TS=randomized controlled trial) AND LANGUAGE: (English)
21 148,316 #20 OR #19 OR #18 OR #17 OR #16 OR #15 OR #14 OR #13 OR #12 OR #11 OR #10 OR #9 OR #8 OR #7 OR #6 OR #5 OR #4 OR #3 OR #2 OR #1
20 7,765 (TS=thematic analysis) AND LANGUAGE: (English)
19 16,672 (TS=action research) AND LANGUAGE: (English)
18 7,436 (TS=social construction) AND LANGUAGE: (English)
17 5,371 (TS=discursive) AND LANGUAGE: (English)
1611,763 (TS=discourse analysis) AND LANGUAGE: (English)
15 6,704 (TS=(qualitative near/1 data)) AND LANGUAGE: (English)
14 8,581 (TS=(qualitative near/1 analysis)) AND LANGUAGE: (English)
13 3,122 (TS=(qualitative near/1 approach)) AND LANGUAGE: (English)
12 122 (TS=(qualitative near/1 intervention)) AND LANGUAGE: (English)
11 688 (TS=(qualitative near/1 evaluation)) AND LANGUAGE: (English)
10 529 (TS=(qualitative near/1 exploration)) AND LANGUAGE: (English)
9 40,423 (TS=(in depth interview* or semi structured interview* or qualitative interview*)) AND LANGUAGE: (English)
8 7,414 (TS=narrative analysis) AND LANGUAGE: (English)
7 19,879 (TS=mixed method*) AND LANGUAGE: (English)
6 8,541 (TS=grounded theory) AND LANGUAGE: (English)
5 40,298 (TS=focus group*) AND LANGUAGE: (English)
4 2,062 (TS= (qualitative NEAR/1 studies)) AND LANGUAGE: (English)
#3 15,953 ((TS= (qualitative NEAR/1 study))) AND LANGUAGE: (English)
2 8,468 ((TS= (qualitative NEAR/1 method*))) AND LANGUAGE: (English)
1 12,460 (TS= (qualitative NEAR/1 research)) AND LANGUAGE: (English)

Cumulative Index of Nursing & Allied Health Literature
Search run 12th August 2016
URL: http://search.ebscohost.com/

S18 S7 AND S16 Limiters - Published Date: 20100101-20161231 Narrow by Language: - English (167)
S17 S7 AND S16
S16 S8 OR S9 OR S10 OR S11 OR S12 OR S13 OR S14 OR S15
S15 TX discourse analysis OR TX discursive OR TX thematic analysis OR TX ethnography OR TX action research OR TX phenomenological
S14 TX qualitative exploration OR TX qualitative evaluation OR TX qualitative intervention* OR TX qualitative approach OR TX qualitative analysis OR TX qualitative data
S13 TX mixed method* OR TX semi structured interview* OR TX in depth interview*
S12 TX focus group* OR TX grounded theory OR TX narrative analysis
S11 TX qualitative n3 research OR TX qualitative n3 method* OR TX qualitative n3 study
S10 (MH "Focus Groups")
S9 (MH "Semi-Structured Interview") OR (MH "Structured Interview") OR (MH "Narratives")
S8 (MH "Qualitative Studies+")

S7 S3 AND S6
S6 S4 OR S5
S5 TX ((recruit$ or participat$ or take part or dropout$ or drop$ out$ or withdr?wl$ or barrier$ or retention or response$ or respond$ or attrition) N10 trial?)
S4 (MH "Research Subjects+")
S3 S1 OR S2
S2 TX ranndomized or randomised or trial*
S1 (MH "Clinical Trials+")

Applied Social Sciences Index and Abstracts
Search run 12th August 2016
http://search.proquest.com/assia/

S8 S5 and S6Limits applied Language:English PY: 2010-2016 (37)
S7 S5 and S6
S6 (recruit* N/10 trial?) OR (participat* N/10 trial? OR "take part" N/10 trial?) OR (dropout* N/10 trial? OR drop* our* N/10 trial?) OR (withdraw* N/10 trial* OR barrier* N/10 trial?) OR (retention N/10 trial? OR response* N/10 trial?) OR (respond* N/10 trial? OR attrition N/10 trial?)
S5 S3 and S4
S4 qualitative OR (focus group* OR interview*) OR (mixed method* OR ethnography) OR (phenomenological OR discourse analysis) OR discursive
S3 S1 or S2
S2 randomized OR randomised
S1 SU.EXACT("Clustor randomized trials") OR SU.EXACT("Clinical randomized controlled trials") OR SU.EXACT("Single blind randomized controlled trials") OR SU.EXACT("Cluster randomized controlled trials") OR SU.EXACT("Randomized controlled trials") OR SU.EXACT("Double blind randomized trials") OR SU.EXACT("Prospective controlled trials") OR SU.EXACT("Double blind randomized controlled trials") OR SU.EXACT("Clinical trials")

For peer review only

S1 Box Steps of Meta-ethnography

1) Identifying the research question

This stage involved us generating a research question specific to our area of interest that we believed could be usefully addressed by referring to qualitative research. In our case this was the question of what influences non-retention within clinical trials from the perspectives of trial withdrawers?

2) Identifying relevant studies

This stage involved making a series of decisions relating to deciding what was relevant to our initial area of interest, deciding on the searching process, inclusion/exclusion decisions and quality assessment. In our case we were interested in any study that reported the use of qualitative methods (for collection and analysis of data) to explore the reasons why individual participants withdraw from clinical trials. We were interested in any reports made by participants themselves or by trial staff, but this had to be specifically in relation to why participants withdraw. We defined withdrawal or non-retention as covering any aspect of attrition recognising that this might cover activities such as cessation of, or withdrawal from the intervention(s), non-attendance at clinic visits, through to non-response to some or all follow up questionnaires etc. We decided that we would exclude studies that did not use qualitative data to collect or analyse their data and also studies reporting findings from trial withdrawers who were not patient participants e.g. GPs in a primary care cluster trial. Following these decisions, a systematic search across a range of databases was conducted with assistance from an information specialist (See S1 Appendix) and all titles and abstracts were screened for inclusion (see S1 Figure). Applying quality criteria to qualitative research remains a contentious issue and there is no consensus regarding whether and how this should be done. However, one author (ZS) undertook a quality assessment of each of the 8 papers that were identified as being eligible for inclusion in the synthesis. Whilst authors of some qualitative evidence syntheses have chosen to exclude what they deem to be poor quality papers, we made the decision not to exclude any of the identified papers. Although all papers had study aims that were amenable to investigation via qualitative means and all included qualitative data, as a team we deemed some as being richer than others in terms of data and insights (i.e. first and second order constructs). Despite this variation in the overall level of quality, due to the small number of identified studies we considered it more important to retain any relevant findings than disregard based on study quality. In doing so, we would argue that all 8 papers contributed useful elements to the collective whole and enabled us to develop our line of argument in terms of the issues of importance regarding trial non-retention.

3) Reading the studies

At this stage, we aimed to become as familiar as possible with the content of all the identified papers with each author independently reading through the data provided and making detailed notes of their observations including identification of preliminary themes. After sharing notes, we met to discuss our findings as a team, comparing and contrasting our preliminary observations etc.

4) Identifying themes

During this next stage (which in practice we found very much related to activities undertaken as part of stage 3), data was then extracted from all 8 papers using a standard form which summarised the main themes, information regarding methods, and any other important information relating to the context of the research (some of this data is illustrated in S1 Table). During this stage, we focussed on both 1st order constructs within included papers (meaning study participant quotations found in

For peer review only - http://bmjopen.bmj.com/site/about/guidelines.xhtml

the results section of papers) along with 2nd order constructs (meaning the interpretations made by the papers' authors, usually found in the discussion and conclusion sections of papers but also sometimes within the results). Using the standard form, the papers were initially organised in chronological order (but as inductive analysis progressed papers were grouped according to emerging themes) and we focused on the findings, concepts and themes used by the papers' authors generating a list of key categories. This document (along with our other written notes and observations) facilitated discussions at a series of subsequent team meetings and were very useful for consideration of how identified themes from one paper might relate to the others.

5) Translating the findings of each study into those of the others

At this key stage (which again in practice we found inter-related to stage 4), we sought to consider the extent to which themes seemed common or distinct across the papers. Our initial grouping of first and second-order constructs across the 8 papers resulted in 14 sub-themes. During the process of translating themes from each of the individual studies into those of the others (i.e. comparing and contrasting across studies), following further team discussion these were then grouped and categorised into 5 broad key themes which we interpreted as characterising the main considerations and features that appeared to influence non-retention in the trials under investigation (See S2 Table).

6) Synthesising the findings

For our synthesis, we considered and discussed both the 1st and 2nd order constructs across each of the papers in an attempt to develop a more nuanced and collective understanding of factors influencing trial non-retention (developing our 'line of argument'). As before, we did this through a process of reflection and team discussions, in an attempt to produce overarching insights into the factors that appear to influence non-retention.

7) Expressing the synthesis

As is common with other meta-ethnographies we sought to express our collective insights in both textual and diagrammatic format within our paper. In doing so, we expressed our synthesis both within our paper as our 'line of argument' (with supportive illustrative data from across the studies) and also as a conceptual diagram (see S2 Figure).

For peer review only

Enhancing transparency in reporting the synthesis of qualitative research: ENTREQ

ENTREQ Statement: content and rationale

The ENTREQ statement consists of 21 items grouped into five main domains: introduction, methods and methodology, literature search and selection, appraisal, and synthesis of findings (Table 1). For each item, a descriptor and examples are provided. Below we present a rationale for each domain and its associated items.

Table 1

Enhancing transparency in reporting the synthesis of qualitative research: the ENTREQ statement

No	Item	Guide and description	
**1**	Aim	State the research question the synthesis addresses.	See Page 1
**2**	Synthesis methodology	Identify the synthesis methodology or theoretical framework which underpins the synthesis, and describe the rationale for choice of methodology *(e.g. meta-ethnography, thematic synthesis, critical interpretive synthesis, grounded theory synthesis, realist synthesis, meta-aggregation, meta-study, framework synthesis).*	See Pages 1-3 and S1 Box
**3**	Approach to searching	Indicate whether the search was pre-planned (*comprehensive search strategies to seek all available studies)* or iterative (*to seek all available concepts until they theoretical saturation is achieved)*.	See Page 2
**4**	Inclusion criteria	Specify the inclusion/exclusion criteria *(e.g. in terms of population, language, year limits, type of publication, study type).*	See Page 2
**5**	Data sources	Describe the information sources used (e.g. *electronic databases (MEDLINE, EMBASE, CINAHL, psycINFO, Econlit), grey literature databases (digital thesis, policy reports), relevant organisational websites, experts, information specialists, generic web*	See Page 2

No	Item	Guide and description	
		searches (Google Scholar) hand searching, reference lists) and when the searches conducted; provide the rationale for using the data sources.	
**6**	Electronic Search strategy	Describe the literature search *(e.g. provide electronic search strategies with population terms, clinical or health topic terms, experiential or social phenomena related terms, filters for qualitative research, and search limits)*.	See Page 2 and Appendix 1
**7**	Study screening methods	Describe the process of study screening and sifting *(e.g. title, abstract and full text review, number of independent reviewers who screened studies).*	See Page 2
**8**	Study characteristics	Present the characteristics of the included studies *(e.g. year of publication, country, population, number of participants, data collection, methodology, analysis, research questions).*	See Page 3-4
**9**	Study selection results	Identify the number of studies screened and provide reasons for study exclusion *(e,g, for comprehensive searching, provide numbers of studies screened and reasons for exclusion indicated in a figure/flowchart; for iterative searching describe reasons for study exclusion and inclusion based on modifications t the research question and/or contribution to theory development).*	See Page 3 and Figure 1
**10**	Rationale for appraisal	Describe the rationale and approach used to appraise the included studies or selected findings *(e.g. assessment of conduct (validity and robustness), assessment of reporting (transparency), assessment of content and utility of the findings).*	See Page 2-3
**11**	Appraisal items	State the tools, frameworks and criteria used to appraise the studies or selected findings *(e.g. Existing tools: CASP, QARI,*	See Page 3

No	Item	Guide and description	
		COREQ, Mays and Pope [25]*; reviewer developed tools; describe the domains assessed: research team, study design, data analysis and interpretations, reporting).*	
**12**	Appraisal process	Indicate whether the appraisal was conducted independently by more than one reviewer and if consensus was required.	See Page 3. 1 reviewer (the main author) initially assessed quality of included studies using the CASP criteria and noted any critical aspects of quality with the study team. During subsequent group discussions we continued to discuss and reflect on key aspects of quality.<br>Due to the small number of eligible studies we decided to include all (please see Pages 9-10.)
**13**	Appraisal results	Present results of the quality assessment and indicate which articles, if any, were weighted/excluded based on the assessment and give the rationale.	See Pages 11
**14**	Data extraction	Indicate which sections of the primary studies were analysed and how were the data extracted from the primary studies? *(e.g. all text under the headings "results /conclusions" were extracted electronically and entered into a computer software).*	See Page 2 and S1 Box
**15**	Software	State the computer software used, if any.	N/A
**16**	Number of reviewers	Identify who was involved in coding and analysis.	See Pages 2
**17**	Coding	Describe the process for coding of data *(e.g. line by line coding to search for concepts).*	See Page 2
**18**	Study comparison	Describe how were comparisons made within and across studies *(e.g. subsequent studies were coded into pre-existing concepts, and new concepts were created when deemed necessary).*	See Page 2 and S1 Box

No	Item	Guide and description	
**19**	Derivation of themes	Explain whether the process of deriving the themes or constructs was inductive or deductive.	See Page 2 and S1 Box
**20**	Quotations	Provide quotations from the primary studies to illustrate themes/constructs, and identify whether the quotations were participant quotations of the author's interpretation.	See Results section and S2 Table
**21**	Synthesis output	Present rich, compelling and useful results that go beyond a summary of the primary studies (e.g. *new interpretation, models of evidence, conceptual models, analytical framework, development of a new theory or construct).*	See Results and discussion section. Also see our conceptual model illustrating our 'line of argument' (S2 Figure).

For peer review only

PRISMA Flow Diagram: Retention Review

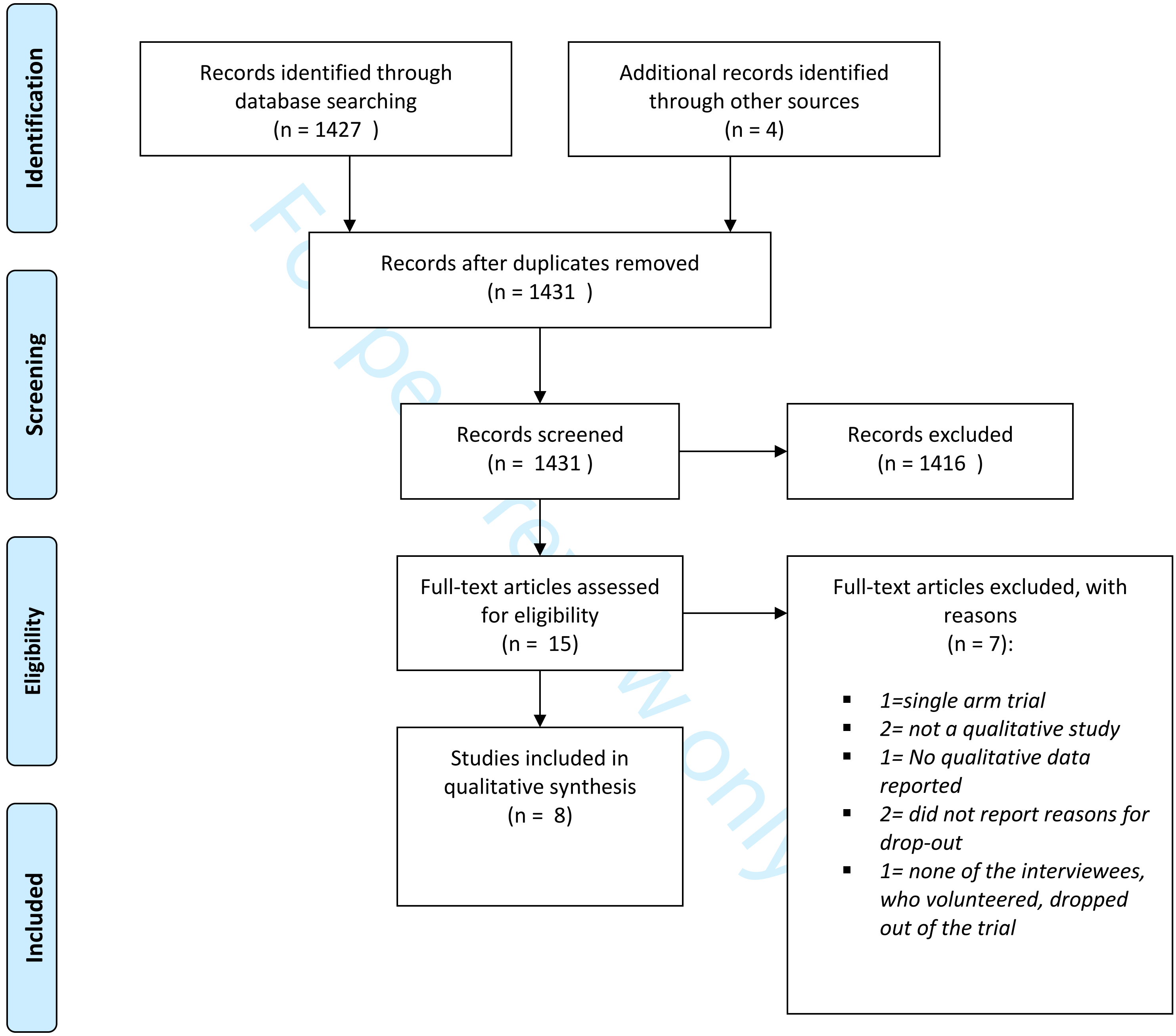

S1 Table Characteristics of included studies

REF	COUNTRY	AIM	CONDITIONS OF FOCUS	PARTICIPANT CHARACTERISTICS	TRIAL COMPARATORS	ATTRITION BEHAVIOUR	DATA COLLECTION METHODS
Nakash et al 2007 [28]	UK	To examine factors affecting response and non-response from the clinical trial participant's perspective.	Severe ankle sprains	8 non-retainers. Age and gender of non-retainers unclear although of the 22 included in this study, 11 were male; 11 female, aged from 16 to 62 yrs (mean age 34 yrs). A purposive sample was sought to represent the diversity of trial participants in age, sex, level of education, occupation and type of ankle support.	Different mechanical supports	Non-response to postal questionnaire follow-up. 8 had not responded to at least one of their follow up questionnaires.	Semi-structured interviews
Nicholas et al 2010 [21]	Australia	To identify participants' reasons for non-adherence to, and attrition from the online intervention	Newly diagnosed bipolar disorder	39 non-retainers. 22 female; 17 male. 20 were aged less than 30 yrs, 14 were married, 29 were tertiary educated and 24 in full time employment. Participants from all 3 study groups were interviewed, 16 from the unsupported intervention group (BEP), 9 from the supported BEP intervention group (BEP+IS), and 14 from the minimal	1 of 2 active interventions (online psycho-education program either alone or with email support from informed supporters) or an attention control condition (online information about bipolar disorder presented in text as bullet points). Both active interventions and control contained 'workbook' activities.	Cessation of therapy sessions and/or non-completion of some or all intervention workbooks; non-completion of some or all control workbooks.	Semi-structured interviews

For peer review only

S1 Table Characteristics of included studies

				information control group.			
Postel et al 2010 [22]	Netherlands	To evaluate an e-therapy program with active therapeutic involvement for problem drinkers. Reasons for drop out were also investigated via a 'dropout' questionnaire consisting mainly of open questions.	Problem drinking	40 non-retainers. No gender or age details provided specifically for non-retainers, although 53.8 % of trial participants were female, mean age 45.3 yrs. Authors also state that in the control group more non-responders than responders were male.	3 month e-therapy programme (consisting of a structured 2 part online treatment programme with asynchronous therapist contact via the internet only) or a waiting list control group (receiving 'no reply' email messages once every 2 weeks). Participants completed online self-report questionnaires at baseline and at 3 months follow up (control group) or at posttreatment, which was approximately 3 months (e therapy group). Weekly alcohol consumption was assessed by a 7 day retrospective drinking diary.	Non-completion of treatment sessions and/or follow-up questionnaires. Non-retention was defined as anyone who did not complete the 3 month assessment. Dropouts in the e therapy group did not complete all 12 treatment sessions: 9 assignments and 3 assessments.	A 'dropout' questionnaire consisting mainly of open questions.
Eborall et al 2011 [27]	UK	To explore people's explanations for declining to participate in the trial, or, having begun the trial, stopping the trial medication	Asymptomatic atherosclerosis	17 non-retainers. No gender or age details provided specifically for non-retainers (as opposed to those who declined to consent), but states that total sample (n=28) had a mean age of 65.2 yrs; 19 were female; wide range of socioeconomic	Aspirin (100mg daily) or placebo for a mean duration of 8.2 yrs.	Cessation of trial medication (unclear whether active medication or placebo). Non-adherence with study medication throughout the trial was 40%; 15% took their medication for less than 6 months.	Semi-structured interviews (n=11) and one focus group (n=6)

S1 Table Characteristics of included studies

				backgrounds.			
Wells et al 2011 [23]	USA	To explore low-income, minority cancer patient perspectives about not adhering or dropping out of depression treatment.	Depression and cancer	20 non-retainers. No gender or age details provided specifically for non-retainers included in the qualitative study but trial non-retainers were described as predominantly female, foreign born, unmarried, unemployed, and older than 50 yrs.	Intervention or usual care. Intervention was an individualised stepped care depression programme provided by a cancer depression clinical specialist in collaboration with a study psychiatrist. Patients in the intervention group were offered antidepressant medication and/or problem solving treatment (PST).	Cessation of problem solving treatment sessions and/or anti-depressant medication.<br><br>PST dropouts were defined as patients who had fewer than 4 PST sessions. PST dropouts included those who initially agreed to be randomised to the intervention, but thereafter had either verbally declined treatment or did not show up for the therapy appointments. This included patients who had refused some sessions, but agreed to remain in the study for outcome interviews.<br><br>Patients receiving antidepressant medication were dropouts if they discontinued treatment within 30 days.	Semi-structured interviews
Shilling et al 2011 [25]	UK	To investigate recruitment processes across a range of clinical trials and from the perspective of parents, young people and practitioners to identify strategies to improve recruitment	Trial 1: Neuro-development disorders<br><br>Trial 2: Osteopenia	3 non-retainers from 2 of the 4 included trials. In trial 1, participants were 11-14 yrs; In trial 2, participants were 4-18 yrs (although demographic details of non-retainers unclear).	Trial 1: Melatonin versus placebo (over 12 week treatment period). Families made 3 additional hospital visits, and received 4 home visits by the research nurse and 3 telephone calls.<br><br>Trial 2: bisphosphonate risedronate or vitamin D	Cessation of trial medication (unclear whether active medication or placebo)	Semi-structured interviews (with the young people and/or their parents).

For peer review only

S1 Table Characteristics of included studies

		and its conduct across a spectrum of trials of medicines for children.<br><br>NB: This was a monograph that included the reporting of a range of trials, two of which reported data on non-retainers and were included in this meta-ethnography.			analogue 1 – alphahydroxychol ecalciferol versus placebo (1 yr treatment period). Young people were seen 7 times over the year. This was timed to coincide with routine clinic visits where possible. Blood samples were also taken (same time as routine visits) and they gave regular urine samples and had 3 x-ray scans and 2 bone radiographs.		
Sanders 2012 [26]	UK	To explore barriers to participation and adoption of tele-health and tele-care from the perspective of people who declined to participate or withdrew from the trial	Diabetes, COPD, heart failure, or social care needs	3 non-retainers (all in the intervention arm), 1 female (diabetes); 2 male (1 x COPD; 1 x diabetes + heart and lung problems. 73-85 yrs.	The RCT was a cluster design with GP practices being randomised to receive access to telehealth or telecare for their populations. Participants randomised to the control arm were offered telehealth or telecare at the end of the 12mth trial.<br><br>Tele-health equipment included a monitor unit via which recordings from peripheral devices were uploaded to a monitoring centre. The monitoring centres prioritised and tailored response according to need based	Cessation of use of telehealth equipment or tele care devices (defined as withdrawing from the trial after joining the intervention arm).	Semi-structured interviews

S1 Table Characteristics of included studies

					on the information received.<br><br>Telecare interventions also varied according to assessed need but included various sensors to detect gas, water overflow, falls and movement around the property. Such sensors would trigger alarms direct to a monitoring centre if anything abnormal was detected, allowing emergency intervention.		
Johansson 2015 [24]	Sweden	To explore participants' experiences of non-adherence to internet-delivered psychological treatment	Generalised anxiety disorder	7 non-retainers, 6 female; 1 male; mean age 39.3 yrs	Intervention consisted of internet delivered psychological treatment, with weekly support from a licensed clinical psychologist. The treatment consisted of 8 weekly self-help modules of text, audio and illustrations, averaging 21 pages per module. All modules contained a homework assignment that needed to be answered and sent to the guiding therapist.<br>No details re. control group given.	Non-adherence to internet delivered psychological treatment.<br><br>Non-adherence was defined as completing at least 1 and no more than 7 treatment modules (out of 8 in total).	Semi-structured interviews

For peer review only

S1 Table Characteristics of included studies

For peer review only

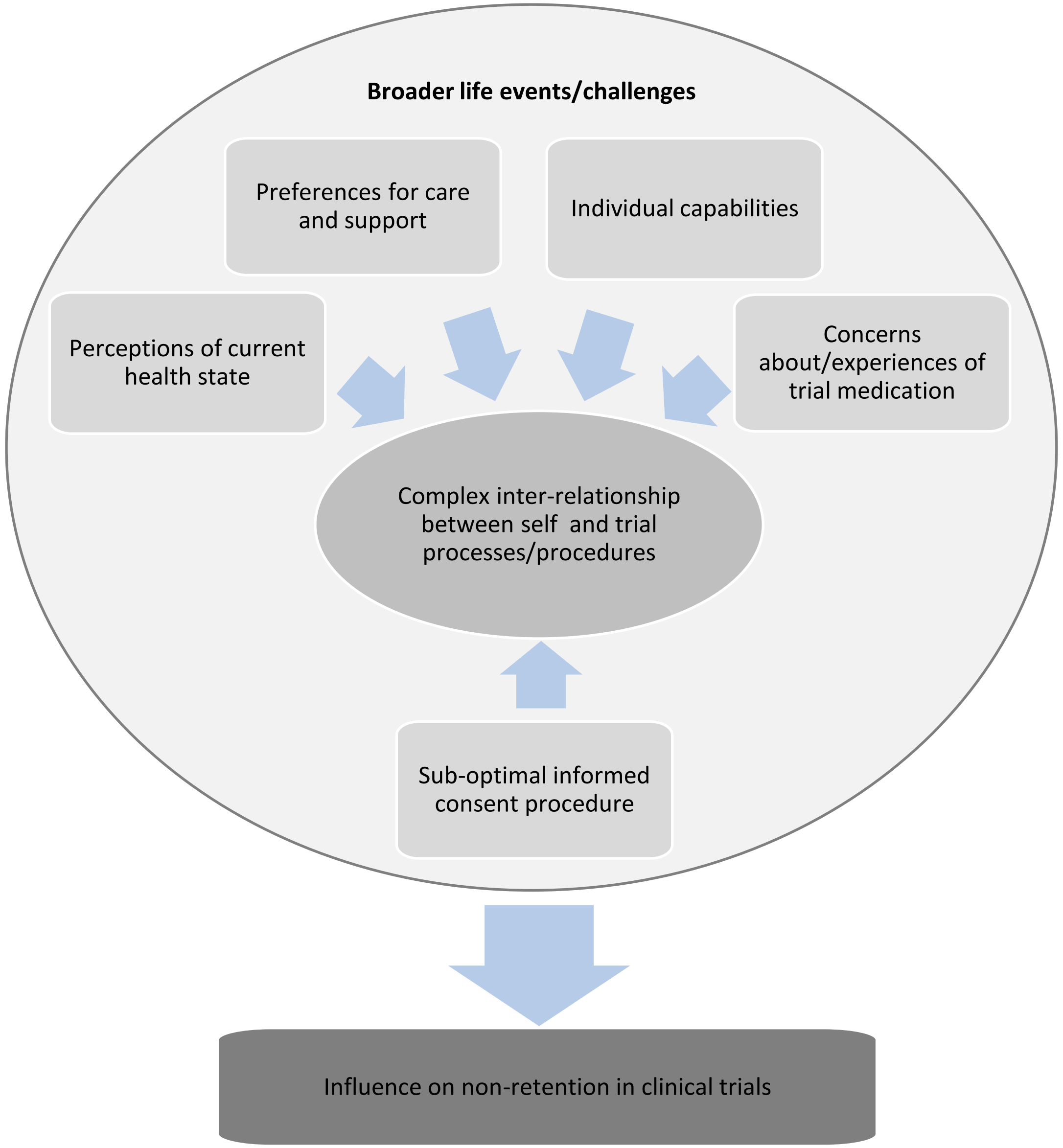
Broader life events/challenges
Preferences for care and support
Individual capabilities
Perceptions of current health state
Concerns about/experiences of trial medication
Complex inter-relationship between self and trial processes/procedures
Sub-optimal informed consent procedure
Influence on non-retention in clinical trials

S2 Table Key themes of influence on decisions to withdraw from trial participation, with corresponding example data.

Theme	Exemplary quote
**Perceptions of current health state in relation to specific aspects of the trial**	*"A very short while after doing the program I fell into another episode, a depressive episode, and pretty much stopped doing everything, the program included"[21, male, 18-29 yrs, BEP+IS group]*<br><br>*"I found it quite confronting, and reading the information made me feel uncomfortable, thinking that these issues related to me – I preferred the ostrich approach" [21, male, 40-49 yrs, BEP group]*<br><br>**This study demonstrates that reasons…were often explained in terms of potential threats to existing self-care, independence, the majority of respondents in this study depicted themselves as too healthy and too independent for the interventions to be of value [26, telehealth and telecare interventions in aging populations]**
**The 'fit' of aspects of the trial with individual preferences for care and support**	**Two respondents who withdrew from the trial described how the service changes they experienced caused additional stress. For example, one woman said she** *'did not want to be a nurse' …***and she was much happier to have returned to a regular appointment (fortnightly) with the community matron. Another man described the good care he received prior to joining the trial, but how he was subsequently discharged from the specialist professionals who had been involved in his care…..he described his main problems as** *'complex problems with my heart and breathing,'* **and that the faulty recordings and changes in service provision were causing him great stress [26, telehealth and telecare interventions in aging populations]**<br><br>**Some participants changed to other treatment formats after terminating the Internet-delivered therapy. One participant explained that she had never prioritised her own personal development and that an individual therapy consisting of face to face meetings was needed to get away from home and focus on the therapy [24, internet-delivered psychological treatment for people with generalised anxiety disorder]**
**The compatibility of aspects of the trial with individual capabilities**	**Twelve of the 20 patients interviewed acknowledged that they had dropped out of treatment, citing several reasons and circumstances related to dropping out of treatment. These included…cultural (which included language communication problems) [23, problem solving**

	**treatment sessions and/or anti-depressant medication for people with depression and cancer]**<br><br>**Difficulty with literacy [28, Different mechanical supports for people with severe ankle sprains]**<br><br>**One critique was that information was difficult to understand because the content was perceived as complex and abstract. In some cases the participants felt unintelligent for their inability to understand [24, internet-delivered psychological treatment for people with generalised anxiety disorder]**
**Concerns about or experiences of trial medication**	**One mother's discovery, via an internet search done by the child's father, that the trial drug was unlicensed for children had left her concerned about the safety of the trial and she subsequently withdrew her child from MENDS [trial] [25]**<br><br>*"I really felt I'd got gall bladder trouble again because [the pain] was from here right through into me kidneys and really severe. So I went to my GP, and she just checked round and said straight away, 'don't take anymore, and ring [the trial] and tell them'"[27, Aspirin for people with Asymptomatic atherosclerosis]*
**Considerations around extent to which trial participation could be appropriately accommodated into broader life circumstances**	**The participants' statements regarding non-adhering showed an incompatible relationship between the length of the weekly text modules and factors or conditions in the personal life of the participants [24, internet-delivered psychological treatment for people with generalised anxiety disorder]**<br><br>*"…as a student you read so much already. I felt like I couldn't muster more energy or more time to spend by the computer and to read 10 or 20 more pages and also answer questions. It felt as if you were inclined to have a very structured life already to handle that" [24, internet-delivered psychological treatment for people with generalised anxiety disorder]*<br><br>**These were reasons such as pregnancy, exams, or work commitments which participants felt were the cause of their non-response [28 Different mechanical supports for people with severe ankle sprains]**<br><br>**We could establish that in the e-therapy group 11 participants dropped out because of personal**

For peer review only

	**reasons unrelated to the e-therapy program or the study (eg, ill family member) [22]**

Primary study participant quotes (1st order constructs) are displayed in italics and primary study author interpretations (2nd order constructs) are presented in bold. Study references and details of participants/interventions where available have been added to the end of exemplar quotes.

For peer review only

Exploring non-retention in clinical trials: A meta-ethnographic synthesis of studies reporting participant reasons for drop out

Journal:	*BMJ Open*
Manuscript ID	bmjopen-2018-021959.R1
Article Type:	Research
Date Submitted by the Author:	11-Sep-2018
Complete List of Authors:	Skea, Zoë; Universit of Aberdeen, Health Services Research Unit<br>Newlands, Rumana ; University of Aberdeen College of Life Sciences and Medicine, Health Services Research Unit<br>Gillies, Katie; University of Aberdeen, Health Services Research Unit
**Primary Subject Heading**:	Health services research
Secondary Subject Heading:	Qualitative research
Keywords:	QUALITATIVE RESEARCH, Clinical trials < THERAPEUTICS, Retention

Exploring non-retention in clinical trials: A meta-ethnographic synthesis of studies reporting participant reasons for drop out

Author: Dr Zoë Christina Skea,

Corresponding author: Dr Zoë Christina Skea, Health Services Research Unit, 3rd Floor, Health Sciences Building, University of Aberdeen, Aberdeen, AB25 2ZD, Tel: +44 (0) 1224 438153. email:z.skea@abdn.ac.uk. Fax: +44 (0) 1224 438165.

Co-authors: Dr Rumana Newlands, University of Aberdeen, Aberdeen, UK. Dr Katie Gillies, University of Aberdeen, Aberdeen, UK.

Key words: Qualitative research; Clinical Trials; Retention.

Word count: 8940

Abstract

Objectives

To undertake a meta-ethnographic synthesis of findings from primary qualitative studies that have explored participant reported factors influencing non-retention within a clinical trial context.

Design

A systematic search and meta-ethnography was conducted for published papers (from 1946 –July 2018) that contained qualitative data from trial non-retainers.

Participants

We identified 11 qualitative studies reporting data from 13 trials. The studies were undertaken between 2008 and 2015. Each study included between 3 and 40 people who had dropped out from a trial, with findings from 168 people in total reported across the papers.

Results

Emergent from our synthesis was the significance of trial non-retainers' perceptions around the personal 'fit' of key aspects of the trial with their personal beliefs, preferences, capabilities or life circumstances. These related to their own health state; preferences for receiving trial 'care'; individual capabilities; beliefs about or experiences of trial medication; and considerations whether trial participation could be accommodated into their broader lives. All these factors raise important issues around the extent to which initial decisions to participate were fully informed.

Conclusions

To improve retention in clinical trials, researchers should work to reduce the burden on trial participants both through the design of the intervention itself as well as through simplified data collection processes. Providing more detail on the nature of the trial interventions and what can be expected by 'participation' at the consenting stage may prove helpful in order to manage expectations.

Strengths and limitations of this study

For peer review only - http://bmjopen.bmj.com/site/about/guidelines.xhtml

- Trial retention has recently been identified as one of the top three priorities for methodological research by UK trialists.

- Within the context of clinical trials, issues around retention have not received equal scrutiny compared to methodological questions about trial recruitment despite being arguably just as important for trial validity.

- Understanding the complex reasons why trial participants leave a trial after initially consenting is important if trialists are to be able to design effective intervention strategies to address the problem.

- To our knowledge this is the first synthesis of key findings from qualitative studies exploring participants' perspectives of trial non-retention which provides learning across their collective contributions.

- Our synthesis only included 11 eligible papers reporting findings across 13 trials, 5 of which were set within a mental health context and all of which were conducted in high-income countries. This could have issues for the transferability of findings.

Introduction

Randomised controlled trials are integral for evidenced based clinical decision making. Within the context of clinical trials, the focus of much methodological research in recent years has been on issues specifically relating to trial recruitment, including significant investigation into how to increase the numbers of prospective participants recruited [1,2]. A key focus of much of this research has been on trial participants' perspectives and experiences particularly around why they do or do not choose to consent to participate in clinical trials [3,4,5,6,7,]. Whilst issues relating to trial recruitment are undoubtedly important, issues around retention (i.e. ensuring that trial participants remain in the trial to provide primary outcome data) have not received equal scrutiny in the literature despite being arguably just as important for trials in terms of ensuring that research questions are adequately answered [2].

Trial retention was recently identified in the top three priorities for methodological research by UK trialists [8]. Most trials experience the issue of missing data often referred to as a 'loss to follow-up', 'attrition' or 'drop out' and this can bias the findings of a trial. Some recent quantitative surveys have identified participant characteristics (e.g. age, gender, physical or mental health) or trial processes (e.g. study duration or length and relevance of outcome measures) as being potential

For peer review only - http://bmjopen.bmj.com/site/about/guidelines.xhtml

predictors of trial retention [9,10,11]. However, these studies are small in size, often limited to a particular clinical context, and the items included in the surveys are often identified by researchers rather than asking participants what items should be included. In addition, they lack any in depth exploration of the relevant issues affecting why participants withdraw, as reported by participants.

Understanding the complex reasons why trial participants leave a trial (either actively (e.g. by requesting no further follow up or purposefully not returning data) or passively (e.g. forgetting to return a questionnaire or attend a clinic visit)) after initially consenting to participation is important especially if those reasons are modifiable. This understanding of participant perspectives then becomes crucial if trialists are to be able to design effective intervention strategies to address the problem.

The approach of conducting in-depth qualitative research within the context of clinical trials is considered particularly useful for improving the evidence base for how trialists conduct them [12]. Indeed this approach has been used widely to explore perspectives on trial recruitment both in terms of primary qualitative studies and secondary syntheses. To our knowledge this is the first synthesis of key findings from studies exploring participants' perspectives of trial non-retention which provides learning across their collective contributions. Our aim was to undertake a meta-ethnographic synthesis of findings from primary qualitative studies and our specific research question was 'what influences non-retention in clinical trials'?

Methods

A systematic literature search and meta-ethnography was conducted (See S1 ENTREQ Checklist). This meta-ethnography was undertaken in two parts. Our original systematic search and synthesis was undertaken in August 2016. To integrate potentially more recent relevant research, we undertook an update in July 2018.

Meta-ethnography essentially involves an 'interpretive and inductive' approach to synthesising studies [13,14]. Essentially meta-ethnography involves the process of 'translating' the findings of individual qualitative studies so that they can be considered in relation to one another with the aim of identifying and building new conceptual knowledge on a particular topic [13,14]. The process of 'translating' findings across studies can be either 'reciprocal' or 'refutational' depending on how individual studies relate to each other [13].

Searching and identification of relevant studies

A systematic search was conducted for published papers that contained qualitative data about trial participants' reasons for not completing some or all of the processes involved in a clinical trial after initially consenting to take part (which we describe as constituting non-retention). Search strategies were informed by previous studies [12] and are provided in Appendix 1. Seven electronic databases were searched by an information specialist: Embase, Ovid MEDLINE, PsycINFO, Cochrane Central Register of Controlled Trials, The Social Sciences Citation Index (SSCI), Cumulative Index of Nursing & Allied Health Literature, and Applied Social Sciences Index and Abstracts (ASSIA) and covered papers

For peer review only - http://bmjopen.bmj.com/site/about/guidelines.xhtml

published from 1946 to August, 2016 (first search) and from October 2017 – July 2018 (updated search). Google Scholar and bibliographies of identified publications were also searched manually for additional potentially eligible papers.

For both searches, one author screened all titles and abstracts (RN for original search; ZS for update) with a second author (KG) screening a random 10% sample. Eligible studies included those that used qualitative methods and contained qualitative data exploring any aspect of non-retention from the perspective of patient participants (recognising that non-retention might cover activities such as cessation of or withdrawal from the intervention(s), non-attendance at clinic visits, through to non-response to some or all follow up questionnaires etc).

Analysis and synthesis

In order to collate and synthesise the available primary research, the seven steps of meta-ethnography as listed in S1 Box were followed. In summary, the three authors (ZS, RN, KG) each read and systematically extracted data from the included papers, shared notes and discussed study findings and interpretations during a series of group meetings. The papers were initially organised in chronological order (but as inductive analysis progressed papers were grouped according to emerging themes) and we focused on the findings, concepts and themes used by the papers' authors generating a list of key categories. We used a standard form which summarised the main themes, information regarding methods, and any other important information relating to the context of the research within each study (some of this data is illustrated in S1 Table). Although we initially organised papers chronologically in this table, we used it to facilitate a series of further group discussions around emerging issues and as inductive analysis progressed we grouped and discussed our data according to the 5 key emerging themes (See S3 Table)**.** In line with the process of undertaking a meta-ethnography, primary data or 'first order constructs' (quotations from study participants who had not completed any or some of the various trial processes) and authors' interpretations of these data ('second order constructs') were extracted, compared and contrasted between studies (enabling us to produce a 'reciprocal translation'), and organised into themes to facilitate the development of new insights or a 'line of argument' [13].

Study Quality

One author (ZS) undertook a quality assessment of each of the papers included in the synthesis. This was based on the Critical Appraisal Skills Programme (CASP) criteria [15] which was used to appraise the identified primary studies and consider their inclusion into the synthesis (See S1 CASP Checklist). Questions developed by the CASP have been used previously for appraising the quality of studies for inclusion in meta-ethnography [16,17,18,19,20].

Results

Description of Studies

The database search produced 1431 abstracts for the initial search and 697 abstracts for the update (see S1 Figure and S2 Figure for details). We only included studies that provided data about reasons for non-retention from the included study participants and/or in the authors' reflections. In all, 11

For peer review only - http://bmjopen.bmj.com/site/about/guidelines.xhtml

papers met our inclusion criteria (8 were identified from the initial search and 3 from the update). The focus and key study characteristics for the 11 included papers are outlined in S1 Table. The identified papers were conducted in 7 countries (UK, USA, Australia, Sweden, The Netherlands, Denmark and Spain) and discussed non-retention in 13 separate trials. Six of the papers focussed solely on reasons for non-retention [21,22,23,24, 25, 26], with the remaining 5 also considering reasons for consenting [27], non-consenting [28,29] and retention [30,31]. Each study included between 3 and 40 people who had dropped out from a trial, with findings from 168 people in total reported across the papers. As can be seen from S1 Table the setting of the trials in which the qualitative research was embedded included a range of clinical contexts such as: mental health problems [21,24,26]; mental health problems and cancer [23]; problem drinking [22, 25]; Type 1 diabetes [30]; diabetes, Chronic obstructive pulmonary disease, heart failure, or social care needs [28]; severe ankle sprains [31]; asymptomatic atherosclerosis [29]; neurodevelopment disorders [27], and osteopenia [27]. As expected, the clinical context differed as did the interventions under investigation and included: telehealth equipment or tele care devices [28]; web-based psycho-educational/cognitive therapy based support tools [21,22,24, 26]; anti-depressant medication and/or cognitive behavioural therapy [23]; exercise [25,30] various mechanical ankle supports [31]; aspirin [29]; melatonin [27]; and bisphosphonate risedronate or vitamin D analogue 1 – alphahydroxychol ecalciferol [27].

Findings were presented from trial non-retainers both before outcome data had been collected (e.g. those who withdrew from the intervention) and/or during the follow up when outcome data was being collected – in other words, papers included a mix in terms of non-retention behaviour (See S1 Table for a summary of non-retention behaviour i.e. non-adherence to intervention, non-return of questionnaires). For example, 8 studies reported aspects related to non-adherence to trial intervention: 3 of these reported cessation of trial medication [23,27 for both trials,29]; 5 reported cessation of treatment therapy sessions [21,22,23,24,26]; 1 reported cessation of use of telehealth equipment or tele health devices [28] and another reported non-completion of study workbooks [21]. Two studies reported non-return of follow-up questionnaires [22, 31]. For 2 studies, non-retention behaviour was unspecified [25,30] Three of the 11 studies appeared to have included only the views of those who had dropped out of the active intervention arms of the trial [23,24,28]. For 4 studies it was unclear whether data was from intervention or control groups [27 for both trials, 26,29,31,] and only 4 studies specifically stated that they included views of both those in the intervention and control groups [21,22,25,30].

Nine of the 11 studies used semi-structured interviews to collect data from people who had withdrawn from the main trial [21,23,24,25,26, 27,28,30 31]; 1 used a combination of focus groups and interviews[29] and another distributed a questionnaire that contained various open ended response options [23] (NB: only the qualitative data are reported and referred to in this paper). Although some papers provided gender, age and/or demographic details for participants taking part in the trial in question, as can be seen from S1 Table, this information was less comprehensive for those who had dropped out of the trial. Where participant characteristic information was provided in the original studies we have included this at the end of the quotes presented to illustrate findings.

Key themes from the synthesis

For peer review only - http://bmjopen.bmj.com/site/about/guidelines.xhtml

Our grouping of first and second-order constructs across the 8 initially identified papers resulted in 14 sub-themes. During the process of translating themes from each of the individual studies (i.e. comparing and contrasting across studies) these sub-themes were then grouped and categorised into 5 broad key themes which characterised the main considerations and features that appeared to influence non-retention in the trials under investigation (See S2 Table). For the 3 subsequently identified papers, we repeated the various stages of meta-ethnography - in essence comparing for 'fit' and checking for any additional themes [32,33]. For the update, we attempted to follow the 'extend and renovate the house' approach [33], which involves examining the newly included studies to establish whether they add new concepts or contribute to existing ones. During this process, we were confident that concepts identified in the later 3 papers supported and complemented our originally identified 5 key themes (from the original 8 studies) with no new concepts emerging.

These themes were: 1) Perceptions of current health state in relation to specific aspects of the trial; 2) the 'fit' of aspects of the trial with individual preferences for care and support; 3) the compatibility of aspects of trial processes with individual capabilities; 4) concerns about or experiences of trial medication; and 5) considerations around the extent to which trial participation could be appropriately accommodated into individuals' broader lives.

As these theme labels suggest, within them they accommodate a spectrum of views or experiences.

The 5 broad key themes identified as influencing participants' non-retention in clinical trials are illustrated with example data in S2 Table. In S2 Table, primary study participant quotes illustrating first order constructs are displayed in italics, and primary study author interpretations illustrating second order constructs are presented in bold text. In the rest of this paper, primary study participant quotes are displayed in italics.

Influences on participant non-retention in clinical trials: a line of argument

Expressed below is our 'line of argument' which is organised into themes to facilitate the development of cumulative insights (S3 Figure conceptually illustrates the line of argument developed from the synthesis). These themed influences appear to be weighed up during the participant's involvement in the trial and set alongside the complex inter-relationship between self and trial process/procedures and ultimately impact on their retention in the trial Overall, our argument emphasises the significance of trial participants' perceptions around the 'fit' of key aspects of the trial (intervention and trial processes) with their personal beliefs, preferences, capabilities or life circumstances. These factors (which were not necessarily mutually exclusive) related to beliefs about their own health state, preferences for how they wanted to receive care, their individual capabilities, beliefs about or experiences of trial medication, and also considerations around the extent to which trial participation could be appropriately accommodated (or not) into their broader lives. All of these were set against the overall backdrop of their balance between their sense of self and the trial processes and procedures – this providing the overarching explanation for the influence on retention in trials Implicit within several of these identified factors is the suggestion that there may have been deficits within the initial trial consenting process which led to participants (who subsequently withdrew) not being fully informed or at least not realising what the trial expected of

For peer review only - http://bmjopen.bmj.com/site/about/guidelines.xhtml

them and what they could expect of the trial. These findings are discussed in more detail below and arranged across 5 key themes.

1) Perceptions of current health state in relation to specific aspects of the trial.

This theme describes how aspects of the trial might not be right for people as individuals. For example, across 8 of the 11 studies a key influence on decisions to discontinue trial participation appeared to relate to perceptions of either being 'too well' to warrant further engagement with the trial [21,22,23, 25, 28,29,31] or struggling with the compatibility of aspects of the trial, particularly the interventions or ways outcomes were assessed, with their personal sense of self [21,22,25,28,29,30]. Conversely, other participants described periods of feeling too unwell to be able to engage appropriately in trial processes.

a) Being too well to engage further with trial processes

Some participants cited a belief that they had suitably recovered part way through a particular trial as a reason for discontinuing trial medication and/or problem solving treatment exercises [21,22,23, 25]:

"*I just don't want to be reminded of the alcohol thing, because I actually think it's over*" [25; Female, 30-68 years, Alcohol Use Disorder]

"Things really improved for me…I just felt really good and didn't really feel like I had that much to offer in regard to finding out more about it" [21; Female, 30-39 years, Bipolar disorder, control group]

"I have been sufficiently helped" [22; No gender/age details, Problem drinker, Intervention group]

Participants also cited recovery as a reason for not completing and returning all the required follow-up outcome assessment questionnaires [28, Severe ankle sprains] perhaps highlighting here the importance at the consenting stage of making sure participants are fully informed about the value of sustained engagement throughout the duration of the trial (even if they feel they are no longer personally benefiting from that engagement).

b) Lack of compatibility with personal sense of self

Sometimes reasoning around trial withdrawal related to participants' struggle to accommodate aspects of the trial with their personal sense of self at the time [25,28,29], suggesting that the intervention challenged their sense of self somehow. Again perhaps indicating the importance for initial trial recruitment consultations to include adequate discussions about the nature of the study intervention and also what will be expected of participants in terms of engagement with them. For example, a belief that they could self-manage or cope well enough without the need to engage with the trial support intervention [28; self –care intervention to facilitate support for self-management in aging populations]; a belief that they were too overweight and unfit to participate in a group exercise intervention [25; exercise intervention for people with alcohol use disorder] a belief that they had adequately managed their condition thus far without the need for any medication [29; aspirin for asymptomatic atherosclerosis] and also non-acceptance of a diagnosis amongst those

For peer review only - http://bmjopen.bmj.com/site/about/guidelines.xhtml

newly diagnosed [21; with bi-polar disorder; 30; with type 1 diabetes] as a reason for not relating to (or seeing any value in) the study interventions:

[Discussing the need to keep active rather than monitoring his health indoors using tele-health equipment] *"You've got [to have] the will power…if you can't do it I am finished. If I wouldn't have that I'd be, I'd be stuck inside here you know, and looking through the window like…I throw myself in the garden and everything. Everything I do I'm working on, I cook myself dinners and everything.'* [28; Male; 85yers; COPD]

"I think if it had been medication that I needed to take, I would have taken it" [29; Male; 72; stopped taking aspirin medication]

"If you're taking a lot, it knocks the hell out of your stomach…Given the choice, I'd rather not take medication full stop" [29; Male; 55; stopped taking trial medication for asymptomatic atherosclerosis]

"I wasn't ready to accept the illness. At that stage after diagnosis I wasn't willing to change my life according to the program." [21; Male; 18-29 yrs; Bipolar disorder, control group]

"Don't think it kind of really sank in as to what I'd been diagnosed with … It had kind of hit me and I wasn't really dealing with having it … [30; Female, 19-55yrs; Type 1 diabetes]

c) *Being 'too ill' to be able to engage appropriately with trial interventions*

Conversely, within all of the papers focussing on interventions for mental health conditions, and in 1 paper focussing on people newly diagnosed with Type 1 diabetes, participants described being 'too ill' to be able to engage appropriately in trial processes [21,23,24, 26,30]. Reasons discussed in this context related to feeling either too fragile, depressed, too manic, or too emotional/stressed at certain times to be able to complete the required intervention tasks (e.g. e-health intervention and associated workbook activities; cognitive behavioural therapy; taking blood samples) and also a concern that engagement with the intervention could act as a 'trigger' in terms of exacerbating anxiety symptoms:

"I was feeling that the therapy wasn't going to help me with my problems. I thought it could lead me to be even more anxious and that it wasn't going to be beneficial for me. So, I felt that I was going to waste my time if I continued" [26; no gender details, 21-59 yrs, people with a range of serious mental health problems]

"I did not cope with the exercises. I did them at the start but it gradually became more difficult to complete them…..particularly the breathing exercises. I got a bit dizzy and it increased my feelings of anxiety" [24, no gender or age details, generalised anxiety disorder]

"The biggest problem I have with my bipolar disorder is consistency; when I'm down I can't even brush my teeth or get up in the morning. So doing an education program with workbooks was beyond me" [21, Female, 18-29 yrs, Bipolar disorder, BEP group]

For peer review only - http://bmjopen.bmj.com/site/about/guidelines.xhtml

"I often go walking when having highs because I have to keep moving, so I didn't want to sit at a computer" [21, Male, 40-49 yrs, Bipolar disorder, BEP+IS group]

2) The 'fit' of aspects of the trial with individual preferences for care and support

Across 8 of the 11 studies another important influence in decisions to discontinue trial participation appeared to relate to the fit of aspects of the intervention with preferences for how participants wanted to receive care and support [21, 22,23,24,25,26,28,31], implicitly suggesting that the initial trial consenting process may have been sub-optimal in key ways. Participants in these trials discussed how aspects of the design of the interventions were not individualised or tailored enough to be helpful and others commented on interventions being either too technical, too physically demanding, too intensive or conversely too basic:

"I needed a therapy that could better address what I felt. It didn't give me a specific answer to my worries". [26, no gender details, 21-59 yrs, people with a range of serious mental health problems]

"I would have liked to have more of a personal contact, it became a little distant everything, to do on the internet, because it is so heavy stuff, it's nice to meet a real person when you're working with heavy things like this" [24, no gender or age details, generalised anxiety disorder]

"I wanted something more about me specifically, as opposed to talking about general issues" [21, Male, 40-49 yrs, Bipolar disorder, BEP group]

"The information in the modules was too general and too limited" [21, Male, 18-29 yrs, Bipolar disorder, BEP group]

Some other participants simply indicated that they had been unhappy or dissatisfied or "not comfortable" with the treatment they had received although specific reasons were not provided within the included studies [22,23,31].

3) The compatibility of aspects of trial processes with individual capabilities

Across 3 of the 11 studies [23,24,31] the extent to which aspects of the interventions were deemed to be appropriately 'pitched' at the individual emerged as being of importance. For example, participants cited attention problems and limited reading and writing skills as a reason for withdrawing from internet delivered cognitive behavioural therapy [24] or as a reason for non-response to follow-up questionnaires [31]. Communication and cultural issues were also cited as reasons for the discontinuation of problem solving treatments [23], suggesting that these issues would benefit from greater consideration and discussion at the consenting stage:

"I thought that it was too much to read, and I cannot read anything at all that I need to remember or learn. It goes in here and out there [pointing at the ears]" [24, no gender or age details, generalised anxiety disorder]

4) Concerns about or experiences of the trial medication

For peer review only - http://bmjopen.bmj.com/site/about/guidelines.xhtml

Across 2 of the 8 studies which were set in trials testing drug interventions, [27 – 2 trials; 29] concerns about the study medication were cited as reasons for discontinuing with trial participation. These included concerns that the trial drug(s) were not properly tested/licensed [27], concerns that the trial medication could negatively interact with other prescribed medication [29], through to citing a dislike of taking too much medication [29] or that the trial medication tasted offensive [27]. Constructs within this key theme again suggest potential issues with the informed consent process and highlight the importance of discussions about the purpose of any trial, the nature of trial medications and also the implication for participation of having certain co-morbidities, linking back in to the complex inter-relationship between self and trial process/procedures:

"It just scared me when it said not to be given to children under 20…I didn't understand they weren't licensed for children…and that's what I thought it was, just to see if it worked, not to actually like so then it could be licensed" [27; Mother of child in trial for young people aged 4-18yrs with rheumatic diseases]

"again I found that I had stomach problems with the tablet so I assumed that it must be the aspirin…[29, Female, 63 yrs, stopped taking trial medication for asymptomatic atherosclerosis]

"..and they discovered I had heart fibrillation…After that I'd to go on warfarin you see, so that's why I had to drop out because warfarin and aspirin just don't agree" [29, Female, 77 yrs, stopped taking trial medication for asymptomatic atherosclerosis]

"I didn't think I really wanted to go on at the start but mum and dad persuaded me to. And so…when I was getting really fed up I just said 'No I don't want to' because I didn't like the taste [of the medicine] [27; POP trial; young person 11-14 yrs]

5) Considerations around the extent to which trial participation could be appropriately accommodated into their broader lives

Aside from issues relating to beliefs about current health state, individual capabilities, preferences for care and concerns about side effects, participants also discussed how decisions to discontinue with trial participation related to other life 'events' that tended to take priority over or made it hard for them to engage fully with the various demands of the trial [21, 22,23,24,25,26,30,31]. These factors appeared less directly related to the nature of the trial interventions themselves and more about the challenges of life in general (with one study [31] suggesting that these people could be classed more as 'happy' rather than 'unhappy' non-responders, in the sense that non-retention may be related to aspects out-with the trial itself). Reasoning here involved trading off trial participation with competing priorities and ranged from events such as work or family commitments, moving to another country, exams, pregnancies, postal strikes etc and more generally simply daily routines that got in the way. Within this theme participants also sometimes cited 'laziness' or 'forgetfulness' as reasons for why they had either not completed trial interventions or had not responded to follow-up questionnaires with some apparently being unaware that they were being considered as 'drop-outs' be study researchers:

For peer review only - http://bmjopen.bmj.com/site/about/guidelines.xhtml

[discussing cessation of therapy sessions/non-completion of study workbooks]*"I didn't have the time, and with everything else, it wasn't a priority"* [21, Female, 18-29 yrs, Bipolar disorder, control group]

[describing why they did not return a follow-up questionnaire] *"Do you know what…laziness I'm just gonna put it down to that"*

Researcher: *"OK and em it wasn't because you were disgruntled about part of the project?"*

"Definitely not no" [31, no gender/age details given, severe ankle sprains)

[discussing cessation of problem solving treatment sessions]"Did I drop out? No, I didn't dropout. I became busy and I figured I started missing calls." [23, Female, no age details, Cancer and depression)

Discussion

Principal findings

Our meta-ethnographic synthesis sought to explore factors that influence non-retention within clinical trial contexts. We identified 11 qualitative studies reporting data from 13 trials that explored participant reported reasons for not completing any or some of the various trial processes (after initially consenting to take part). What emerged from our analysis was the importance of trial participants' perceptions about the personal compatibility of key aspects of the trial with their personal beliefs, preferences, capabilities or life circumstances. These factors related to their own health state, preferences for how they wanted to receive care, their individual capabilities, beliefs about or experiences of trial medication, and also considerations around the extent to which trial participation could be appropriately accommodated or not into their broader lives (Conceptually illustrated in S3 Figure). Our synthesis has also highlighted that people's reasoning around dropping out of a trial can be described as being more or less 'active' in nature, with some people in our synthesis not even realising that they were being considered by the researchers as trial 'drop outs' [23]. All these factors raise important issues around the extent to which initial decisions to participate were fully informed and illustrate the importance for trial recruiters of ensuring that prospective participants are made aware of what the trial will entail and also what will be expected of them in terms of full participation.

Quantitative surveys have tended to investigate non-retention in the context of non-response to follow up questionnaires. These studies have identified either participant characteristics or trial processes as being potential predictors of trial retention [9,10,11].Whilst these studies have a place, it is arguably difficult to influence some of these previously identified factors influencing retention as they may not be modifiable e.g. age or study duration. Our synthesis of more in depth qualitative studies has usefully built on these findings and has enabled a more nuanced understanding of key issues of relevance (which are potentially modifiable) relating to non-adherence to interventions and non-return of follow-up questionnaires. Participant characteristics as well as trial processes are of importance but we have also demonstrated that there can be a complex inter-relationship between the two. For example, a perception that the nature of the intervention negatively affects one's

For peer review only - http://bmjopen.bmj.com/site/about/guidelines.xhtml

mental health can be of importance as can perceptions about the nature of the intervention in relation to perceptions of self or in relation to personal preferences for care and support. Furthermore, the compatibility or otherwise of various trial processes with individual capabilities can have implications for retention. Reasons given for not completing various trial processes were not necessarily mutually exclusive, but were rather a synergistic combination of factors that could apparently work towards trial non-retention. Our findings also highlight that some participants' behaviour around leaving a trial could be described as being more or less 'active' in nature (e.g. stopping trial medication because of a concern around side effects (active) versus simply not remembering or being too busy to return a questionnaire (passive)). This is an important finding and one that has not been given due consideration in previous literature to date. People's views and life situations can change over time, all having the potential to impact on their retention within a trial. Furthermore, different types of trials are likely to present particular challenges in terms of their potential for non-retention. It could also be that certain types of reasoning might be more or less modifiable and easier to address particularly if they can be anticipated upfront during the trial design stage.

A recent study exploring reasons why people declined trial participation at the consent to recruitment stage has found that most declined at the outset because they judged themselves ineligible or not in need of the specific trial therapy in question [34]. The study authors suggest that to improve recruitment to trials the most successful interventions are likely to be the ones that focus on patients' assessments of their own eligibility and their potential to benefit from the trial treatment, rather than reducing trial burden per se. In our synthesis we found that perceptions around eligibility and assessments regarding potential to benefit from the trial treatment were also considerations for people who had initially decided to join but who had subsequently ceased to engage. For example, this included those who felt that they had recovered such they did not need to engage further [21,22,23,25] and those who felt they could manage sufficiently well without engaging with the intervention [21,28,29]. However, in the context of non-retention, it is worth considering issues around trial burden (e.g. interventions that might be perceived to be too technical or too demanding given a person's health state) as well as issues around preference for particular styles of care and support and acknowledging that the specific intervention and, or, the ways outcomes are assessed has to be compatible within the context of trial participants' broader lives. In other words, issues around reducing trial burden is of importance, both in terms of the intervention itself and also the ways that follow up data is collected.

We know from previous syntheses of qualitative studies focusing on trial recruitment that people often choose to enter into trials in the hope of gaining some help for themselves from the intervention (even if they also state they are doing so for altruistic reasons – i.e. to benefit research more generally), so called 'conditional altruism' [7]. Some participants in our synthesis described perceptions around feeling too ill to continue taking part or feeling suitably better such that trial engagement was no longer warranted [21,22,23,24,25]. This perception of improvement in health would appear to resonate with the concept of conditional altruism in the sense that people might cease participation if they perceive their condition improves or conversely deteriorates, such that in effect their benefit for self has been realised and their continued participation is no longer warranted. Our finding here is perhaps exaggerated in trials with a mental health context (which applied to 6 of the 13 included trials), where diagnoses can adversely affect people's ability and inclination to initially take part in research [34,35,36]. We have shown that this issue also has

For peer review only - http://bmjopen.bmj.com/site/about/guidelines.xhtml

relevance for retention in such trials as people's health states can be particularly vulnerable to fluctuation [34]. A recent meta-synthesis of factors affecting recruitment to depression trials [37] indicated that decisions can depend on issues relating to: perceptions of health at the time of invite; attitudes towards the research and trial interventions; and the demands of the trial. Our synthesis has shown that some of this reasoning might also have the potential to impact on non-retention in those who are successfully recruited. Furthermore, previous research has suggested that the therapeutic alliance can have an impact on adherence to treatment [38]. Within the papers included in our synthesis, this was not something that was discussed per se. However, as one of our key themes illustrate, some decisions to discontinue trial participation appeared to relate to the fit of aspects of the intervention with preferences for how participants wanted to receive care and support. Within this, some trial non-retainers stated that they had wanted more face-to-face personal contact with for example, a therapist. This comparable finding could suggest that the underlying beliefs, preferences and expectations about trial participation are not explored and unpacked fully during trial consenting discussions.

Strengths and limitations

Undertaking a meta-ethnographic synthesis of findings from qualitative studies that explored factors influencing non-retention within clinical trials has allowed us to gain important new shared insights into factors that seem to affect retention across a range of trial contexts - to our knowledge this is the first study to have synthesised these primary studies in this way. However, our systematic search identified only 11 eligible papers reporting findings across 13 trials, 5 of which had a mental health context and all of which were conducted in high-income countries. Furthermore, unlike for example surgical trials, all the included papers incorporated within their trials, interventions that participants could choose to discontinue engaging with (e.g. taking drugs; stopping CBT etc). Whilst qualitative research does not usually intend to be generalizable, it is nevertheless important to consider the transferability of our findings to other clinical trial contexts and settings and one could argue that participants within e.g. mental health trials, surgical trials, or trials that involve surrogate/proxy consent including those involving children [27] might face very different issues and challenges regarding retention. Although we were reassured that the key themes we identified had resonance across the included papers to a greater or lesser extent and so are likely to be important considerations within a range of clinical trial contexts, some influences on trial non-retention are likely to be more trial specific than others (e.g. concerns about trial medication).

We carried out a quality assessment of the 11 included papers (see S1 CASP Checklist). Although all papers had study aims that were amenable to investigation via qualitative means and all included qualitative data, some were deemed richer than others in terms of data and insights (i.e. first and second order constructs). For example, 1 paper only reported qualitative data from open ended questionnaire response options [22], and 2 were deemed less useful in terms of presenting only very limited qualitative data (both first and second order constructs) (22,23)). Nevertheless, we did feel that they provided some helpful insights that usefully built on the findings of the other papers. Furthermore, despite some variation in the overall level of quality, due to the small number of included studies we felt it was more important to retain any relevant findings rather than disregard based on study quality. In doing so, we would argue that all eight papers contributed useful elements to the collective whole and enabled us to develop our line of argument in terms of the issues of importance regarding trial non-retention.

For peer review only - http://bmjopen.bmj.com/site/about/guidelines.xhtml

Practice Implications

To improve retention in clinical trials, researchers should work to reduce the burden on trial participants both through the design of the intervention itself as well as through simplified data collection processes. Providing more detail on the nature of the trial interventions and what can be expected by 'participation' at the consenting stage may prove helpful in order to manage expectations.

Some people in our synthesis appeared to be unaware that they were being considered as trial non-retainers by the study researchers. This raises the question of participants' understanding of the importance of remaining in a trial for its duration (i.e. completing the intervention and the outcome assessments) and its implications for the study in question.

A recent study of patient information documentation from UK NIHR funded trials has highlighted that withdrawal and retention are poorly described and that statements about the value of retention are infrequent [39]. The authors of this study also noted that there was an over-emphasis on a prospective participant's right to withdraw without corresponding information about the importance for research of sustained participation [39]. If trialists want to improve retention to clinical trials as well as recruitment then there is an argument for giving the latter more prominence in patient information materials (and also during any trial recruitment discussions). Trial decision aids have shown some promise here in terms of having the potential to support more informed decisions around trial participation including around issues of understanding, and also the extent of involvement and commitment to a trial over time [40].

Our synthesis also potentially highlights the issue of people's awareness or lack thereof of what the trial interventions would entail. For example, some participants were apparently not fully aware of what the intervention would entail before consenting and described after they had initially agreed to take part that it was incompatible either with their sense of self (i.e. a perception that it would not help them or was not needed) or that it did not suit with their individual capabilities (i.e. being computer based and being perceived as too technical) [21,23,24,26,27,28]. If trialists want to improve retention then this suggests an argument for also providing more detail on the nature of the trial interventions at the consenting stage in order to manage expectations. We know from previous literature that patient/public involvement at the front end of trial design tends to be extremely limited if indeed it happens at all [41,42]. Given some of the key factors we found as being influential for non-retention, one could speculate that some early and meaningful patient/public involvement would be particularly useful (e.g. for ensuring that aspects of the trial are user-friendly and as compatible as possible with the target population's likely preferences and capabilities).

Implications for Research

A Cochrane review investigating interventions to improve retention in trials has highlighted that most strategies to improve retention have focussed on trying to improve follow-up questionnaire response [43]. Of these interventions, only monetary incentives have been shown to have a significant effect on return of questionnaires and the review highlighted that very few studies

For peer review only - http://bmjopen.bmj.com/site/about/guidelines.xhtml

included trial participants in their design or development [43]. Our synthesis has demonstrated that there may be a range of issues relevant to trial participants that influence non-retention which may not be amenable to modification by 'incentives' or other interventions that fail to consider participants during development.

As mentioned previously, qualitative methods to improve recruitment to trials is now recognised as a well-established methodology built into the design and delivery of large publically funded clinical trials. The Qunitet Recruitment Intervention (QRI) is gathering momentum across a range of trials and Clinical Trials Units as a mechanism to unpack many of the nuances around how participants are recruited to RCTs [44]. Many of the approaches in the QRI are directly transferable to questions about retention. For example, how it is discussed in consultations and trial paperwork, what do stakeholders (trial participants and trial staff) report as the barriers and facilitators to retention, and work in this area could prove fruitful for minimising non-retention in ongoing RCTs. However, despite there being a clear need for more research in the context of trial retention, we also recognise the inherent challenges for researchers in obtaining the necessary ethical approvals for this type of research (particularly as current recruitment materials for trial participants tend to emphasise prospective participants' right to withdraw without given any reasons etc). Therefore, development of shareable resources to facilitate regulatory approvals may be an important contribution for the trials methodology community.

Finally, given that 5 of the 11 included studies focused on qualitative research within mental health trials there is certainly scope for further primary studies exploring the barriers and enablers to trial retention from a participant's perspective. Ideally, these studies would consider and explore all aspects of trial process relevant for retention, including how aspects of the trial are discussed within recruitment consultations. Interestingly, our search did not identify any studies that had explored reasons for trial participants' non-attendance at trial follow-up visits. This mirrors the findings of the Cochrane review on interventions to improve retention to trials which also found very little evidence with regard to interventions to improve attendance at follow-up visits [43].

Conclusions

Our systematic literature search and synthesis has highlighted that there is very little published qualitative literature exploring participant reported reasons for non-retention in clinical trials. Researchers have already called for 'a science of recruitment' in recognition that recruiting for science (e.g. trials) is not currently underpinned by an evidence base around the factors which might have the potential to impact on recruitment [1]. This is undoubtedly important but we would also argue that we need to develop a parallel focus on 'a science of retention' if we are to start to be able to tackle the very real issue of non-retention in clinical trials. Our qualitative synthesis (of albeit a small set of studies) feeds into this relatively undeveloped science and has shed some important light on the factors that might influence non-retention in clinical trials- factors that have implications both for practice and for further research. Taken together, the findings presented here and the subsequent implications for practice and research highlight the critical need to plan for retention as much as for recruitment during trial design and not treat it like the overlooked trial conduct 'Cinderella'.

For peer review only - http://bmjopen.bmj.com/site/about/guidelines.xhtml

Supporting Information

S1 ENTREQ Checklist

S1 Appendix. Database search strategies

S1 Box Steps of Meta-ethnography

S1 Figure. PRISMA flow diagram

S2 Figure. PRISMA flow diagram – updated search

S1 Table. Characteristics of included studies

S2 Table. Key themes of influence on decisions to withdraw from trial participation

S3 Table. Spread of themes across included studies

S3 Figure. Conceptual diagram

S1 CASP Checklist

Acknowledgements

We would like to thank Cynthia Fraser for her assistance in helping develop the database searching strategies.

Contributors

KG conceived the study idea. RN screened all titles and abstracts in initial search; ZS screened all titles and abstracts in updated search with KG screening a random 10% sample from both searches. ZCS, RN and KG conducted the data analysis and ZCS wrote the initial and subsequent manuscript drafts. All the authors contributed critically to discussions about interpretation of data and revisions of manuscript drafts. All the authors approved the final version.

Funding

ZCS was supported by a core grant from the CSO (reference CZU/3/3) and a Wellcome Trust Institutional Strategic Support Fund award (reference RG12724-18). RN was supported by a Wellcome Trust Institutional Strategic Support Fund award (reference RG12724-18). KG was supported by an MRC Methodology Research Fellowship (MR/L01193X/1).

Data sharing statement

This is a review of published studies which are available to access through the relevant journals.

Competing interests statement

There are no competing interests for any author.

For peer review only

References

1. Bower P, Wallace P, Ward E, Graffy J, Miller J, Delaney B, Kinmonth AL. Improving recruitment to health research in primary care. Fam Pract. 2009;26(5):391–7.
2. Adamson J, Hewitt CE, Torgerson DJ. Producing better evidence on how to improve randomised controlled trials. BMJ. 2015;351:h4923.
3. Blanch DC, Rudd RE, Wright E, et al. Predictors of refusal during a multi-step recruitment process for a randomized controlled trial of arthritis education. Patient Educ Couns 2008;73:280–5.
4. Madsen SM, Holm S, Davidsen B, et al. Ethical aspects of clinical trials: the attitudes of participants in two non-cancer trials. J Intern Med. 2000;248:463–74.
5. Verheggen FW, Nieman F, Jonkers R. Determinants of patient participation in clinical studies requiring informed consent: why patients enter a clinical trial. Patient Educ Couns 1998;35:111–25.
6. Locock L, Smith L. Personal benefit, or benefiting others? Deciding whether to take part in clinical trials. Clin Trials 2011;8:85–93.
7. McCann SK, Campbell MK, Entwistle VA. Reasons for participating in randomised controlled trials: conditional altruism and considerations for self. Trials 2010;11:31.
8. Smith CT, Hickey H, Clarke M, Blazeby J, Williamson P. The trials methodological research agenda: results from a priority setting exercise. Trials. 2014;15(1):32.
9. Applebaum AJ, Lichtenthal WG, Pessin HA, et al. Factors associated with attrition from a randomized controlled trial of meaning-centered group psychotherapy for patients with advanced cancer. Psycho-Oncology. 2012; Nov;21(11):1195-204. doi: 10.1002/pon.2013. Epub 2011 Jul 12.
10. McCambridge J, Kalaitzaki E, White IR, Khadjesari Z, Murray E, Linke S et al. Impact of length or relevance of questionnaires on attrition in online trials: randomized controlled trial. Journal of Medical Internet Research. 2011 Oct;13(4):e96. e96. Available from, DOI: 10.2196/jmir.1733.
11. Hui D, Glitza I, Chisholm G et al. Attrition rates, reasons, and predictive factors in supportive care and palliative oncology clinical trials. Cancer. 2013:119;5:1098-1105.
12. O'Cathain A, Thomas K, Drabble Set al. The QUAlitative Research in Trials (QUART) study. Health Technology Assessment. 2014;18: No. 38 DOI: 10.3310/hta18380.
13. Noblit GW, Hare RD. Meta-ethnography: synthesizing qualitative studies. 1988. Newbury Park: Sage.
14. Britten N, Campbell R, Pope C et al. Using meta ethnography to synthesise qualitative research: a worked example. J Health Serv Res Policy. 2002;7:4:209–215.
15. Critical Appraisal Skills Programme (2017). CASP (Qualitative Research) Checklist. [online] Available at: http://docs.wixstatic.com/ugd/dded87_25658615020e427da194a325e7773d42.pdf Accessed: July 2017.
16. Campbell R, Pound P, Pope C et al. Evaluating meta-ethnography: a synthesis of qualitative research on lay experiences of diabetes and diabetes care. Social Science and Medicine. 2003;56:671-684.

For peer review only - http://bmjopen.bmj.com/site/about/guidelines.xhtml

17. Pound P, Britten N, Morgan M, et al. Resisting medicines: a synthesis of qualitative studies of medicine taking. Soc Sci Med. 2005. 61: 133-155. 10.1016/j.socscimed.2004.11.063.
18. Atkins S, Lewin S, Smith H, et al. Conducting a meta-ethnography of qualitative literature: Lessons learnt. BMC Medical Research Methodology. 2008, 8:21 doi:10.1186/1471-2288-8-21.
19. Schumm K, Skea Z, McKee L, N'Dow J. 'They're doing surgery on two people': a meta-ethnography of the influences on couples' treatment decision making for prostate cancer. Health Expectations. 2010:13, pp.335–349.
20. Black M, Entwistle VA, Bhattacharya S, et al. Vaginal birth after caesarean section: why is uptake so low? Insights from a meta-ethnographic synthesis of women's accounts of their birth choices. BMJ Open 2016;6: e008881. doi:10.1136/ bmjopen-2015-008881.
21. Nicholas J, Proudfoot J, Parker G, et al. "The Ins and Outs of an Online Bipolar Education Program: A Study of Program Attrition."J Med Internet Res 2010 (Dec 19); 12(5):e57.
22. Postel M, de Haan H, ter Huurne E, et al. Effectiveness of a Web-based Intervention for Problem Drinkers and Reasons for Dropout: Randomized Controlled Trial. J Med Internet Res 2010;12(4):e68.
23. Wells A, Palinkas L, Qiu X, et al. Cancer patients' perspectives on discontinuing depression treatment: the "drop out" phenomenon. Patient preference and Adherence. 2011:5;465-470.
24. Johansson O, Michel, T, Andersson G, et al. Experiences of non-adherence to Internet-delivered cognitive behavior therapy: A qualitative study. Internet Interventions. 2015:2;137-142.
25. Sari S, Muller AE, Roessler KK. Exercising alcohol patients don't lack motivation but struggle with structures, emotions and social context – a qualitative dropout study. BMC Family Practice 2017:18;45.
26. Fernandez-Alvarez J, Diaz-Garcia A, Gonzalez-Robles A et al. Dropping out of a transdiagnostic online intervention: A qualitative analysis of clients' experiences. Internet Interventions. 2017:10; 29-38.
27. Shilling, V, Williamson, P R, Hickey, H, Sowden, E, Smyth, R L and Young, B (2011) Processes in recruitment to randomised controlled trials of medicines for children (RECRUIT): a qualitative study. Project Report. 2011: 15. NIHR Journals Library.
28. Sanders C, Rogers A, Bowen R, et al. Exploring barriers to participation and adoption of telehealth and telecare within the Whole System Demonstrator trial: a qualitative study. BMC Health Services Research. 2012: **12**:220 https://doi.org/10.1186/1472-6963-12-220.
29. Eborall H, Stewart M, Cunningham-Burley S, et al. et al. Accrual and drop out in a primary prevention randomised controlled trial: qualitative study.Trials. 2011: 12:7 http://www.trialsjournal.com/content/12/1/7.
30. Henshall C, Narendran P, Andrews RC et al. Qualitative study of barriers to clinical trial retention in adults with type 1 diabetes. BMJ Open. 2018;8:e022353.
31. Nakash R, Hutton J, Lamb S, et al. Response and non-response to postal questionnaire follow-up in a clinical trial – a qualitative study of the patient's perspective. Journal of Evaluation in Clinical Practice. 2008:14;226-235.
32. Lang H, France E, Williams B et al. The psychological experience of living with head and neck cancer: a systematic review and synthesis. Psychooncology. 2013:22;12:2648-63.
33. France E, Wells M, Lang H et al. Why, When and how to update a meta-ethnography qualitative synthesis. Systematic Reviews. 2016:5:44.
34. Hughes-Morley A, Young B, Hempel R, Russell I, Waheed W, Bower P. What can we learn from trial decliners about improving recruitment? Qualitative study *Trials.*2016;**17**:494.

For peer review only - http://bmjopen.bmj.com/site/about/guidelines.xhtml

35. Halpern SD, Karlawish JHT, Berlin JA. The continuing unethical conduct of underpowered clinical trials. JAMA. 2002;288:358–62.
36. Drueke TB, Descamps-Latscha B, Locatelli F. Stopping a medical research project for financial reasons. Nephrol Dial Transplant. 2003;18(10):1982–3.
37. Hughes-Morley A, Young B, Waheed W, Small N, Bower P. Factors affecting recruitment into depression trials: systematic review, meta-synthesis and conceptual framework. J Affect Disord. 2015;172C:274–90.
38. Thompson L, McCabe R. The effect of clinician-patient alliance and communication on treatment adherence in mental health care: a systematic review. BMC Psychiatry. 2012;12:87. doi:10.1186/1471-244X-12-87.
39. Kearney A, Rosala-Hallas A, Bacon N, et al. Reducing attrition: the communication of retention and withdrawal within patient information sheets. Trials. 2017:18;(Suppl 1):P.489.
40. Gillies K, Skea ZC, Campbell MK. Decision aids for randomised controlled trials: a qualitative exploration of stakeholders' views. BMJ Open 2014;4:e005734. doi:10.1136/bmjopen-2014-005734.
41. Gamble C, Dudley L, Allam A, et al. Patient and public involvement in the early stages of clinical trial development: a systematic cohort investigation. BMJ Open 2014;4:e005234. doi:10.1136/bmjopen-2014- 005234.
42. Buck D, Gamble C, Dudley L, et al. From plans to actions in patient and public involvement: qualitative study of documented plans and the accounts of researchers and patients sampled from a cohort of clinical trials. BMJ Open 2014;4:e006400. doi:10.1136/bmjopen-2014- 006400.
43. Brueton VC, Tierney J, Stenning S, Harding S, Meredith S, Nazareth I, Rait G. Strategies to improve retention in randomised trials. Cochrane Database of Systematic Reviews 2013, Issue 12. Art. No.: MR000032. DOI: 10.1002/14651858.MR000032.pub2.
44. Donovan J, Rooshenas L, Jepson M et al. Optimising recruitment and informed consent in randomised controlled trials: the development and implementation of the Quintet Recruitment Intervention (QRI). Trials. 2016; 17:283. DOI 10.1186/s13063-016-1391-4.

For peer review only

For peer review only - http://bmjopen.bmj.com/site/about/guidelines.xhtml

Search strategies

July 2018 Update

Database: Embase <1996 to 2018 Week 30>, Ovid MEDLINE(R) and Epub Ahead of Print, In-Process & Other Non-Indexed Citations, and Daily <1946 to July 23, 2018>

Search run 24th July 2018

URL: https://ovidsp.ovid.com/

1 exp clinical trial/ (1980088)

2 randomi?ed control$ trial$.tw. (336149)

3 randomi?ed controlled trial?.kw. (23247)

4 clinical trial?.tw,kw. (729051)

5 controlled trial?.tw,kw. (429924)

6 controlled clinical trial?.tw,kw. (50908)

7 pragmatic trial?.tw,kw. (2136)

8 complex intervention?.tw,kw. (4998)

9 or/1-8 (2639576)

10 qualitative research/ (94827)

11 qualitative research.tw,kw. (34500)

12 (qualitative adj3 method$).tw. (53149)

13 (qualitative method? or qualitative methodology).kw. (2740)

14 (qualitative adj3 stud$).tw. (95856)

15 qualitative study.kw. (2597)

16 focus groups/ use ppez (24850)

17 focus group?.tw,kw. (82443)

18 grounded theory/ (6039)

19 grounded theory.tw,kw. (21174)

20 narrative analys?s.tw,kw. (2147)

21 process evaluation.tw,kw. (5815)

22 mixed method?.tw,kw. (30409)

23 mixed method$.mp. (31273)

24 mixed methodology.tw,kw. (711)

25 (in depth adj4 interview$).tw. (41493)

26 in depth interview?.kw. (201)

27 ((semi structured or semistructured) adj5 interview$).tw. (89673)

28 semi structured interview?.kw. (277)

For peer review only

29	qualitative interview$.tw. (18002)
30	qualitative interview?.kw. (432)
31	(interview$ and theme$).tw. (61425)
32	interview?.kw. (6730)
33	(interview$ and audio recorded).tw. (5252)
34	qualitative case stud$.tw. (2011)
35	descriptive case stud$.tw. (475)
36	qualitative case study.kw. (25)
37	descriptive case study.kw. (0)
38	qualitative exploration.tw,kw. (1994)
39	qualitative evaluation.tw,kw. (6296)
40	qualitative intervention.tw,kw. (25)
41	qualitative approach.tw,kw. (7887)
42	qualitative inquiry.tw,kw. (1197)
43	qualitativ$ analys$.tw. (31755)
44	qualitative analysis.kw. (1269)
45	(qualitative adj3 data).tw. (34567)
46	qualitative data.kw. (152)
47	discourse analysis.tw,kw. (3342)
48	discursive.tw,kw. (3245)
49	phenomenological.tw,kw. (29346)
50	thematic analysis.tw,kw. (27166)
51	ethnograph$.tw. (18207)
52	ethnography.kw. (1849)
53	action research.tw,kw. (7525)
54	ethno?methodology.tw,kw. (150)
55	social construction.tw,kw. (1643)
56	or/10-55 (426531)
57	phenomenological characteristics.tw,kw. (242)
58	phenomenological model.tw,kw. (1806)
59	action research arm test.tw,kw. (1065)
60	protocol.ti. (79269)
61	or/57-60 (82341)
62	56 not 61 (418963)
63	9 and 62 (25059)
64	Patient Dropout/ use ppez (7679)
65	Patient Dropouts/ use emef (433)

For peer review only - http://bmjopen.bmj.com/site/about/guidelines.xhtml

66 Patient Recruitment/ use ppez (59391)

67 Research Subjects/ use emef (4918)

68 patient recruitment.kw. (179)

69 attrition.kw. (1451)

70 patient retention.kw. (32)

71 ((recruit$ or participat$ or take part or dropout$ or drop$ out$ or withdr?wl$ or barrier$ or retention or response$ or respond$ or attrition) adj10 trial?).tw. (134886)

72 or/64-71 (204550)

73 63 and 72 (3670)

74 limit 73 to english language (3640)

75 74 not abstract.pt. (3020)

76 exp animals/ not human/ (7165523)

77 exp nonhuman/ not humans/ (3518640)

78 75 not (76 or 77) (3011)

79 limit 78 to yr="2010 -Current" (2280)

80 remove duplicates from 79 (1325)

****

Database: PsycINFO <2002 to July Week 3 2018>

Search run 24th July 2018

URL: https://ovidsp.ovid.com/

1 clinical trials/ (10606)

2 randomi?ed control$ trial$.tw. (26600)

3 clinical trial?.tw. (27527)

4 controlled trial?.tw. (31463)

5 controlled clinical trial?.tw. (2319)

6 pragmatic trial?.tw. (191)

7 complex intervention?.tw. (624)

8 or/1-7 (58183)

9 qualitative research/ (7513)

10 qualitative research.tw. (17348)

11 (qualitative adj3 method$).tw. (21172)

12 (qualitative adj3 stud$).tw. (46815)

13 focus group?.tw. (27999)

14 grounded theory/ (3229)

15 grounded theory.tw. (12698)

For peer review only - http://bmjopen.bmj.com/site/about/guidelines.xhtml

16 narrative analys?s.tw. (1966)
17 process evaluation.tw. (1167)
18 mixed method?.tw. (18416)
19 mixed methodology.tw. (731)
20 (in depth adj4 interview$).tw. (21003)
21 ((semi structured or semistructured) adj5 interview$).tw. (34163)
22 qualitative interview$.tw. (7708)
23 (interview$ and theme$).tw. (33368)
24 interview?.kw. (0)
25 (interview$ and audio recorded).tw. (1253)
26 qualitative case stud$.tw. (4333)
27 descriptive case stud$.tw. (565)
28 qualitative exploration.tw. (944)
29 qualitative evaluation.tw. (751)
30 qualitative intervention.tw. (9)
31 qualitative approach.tw. (3312)
32 qualitative inquiry.tw. (1457)
33 qualitativ$ analys$.tw. (10100)
34 (qualitative adj3 data).tw. (17288)
35 discourse analysis/ (6373)
36 discursive.tw,kw. (7705)
37 phenomenological.tw. (20981)
38 thematic analysis.tw. (9031)
39 ethnograph$.tw. (21222)
40 action research.tw. (6521)
41 ethno?methodology.tw. (369)
42 social construction.tw. (2816)
43 or/9-42 (211086)
44 phenomenological characteristics.tw. (129)
45 phenomenological model.tw. (123)
46 action research arm test.tw. (110)
47 protocol.ti. (2319)
48 or/44-47 (2678)
49 43 not 48 (210507)
50 experimental attrition/ (307)
51 experimental recruitment/ (96)
52 experimental subjects/ (2407)

For peer review only

53 dropouts/ (348)

54 ((recruit$ or participat$ or take part or dropout$ or drop$ out$ or withdr?wl$ or barrier$ or retention or response$ or respond$ or attrition) adj10 trial?).tw. (13610)

55 or/50-54 (16312)

56 8 and 49 and 55 (484)

57 exp animals/ not human/ (179236)

58 56 not 57 (484)

59 limit 58 to (english language and yr="2010 -Current") (339)

****

Cochrane Central Register of Controlled Trials : Issue 7, 2018

Search run 25th July 2018

URL: http://www.cochranelibrary.com/

#1 MeSH descriptor: [Qualitative Research] this term only

#2 qualitative NEXT research:ti,ab,kw or qualitative NEXT method:ti,ab,kw or qualitative NEXT study:ti,ab,kw (Word variations have been searched)

#3 MeSH descriptor: [Focus Groups] this term only

#4 MeSH descriptor: [Grounded Theory] this term only

#5 mixed NEXT method:ti,ab,kw or narrative NEXT analysis:ti,ab,kw (Word variations have been searched)

#6 interview:ti,ab,kw

#7 qualitative case study:ti,ab,kw or descriptive case study:ti,ab,kw (Word variations have been searched)

#8 qualitative NEXT exploration:ti,ab,kw or qualitative NEXT evaluation:ti,ab,kw or qualitative intervention:ti,ab,kw or qualitative approach:ti,ab,kw or qualitative analysis: ti,ab,kw (Word variations have been searched)

#9 qualitative data:ti,ab,kw (Word variations have been searched)

#10 discourse analysis:ti,ab,kw or discursive:ti,ab,kw (Word variations have been searched)

#11 social construction:ti,ab,kw or action research:ti,ab,kw or ethnography:ti,ab,kw or thematic analysis:ti,ab,kw or phenomenological:ti,ab,kw (Word variations have been searched)

#12 #1 or #2 or #3 or #4 or #5 or #6 or #7 or #8 or #9 or #10 or #11

#13 MeSH descriptor: [Patient Dropouts] this term only

#14 MeSH descriptor: [Patient Selection] this term only

#15 ((recruit$ or participat$ or take part or dropout$ or drop$ out$ or withdr?wl$ or barrier$ or retention or response$ or respond$ or attrition) near/10 trial?):ti,ab,kw (Word variations have been searched)

For peer review only

#16 #13 or #14 or #15

#17 #12 and #16 Publication Year from 2010 to 2018, in Trials

#18 abstract:pt (Word variations have been searched)

#19 #17 not #18 (385)

Social Sciences Citation Index

Search run: 24th July 2018

Web of Knowledge: URL http://wok.mimas.ac.uk/

For peer review only

#32 382 #21 AND #24 AND #31 Refined by: DOCUMENT TYPES: (ARTICLE OR REVIEW) Indexes=SSCI Timespan=2010-2018

31 12,293 #25 OR #26 OR #27 OR #28 OR #29 OR #30

30 1,646 (TS=patient dropout) AND LANGUAGE: (English)

29 1,311 (TS=patient attrition) AND LANGUAGE: (English)

28 2,040 (TS=patient retention) AND LANGUAGE: (English)

27 4761 (TS=((withdraw$ or barrier$ or retention or response$ or respond$ or attrition) NEAR/10 trial?)) AND LANGUAGE: (English)

26 136 (TS=(("take part" or dropout$ or "drop$ out") NEAR/10 trial?)) AND LANGUAGE: (English)

25 220 (TS=((recruit$ or participat$) NEAR/10 trial?)) AND LANGUAGE: (English)

24 42,063 #23 OR #22

23 42,671 (TS=randomised controlled trial) AND LANGUAGE: (English)

22 42,671 (TS=randomized controlled trial) AND LANGUAGE: (English)

21 148,316 #20 OR #19 OR #18 OR #17 OR #16 OR #15 OR #14 OR #13 OR #12 OR #11 OR #10 OR #9 OR #8 OR #7 OR #6 OR #5 OR #4 OR #3 OR #2 OR #1

20 7,765 (TS=thematic analysis) AND LANGUAGE: (English)

19 16,672 (TS=action research) AND LANGUAGE: (English)

18 7,436 (TS=social construction) AND LANGUAGE: (English)

17 5,371 (TS=discursive) AND LANGUAGE: (English)

1611,763 (TS=discourse analysis) AND LANGUAGE: (English)

15 6,704 (TS=(qualitative near/1 data)) AND LANGUAGE: (English)

14 8,581 (TS=(qualitative near/1 analysis)) AND LANGUAGE: (English)

13 3,122 (TS=(qualitative near/1 approach)) AND LANGUAGE: (English)

12 122 (TS=(qualitative near/1 intervention)) AND LANGUAGE: (English)

11 688 (TS=(qualitative near/1 evaluation)) AND LANGUAGE: (English)

10 529 (TS=(qualitative near/1 exploration)) AND LANGUAGE: (English)

For peer review only - http://bmjopen.bmj.com/site/about/guidelines.xhtml

9 40,423 (TS=(in depth interview* or semi structured interview* or qualitative interview*)) AND LANGUAGE: (English)

8 7,414 (TS=narrative analysis) AND LANGUAGE: (English)

7 19,879 (TS=mixed method*) AND LANGUAGE: (English)

6 8,541 (TS=grounded theory) AND LANGUAGE: (English)

5 40,298 (TS=focus group*) AND LANGUAGE: (English)

4 2,062 (TS= (qualitative NEAR/1 studies)) AND LANGUAGE: (English)

#3 15,953 ((TS= (qualitative NEAR/1 study))) AND LANGUAGE: (English)

2 8,468 ((TS= (qualitative NEAR/1 method*))) AND LANGUAGE: (English)

1 12,460 (TS= (qualitative NEAR/1 research)) AND LANGUAGE: (English)

Cumulative Index of Nursing & Allied Health Literature

Search run 25th July 2018

URL: http://search.ebscohost.com/

S18 S7 AND S16 Limiters - Published Date: 20100101-20181231 Narrow by Language: - English (278)

S17 S7 AND S16

S16 S8 OR S9 OR S10 OR S11 OR S12 OR S13 OR S14 OR S15

S15 TX discourse analysis OR TX discursive OR TX thematic analysis OR TX ethnography OR TX action research OR TX phenomenological

S14 TX qualitative exploration OR TX qualitative evaluation OR TX qualitative intervention* OR TX qualitative approach OR TX qualitative analysis OR TX qualitative data

S13 TX mixed method* OR TX semi structured interview* OR TX in depth interview*

S12 TX focus group* OR TX grounded theory OR TX narrative analysis

S11 TX qualitative n3 research OR TX qualitative n3 method* OR TX qualitative n3 study

S10 (MH "Focus Groups")

S9 (MH "Semi-Structured Interview") OR (MH "Structured Interview") OR (MH "Narratives")

S8 (MH "Qualitative Studies+")

S7 S3 AND S6

S6 S4 OR S5

S5 TX ((recruit$ or participat$ or take part or dropout$ or drop$ out$ or withdr?wl$ or barrier$ or retention or response$ or respond$ or attrition) N10 trial?)

S4 (MH "Research Subjects+")

S3 S1 OR S2

For peer review only - http://bmjopen.bmj.com/site/about/guidelines.xhtml

S2 TX ranndomized or randomised or trial*

S1 (MH "Clinical Trials+")

Applied Social Sciences Index and Abstracts

Search run 25th July 2018

http://search.proquest.com/assia/

S8 S5 and S6Limits applied Language:English PY: 2010-2018 (373)

S7 S5 and S6

S6 (recruit* N/10 trial?) OR (participat* N/10 trial? OR "take part" N/10 trial?) OR (dropout* N/10 trial? OR drop* our* N/10 trial?) OR (withdraw* N/10 trial* OR barrier* N/10 trial?) OR (retention N/10 trial? OR response* N/10 trial?) OR (respond* N/10 trial? OR attrition N/10 trial?)

S5 S3 and S4

S4 qualitative OR (focus group* OR interview*) OR (mixed method* OR ethnography) OR (phenomenological OR discourse analysis) OR discursive

S3 S1 or S2

S2 randomized OR randomised

S1 SU.EXACT("Clustor randomized trials") OR SU.EXACT("Clinical randomized controlled trials") OR SU.EXACT("Single blind randomized controlled trials") OR SU.EXACT("Cluster randomized controlled trials") OR SU.EXACT("Randomized controlled trials") OR SU.EXACT("Double blind randomized trials") OR SU.EXACT("Prospective controlled trials") OR SU.EXACT("Double blind randomized controlled trials") OR SU.EXACT("Clinical trials")

For peer review only - http://bmjopen.bmj.com/site/about/guidelines.xhtml

S1 Box ~~Steps of Meta-ethnography~~Our analytical approach

1) ~~Identifying the research question~~Getting started

This stage involved us generating a research question specific to our area of interest that we believed could be usefully addressed by referring to qualitative research. In our case this was the question of what influences non-retention within clinical trials from the perspectives of trial withdrawers?

2) ~~Identifying relevant studies~~Describing what is relevant to initial interest

This stage involved making a series of decisions relating to deciding what was relevant to our initial area of interest, deciding on the searching process, inclusion/exclusion decisions and quality assessment. In our case we were interested in any study that reported the use of qualitative methods (for collection and analysis of data) to explore the reasons why individual participants withdraw from clinical trials. We were interested in any reports made by participants themselves or by trial staff, but this had to be specifically in relation to why participants withdraw. We defined withdrawal or non-retention as covering any aspect of attrition recognising that this might cover activities such as cessation of, or withdrawal from the intervention(s), non-attendance at clinic visits, through to non-response to some or all follow up questionnaires etc. We decided that we would exclude studies that did not use qualitative ~~data~~ means to collect or analyse their data and also studies reporting findings from trial withdrawers who were not patient participants e.g. GPs in a primary care cluster trial. Following these decisions, a systematic search across a range of databases was conducted with assistance from an information specialist (See S1 Appendix) and all titles and abstracts were screened for inclusion (see S1 and S2 Figures). Applying quality criteria to qualitative research remains a contentious issue and there is no consensus regarding whether and how this should be done (Mays 2000; McEwan 2004). However, one author (ZS) undertook a quality assessment of each of the ~~8~~ 11 papers that were identified as being eligible for inclusion in the synthesis. Whilst authors of some qualitative evidence syntheses have chosen to exclude what they deem to be poor quality papers, we made the decision not to exclude any of the identified papers. Although all papers had study aims that were amenable to investigation via qualitative means and all included qualitative data, as a team we deemed some as being richer than others in terms of data and insights (i.e. first and second order constructs). Despite this variation in the overall level of quality, due to the small number of identified studies we considered it more important to retain any relevant findings than disregard based on study quality. In doing so, we would argue that all ~~8~~ 11 papers contributed useful elements to the collective whole and enabled us to develop our line of argument in terms of the issues of importance regarding trial non-retention.

3) Reading the studies

At this stage, we aimed to become as familiar as possible with the content of all the identified papers with each author independently reading through all of the data provided and making detailed notes of their observations including identification of preliminary themes. After sharing notes, we met to discuss our findings as a team, comparing and contrasting our preliminary observations etc. We repeated this process for the 3 papers that we identified in our updated database search.

4) ~~Identifying themes~~Determining how the studies are related

In describing this phase, Noblit and Hare 1988 state that "In doing a synthesis, the various studies must be 'put together'. This requires determining the relationships between the studies to be

For peer review only

synthesized. We think it makes sense to create a list of the key metaphors, phrases, ideas, and/or concepts (and their relations) used in each account and to juxtapose them…" During this next stage (which in practice we found very much related to activities undertaken as part of stage 3), data was then extracted initially from all 8 papers (retrieved from the 1st database search) using a standard form which summarised the main phrases, themes and ideas, along with, information regarding methods, and any other important information relating to the context of the research (some of this data is illustrated in S1 Table). During this stage, we focussed on both 1st order constructs within included papers (meaning study participant quotations found in the results section of papers) along with 2nd order constructs (meaning the interpretations made by the papers' authors, usually found in the discussion and conclusion sections of papers but also sometimes within the results). Using the standard form, the papers were initially organised in chronological order (but as inductive analysis progressed papers were grouped according to emerging themes) and we focused on the findings, concepts and themes used by the papers' authors generating a list of key categories. This document (along with our other written notes and observations) facilitated discussions at a series of subsequent team meetings and were very useful for consideration of how identified themes from one paper might relate to the others. We added similar data from the additionally identified 3 papers to this form, to allow us to compare and contrast findings with the earlier 8 papers.

5) Translating the findings of each study into those of the othersstudies into one another

5) Noblit and Hare 1988 state that "In its simplest form, translation involves treating the accounts as analogies: One program is like another except….It also compares both the metaphors or concepts and their interactions in one account with the metaphors and their interactions in the other accounts."

At this key stage (which again in practice we found inter-related to stage 4), following this process, we sought to consider the extent to which themes and concepts seemed common or distinct across the papers. Our initial grouping of first 1st and second order2nd order constructs across the 8 papers resulted in 14 sub-themes. These were issues/ideas that we each considered important in terms of things that might make people withdraw from trials. During the process of translating themes/concepts from each of the individual studies into those of the others (i.e. comparing and contrasting across studies), following further team discussion these were then grouped and categorised into 5 broad key themes (as it became apparent that some of sub-themes were related or overlapped). We interpreted our 5 key themes which we interpreted as characterising the main considerations and features that appeared to influence non-retention in the trials under investigation (See S2 Table). For the 3 subsequently identified papers, we repeated this stage by comparing and contrasting concepts and their interactions in these 3 accounts with the concepts identified in the original 8 accounts– in essence comparing for 'fit' and checking for any additional themes (Lang 2013). During this process, we were confident that concepts identified in the later 3 papers supported and complemented our originally identified 5 key themes with no new themes emerging.

6) Synthesizsing the findingstranslations

Noblit and Hare 1988 state that "Synthesis refers to making a whole into something more than the parts alone imply." For our synthesis, what we were attempting to do at this stage was to move towards an explanatory analysis. we We considered and discussed both the 1st and 2nd order constructs across each of the papershow the various translations compared in an attempt to develop a more nuanced and collective understanding of factors influencing trial non-retention (in doing so, developing our 'line of argument' synthesis). As before, we did this through a process of reflection and team discussions, in an attempt to produce overarching insights into the factors that appear to influence non-retention.

Formatted: Superscript

Formatted: No bullets or numbering

Formatted: Superscript

Formatted: Superscript

Formatted: Font: Not Bold

For peer review only

7) Expressing the synthesis

As is common with other meta-ethnographies we sought to express our collective insights in both textual and diagrammatic format within our paper. In doing so, we expressed our synthesis both within our paper as our 'line of argument' (with supportive illustrative data from across the studies) and also as a conceptual diagram (see S32 Figure).

For peer review only

	Nakash 2008	Nicholas 2010	Postel 2010	Eborall 2011	Wells 2011	Shilling 2011	Sanders 2012	Johannson 2015	Sari 2017	Fernandez-Alvarez 2017	Henshall 2018
Was there a clear statement of the aims of the research?	Yes	Yes	Yes	No – not explicitly in abstract or background	Yes	Yes	Yes	Yes	Yes	Yes	Yes
Is a qualitative methodology appropriate?	Yes	Yes	This reported some qualitative data from open ended questionnaire response options	Yes	Yes	Yes	Yes	Yes	Yes	Yes	Yes
Was the research design appropriate to address the aims of the research?	Yes	Yes	Unclear	Yes	Yes	Yes	Yes	Yes	Yes	Yes	Yes
Was the recruitment strategy appropriate to the aims of the research?	Yes	Yes	Yes	Yes	Yes	Yes	Yes	Yes	Yes	Yes	Yes
Was the data collected in a way that addressed the research issue?	Yes	Yes	Unclear	Yes	Yes	Yes	Yes	Yes	Yes	Yes	Yes
Has the relationship between researcher and participants been adequately considered?	Unclear – not mentioned in paper	Unclear - not mentioned in paper	Unclear	Unclear – status of interviewer mentioned but not discussed further	Unclear - not mentioned in paper	Unclear- not mentioned in paper	Unclear – not mentioned in paper	Yes, this was discussed	Yes, this was discussed	Yes, this was discussed	Yes, this was discussed

For peer review only

Have ethical issues been taken into consideration?	Yes	Yes	Yes	Yes	Unclear	Yes	Unclear	Yes	Unclear	Unclear – informed consent mentioned, but not ethical approvals	Unclear – informed consent mentioned, but not ethical approvals
Was the data analysis sufficiently rigorous?	Yes, apparently so (although not huge detail)	Yes	Unclear – not mentioned other than that the data was systematically analysed – reflects that it was not a qualitative study as such.	Yes	Unclear – very brief details and presented in quantitative manner	Yes	Yes	Yes	Yes	Yes	Yes
Is there a clear statement of findings?	Yes	Yes	Yes, but brief	Yes	Yes	Yes, but focus of report was not on reasons for withdrawing and so this was somewhat buried and limited.	Yes	Yes	Yes	Yes	Yes
How valuable is the research to our review?	Useful findings – NB: Limited 1st order construct data perhaps because paper also included data from trial retainers (and this seemed to be main emphasis)	Useful – perhaps one of the richer papers in terms of insights and data	Less useful than other studies but still provides helpful insights that help to build on the findings of other studies. Qualitative data presented was very limited (both 1st and 2nd	Useful – more 1st order constructs than some of the other papers e.g. Nakash, Sanders	Less useful than other studies but still provides helpful insights that help build on other studies. Qualitative data presented was very limited	Useful – but study focussed on decliners and withdrawers and provided limited data from the latter.	Useful – but only 3 were withdrawers, rest were decliners and so like Nakash the emphasis of the paper (and the bulk of data and reflection provided) was on decliners. Few 1st order	Useful – perhaps one of the richer papers in terms of insights and data	Useful, although limited 1st order constructs	Useful – more 1st order constructs than some of the other papers e.g. Nakash, Sanders	Useful findings – NB: Limited 1st order construct data perhaps because paper also included data from trial retainers – out of 20 participants interviewed,

For peer review only

			order constructs) and was discussed in a more quantitative way.		(both 1st and 2nd order constructs) and was discussed in a more quantitative way.		constructs from withdrawers.				only 4 had dropped out.

For peer review only

Enhancing transparency in reporting the synthesis of qualitative research: ENTREQ

ENTREQ Statement: content and rationale

The ENTREQ statement consists of 21 items grouped into five main domains: introduction, methods and methodology, literature search and selection, appraisal, and synthesis of findings (Table 1). For each item, a descriptor and examples are provided. Below we present a rationale for each domain and its associated items.

Table 1

Enhancing transparency in reporting the synthesis of qualitative research: the ENTREQ statement

No	Item	Guide and description	
**1**	Aim	State the research question the synthesis addresses.	See Page 3
**2**	Synthesis methodology	Identify the synthesis methodology or theoretical framework which underpins the synthesis, and describe the rationale for choice of methodology *(e.g. meta-ethnography, thematic synthesis, critical interpretive synthesis, grounded theory synthesis, realist synthesis, meta-aggregation, meta-study, framework synthesis).*	See Pages 3-4 and S1 Box
**3**	Approach to searching	Indicate whether the search was pre-planned (*comprehensive search strategies to seek all available studies)* or iterative (*to seek all available concepts until they theoretical saturation is achieved)*.	See Page 3
**4**	Inclusion criteria	Specify the inclusion/exclusion criteria *(e.g. in terms of population, language, year limits, type of publication, study type).*	See Page 3-4
**5**	Data sources	Describe the information sources used (e.g. *electronic databases (MEDLINE, EMBASE, CINAHL, psycINFO, Econlit), grey literature databases (digital thesis, policy reports), relevant organisational websites,*	See Page 3

No	Item	Guide and description	
		experts, information specialists, generic web searches (Google Scholar) hand searching, reference lists) and when the searches conducted; provide the rationale for using the data sources.	
**6**	Electronic Search strategy	Describe the literature search *(e.g. provide electronic search strategies with population terms, clinical or health topic terms, experiential or social phenomena related terms, filters for qualitative research, and search limits).*	See Page 3 and Appendix 1
**7**	Study screening methods	Describe the process of study screening and sifting *(e.g. title, abstract and full text review, number of independent reviewers who screened studies).*	See Page 3-4
**8**	Study characteristics	Present the characteristics of the included studies *(e.g. year of publication, country, population, number of participants, data collection, methodology, analysis, research questions).*	See Page 4-5
**9**	Study selection results	Identify the number of studies screened and provide reasons for study exclusion *(e,g, for comprehensive searching, provide numbers of studies screened and reasons for exclusion indicated in a figure/flowchart; for iterative searching describe reasons for study exclusion and inclusion based on modifications t the research question and/or contribution to theory development).*	See Page 4 and Figure 1 and 2
**10**	Rationale for appraisal	Describe the rationale and approach used to appraise the included studies or selected findings *(e.g. assessment of conduct (validity and robustness), assessment of reporting (transparency), assessment of content and utility of the findings).*	See Page 3-4

No	Item	Guide and description	
**11**	Appraisal items	State the tools, frameworks and criteria used to appraise the studies or selected findings *(e.g. Existing tools: CASP, QARI, COREQ, Mays and Pope* [25]*; reviewer developed tools; describe the domains assessed: research team, study design, data analysis and interpretations, reporting).*	See Page 4 and S1 CASP checklist
**12**	Appraisal process	Indicate whether the appraisal was conducted independently by more than one reviewer and if consensus was required.	See Page 4. 1 reviewer (the main author) initially assessed quality of included studies using the CASP criteria and noted any critical aspects of quality with the study team. During subsequent group discussions we continued to discuss and reflect on key aspects of quality. Due to the small number of eligible studies we decided to include all (please see discussion section and also S1 CASP checklist)
**13**	Appraisal results	Present results of the quality assessment and indicate which articles, if any, were weighted/excluded based on the assessment and give the rationale.	Please see discussion section and S1 CASP checklist
**14**	Data extraction	Indicate which sections of the primary studies were analysed and how were the data extracted from the primary studies? *(e.g. all text under the headings "results /conclusions" were extracted electronically and entered into a computer software).*	See Page 4 and S1 Box
**15**	Software	State the computer software used, if any.	N/A
**16**	Number of reviewers	Identify who was involved in coding and analysis.	See Pages 4
**17**	Coding	Describe the process for coding of data *(e.g. line by line coding to search for concepts).*	See Page 4

No	Item	Guide and description	
**18**	Study comparison	Describe how were comparisons made within and across studies *(e.g. subsequent studies were coded into pre-existing concepts, and new concepts were created when deemed necessary).*	See Page 4, S1 Box, S3 Table
**19**	Derivation of themes	Explain whether the process of deriving the themes or constructs was inductive or deductive.	See Page 4,S1 Box, S3 Table
**20**	Quotations	Provide quotations from the primary studies to illustrate themes/constructs, and identify whether the quotations were participant quotations of the author's interpretation.	See Results section and S2 Table
**21**	Synthesis output	Present rich, compelling and useful results that go beyond a summary of the primary studies (e.g. *new interpretation, models of evidence, conceptual models, analytical framework, development of a new theory or construct).*	See Results and discussion section. Also see our conceptual model illustrating our 'line of argument' (S3 Figure).

For peer review only - http://bmjopen.bmj.com/site/about/guidelines.xhtml

PRISMA Flow Diagram: Retention Review

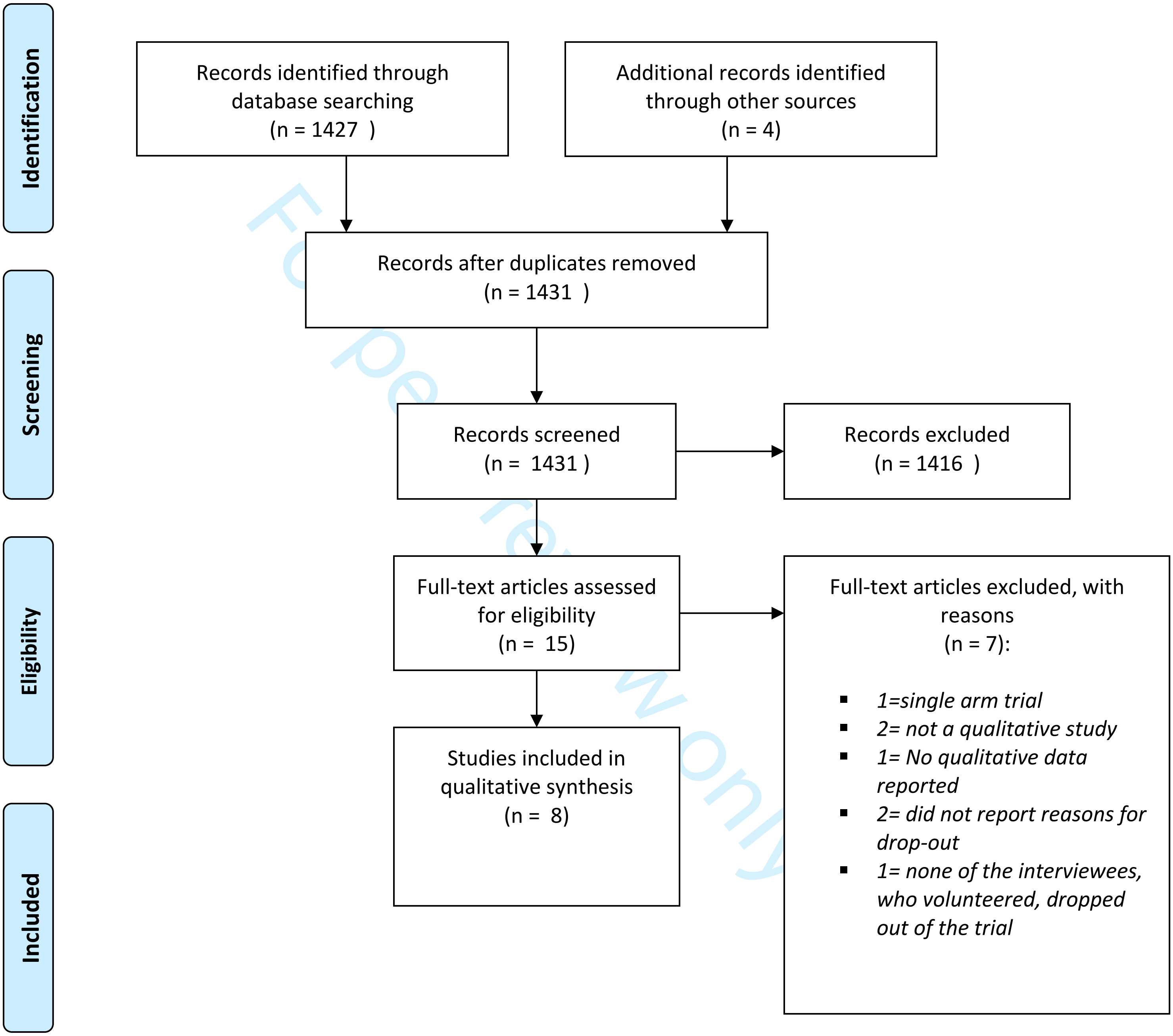

For peer review only

PRISMA Flow Diagram: Retention Review (search update)

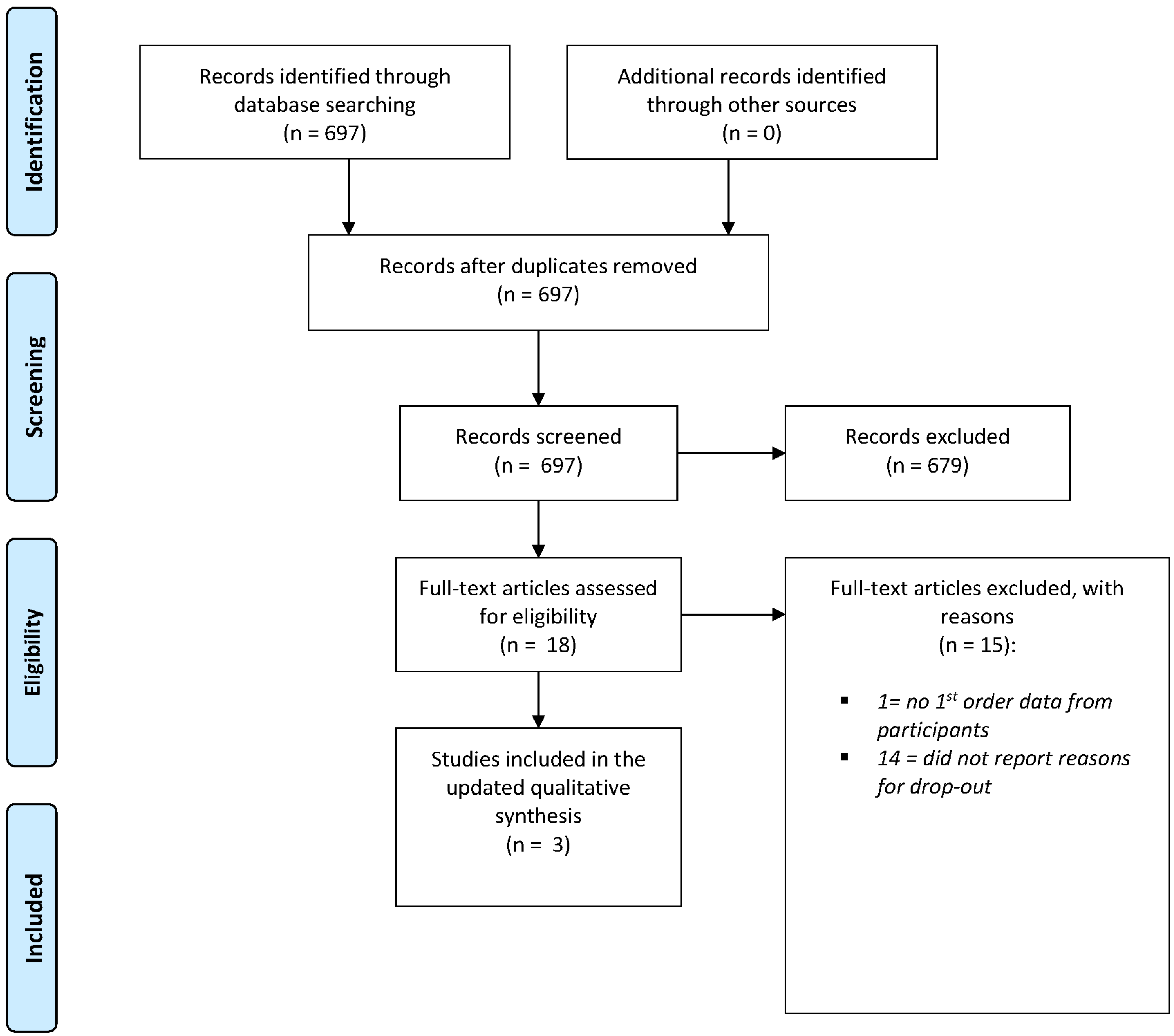

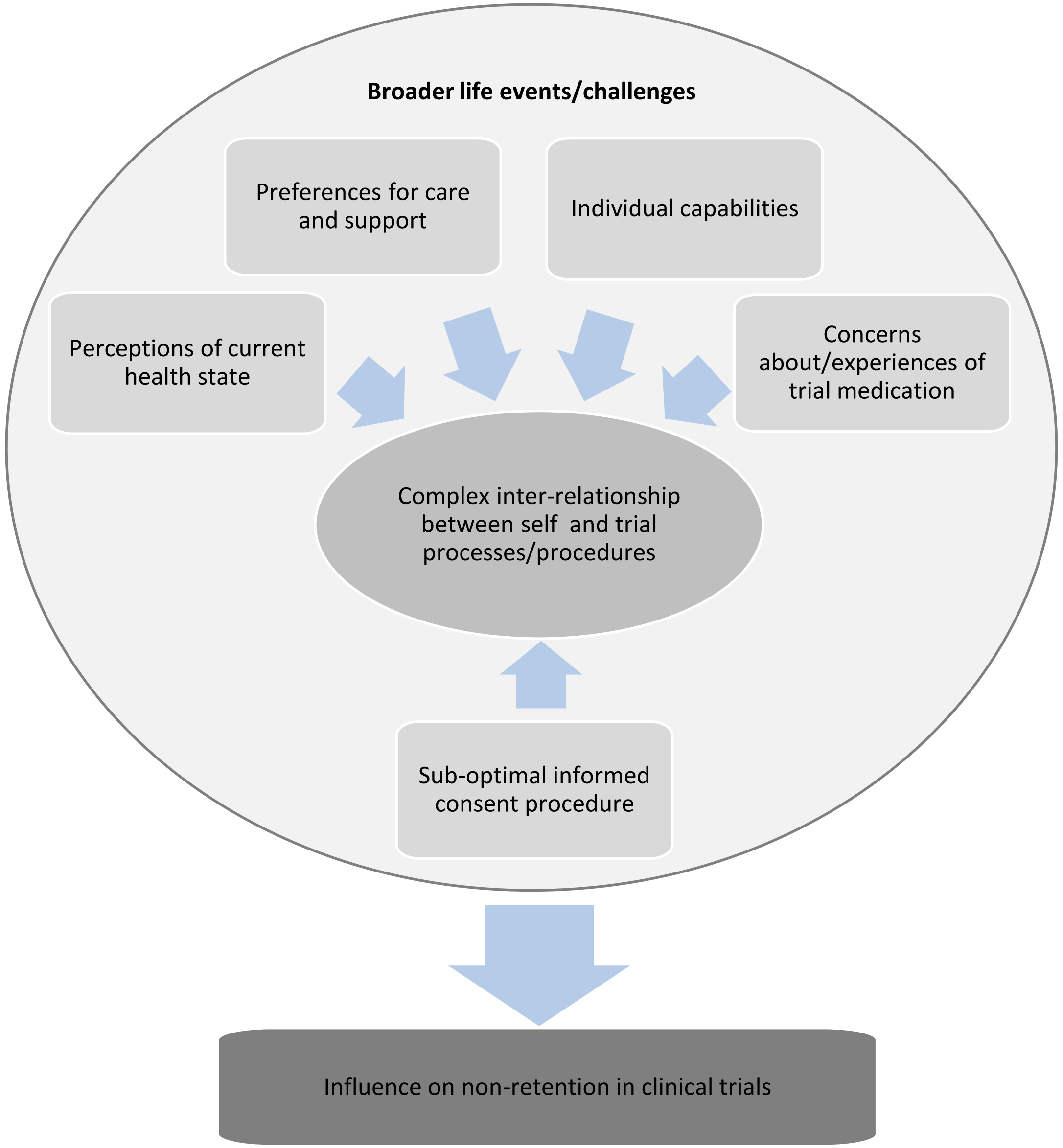
Broader life events/challenges
Preferences for care and support
Individual capabilities
Perceptions of current health state
Concerns about/experiences of trial medication
Complex inter-relationship between self and trial processes/procedures
Sub-optimal informed consent procedure
Influence on non-retention in clinical trials

S1 Table Characteristics of included studies

REF	COUNTRY	AIM	CONDITIONS OF FOCUS	PARTICIPANT CHARACTERISTICS	TRIAL COMPARATORS	ATTRITION BEHAVIOUR	DATA COLLECTION METHODS
Nakash et al 2007 [~~28~~31]	UK	To examine factors affecting response and non-response from the clinical trial participant's perspective.	Severe ankle sprains	8 non-retainers. Age and gender of non-retainers unclear although of the 22 included in this study, 11 were male; 11 female, aged from 16 to 62 yrs (mean age 34 yrs). A purposive sample was sought to represent the diversity of trial participants in age, sex, level of education, occupation and type of ankle support.	Different mechanical supports	Non-response to postal questionnaire follow-up. 8 had not responded to at least one of their follow up questionnaires.	Semi-structured interviews
Nicholas et al 2010 [21]	Australia	To identify participants' reasons for non-adherence to, and attrition from the online intervention	Newly diagnosed bipolar disorder	39 non-retainers. 22 female; 17 male. 20 were aged less than 30 yrs, 14 were married, 29 were tertiary educated and 24 in full time employment. Participants from all 3 study groups were interviewed, 16 from the unsupported intervention group (BEP), 9 from the supported BEP intervention group (BEP+IS), and 14 from the minimal	1 of 2 active interventions (online psycho-education program either alone or with email support from informed supporters) or an attention control condition (online information about bipolar disorder presented in text as bullet points). Both active interventions and control contained 'workbook' activities.	Cessation of therapy sessions and/or non-completion of some or all intervention workbooks; non-completion of some or all control workbooks.	Semi-structured interviews

For peer review only

For peer review only - http://bmjopen.bmj.com/site/about/guidelines.xhtml

S1 Table Characteristics of included studies

				information control group.			
Postel et al 2010 [22]	Netherlands	To evaluate an e-therapy program with active therapeutic involvement for problem drinkers. Reasons for drop out were also investigated via a 'dropout' questionnaire consisting mainly of open questions.	Problem drinking	40 non-retainers. No gender or age details provided specifically for non-retainers, although 53.8 % of trial participants were female, mean age 45.3 yrs. Authors also state that in the control group more non-responders than responders were male.	3 month e-therapy programme (consisting of a structured 2 part online treatment programme with asynchronous therapist contact via the internet only) or a waiting list control group (receiving 'no reply' email messages once every 2 weeks). Participants completed online self-report questionnaires at baseline and at 3 months follow up (control group) or at posttreatment, which was approximately 3 months (e therapy group). Weekly alcohol consumption was assessed by a 7 day retrospective drinking diary.	Non-completion of treatment sessions and/or follow-up questionnaires. Non-retention was defined as anyone who did not complete the 3 month assessment. Dropouts in the e therapy group did not complete all 12 treatment sessions: 9 assignments and 3 assessments.	A 'dropout' questionnaire consisting mainly of open questions.
Eborall et al 2011 [2730]	UK	To explore people's explanations for declining to participate in the trial, or, having begun the trial, stopping the trial medication	Asymptomatic atherosclerosis	17 non-retainers. No gender or age details provided specifically for non-retainers (as opposed to those who declined to consent), but states that total sample (n=28) had a mean age of 65.2 yrs; 19 were female; wide	Aspirin (100mg daily) or placebo for a mean duration of 8.2 yrs.	Cessation of trial medication (unclear whether active medication or placebo). Non-adherence with study medication throughout the trial was 40%; 15% took their medication for less than 6 months.	Semi-structured interviews (n=11) and one focus group (n=6)

For peer review only

S1 Table Characteristics of included studies

				range of socioeconomic backgrounds.			
Wells et al 2011 [23]	USA	To explore low-income, minority cancer patient perspectives about not adhering or dropping out of depression treatment.	Depression and cancer	20 non-retainers. No gender or age details provided specifically for non-retainers included in the qualitative study but trial non-retainers were described as predominantly female, foreign born, unmarried, unemployed, and older than 50 yrs.	Intervention or usual care. Intervention was an individualised stepped care depression programme provided by a cancer depression clinical specialist in collaboration with a study psychiatrist. Patients in the intervention group were offered antidepressant medication and/or problem solving treatment (PST).	Cessation of problem solving treatment sessions and/or anti-depressant medication.<br><br>PST dropouts were defined as patients who had fewer than 4 PST sessions. PST dropouts included those who initially agreed to be randomised to the intervention, but thereafter had either verbally declined treatment or did not show up for the therapy appointments. This included patients who had refused some sessions, but agreed to remain in the study for outcome interviews.<br><br>Patients receiving antidepressant medication were dropouts if they discontinued treatment within 30 days.	Semi-structured interviews
Shilling et al 2011 [275]	UK	To investigate recruitment processes across a range of clinical trials and from the perspective of parents, young people and practitioners to identify strategies to	Trial 1: Neuro-development disorders<br><br>Trial 2: Osteopenia	3 non-retainers from 2 of the 4 included trials. In trial 1, participants were 11-14 yrs; In trial 2, participants were 4-18 yrs (although demographic details of non-retainers unclear).	Trial 1: Melatonin versus placebo (over 12 week treatment period). Families made 3 additional hospital visits, and received 4 home visits by the research nurse and 3 telephone calls.	Cessation of trial medication (unclear whether active medication or placebo)	Semi-structured interviews (with the young people and/or their parents).

For peer review only

S1 Table Characteristics of included studies

		improve recruitment and its conduct across a spectrum of trials of medicines for children.<br><br>NB: This was a monograph that included the reporting of a range of trials, two of which reported data on non-retainers and were included in this meta-ethnography.			Trial 2: bisphosphonate risedronate or vitamin D analogue 1 – alphahydroxychol ecalciferol versus placebo (1 yr treatment period). Young people were seen 7 times over the year. This was timed to coincide with routine clinic visits where possible. Blood samples were also taken (same time as routine visits) and they gave regular urine samples and had 3 x-ray scans and 2 bone radiographs.		
Sanders 2012 [28~~6~~]	UK	To explore barriers to participation and adoption of tele-health and tele-care from the perspective of people who declined to participate or withdrew from the trial	Diabetes, COPD, heart failure, or social care needs	3 non-retainers (all in the intervention arm), 1 female (diabetes); 2 male (1 x COPD; 1 x diabetes + heart and lung problems. 73-85 yrs.	The RCT was a cluster design with GP practices being randomised to receive access to telehealth or telecare for their populations. Participants randomised to the control arm were offered telehealth or telecare at the end of the 12mth trial.<br><br>Tele-health equipment included a monitor unit via which recordings from peripheral devices were uploaded to a monitoring centre. The monitoring centres prioritised and	Cessation of use of telehealth equipment or tele care devices (defined as withdrawing from the trial after joining the intervention arm).	Semi-structured interviews

For peer review only

S1 Table Characteristics of included studies

					tailored response according to need based on the information received.<br><br>Telecare interventions also varied according to assessed need but included various sensors to detect gas, water overflow, falls and movement around the property. Such sensors would trigger alarms direct to a monitoring centre if anything abnormal was detected, allowing emergency intervention.		
Johansson 2015 [24]	Sweden	To explore participants' experiences of non-adherence to internet-delivered psychological treatment	Generalised anxiety disorder	7 non-retainers, 6 female; 1 male; mean age 39.3 yrs	Intervention consisted of internet delivered psychological treatment, with weekly support from a licensed clinical psychologist. The treatment consisted of 8 weekly self-help modules of text, audio and illustrations, averaging 21 pages per module. All modules contained a homework assignment that needed to be answered and sent to the guiding therapist.	Non-adherence to internet delivered psychological treatment.<br><br>Non-adherence was defined as completing at least 1 and no more than 7 treatment modules (out of 8 in total).	Semi-structured interviews

For peer review only

S1 Table Characteristics of included studies

					No details re. control group given.		
Sari 2017 [25]	Denmark	To investigate perceived barriers to participate in an exercise intervention among alcohol use disorder patients, who dropped out from the Healthy Lifestyle Study	Alcohol Use Disorder	17 non-retainers, 4 female; 13 male; age 30-68 yrs. Nine were allocated to the group intervention, 7 were allocated to the individual intervention and one were allocated to the control group.	Treatment as-usual or one of two 6-month interventions that were selected on the basis of existing evidence-based studies. In the first intervention group, participants exercised individually after receiving basic instructions and a training program for home use. In the second intervention group, several patients exercised together with two instructors in 60-min training sessions twice a week. Running was the specific exercise form for both groups in the study.	Drop out was indicated when participants directly reported to project personnel or when they ceded participation without contact. Their length of participation before dropout varied from 2 days to 12 weeks.	Semi-structured interviews
Fernandez-Alvarez 2017 [26]	Spain	To conduct a qualitative analysis of the subjective experience of a sample of patients who dropped out of a transdiagnostic Internet based treatment for emotional disorders.	Obsessive-compulsive disorder; Agoraphobia;Social Anxiety Disorder; Major Depressive Disorder; Generalized Anxiety Disorder	10 non-retainers, 8 female; 2 men, age 21-59 yrs. Unclear whether control or treatment arm.	The protocol consists of 12 modules, and participants are encouraged to complete one module per week. Two RCTs are being conducted using the protocol. The purpose of one of the RCTs is to analyze the effectiveness of a transdiagnostic IBT compared to treatment as usual as provided in the Spanish public mental	~~No specific details given other than '18 I~~individuals who dropped out of ~~these two RCTs'~~treatment after completing a minimum of 3 modules.	Semi-structured interviews

For peer review only

S1 Table Characteristics of included studies

					health care system. Both treatment protocols are web-based, self-administered treatments with minimum contact/support from a therapist that consists in a weekly phone call lasting 5 to 10 min to each participant. A non-human support is delivered through two weekly mobile phone text messages that are automatically sent and aim to remind the participants of the importance of reviewing the modules as well as doing the homework tasks.		
Henshall 2018 [29]	UK	To determine the overall experiences of newly diagnosed adults with T1D in an exercise study, and to understand issues that influence the retention of trial participants in such studies	Newly diagnosed Type 1 diabetes	4 non-retainers; 2 female; 2 male age 19 - 55 yrs (2 in intervention arm/2 in control arm)	Conventional treatment or exercise, stratified on beta cell function and fitness. The exercise group were encouraged to increase their level of activity to a minimum of 150 minutes of moderate to vigorous intensity exercise per week, aiming for 240 minutes per week of exercise for 12 months.	No specific details given other than 'those who withdrew before the end of the study'	Semi-structured interviews

For peer review only

S1 Table Characteristics of included studies

For peer review only

S2 Table Key themes of influence on decisions to withdraw from trial participation, with corresponding example data.

Theme	Exemplary quote
**Perceptions of current health state in relation to specific aspects of the trial**	*"A very short while after doing the program I fell into another episode, a depressive episode, and pretty much stopped doing everything, the program included"[21, male, 18-29 yrs, BEP+IS group]*<br><br>*"I found it quite confronting, and reading the information made me feel uncomfortable, thinking that these issues related to me – I preferred the ostrich approach" [21, male, 40-49 yrs, BEP group]*<br><br>**This study demonstrates that reasons…were often explained in terms of potential threats to existing self-care, independence, the majority of respondents in this study depicted themselves as too healthy and too independent for the interventions to be of value [28, telehealth and telecare interventions in aging populations]**
**The 'fit' of aspects of the trial with individual preferences for care and support**	**Two respondents who withdrew from the trial described how the service changes they experienced caused additional stress. For example, one woman said she** *'did not want to be a nurse' …***and she was much happier to have returned to a regular appointment (fortnightly) with the community matron. Another man described the good care he received prior to joining the trial, but how he was subsequently discharged from the specialist professionals who had been involved in his care…..he described his main problems as** *'complex problems with my heart and breathing,'* **and that the faulty recordings and changes in service provision were causing him great stress [28, telehealth and telecare interventions in aging populations]**<br><br>**Some participants changed to other treatment formats after terminating the Internet-delivered therapy. One participant explained that she had never prioritised her own personal development and that an individual therapy consisting of face to face meetings was needed to get away from home and focus on the therapy [24, internet-delivered psychological treatment for people with generalised anxiety disorder]**
**The compatibility of aspects of the trial with individual capabilities**	**Twelve of the 20 patients interviewed acknowledged that they had dropped out of treatment, citing several reasons and circumstances related to dropping out of treatment. These**

	**included…cultural (which included language communication problems) [23, problem solving treatment sessions and/or anti-depressant medication for people with depression and cancer]**<br><br>**Difficulty with literacy [31, Different mechanical supports for people with severe ankle sprains]**<br><br>**One critique was that information was difficult to understand because the content was perceived as complex and abstract. In some cases the participants felt unintelligent for their inability to understand [24, internet-delivered psychological treatment for people with generalised anxiety disorder]**
**Concerns about or experiences of trial medication**	**One mother's discovery, via an internet search done by the child's father, that the trial drug was unlicensed for children had left her concerned about the safety of the trial and she subsequently withdrew her child from MENDS [trial] [27]**<br><br>*"I really felt I'd got gall bladder trouble again because [the pain] was from here right through into me kidneys and really severe. So I went to my GP, and she just checked round and said straight away, 'don't take anymore, and ring [the trial] and tell them'"[30, Aspirin for people with Asymptomatic atherosclerosis]*
**Considerations around extent to which trial participation could be appropriately accommodated into broader life circumstances**	**The participants' statements regarding non-adhering showed an incompatible relationship between the length of the weekly text modules and factors or conditions in the personal life of the participants [24, internet-delivered psychological treatment for people with generalised anxiety disorder]**<br><br>*"…as a student you read so much already. I felt like I couldn't muster more energy or more time to spend by the computer and to read 10 or 20 more pages and also answer questions. It felt as if you were inclined to have a very structured life already to handle that" [24, internet-delivered psychological treatment for people with generalised anxiety disorder]*<br><br>**These were reasons such as pregnancy, exams, or work commitments which participants felt were the cause of their non-response [31 Different mechanical supports for people with severe ankle sprains]**

For peer review only

	**We could establish that in the e-therapy group 11 participants dropped out because of personal reasons unrelated to the e-therapy program or the study (eg, ill family member) [22]**

Primary study participant quotes (1st order constructs) are displayed in italics and primary study author interpretations (2nd order constructs) are presented in bold. Study references and details of participants/interventions where available have been added to the end of exemplar quotes.

For peer review only

S3 Table

	Inter-relationship between self and trial process/procedures													
**Themes**	**1. Perceptions of current health state in relation to specific aspects of the trial**		**2. The 'fit of aspects of the trial with individual preferences for care and support**						**3. The compatibility of aspects of trial processes with individual capabilities**		**4. Concerns about or experiences of trial medication**			**5. Considerations around the extent to which trial participation could be appropriately accommodated into individuals' broader lives**
14 Sub-themes	Perception of being too well	Perception of being too ill	Not individual /tailored/ personalised enough	Too basic	Inflexible	Too technical	Too intensive	Too stressful	Not tailored to individual capabilities	Cognitively not pitched at the individual	Medication not necessary	Potentially dangerous	Contraindications or side effects	Aspects of life getting in the way
Nakash 2008	*								*	*				*
Nicholas 2010	*	*	*	*	*									*
Postel 2010	*						*	*						*
Eborall 2011	*										*		*	*
Wells 2011	*	*							*	*				
Shilling 2011												*	*	
Sanders 2012	*			*	*	*								
Johansson 2015		*	*		*				*	*				*
Sari 2017	*						*							*
Fernandez-Alvarez 2017		*	*	*										*
Henshall 2018		*												*

*denotes presence of themes across papers

For peer review only

BMJ Open

Exploring non-retention in clinical trials: A meta-ethnographic synthesis of studies reporting participant reasons for drop out

Journal:	*BMJ Open*
Manuscript ID	bmjopen-2018-021959.R2
Article Type:	Research
Date Submitted by the Author:	25-Feb-2019
Complete List of Authors:	Skea, Zoë; Universit of Aberdeen, Health Services Research Unit<br>Newlands, Rumana ; University of Aberdeen College of Life Sciences and Medicine, Health Services Research Unit<br>Gillies, Katie; University of Aberdeen, Health Services Research Unit
**Primary Subject Heading**:	Health services research
Secondary Subject Heading:	Qualitative research
Keywords:	QUALITATIVE RESEARCH, Clinical trials < THERAPEUTICS, Retention

SCHOLARONE™ Manuscripts

Exploring non-retention in clinical trials: A meta-ethnographic synthesis of studies reporting participant reasons for drop out

Author: Dr Zoë Christina Skea,

Corresponding author: Dr Zoë Christina Skea, Health Services Research Unit, 3rd Floor, Health Sciences Building, University of Aberdeen, Aberdeen, AB25 2ZD, Tel: +44 (0) 1224 438153. email:z.skea@abdn.ac.uk. Fax: +44 (0) 1224 438165.

Co-authors: Dr Rumana Newlands, University of Aberdeen, Aberdeen, UK. Dr Katie Gillies, University of Aberdeen, Aberdeen, UK.

Key words: Qualitative research; Clinical Trials; Retention.

Word count: 7328

Abstract

Objectives

To undertake a meta-ethnographic synthesis of findings from primary studies reporting qualitative data that have explored participant reported factors influencing non-retention within a clinical trial context.

Design

A systematic search and meta-ethnography was conducted for published papers (from 1946 –July 2018) that contained qualitative data from trial non-retainers.

Participants

We identified 11 studies reporting qualitative data from 13 trials. The studies were undertaken between 2008 and 2018. Each study included between 3 and 40 people who had dropped out from a trial, with findings from 168 people in total reported across the papers.

Results

Emergent from our synthesis was the significance of trial non-retainers' perceptions around the personal 'fit' of key aspects of the trial with their personal beliefs, preferences, capabilities or life circumstances. These related to their own health state; preferences for receiving trial 'care'; individual capabilities; beliefs about or experiences of trial medication; and considerations whether trial participation could be accommodated into their broader lives. All these factors raise important issues around the extent to which initial decisions to participate were fully informed.

Conclusions

To improve retention in clinical trials, researchers should work to reduce the burden on trial participants both through the design of the intervention itself as well as through simplified data collection processes. Providing more detail on the nature of the trial interventions and what can be expected by 'participation' at the consenting stage may prove helpful in order to manage expectations.

For peer review only - http://bmjopen.bmj.com/site/about/guidelines.xhtml

Strengths and limitations of this study

- Trial retention has recently been identified as one of the top three priorities for methodological research by UK trialists.
- Within the context of clinical trials, issues around retention have not received equal scrutiny compared to methodological questions about trial recruitment despite being arguably just as important for trial validity.
- Understanding the complex reasons why trial participants leave a trial after initially consenting is important if trialists are to be able to design effective intervention strategies to address the problem.
- To our knowledge this is the first synthesis of key qualitative findings from studies exploring participants' perspectives of trial non-retention which provides learning across their collective contributions.
- Our synthesis only included 11 eligible papers reporting findings across 13 trials, 5 of which were set within a mental health context and all of which were conducted in high-income countries. This could have issues for the transferability of findings.

Introduction

Randomised controlled trials are integral for evidenced based clinical decision making. Within the context of clinical trials, the focus of much methodological research in recent years has been on issues specifically relating to trial recruitment, including significant investigation into how to increase the numbers of prospective participants recruited [1,2]. A key focus of much of this research has been on trial participants' perspectives and experiences particularly around why they do or do not choose to consent to participate in clinical trials [3,4,5,6,7,]. Whilst issues relating to trial recruitment are undoubtedly important, issues around retention (i.e. ensuring that trial participants remain in the trial to provide primary outcome data) have not received equal scrutiny in the literature despite being arguably just as important for trials in terms of ensuring that research questions are adequately answered [2].

Trial retention was recently identified in the top three priorities for methodological research by UK trialists [8]. Most trials experience the issue of missing data often referred to as a 'loss to follow-up', 'attrition' or 'drop out' and this can bias the findings of a trial. Some recent quantitative surveys have identified participant characteristics (e.g. age, gender, physical or mental health) or trial

For peer review only - http://bmjopen.bmj.com/site/about/guidelines.xhtml

processes (e.g. study duration or length and relevance of outcome measures) as being potential predictors of trial retention [9,10,11]. However, these studies are small in size, often limited to a particular clinical context, and the items included in the surveys are often identified by researchers rather than asking participants what items should be included. In addition, they lack any in depth exploration of the relevant issues affecting why participants withdraw, as reported by participants.

Understanding the complex reasons why trial participants leave a trial (either actively (e.g. by requesting no further follow up or purposefully not returning data) or passively (e.g. forgetting to return a questionnaire or attend a clinic visit)) after initially consenting to participation is important especially if those reasons are modifiable. This understanding of participant perspectives then becomes crucial if trialists are to be able to design effective intervention strategies to address the problem.

The approach of conducting in-depth qualitative research within the context of clinical trials is considered particularly useful for improving the evidence base for how trialists conduct them [12]. Indeed this approach has been used widely to explore perspectives on trial recruitment both in terms of primary qualitative studies and secondary syntheses. To our knowledge this is the first synthesis of key findings from studies exploring participants' perspectives of trial non-retention which provides learning across their collective contributions. Our aim was to undertake a meta-ethnographic synthesis of findings from such studies and our specific research question was 'what influences non-retention in clinical trials'?

Methods

A systematic literature search and meta-ethnography was conducted (See S1 ENTREQ Checklist). This meta-ethnography was undertaken in two parts. Our original systematic search and synthesis was undertaken in August 2016. To integrate potentially more recent relevant research, we undertook an update in July 2018.

Meta-ethnography essentially involves an 'interpretive and inductive' approach to synthesising studies [13,14]. Essentially meta-ethnography involves the process of 'translating' the findings of individual qualitative studies so that they can be considered in relation to one another with the aim of identifying and building new conceptual knowledge on a particular topic [13,14]. The process of 'translating' findings across studies can be either 'reciprocal' or 'refutational' depending on how individual studies relate to each other [13].

Searching and identification of relevant studies

A systematic search was conducted for published papers that contained qualitative data about trial participants' reasons for not completing some or all of the processes involved in a clinical trial after initially consenting to take part (which we describe as constituting non-retention). Search strategies were informed by previous studies [12] and are provided in Appendix 1. Seven electronic databases were searched by an information specialist: Embase, Ovid MEDLINE, PsycINFO, Cochrane Central Register of Controlled Trials, The Social Sciences Citation Index (SSCI), Cumulative Index of Nursing &

For peer review only - http://bmjopen.bmj.com/site/about/guidelines.xhtml

Allied Health Literature, and Applied Social Sciences Index and Abstracts (ASSIA) and covered papers published from 1946 to August, 2016 (first search) and from August 2016 – July 2018 (updated search). Google Scholar and bibliographies of identified publications were also searched manually for additional potentially eligible papers.

For both searches, one author screened all titles and abstracts (RN for original search; ZS for update) with a second author (KG) screening a random 10% sample. Eligible studies included those that used qualitative methods and contained qualitative data exploring any aspect of non-retention from the perspective of patient participants (recognising that non-retention might cover activities such as cessation of or withdrawal from the intervention(s), non-attendance at clinic visits, through to non-response to some or all follow up questionnaires etc).

Analysis and synthesis

In order to collate and synthesise the available primary research, the seven steps of meta-ethnography as listed in S1 Box were followed. In summary, the three authors (ZS, RN, KG) each read and systematically extracted data from the included papers, shared notes and discussed study findings and interpretations during a series of group meetings. The papers were initially organised in chronological order (but as inductive analysis progressed papers were grouped according to emerging themes) and we focused on the findings, concepts and themes used by the papers' authors generating a list of key categories. We used a standard form which summarised the main themes, information regarding methods, and any other important information relating to the context of the research within each study (some of this data is illustrated in S1 Table). Although we initially organised papers chronologically in this table, we used it to facilitate a series of further group discussions around emerging issues (See Table S2 for examples). As inductive analysis progressed we grouped and discussed our data according to the 5 key emerging themes (See S3 Table). In line with the process of undertaking a meta-ethnography, primary data or 'first order constructs' (quotations from study participants who had not completed any or some of the various trial processes) and authors' interpretations of these data ('second order constructs') were extracted, compared and contrasted between studies (enabling us to produce a 'reciprocal translation'), and organised into themes to facilitate the development of new insights or a 'line of argument' [13].

Study Quality

One author (ZS) undertook a quality assessment of each of the papers included in the synthesis. This was based on the Critical Appraisal Skills Programme (CASP) criteria [15] which was used to appraise the identified primary studies and consider their inclusion into the synthesis (See S1 CASP Checklist). Questions developed by the CASP have been used previously for appraising the quality of studies for inclusion in meta-ethnography [16,17,18,19,20].

Patient and Public Involvement

This research was done without patient involvement. Patients were not invited to comment on the study design and were not consulted to develop patient relevant outcomes or interpret the results. Patients were not invited to contribute to the writing or editing of this document for readability or accuracy.

For peer review only - http://bmjopen.bmj.com/site/about/guidelines.xhtml

Results

Description of Studies

The database search produced 1431 abstracts for the initial search and 697 abstracts for the update (see S1 Figure and S2 Figure for details). We only included studies that provided data about reasons for non-retention from the included study participants and/or in the authors' reflections. In all, 11 papers met our inclusion criteria (8 were identified from the initial search and 3 from the update). The focus and key study characteristics for the 11 included papers are outlined in S1 Table. The identified papers were conducted in 7 countries (UK, USA, Australia, Sweden, The Netherlands, Denmark and Spain) and discussed non-retention in 13 separate trials. Six of the papers focussed solely on reasons for non-retention [21,22,23,24, 25, 26], with the remaining 5 also considering reasons for consenting [27], non-consenting [28,29] and retention [30,31]. The findings in this synthesis relate to the data from non-retainers only. Each study included between 3 and 40 people who had dropped out from a trial, with findings from 168 people in total reported across the papers. As can be seen from S1 Table the setting of the trials in which the qualitative research was embedded included a range of clinical contexts such as: mental health problems [21,24,26]; mental health problems and cancer [23]; problem drinking [22, 25]; Type 1 diabetes [30]; diabetes, Chronic obstructive pulmonary disease, heart failure, or social care needs [28]; severe ankle sprains [31]; asymptomatic atherosclerosis [29]; neurodevelopment disorders [27], and osteopenia [27]. As expected, the clinical context differed as did the interventions under investigation and included: telehealth equipment or tele care devices [28]; web-based psycho-educational/cognitive therapy based support tools [21,22,24, 26]; anti-depressant medication and/or cognitive behavioural therapy [23]; exercise [25,30] various mechanical ankle supports [31]; aspirin [29]; melatonin [27]; and bisphosphonate risedronate or vitamin D analogue 1 – alphahydroxychol ecalciferol [27].

Findings were presented from trial non-retainers both before outcome data had been collected (e.g. those who withdrew from the intervention) and/or during the follow up when outcome data was being collected – in other words, papers included a mix in terms of non-retention behaviour (See S1 Table for a summary of non-retention behaviour i.e. non-adherence to intervention, non-return of questionnaires). For example, 8 studies reported aspects related to non-adherence to trial intervention: 3 of these reported cessation of trial medication [23,27 for both trials,29]; 5 reported cessation of treatment therapy sessions [21,22,23,24,26]; 1 reported cessation of use of telehealth equipment or tele health devices [28] and another reported non-completion of study workbooks [21]. Two studies reported non-return of follow-up questionnaires [22, 31]. For 2 studies, non-retention behaviour was unspecified [25,30] Three of the 11 studies appeared to have included only the views of those who had dropped out of the active intervention arms of the trial [23,24,28]. For 4 studies it was unclear whether data was from intervention or control groups [27 for both trials, 26,29,31,] and only 4 studies specifically stated that they included views of both those in the intervention and control groups [21,22,25,30].

Nine of the 11 studies used semi-structured interviews to collect data from people who had withdrawn from the main trial [21,23,24,25,26, 27,28,30 31]; 1 used a combination of focus groups and interviews[29] and another distributed a questionnaire that contained various open ended response options [23] (NB: only the qualitative data are reported and referred to in this paper). Although some papers provided gender, age and/or demographic details for participants taking part

For peer review only - http://bmjopen.bmj.com/site/about/guidelines.xhtml

in the trial in question, as can be seen from S1 Table, this information was less comprehensive for those who had dropped out of the trial. Where participant characteristic information was provided in the original studies we have included this at the end of the quotes presented to illustrate findings.

Key themes from the synthesis

Our grouping of first and second-order constructs across the 8 initially identified papers resulted in 14 sub-themes. During the process of translating themes from each of the individual studies (i.e. comparing and contrasting across studies) these sub-themes were then grouped and categorised into 5 broad key themes which characterised the main considerations and features that appeared to influence non-retention in the trials under investigation (See S2 Table). For the 3 subsequently identified papers, we repeated the various stages of meta-ethnography - in essence comparing for 'fit' and checking for any additional themes [32,33]. For the update, we attempted to follow the 'extend and renovate the house' approach [33], which involves examining the newly included studies to establish whether they add new concepts or contribute to existing ones. During this process, we were confident that concepts identified in the later 3 papers supported and complemented our originally identified 5 key themes (from the original 8 studies) with no new concepts emerging.

These themes were: 1) Perceptions of current health state in relation to specific aspects of the trial; 2) the 'fit' of aspects of the trial with individual preferences for care and support; 3) the compatibility of aspects of trial processes with individual capabilities; 4) concerns about or experiences of trial medication; and 5) considerations around the extent to which trial participation could be appropriately accommodated into individuals' broader lives.

As these theme labels suggest, within them they accommodate a spectrum of views or experiences.

The 5 broad key themes identified as influencing participants' non-retention in clinical trials are illustrated with example data in S2 Table. In S2 Table, primary study participant quotes illustrating first order constructs are displayed in italics, and primary study author interpretations illustrating second order constructs are presented in bold text. In the rest of this paper, primary study participant quotes are displayed in italics.

Influences on participant non-retention in clinical trials: a line of argument

Expressed below is our 'line of argument' which is organised into themes to facilitate the development of cumulative insights (S3 Figure conceptually illustrates the line of argument developed from the synthesis). These themes appear to be weighed up during the participant's involvement in the trial and set alongside the complex inter-relationship between self and trial process/procedures and ultimately impact on their retention in the trial. Overall, our argument emphasises the significance of trial participants' perceptions around the 'fit' of key aspects of the trial (intervention and trial processes) with their personal beliefs, preferences, capabilities or life circumstances. These factors (which were not necessarily mutually exclusive) related to beliefs about their own health state, preferences for how they wanted to receive care, their individual capabilities, beliefs about or experiences of trial medication, and also considerations around the extent to which trial participation could be appropriately accommodated (or not) into their broader lives. All of these

For peer review only - http://bmjopen.bmj.com/site/about/guidelines.xhtml

were set against the overall backdrop of the balance between their sense of self and the trial processes and procedures – this providing the overarching explanation for the influence on retention in trials Implicit within several of these identified factors is the suggestion that there may have been deficits within the initial trial consenting process which led to participants (who subsequently withdrew) not being fully informed or at least not realising what the trial expected of them and what they could expect of the trial. These findings are discussed in more detail below and arranged across 5 key themes.

1) Perceptions of current health state in relation to specific aspects of the trial.

This theme describes how aspects of the trial might not be right for people as individuals. For example, across 8 of the 11 studies a key influence on decisions to discontinue trial participation appeared to relate to perceptions of either being 'too well' to warrant further engagement with the trial [21,22,23, 25, 28,29,31] or struggling with the compatibility of aspects of the trial, particularly the interventions or ways outcomes were assessed, with their personal sense of self [21,22,25,28,29,30]. Conversely, other participants described periods of feeling too unwell to be able to engage appropriately in trial processes.

a) Being too well to engage further with trial processes

Some participants cited a belief that they had suitably recovered part way through a particular trial as a reason for discontinuing trial medication and/or problem solving treatment exercises [21,22,23, 25]. For some, this was also linked to not wanting to be reminded about health issues that they considered to be over:

"I just don't want to be reminded of the alcohol thing, because I actually think it's over" [25; Female, 30-68 years, Alcohol Use Disorder]

"Things really improved for me…I just felt really good and didn't really feel like I had that much to offer in regard to finding out more about it" [21; Female, 30-39 years, Bipolar disorder, control group]

"I have been sufficiently helped" [22; No gender/age details, Problem drinker, Intervention group]

Participants also cited recovery as a reason for not completing and returning all the required follow-up outcome assessment questionnaires [28, Severe ankle sprains] perhaps highlighting here the importance at the consenting stage of making sure participants are fully informed about the value of sustained engagement throughout the duration of the trial (even if they feel they are no longer personally benefiting from that engagement).

b) Lack of compatibility with personal sense of self

Sometimes reasoning around trial withdrawal related to participants' struggle to accommodate aspects of the trial with their personal sense of self at the time [25,28,29], suggesting that the intervention challenged their sense of self somehow. Again perhaps indicating the importance for initial trial recruitment consultations to include adequate discussions about the nature of the study intervention and also what will be expected of participants in terms of engagement with them. For example, a belief that they could self-manage or cope well enough without the need to engage with

For peer review only - http://bmjopen.bmj.com/site/about/guidelines.xhtml

the trial support intervention [28; self –care intervention to facilitate support for self-management in aging populations]; a belief that they were too overweight and unfit to participate in a group exercise intervention [25; exercise intervention for people with alcohol use disorder] a belief that they had adequately managed their condition thus far without the need for any medication [29; aspirin for asymptomatic atherosclerosis] and also non-acceptance of a diagnosis amongst those newly diagnosed [21; with bi-polar disorder; 30; with type 1 diabetes] as a reason for not relating to (or seeing any value in) the study interventions:

[Discussing the need to keep active rather than monitoring his health indoors using tele-health equipment] *"You've got [to have] the will power…if you can't do it I am finished. If I wouldn't have that I'd be, I'd be stuck inside here you know, and looking through the window like…I throw myself in the garden and everything. Everything I do I'm working on, I cook myself dinners and everything.'* [28; Male; 85yers; COPD]

"I think if it had been medication that I needed to take, I would have taken it" [29; Male; 72; stopped taking aspirin medication]

"If you're taking a lot, it knocks the hell out of your stomach…Given the choice, I'd rather not take medication full stop" [29; Male; 55; stopped taking trial medication for asymptomatic atherosclerosis]

"I wasn't ready to accept the illness. At that stage after diagnosis I wasn't willing to change my life according to the program." [21; Male; 18-29 yrs; Bipolar disorder, control group]

"Don't think it kind of really sank in as to what I'd been diagnosed with … It had kind of hit me and I wasn't really dealing with having it … [30; Female, 19-55yrs; Type 1 diabetes]

If trial participants believed that the trial did not fit with their personal sense of self this was also linked to an emotional response. For example, feelings of guilt and shame that they were too overweight and unfit to participate in a group exercise intervention [25; exercise intervention for people with alcohol use disorder].

c) *Being 'too ill' to be able to engage appropriately with trial interventions*

Conversely, within all of the papers focussing on interventions for mental health conditions, and in 1 paper focussing on people newly diagnosed with Type 1 diabetes, participants described being 'too ill' to be able to engage appropriately in trial processes [21,23,24, 26,30]. Reasons discussed in this context related to feeling either too fragile, depressed, too manic, or too emotional/stressed at certain times to be able to complete the required intervention tasks (e.g. e-health intervention and associated workbook activities; cognitive behavioural therapy; taking blood samples) and also a concern that engagement with the intervention could act as a 'trigger' in terms of exacerbating anxiety symptoms:

"I was feeling that the therapy wasn't going to help me with my problems. I thought it could lead me to be even more anxious and that it wasn't going to be beneficial for me. So, I felt that I was going to waste my time if I continued" [26; no gender details, 21-59 yrs, people with a range of serious mental health problems]

For peer review only - http://bmjopen.bmj.com/site/about/guidelines.xhtml

"I did not cope with the exercises. I did them at the start but it gradually became more difficult to complete them…..particularly the breathing exercises. I got a bit dizzy and it increased my feelings of anxiety" [24, no gender or age details, generalised anxiety disorder]

"The biggest problem I have with my bipolar disorder is consistency; when I'm down I can't even brush my teeth or get up in the morning. So doing an education program with workbooks was beyond me" [21, Female, 18-29 yrs, Bipolar disorder, BEP group]

"I often go walking when having highs because I have to keep moving, so I didn't want to sit at a computer" [21, Male, 40-49 yrs, Bipolar disorder, BEP+IS group]

As with the earlier sub-themes in this section, emotional influences were also woven through this perception of being 'too ill' to engage with the trial. One study pointed to the 'emotional impact of the cancer diagnosis' as being an influential factor linked to participant drop out [23].

2) The 'fit' of aspects of the trial with individual preferences for care and support

Across 8 of the 11 studies another important influence in decisions to discontinue trial participation appeared to relate to the fit of aspects of the intervention with preferences for how participants wanted to receive care and support [21, 22,23,24,25,26,28,31], implicitly suggesting that the initial trial consenting process may have been sub-optimal in key ways. Participants in these trials discussed how aspects of the design of the interventions were not individualised or tailored enough to be helpful and others commented on interventions being either too technical, too physically demanding, too intensive or conversely too basic:

"I needed a therapy that could better address what I felt. It didn't give me a specific answer to my worries". [26, no gender details, 21-59 yrs, people with a range of serious mental health problems]

"I would have liked to have more of a personal contact, it became a little distant everything, to do on the internet, because it is so heavy stuff, it's nice to meet a real person when you're working with heavy things like this" [24, no gender or age details, generalised anxiety disorder]

"I wanted something more about me specifically, as opposed to talking about general issues" [21, Male, 40-49 yrs, Bipolar disorder, BEP group]

"The information in the modules was too general and too limited" [21, Male, 18-29 yrs, Bipolar disorder, BEP group]

Some other participants simply indicated that they had been unhappy or dissatisfied or "not comfortable" with the treatment they had received although specific reasons were not provided within the included studies [22,23,31].

3) The compatibility of aspects of trial processes with individual capabilities

Across 3 of the 11 studies [23,24,31] the extent to which aspects of the interventions were deemed to be appropriately 'pitched' at the individual emerged as being of importance. For example, participants cited attention problems and limited reading and writing skills as a reason for withdrawing from internet delivered cognitive behavioural therapy [24] or as a reason for non-

For peer review only - http://bmjopen.bmj.com/site/about/guidelines.xhtml

response to follow-up questionnaires [31], with participants in one of these studies staing that they felt unintelligent because of their inability to understand [24].Communication and cultural issues were also cited as reasons for the discontinuation of problem solving treatments [23], suggesting that these issues would benefit from greater consideration and discussion at the consenting stage:

"I thought that it was too much to read, and I cannot read anything at all that I need to remember or learn. It goes in here and out there [pointing at the ears]" [24, no gender or age details, generalised anxiety disorder]

4) Concerns about or experiences of the trial medication

Across 2 of the 8 studies which were set within trials testing drug interventions, [27 – 2 trials; 29] concerns about the study medication were cited as reasons for discontinuing with trial participation. These included concerns that the trial drug(s) were not properly tested/licensed [27], concerns that the trial medication could negatively interact with other prescribed medication [29], through to citing a dislike of taking too much medication [29] or that the trial medication tasted offensive [27]. Constructs within this key theme again suggest potential issues with the informed consent process and highlight the importance of discussions about the purpose of any trial, the nature of trial medications and also the implication for participation of having certain co-morbidities, linking back in to the complex inter-relationship between self and trial process/procedures:

"It just scared me when it said not to be given to children under 20…I didn't understand they weren't licensed for children…and that's what I thought it was, just to see if it worked, not to actually like so then it could be licensed" [27; Mother of child in trial for young people aged 4-18yrs with rheumatic diseases]

"again I found that I had stomach problems with the tablet so I assumed that it must be the aspirin…[29, Female, 63 yrs, stopped taking trial medication for asymptomatic atherosclerosis]

"..and they discovered I had heart fibrillation…After that I'd to go on warfarin you see, so that's why I had to drop out because warfarin and aspirin just don't agree" [29, Female, 77 yrs, stopped taking trial medication for asymptomatic atherosclerosis]

"I didn't think I really wanted to go on at the start but mum and dad persuaded me to. And so…when I was getting really fed up I just said 'No I don't want to' because I didn't like the taste [of the medicine] [27; POP trial; young person 11-14 yrs]

5) Considerations around the extent to which trial participation could be appropriately accommodated into their broader lives

Aside from issues relating to beliefs about current health state, individual capabilities, preferences for care and concerns about side effects, participants also discussed how decisions to discontinue with trial participation related to other life 'events' that tended to take priority over or made it hard for them to engage fully with the various demands of the trial [21, 22,23,24,25,26,30,31]. These factors appeared less directly related to the nature of the trial interventions themselves and more about the challenges of life in general (with one study [31] suggesting that these people could be

For peer review only - http://bmjopen.bmj.com/site/about/guidelines.xhtml

classed more as 'happy' rather than 'unhappy' non-responders, in the sense that non-retention may be related to aspects out-with the trial itself). Reasoning here involved trading off trial participation with competing priorities and ranged from events such as work or family, moving to another country, exams, pregnancies, postal strikes etc and more generally simply daily routines that got in the way. Within one study [25], the importance of existing social networks was highlighted, with some participants citing a lack of support from family members as a reason for discontinuing trial participation. Within this theme participants also sometimes cited 'laziness' or 'forgetfulness' as reasons for why they had either not completed trial interventions or had not responded to follow-up questionnaires with some apparently being unaware that they were being considered as 'drop-outs' by study researchers:

[discussing cessation of therapy sessions/non-completion of study workbooks]*"I didn't have the time, and with everything else, it wasn't a priority"* [21, Female, 18-29 yrs, Bipolar disorder, control group]

[describing why they did not return a follow-up questionnaire] *"Do you know what…laziness I'm just gonna put it down to that"*

Researcher: *"OK and em it wasn't because you were disgruntled about part of the project?"*

"Definitely not no" [31, no gender/age details given, severe ankle sprains)

[discussing cessation of problem solving treatment sessions]"Did I drop out? No, I didn't dropout. I became busy and I figured I started missing calls." [23, Female, no age details, Cancer and depression)

Discussion

Principal findings

Our meta-ethnographic synthesis sought to explore factors that influence non-retention within clinical trial contexts. We identified 11 studies (reporting qualitative data from 13 trials) that explored participant reported reasons for not completing any or some of the various trial processes (after initially consenting to take part). What emerged from our analysis was the importance of trial participants' perceptions about the personal compatibility of key aspects of the trial with their personal beliefs, preferences, capabilities or life circumstances. These factors related to their own health state, preferences for how they wanted to receive care, their individual capabilities, beliefs about or experiences of trial medication, and also considerations around the extent to which trial participation could be appropriately accommodated or not into their broader lives (Conceptually illustrated in S3 Figure). Our synthesis has also highlighted that people's reasoning around dropping out of a trial can be described as being more or less 'active' in nature, with some people in our synthesis not even realising that they were being considered by the researchers as trial 'drop outs' [23]. All these factors raise important issues around the extent to which initial decisions to participate were fully informed and illustrate the importance for trial recruiters of ensuring that prospective participants are made aware of what the trial will entail and also what will be expected of them in terms of full participation.

For peer review only - http://bmjopen.bmj.com/site/about/guidelines.xhtml

Quantitative surveys have tended to investigate non-retention in the context of non-response to follow up questionnaires. These studies have identified either participant characteristics or trial processes as being potential predictors of trial retention [9,10,11].Whilst these studies have a place, it is arguably difficult to influence some of these previously identified factors influencing retention as they may not be modifiable e.g. age or study duration. Our synthesis of more in depth qualitative data has usefully built on these findings and has enabled a more nuanced understanding of key issues of relevance (which are potentially modifiable) relating to non-adherence to interventions and non-return of follow-up questionnaires. Participant characteristics as well as trial processes are of importance but we have also demonstrated that there can be a complex inter-relationship between the two. For example, a perception that the nature of the intervention negatively affects one's mental health can be of importance as can perceptions about the nature of the intervention in relation to perceptions of self or in relation to personal preferences for care and support. Furthermore, the compatibility or otherwise of various trial processes with individual capabilities can have implications for retention. Reasons given for not completing various trial processes were not necessarily mutually exclusive, but were rather a synergistic combination of factors that could apparently work towards trial non-retention. Our findings also highlight that some participants' behaviour around leaving a trial could be described as being more or less 'active' in nature (e.g. stopping trial medication because of a concern around side effects (active) versus simply not remembering or being too busy to return a questionnaire (passive)). This is an important finding and one that has not been given due consideration in previous literature to date. People's views and life situations can change over time, all having the potential to impact on their retention within a trial. Furthermore, different types of trials are likely to present particular challenges in terms of their potential for non-retention. It could also be that certain types of reasoning might be more or less modifiable and easier to address particularly if they can be anticipated upfront during the trial design stage.

A recent study exploring reasons why people declined trial participation at the consent to recruitment stage has found that most declined at the outset because they judged themselves ineligible or not in need of the specific trial therapy in question [34]. The study authors suggest that to improve recruitment to trials the most successful interventions are likely to be the ones that focus on patients' assessments of their own eligibility and their potential to benefit from the trial treatment, rather than reducing trial burden per se. In our synthesis we found that perceptions around eligibility and assessments regarding potential to benefit from the trial treatment were also considerations for people who had initially decided to join but who had subsequently ceased to engage. For example, this included those who felt that they had recovered such they did not need to engage further [21,22,23,25] and those who felt they could manage sufficiently well without engaging with the intervention [21,28,29]. However, in the context of non-retention, it is worth considering issues around trial burden (e.g. interventions that might be perceived to be too technical or too demanding given a person's health state) as well as issues around preference for particular styles of care and support and acknowledging that the specific intervention and, or, the ways outcomes are assessed has to be compatible within the context of trial participants' broader lives. In other words, issues around reducing trial burden is of importance, both in terms of the intervention itself and also the ways that follow up data is collected.

We know from previous syntheses of qualitative studies focusing on trial recruitment that people often choose to enter into trials in the hope of gaining some help for themselves from the

For peer review only - http://bmjopen.bmj.com/site/about/guidelines.xhtml

intervention (even if they also state they are doing so for altruistic reasons – i.e. to benefit research more generally), so called 'conditional altruism' [7]. Some participants in our synthesis described perceptions around feeling too ill to continue taking part or feeling suitably better such that trial engagement was no longer warranted [21,22,23,24,25]. This perception of improvement in health would appear to resonate with the concept of conditional altruism in the sense that people might cease participation if they perceive their condition improves or conversely deteriorates, such that in effect their benefit for self has been realised and their continued participation is no longer warranted. Our finding here is perhaps exaggerated in trials with a mental health context (which applied to 6 of the 13 included trials), where diagnoses can adversely affect people's ability and inclination to initially take part in research [34,35,36]. We have shown that this issue also has relevance for retention in such trials as people's health states can be particularly vulnerable to fluctuation [34]. A recent meta-synthesis of factors affecting recruitment to depression trials [37] indicated that decisions can depend on issues relating to: perceptions of health at the time of invite; attitudes towards the research and trial interventions; and the demands of the trial. Our synthesis has shown that some of this reasoning might also have the potential to impact on non-retention in those who are successfully recruited. Furthermore, previous research has suggested that the therapeutic alliance can have an impact on adherence to treatment [38]. Within the papers included in our synthesis, this was not something that was discussed per se. However, as one of our key themes illustrate, some decisions to discontinue trial participation appeared to relate to the fit of aspects of the intervention with preferences for how participants wanted to receive care and support. Within this, some trial non-retainers stated that they had wanted more face-to-face personal contact with for example, a therapist. This comparable finding could suggest that the underlying beliefs, preferences and expectations about trial participation are not explored and unpacked fully during trial consenting discussions.

Strengths and limitations

We recognise that different review teams may interpret qualitative data in slightly different ways due to pre-existing world views or expertise across research areas. However, a strength of undertaking a meta-ethnographic synthesis of findings from studies providing qualitative data on factors influencing non-retention within clinical trials is that it has allowed us to gain important new shared insights into factors that seem to affect retention across a range of trial contexts - to our knowledge this is the first study to have synthesised these primary studies in this way. Through synthesising, we have been able to pull insights from across studies, providing learning from their collective contributions. However, our systematic search identified only 11 eligible papers reporting findings across 13 trials, 5 of which had a mental health context and all of which were conducted in high-income countries. This in part perhaps reflects the difficulties researchers face in gaining access to the views of those who disengage with research. Furthermore, unlike for example surgical trials, all the included papers incorporated within their trials, interventions that participants could choose to discontinue engaging with (e.g. taking drugs; stopping CBT etc). Whilst qualitative research does not usually intend to be generalizable, it is nevertheless important to consider the transferability of our findings to other clinical trial contexts and settings and one could argue that participants within e.g. mental health trials, surgical trials, or trials that involve surrogate/proxy consent including those involving children [27] might face very different issues and challenges regarding retention. Although we were reassured that the key themes we identified had resonance across the included papers to a greater or lesser extent and so are likely to be important considerations within a range of clinical

For peer review only - http://bmjopen.bmj.com/site/about/guidelines.xhtml

trial contexts, some influences on trial non-retention are likely to be more trial specific than others (e.g. concerns about trial medication).

We carried out a quality assessment of the 11 included papers (see S1 CASP Checklist). Although all papers had study aims that were amenable to investigation via qualitative means and all included qualitative data, some were deemed richer than others in terms of data and insights (i.e. first and second order constructs). Arguably, this made undertaking a meta-ethnography in this context quite challenging as the number of studies and volume and/or quality of available data can affect depth of analysis. For example, 1 paper only reported qualitative data from open ended questionnaire response options [22], and 2 were deemed less useful in terms of presenting only very limited qualitative data (both first and second order constructs) (22,23)). Nevertheless, we did feel that they provided some helpful insights that usefully built on the findings of the other papers. Furthermore, despite some variation in the overall level of quality, due to the small number of included studies we felt it was more important to retain any relevant findings rather than disregard based on study quality. In doing so, we would argue that all 11 papers contributed useful elements to the collective whole and enabled us to develop our line of argument in terms of the issues of importance regarding trial non-retention.

<u>Practice Implications</u>

The way in which a trial is presented to individuals needs to take account of the influencing factors we have identified in this synthesis. Whilst not all the factors we identified are modifiable there influence needs to be recognised. We would argue that trialists need to think carefully about how the design of their trial might contribute to non-retention and that there is potential to modify trial design to improve retention.

To improve retention in clinical trials, researchers should work to reduce the burden on trial participants both through the design of the intervention itself as well as through simplified data collection processes. Providing more detail on the nature of the trial interventions and what can be expected by 'participation' (i.e. when and how data will be collected) at the consenting stage may prove helpful in order to manage expectations.

Some people in our synthesis appeared to be unaware that they were being considered as trial non-retainers by the study researchers. This raises the question of participants' understanding of the importance of remaining in a trial for its duration (i.e. completing the intervention and the outcome assessments) and its implications for the study in question. This finding is supported by arecent study of patient information documentation from UK NIHR funded trials that has highlighted that withdrawal and retention are poorly described and that statements about the value of retention are infrequent [39]. If trialists want to improve retention to clinical trials then there is an argument for giving the importance of completing the trial more prominence in patient information materials (and also during any trial recruitment discussions).

Our synthesis also potentially highlights the issue of people's awareness or lack thereof of what the trial interventions would entail. If trialists want to improve retention then this suggests an argument for also providing more detail on the nature of the trial interventions at the consenting stage in

For peer review only - http://bmjopen.bmj.com/site/about/guidelines.xhtml

order to manage expectations. We know from previous literature that patient/public involvement at the front end of trial design tends to be extremely limited if indeed it happens at all [40,41]. Given some of the key factors we found as being influential for non-retention, one could speculate that some early and meaningful patient/public involvement would be particularly useful (e.g. for ensuring that aspects of the trial are user-friendly and as compatible as possible with the target population's likely preferences and capabilities).

Implications for Research

A Cochrane review investigating interventions to improve retention in trials has highlighted that most strategies to improve retention have focussed on trying to improve follow-up questionnaire response [42]. Of these interventions, only monetary incentives have been shown to have a significant effect on return of questionnaires and the review highlighted that very few studies included trial participants in their design or development [42]. Our synthesis has demonstrated that there may be a range of issues relevant to trial participants that influence non-retention which may not be amenable to modification by 'incentives' or other interventions that fail to consider participants during development.

As mentioned previously, qualitative methods to improve recruitment to trials is now recognised as a well-established methodology built into the design and delivery of large publically funded clinical trials. The Qunitet Recruitment Intervention (QRI) is gathering momentum across a range of trials and Clinical Trials Units as a mechanism to unpack many of the nuances around how participants are recruited to RCTs [43]. Many of the approaches in the QRI are directly transferable to questions about retention. For example, how it is discussed in consultations and trial paperwork, what do stakeholders (trial participants and trial staff) report as the barriers and facilitators to retention, and work in this area could prove fruitful for minimising non-retention in ongoing RCTs. However, despite there being a clear need for more research in the context of trial retention, we also recognise the inherent challenges for researchers in obtaining the necessary ethical approvals for this type of research (particularly as current recruitment materials for trial participants tend to emphasise prospective participants' right to withdraw without given any reasons etc). Therefore, development of shareable resources to facilitate regulatory approvals may be an important contribution for the trials methodology community.

Finally, given that synthesis was based on a sparse data set, with 5 of the 11 included studies focused on qualitative research within mental health trials, there is certainly scope for more good quality, rigorous primary studies exploring the barriers and enablers to trial retention from a participant's perspective across a range of clinical specialties and trial design types. Interestingly, our search did not identify any studies that had explored reasons for trial participants' non-attendance at trial follow-up visits. Ideally, future studies should consider and explore all aspects of trial process relevant for retention, including completion and return of data (and its mode of delivery or collection), and attendance at follow-up visits. A recent prioritisation exercise for research into trial retention has now identified the Top 10 unanswered questions for trial retention [44]. Many of these Top 10 questions lend themselves well to enquiry by qualitative research methods and priorities should be focussed here.

For peer review only - http://bmjopen.bmj.com/site/about/guidelines.xhtml

Conclusions

Our systematic literature search and synthesis has highlighted that there is very little published qualitative literature exploring participant reported reasons for non-retention in clinical trials. Researchers have already called for ‘a science of recruitment’ in recognition that recruiting for science (e.g. trials) is not currently underpinned by an evidence base around the factors which might have the potential to impact on recruitment [1]. This is undoubtedly important but we would also argue that we need to develop a parallel focus on ‘a science of retention’ if we are to start to be able to tackle the very real issue of non-retention in clinical trials. Our qualitative synthesis (of albeit a small set of studies) feeds into this relatively undeveloped science and has shed some important light on the factors that might influence non-retention in clinical trials- factors that have implications both for practice and for further research. Taken together, the findings presented here and the subsequent implications for practice and research highlight the critical need to plan for retention as much as for recruitment during trial design and not treat it like the overlooked trial conduct ‘Cinderella’.

Supporting Information

S1 ENTREQ Checklist

S1 Appendix. Database search strategies

S1 Box Steps of Meta-ethnography

S1 Figure. PRISMA flow diagram

S2 Figure. PRISMA flow diagram – updated search

S1 Table. Characteristics of included studies

S2 Table. Key themes of influence on decisions to withdraw from trial participation

S3 Table. Spread of themes across included studies

S3 Figure. Conceptual diagram

S1 CASP Checklist

Acknowledgements

We would like to thank Cynthia Fraser for her assistance in helping develop the database searching strategies.

Contributors

For peer review only - http://bmjopen.bmj.com/site/about/guidelines.xhtml

For peer review only

KG conceived the study idea. RN screened all titles and abstracts in initial search; ZS screened all titles and abstracts in updated search with KG screening a random 10% sample from both searches. ZCS, RN and KG conducted the data analysis and ZCS wrote the initial and subsequent manuscript drafts. All the authors contributed critically to discussions about interpretation of data and revisions of manuscript drafts. All the authors approved the final version.

Funding

ZCS was supported by a core grant from the CSO (reference CZU/3/3) and a Wellcome Trust Institutional Strategic Support Fund award (reference RG12724-18). RN was supported by a Wellcome Trust Institutional Strategic Support Fund award (reference RG12724-18). KG was supported by an MRC Methodology Research Fellowship (MR/L01193X/1).

Data sharing statement

This is a review of published studies which are available to access through the relevant journals.

Competing interests statement

There are no competing interests for any author.

References

1. Bower P, Wallace P, Ward E, Graffy J, Miller J, Delaney B, Kinmonth AL. Improving recruitment to health research in primary care. Fam Pract. 2009;26(5):391–7.
2. Adamson J, Hewitt CE, Torgerson DJ. Producing better evidence on how to improve randomised controlled trials. BMJ. 2015;351:h4923.
3. Blanch DC, Rudd RE, Wright E, et al. Predictors of refusal during a multi-step recruitment process for a randomized controlled trial of arthritis education. Patient Educ Couns 2008;73:280–5.
4. Madsen SM, Holm S, Davidsen B, et al. Ethical aspects of clinical trials: the attitudes of participants in two non-cancer trials. J Intern Med. 2000;248:463–74.
5. Verheggen FW, Nieman F, Jonkers R. Determinants of patient participation in clinical studies requiring informed consent: why patients enter a clinical trial. Patient Educ Couns 1998;35:111–25.
6. Locock L, Smith L. Personal benefit, or benefiting others? Deciding whether to take part in clinical trials. Clin Trials 2011;8:85–93.
7. McCann SK, Campbell MK, Entwistle VA. Reasons for participating in randomised controlled trials: conditional altruism and considerations for self. Trials 2010;11:31.
8. Smith CT, Hickey H, Clarke M, Blazeby J, Williamson P. The trials methodological research agenda: results from a priority setting exercise. Trials. 2014;15(1):32.
9. Applebaum AJ, Lichtenthal WG, Pessin HA, et al. Factors associated with attrition from a randomized controlled trial of meaning-centered group psychotherapy for patients with advanced cancer. Psycho-Oncology. 2012; Nov;21(11):1195-204. doi: 10.1002/pon.2013. Epub 2011 Jul 12.
10. McCambridge J, Kalaitzaki E, White IR, Khadjesari Z, Murray E, Linke S et al. Impact of length or relevance of questionnaires on attrition in online trials: randomized controlled

For peer review only - http://bmjopen.bmj.com/site/about/guidelines.xhtml

trial. Journal of Medical Internet Research. 2011 Oct;13(4):e96. e96. Available from, DOI: 10.2196/jmir.1733.

11. Hui D, Glitza I, Chisholm G et al. Attrition rates, reasons, and predictive factors in supportive care and palliative oncology clinical trials. Cancer. 2013:119;5:1098-1105.
12. O'Cathain A, Thomas K, Drabble Set al. The QUAlitative Research in Trials (QUART) study. Health Technology Assessment. 2014;18: No. 38 DOI: 10.3310/hta18380.
13. Noblit GW, Hare RD. Meta-ethnography: synthesizing qualitative studies. 1988. Newbury Park: Sage.
14. Britten N, Campbell R, Pope C et al. Using meta ethnography to synthesise qualitative research: a worked example. J Health Serv Res Policy. 2002;7:4:209–215.
15. Critical Appraisal Skills Programme (2017). CASP (Qualitative Research) Checklist. [online] Available at: http://docs.wixstatic.com/ugd/dded87_25658615020e427da194a325e7773d42.pdf Accessed: July 2017.
16. [illegible]ell [illegible] Pound [illegible], P[illegible] C et a[illegible] [illegible]valuating [illegible]eta-ethnography[illegible] a synthes[illegible] of qualitative [illegible]
17. Pound P, Britten N, Morgan M, et al. Resisting medicines: a synthesis of qualitative studies of medicine taking. Soc Sci Med. 2005. 61: 133-155. 10.1016/j.socscimed.2004.11.063.
18. Atkins S, Lewin S, Smith H, et al. Conducting a meta-ethnography of qualitative literature: Lessons learnt. BMC Medical Research Methodology. 2008, 8:21 doi:10.1186/1471-2288-8-21.
19. Schumm K, Skea Z, McKee L, N'Dow J. 'They're doing surgery on two people': a meta-ethnography of the influences on couples' treatment decision making for prostate cancer. Health Expectations. 2010:13, pp.335–349.
20. Black M, Entwistle VA, Bhattacharya S, et al. Vaginal birth after caesarean section: why is uptake so low? Insights from a meta-ethnographic synthesis of women's accounts of their birth choices. BMJ Open 2016;6: e008881. doi:10.1136/ bmjopen-2015-008881.
21. Nicholas J, Proudfoot J, Parker G, et al. "The Ins and Outs of an Online Bipolar Education Program: A Study of Program Attrition."J Med Internet Res 2010 (Dec 19); 12(5):e57.
22. Postel M, de Haan H, ter Huurne E, et al. Effectiveness of a Web-based Intervention for Problem Drinkers and Reasons for Dropout: Randomized Controlled Trial. J Med Internet Res 2010;12(4):e68.
23. Wells A, Palinkas L, Qiu X, et al. Cancer patients' perspectives on discontinuing depression treatment: the "drop out" phenomenon. Patient preference and Adherence. 2011:5;465-470.
24. Johansson O, Michel, T, Andersson G, et al. Experiences of non-adherence to Internet-delivered cognitive behavior therapy: A qualitative study. Internet Interventions. 2015:2;137-142.
25. Sari S, Muller AE, Roessler KK. Exercising alcohol patients don't lack motivation but struggle with structures, emotions and social context – a qualitative dropout study. BMC Family Practice 2017:18;45.
26. Fernandez-Alvarez J, Diaz-Garcia A, Gonzalez-Robles A et al. Dropping out of a transdiagnostic online intervention: A qualitative analysis of clients' experiences. Internet Interventions. 2017:10; 29-38.

For peer review only - http://bmjopen.bmj.com/site/about/guidelines.xhtml

27. Shilling, V, Williamson, P R, Hickey, H, Sowden, E, Smyth, R L and Young, B (2011) Processes in recruitment to randomised controlled trials of medicines for children (RECRUIT): a qualitative study. Project Report. 2011: 15. NIHR Journals Library.
28. Sanders C, Rogers A, Bowen R, et al. Exploring barriers to participation and adoption of telehealth and telecare within the Whole System Demonstrator trial: a qualitative study. BMC Health Services Research. 2012: **12**:220 https://doi.org/10.1186/1472-6963-12-220.
29. Eborall H, Stewart M, Cunningham-Burley S, et al. et al. Accrual and drop out in a primary prevention randomised controlled trial: qualitative study.Trials. 2011: 12:7 http://www.trialsjournal.com/content/12/1/7.
30. Henshall C, Narendran P, Andrews RC et al. Qualitative study of barriers to clinical trial retention in adults with type 1 diabetes. BMJ Open. 2018;8:e022353.
31. Nakash R, Hutton J, Lamb S, et al. Response and non-response to postal questionnaire follow-up in a clinical trial – a qualitative study of the patient's perspective. Journal of Evaluation in Clinical Practice. 2008:14;226-235.
32. Lang H, France E, Williams B et al. The psychological experience of living with head and neck cancer: a systematic review and synthesis. Psychooncology. 2013:22;12:2648-63.
33. France E, Wells M, Lang H et al. Why, When and how to update a meta-ethnography qualitative synthesis. Systematic Reviews. 2016:5:44.
34. Hughes-Morley A, Young B, Hempel R, Russell I, Waheed W, Bower P. What can we learn from trial decliners about improving recruitment? Qualitative study *Trials.*2016;**17**:494.
35. Halpern SD, Karlawish JHT, Berlin JA. The continuing unethical conduct of underpowered clinical trials. JAMA. 2002;288:358–62.
36. Drueke TB, Descamps-Latscha B, Locatelli F. Stopping a medical research project for financial reasons. Nephrol Dial Transplant. 2003;18(10):1982–3.
37. Hughes-Morley A, Young B, Waheed W, Small N, Bower P. Factors affecting recruitment into depression trials: systematic review, meta-synthesis and conceptual framework. J Affect Disord. 2015;172C:274–90.
38. Thompson L, McCabe R. The effect of clinician-patient alliance and communication on treatment adherence in mental health care: a systematic review. BMC Psychiatry. 2012;12:87. doi:10.1186/1471-244X-12-87.
39. Kearney A, Rosala-Hallas A, Bacon N, et al. Reducing attrition: the communication of retention and withdrawal within patient information sheets. Trials. 2017:18;(Suppl 1):P.489.
40. Gamble C, Dudley L, Allam A, et al. Patient and public involvement in the early stages of clinical trial development: a systematic cohort investigation. BMJ Open 2014;4:e005234. doi:10.1136/bmjopen-2014- 005234.
41. Buck D, Gamble C, Dudley L, et al. From plans to actions in patient and public involvement: qualitative study of documented plans and the accounts of researchers and patients sampled from a cohort of clinical trials. BMJ Open 2014;4:e006400. doi:10.1136/bmjopen-2014- 006400.
42. Brueton VC, Tierney J, Stenning S, Harding S, Meredith S, Nazareth I, Rait G. Strategies to improve retention in randomised trials. Cochrane Database of Systematic Reviews 2013, Issue 12. Art. No.: MR000032. DOI: 10.1002/14651858.MR000032.pub2.
43. Donovan J, Rooshenas L, Jepson M et al. Optimising recruitment and informed consent in randomised controlled trials: the development and implementation of the Quintet Recruitment Intervention (QRI). Trials. 2016; 17:283. DOI 10.1186/s13063-016-1391-4.

Authors: Dan Brunsdon; Linda Biesty; Peter Brocklehurst;

For peer review only - http://bmjopen.bmj.com/site/about/guidelines.xhtml

44. Brunsdon D, Biesty L, Brocklehurst et al. What are the most important unanswered research questions in trial retention? A James Lind Alliance Priority Setting Partnership – The PRioRiTy II (Prioritising Retention in Randomised Trials) Study (under review)

For peer review only

Search strategies

July 2018 Update

Database: Embase <1996 to 2018 Week 30>, Ovid MEDLINE(R) and Epub Ahead of Print, In-Process & Other Non-Indexed Citations, and Daily <1946 to July 23, 2018>
Search run 24th July 2018
URL: https://ovidsp.ovid.com/

1 exp clinical trial/ (1980088)
2 randomi?ed control$ trial$.tw. (336149)
3 randomi?ed controlled trial?.kw. (23247)
4 clinical trial?.tw,kw. (729051)
5 controlled trial?.tw,kw. (429924)
6 controlled clinical trial?.tw,kw. (50908)
7 pragmatic trial?.tw,kw. (2136)
8 complex intervention?.tw,kw. (4998)
9 or/1-8 (2639576)
10 qualitative research/ (94827)
11 qualitative research.tw,kw. (34500)
12 (qualitative adj3 method$).tw. (53149)
13 (qualitative method? or qualitative methodology).kw. (2740)
14 (qualitative adj3 stud$).tw. (95856)
15 qualitative study.kw. (2597)
16 focus groups/ use ppez (24850)
17 focus group?.tw,kw. (82443)
18 grounded theory/ (6039)
19 grounded theory.tw,kw. (21174)
20 narrative analys?s.tw,kw. (2147)
21 process evaluation.tw,kw. (5815)
22 mixed method?.tw,kw. (30409)
23 mixed method$.mp. (31273)
24 mixed methodology.tw,kw. (711)
25 (in depth adj4 interview$).tw. (41493)
26 in depth interview?.kw. (201)
27 ((semi structured or semistructured) adj5 interview$).tw. (89673)
28 semi structured interview?.kw. (277)

For peer review only

29 qualitative interview$.tw. (18002)

30 qualitative interview?.kw. (432)

31 (interview$ and theme$).tw. (61425)

32 interview?.kw. (6730)

33 (interview$ and audio recorded).tw. (5252)

34 qualitative case stud$.tw. (2011)

35 descriptive case stud$.tw. (475)

36 qualitative case study.kw. (25)

37 descriptive case study.kw. (0)

38 qualitative exploration.tw,kw. (1994)

39 qualitative evaluation.tw,kw. (6296)

40 qualitative intervention.tw,kw. (25)

41 qualitative approach.tw,kw. (7887)

42 qualitative inquiry.tw,kw. (1197)

43 qualitativ$ analys$.tw. (31755)

44 qualitative analysis.kw. (1269)

45 (qualitative adj3 data).tw. (34567)

46 qualitative data.kw. (152)

47 discourse analysis.tw,kw. (3342)

48 discursive.tw,kw. (3245)

49 phenomenological.tw,kw. (29346)

50 thematic analysis.tw,kw. (27166)

51 ethnograph$.tw. (18207)

52 ethnography.kw. (1849)

53 action research.tw,kw. (7525)

54 ethno?methodology.tw,kw. (150)

55 social construction.tw,kw. (1643)

56 or/10-55 (426531)

57 phenomenological characteristics.tw,kw. (242)

58 phenomenological model.tw,kw. (1806)

59 action research arm test.tw,kw. (1065)

60 protocol.ti. (79269)

61 or/57-60 (82341)

62 56 not 61 (418963)

63 9 and 62 (25059)

64 Patient Dropout/ use ppez (7679)

65 Patient Dropouts/ use emef (433)

For peer review only

For peer review only - http://bmjopen.bmj.com/site/about/guidelines.xhtml

66 Patient Recruitment/ use ppez (59391)

67 Research Subjects/ use emef (4918)

68 patient recruitment.kw. (179)

69 attrition.kw. (1451)

70 patient retention.kw. (32)

71 ((recruit$ or participat$ or take part or dropout$ or drop$ out$ or withdr?wl$ or barrier$ or retention or response$ or respond$ or attrition) adj10 trial?).tw. (134886)

72 or/64-71 (204550)

73 63 and 72 (3670)

74 limit 73 to english language (3640)

75 74 not abstract.pt. (3020)

76 exp animals/ not human/ (7165523)

77 exp nonhuman/ not humans/ (3518640)

78 75 not (76 or 77) (3011)

79 limit 78 to yr="2010 -Current" (2280)

80 remove duplicates from 79 (1325)

****

Database: PsycINFO <2002 to July Week 3 2018>

Search run 24th July 2018

URL: https://ovidsp.ovid.com/

1 clinical trials/ (10606)

2 randomi?ed control$ trial$.tw. (26600)

3 clinical trial?.tw. (27527)

4 controlled trial?.tw. (31463)

5 controlled clinical trial?.tw. (2319)

6 pragmatic trial?.tw. (191)

7 complex intervention?.tw. (624)

8 or/1-7 (58183)

9 qualitative research/ (7513)

10 qualitative research.tw. (17348)

11 (qualitative adj3 method$).tw. (21172)

12 (qualitative adj3 stud$).tw. (46815)

13 focus group?.tw. (27999)

14 grounded theory/ (3229)

15 grounded theory.tw. (12698)

For peer review only - http://bmjopen.bmj.com/site/about/guidelines.xhtml

16 narrative analys?s.tw. (1966)
17 process evaluation.tw. (1167)
18 mixed method?.tw. (18416)
19 mixed methodology.tw. (731)
20 (in depth adj4 interview$).tw. (21003)
21 ((semi structured or semistructured) adj5 interview$).tw. (34163)
22 qualitative interview$.tw. (7708)
23 (interview$ and theme$).tw. (33368)
24 interview?.kw. (0)
25 (interview$ and audio recorded).tw. (1253)
26 qualitative case stud$.tw. (4333)
27 descriptive case stud$.tw. (565)
28 qualitative exploration.tw. (944)
29 qualitative evaluation.tw. (751)
30 qualitative intervention.tw. (9)
31 qualitative approach.tw. (3312)
32 qualitative inquiry.tw. (1457)
33 qualitativ$ analys$.tw. (10100)
34 (qualitative adj3 data).tw. (17288)
35 discourse analysis/ (6373)
36 discursive.tw,kw. (7705)
37 phenomenological.tw. (20981)
38 thematic analysis.tw. (9031)
39 ethnograph$.tw. (21222)
40 action research.tw. (6521)
41 ethno?methodology.tw. (369)
42 social construction.tw. (2816)
43 or/9-42 (211086)
44 phenomenological characteristics.tw. (129)
45 phenomenological model.tw. (123)
46 action research arm test.tw. (110)
47 protocol.ti. (2319)
48 or/44-47 (2678)
49 43 not 48 (210507)
50 experimental attrition/ (307)
51 experimental recruitment/ (96)
52 experimental subjects/ (2407)

For peer review only

For peer review only - http://bmjopen.bmj.com/site/about/guidelines.xhtml

53 dropouts/ (348)

54 ((recruit$ or participat$ or take part or dropout$ or drop$ out$ or withdr?wl$ or barrier$ or retention or response$ or respond$ or attrition) adj10 trial?).tw. (13610)

55 or/50-54 (16312)

56 8 and 49 and 55 (484)

57 exp animals/ not human/ (179236)

58 56 not 57 (484)

59 limit 58 to (english language and yr="2010 -Current") (339)

****

Cochrane Central Register of Controlled Trials : Issue 7, 2018

Search run 25th July 2018

URL: http://www.cochranelibrary.com/

#1 MeSH descriptor: [Qualitative Research] this term only

#2 qualitative NEXT research:ti,ab,kw or qualitative NEXT method:ti,ab,kw or qualitative NEXT study:ti,ab,kw (Word variations have been searched)

#3 MeSH descriptor: [Focus Groups] this term only

#4 MeSH descriptor: [Grounded Theory] this term only

#5 mixed NEXT method:ti,ab,kw or narrative NEXT analysis:ti,ab,kw (Word variations have been searched)

#6 interview:ti,ab,kw

#7 qualitative case study:ti,ab,kw or descriptive case study:ti,ab,kw (Word variations have been searched)

#8 qualitative NEXT exploration:ti,ab,kw or qualitative NEXT evaluation:ti,ab,kw or qualitative intervention:ti,ab,kw or qualitative approach:ti,ab,kw or qualitative analysis: ti,ab,kw (Word variations have been searched)

#9 qualitative data:ti,ab,kw (Word variations have been searched)

#10 discourse analysis:ti,ab,kw or discursive:ti,ab,kw (Word variations have been searched)

#11 social construction:ti,ab,kw or action research:ti,ab,kw or ethnography:ti,ab,kw or thematic analysis:ti,ab,kw or phenomenological:ti,ab,kw (Word variations have been searched)

#12 #1 or #2 or #3 or #4 or #5 or #6 or #7 or #8 or #9 or #10 or #11

#13 MeSH descriptor: [Patient Dropouts] this term only

#14 MeSH descriptor: [Patient Selection] this term only

#15 ((recruit$ or participat$ or take part or dropout$ or drop$ out$ or withdr?wl$ or barrier$ or retention or response$ or respond$ or attrition) near/10 trial?):ti,ab,kw (Word variations have been searched)

For peer review only - http://bmjopen.bmj.com/site/about/guidelines.xhtml

For peer review only

#16 #13 or #14 or #15

#17 #12 and #16 Publication Year from 2010 to 2018, in Trials

#18 abstract:pt (Word variations have been searched)

#19 #17 not #18 (385)

Social Sciences Citation Index

Search run: 24th July 2018

Web of Knowledge: URL http://wok.mimas.ac.uk/

#32 382 #21 AND #24 AND #31 Refined by: DOCUMENT TYPES: (ARTICLE OR REVIEW) Indexes=SSCI Timespan=2010-2018

31 12,293 #25 OR #26 OR #27 OR #28 OR #29 OR #30

30 1,646 (TS=patient dropout) AND LANGUAGE: (English)

29 1,311 (TS=patient attrition) AND LANGUAGE: (English)

28 2,040 (TS=patient retention) AND LANGUAGE: (English)

27 4761 (TS=((withdraw$ or barrier$ or retention or response$ or respond$ or attrition) NEAR/10 trial?)) AND LANGUAGE: (English)

26 136 (TS=(("take part" or dropout$ or "drop$ out") NEAR/10 trial?)) AND LANGUAGE: (English)

25 220 (TS=((recruit$ or participat$) NEAR/10 trial?)) AND LANGUAGE: (English)

24 42,063 #23 OR #22

23 42,671 (TS=randomised controlled trial) AND LANGUAGE: (English)

22 42,671 (TS=randomized controlled trial) AND LANGUAGE: (English)

21 148,316 #20 OR #19 OR #18 OR #17 OR #16 OR #15 OR #14 OR #13 OR #12 OR #11 OR #10 OR #9 OR #8 OR #7 OR #6 OR #5 OR #4 OR #3 OR #2 OR #1

20 7,765 (TS=thematic analysis) AND LANGUAGE: (English)

19 16,672 (TS=action research) AND LANGUAGE: (English)

18 7,436 (TS=social construction) AND LANGUAGE: (English)

17 5,371 (TS=discursive) AND LANGUAGE: (English)

1611,763 (TS=discourse analysis) AND LANGUAGE: (English)

15 6,704 (TS=(qualitative near/1 data)) AND LANGUAGE: (English)

14 8,581 (TS=(qualitative near/1 analysis)) AND LANGUAGE: (English)

13 3,122 (TS=(qualitative near/1 approach)) AND LANGUAGE: (English)

12 122 (TS=(qualitative near/1 intervention)) AND LANGUAGE: (English)

11 688 (TS=(qualitative near/1 evaluation)) AND LANGUAGE: (English)

10 529 (TS=(qualitative near/1 exploration)) AND LANGUAGE: (English)

For peer review only - http://bmjopen.bmj.com/site/about/guidelines.xhtml

9 40,423 (TS=(in depth interview* or semi structured interview* or qualitative interview*)) AND LANGUAGE: (English)

8 7,414 (TS=narrative analysis) AND LANGUAGE: (English)

7 19,879 (TS=mixed method*) AND LANGUAGE: (English)

6 8,541 (TS=grounded theory) AND LANGUAGE: (English)

5 40,298 (TS=focus group*) AND LANGUAGE: (English)

4 2,062 (TS= (qualitative NEAR/1 studies)) AND LANGUAGE: (English)

#3 15,953 ((TS= (qualitative NEAR/1 study))) AND LANGUAGE: (English)

2 8,468 ((TS= (qualitative NEAR/1 method*))) AND LANGUAGE: (English)

1 12,460 (TS= (qualitative NEAR/1 research)) AND LANGUAGE: (English)

Cumulative Index of Nursing & Allied Health Literature

Search run 25th July 2018

URL: http://search.ebscohost.com/

S18 S7 AND S16 Limiters - Published Date: 20100101-20181231 Narrow by Language: - English (278)

S17 S7 AND S16

S16 S8 OR S9 OR S10 OR S11 OR S12 OR S13 OR S14 OR S15

S15 TX discourse analysis OR TX discursive OR TX thematic analysis OR TX ethnography OR TX action research OR TX phenomenological

S14 TX qualitative exploration OR TX qualitative evaluation OR TX qualitative intervention* OR TX qualitative approach OR TX qualitative analysis OR TX qualitative data

S13 TX mixed method* OR TX semi structured interview* OR TX in depth interview*

S12 TX focus group* OR TX grounded theory OR TX narrative analysis

S11 TX qualitative n3 research OR TX qualitative n3 method* OR TX qualitative n3 study

S10 (MH "Focus Groups")

S9 (MH "Semi-Structured Interview") OR (MH "Structured Interview") OR (MH "Narratives")

S8 (MH "Qualitative Studies+")

S7 S3 AND S6

S6 S4 OR S5

S5 TX ((recruit$ or participat$ or take part or dropout$ or drop$ out$ or withdr?wl$ or barrier$ or retention or response$ or respond$ or attrition) N10 trial?)

S4 (MH "Research Subjects+")

S3 S1 OR S2

For peer review only - http://bmjopen.bmj.com/site/about/guidelines.xhtml

S2 TX ranndomized or randomised or trial*

S1 (MH "Clinical Trials+")

Applied Social Sciences Index and Abstracts

Search run 25th July 2018

http://search.proquest.com/assia/

S8 S5 and S6Limits applied Language:English PY: 2010-2018 (373)

S7 S5 and S6

S6 (recruit* N/10 trial?) OR (participat* N/10 trial? OR "take part" N/10 trial?) OR (dropout* N/10 trial? OR drop* our* N/10 trial?) OR (withdraw* N/10 trial* OR barrier* N/10 trial?) OR (retention N/10 trial? OR response* N/10 trial?) OR (respond* N/10 trial? OR attrition N/10 trial?)

S5 S3 and S4

S4 qualitative OR (focus group* OR interview*) OR (mixed method* OR ethnography) OR (phenomenological OR discourse analysis) OR discursive

S3 S1 or S2

S2 randomized OR randomised

S1 SU.EXACT("Clustor randomized trials") OR SU.EXACT("Clinical randomized controlled trials") OR SU.EXACT("Single blind randomized controlled trials") OR SU.EXACT("Cluster randomized controlled trials") OR SU.EXACT("Randomized controlled trials") OR SU.EXACT("Double blind randomized trials") OR SU.EXACT("Prospective controlled trials") OR SU.EXACT("Double blind randomized controlled trials") OR SU.EXACT("Clinical trials")

For peer review only - http://bmjopen.bmj.com/site/about/guidelines.xhtml

S1 Box. Our analytical approach

1) Getting started

This stage involved us generating a research question specific to our area of interest that we believed could be usefully addressed by referring to qualitative research. In our case this was the question of what influences non-retention within clinical trials from the perspectives of trial withdrawers?

2) Describing what is relevant to initial interest

This stage involved making a series of decisions relating to deciding what was relevant to our initial area of interest, deciding on the searching process, inclusion/exclusion decisions and quality assessment. In our case we were interested in any study that reported the use of qualitative methods (for collection and analysis of data) to explore the reasons why individual participants withdraw from clinical trials. We were interested in any reports made by participants themselves or by trial staff, but this had to be specifically in relation to why participants withdraw. We defined withdrawal or non-retention as covering any aspect of attrition recognising that this might cover activities such as cessation of, or withdrawal from the intervention(s), non-attendance at clinic visits, through to non-response to some or all follow up questionnaires etc. We decided that we would exclude studies that did not use qualitative means to collect or analyse their data and also studies reporting findings from trial withdrawers who were not patient participants e.g. GPs in a primary care cluster trial. Following these decisions, a systematic search across a range of databases was conducted with assistance from an information specialist (See S1 Appendix) and all titles and abstracts were screened for inclusion (see S1 and S2 Figures). Applying quality criteria to qualitative research remains a contentious issue and there is no consensus regarding whether and how this should be done (Mays 2000; McEwan 2004). However, one author (ZS) undertook a quality assessment of each of the 11 papers that were identified as being eligible for inclusion in the synthesis. Whilst authors of some qualitative evidence syntheses have chosen to exclude what they deem to be poor quality papers, we made the decision not to exclude any of the identified papers. Although all papers had study aims that were amenable to investigation via qualitative means and all included qualitative data, as a team we deemed some as being richer than others in terms of data and insights (i.e. first and second order constructs). Despite this variation in the overall level of quality, due to the small number of identified studies we considered it more important to retain any relevant findings than disregard based on study quality. In doing so, we would argue that all 11 papers contributed useful elements to the collective whole and enabled us to develop our line of argument in terms of the issues of importance regarding trial non-retention.

3) Reading the studies

At this stage, we aimed to become as familiar as possible with the content of all the identified papers with each author independently reading through all of the data provided and making detailed notes of their observations including identification of preliminary themes. After sharing notes, we met to discuss our findings as a team, comparing and contrasting our preliminary observations etc. We repeated this process for the 3 papers that we identified in our updated database search.

4) Determining how the studies are related

In describing this phase, Noblit and Hare 1988 state that “In doing a synthesis, the various studies must be ‘put together’. This requires determining the relationships between the studies to be

For peer review only - http://bmjopen.bmj.com/site/about/guidelines.xhtml

synthesized. We think it makes sense to create a list of the key metaphors, phrases, ideas, and/or concepts (and their relations) used in each account and to juxtapose them…" During this next stage (which in practice we found very much related to activities undertaken as part of stage 3), data was extracted initially from all 8 papers (retrieved from the 1st database search) using a standard form which summarised the main phrases, themes and ideas, along with, information regarding methods, and any other important information relating to the context of the research (some of this data is illustrated in S1 Table). During this stage, we focussed on both 1st order constructs within included papers (meaning study participant quotations found in the results section of papers) along with 2nd order constructs (meaning the interpretations made by the papers' authors, usually found in the discussion and conclusion sections of papers but also sometimes within the results). Using the standard form, the papers were initially organised in chronological order (but as inductive analysis progressed papers were grouped according to emerging themes) and we focused on the findings, concepts and themes used by the papers' authors generating a list of key categories. This document (along with our other written notes and observations) facilitated discussions at a series of subsequent team meetings and were very useful for consideration of how identified themes from one paper might relate to the others. We added similar data from the additionally identified 3 papers to this form, to allow us to compare and contrast findings with the earlier 8 papers.

5) Translating the studies into one another

Noblit and Hare 1988 state that "In its simplest form, translation involves treating the accounts as analogies: One program is like another except….It also compares both the metaphors or concepts and their interactions in one account with the metaphors and their interactions in the other accounts."

At this key stage (which again in practice we found inter-related to stage 4), following this process, we sought to consider the extent to which themes and concepts seemed common or distinct across the papers. Our initial grouping of 1st and 2nd order constructs across the 8 papers resulted in 14 sub-themes. These were issues/ideas that we each considered important in terms of things that might make people withdraw from trials. During the process of translating themes/concepts from each of the individual studies into those of the others (i.e. comparing and contrasting across studies), following further team discussion these were then grouped and categorised into 5 broad key themes (as it became apparent that some of sub-themes were related or overlapped). We interpreted our 5 key themes as characterising the main considerations and features that appeared to influence non-retention in the trials under investigation (See S2 Table). For the 3 subsequently identified papers, we repeated this stage by comparing and contrasting concepts and their interactions in these 3 accounts with the concepts identified in the original 8 accounts– in essence comparing for 'fit' and checking for any additional themes (Lang 2013). During this process, we were confident that concepts identified in the later 3 papers supported and complemented our originally identified 5 key themes with no new themes emerging.

6) Synthesizing the translations

Noblit and Hare 1988 state that "Synthesis refers to making a whole into something more than the parts alone imply." For our synthesis, what we were attempting to do at this stage was to move towards an explanatory analysis. We considered and discussed how the various translations compared in an attempt to develop a more nuanced and collective understanding of factors influencing trial non-retention (in doing so, developing our 'line of argument' synthesis). As before, we did this through a process of reflection and team discussions, in an attempt to produce overarching insights into the factors that appear to influence non-retention.

For peer review only - http://bmjopen.bmj.com/site/about/guidelines.xhtml

7) Expressing the synthesis

As is common with other meta-ethnographies we sought to express our collective insights in both textual and diagrammatic format within our paper. In doing so, we expressed our synthesis both within our paper as our 'line of argument' (with supportive illustrative data from across the studies) and also as a conceptual diagram (see S3 Figure).

For peer review only

	Nakash 2008	Nicholas 2010	Postel 2010	Eborall 2011	Wells 2011	Shilling 2011	Sanders 2012	Johannson 2015	Sari 2017	Fernandez-Alvarez 2017	Henshall 2018
Was there a clear statement of the aims of the research?	Yes	Yes	Yes	No – not explicitly in abstract or background	Yes	Yes	Yes	Yes	Yes	Yes	Yes
Is a qualitative methodology appropriate?	Yes	Yes	This reported some qualitative data from open ended questionnaire response options	Yes	Yes	Yes	Yes	Yes	Yes	Yes	Yes
Was the research design appropriate to address the aims of the research?	Yes	Yes	Unclear	Yes	Yes	Yes	Yes	Yes	Yes	Yes	Yes
Was the recruitment strategy appropriate to the aims of the research?	Yes	Yes	Yes	Yes	Yes	Yes	Yes	Yes	Yes	Yes	Yes
Was the data collected in a way that addressed the research issue?	Yes	Yes	Unclear	Yes	Yes	Yes	Yes	Yes	Yes	Yes	Yes
Has the relationship between researcher and participants been adequately considered?	Unclear – not mentioned in paper	Unclear - not mentioned in paper	Unclear	Unclear – status of interviewer mentioned but not discussed further	Unclear - not mentioned in paper	Unclear- not mentioned in paper	Unclear – not mentioned in paper	Yes, this was discussed	Yes, this was discussed	Yes, this was discussed	Yes, this was discussed

For peer review only

Have ethical issues been taken into consideration?	Yes	Yes	Yes	Yes	Unclear	Yes	Unclear	Yes	Unclear	Unclear – informed consent mentioned, but not ethical approvals	Unclear – informed consent mentioned, but not ethical approvals
Was the data analysis sufficiently rigorous?	Yes, apparently so (although not huge detail)	Yes	Unclear – not mentioned other than that the data was systematically analysed – reflects that it was not a qualitative study as such.	Yes	Unclear – very brief details and presented in quantitative manner	Yes	Yes	Yes	Yes	Yes	Yes
Is there a clear statement of findings?	Yes	Yes	Yes, but brief	Yes	Yes	Yes, but focus of report was not on reasons for withdrawing and so this was somewhat buried and limited.	Yes	Yes	Yes	Yes	Yes
How valuable is the research to our review?	Useful findings – NB: Limited 1st order construct data perhaps because paper also included data from trial retainers (and this seemed to be main emphasis)	Useful – perhaps one of the richer papers in terms of insights and data	Less useful than other studies but still provides helpful insights that help to build on the findings of other studies. Qualitative data presented was very limited (both 1st and 2nd	Useful – more 1st order constructs than some of the other papers e.g. Nakash, Sanders	Less useful than other studies but still provides helpful insights that help build on other studies. Qualitative data presented was very limited	Useful – but study focussed on decliners and withdrawers and provided limited data from the latter.	Useful – but only 3 were withdrawers, rest were decliners and so like Nakash the emphasis of the paper (and the bulk of data and reflection provided) was on decliners. Few 1st order	Useful – perhaps one of the richer papers in terms of insights and data	Useful, although limited 1st order constructs	Useful – more 1st order constructs than some of the other papers e.g. Nakash, Sanders	Useful findings – NB: Limited 1st order construct data perhaps because paper also included data from trial retainers – out of 20 participants interviewed,

For peer review only

			order constructs) and was discussed in a more quantitative way.		(both 1st and 2nd order constructs) and was discussed in a more quantitative way.		constructs from withdrawers.				only 4 had dropped out.

For peer review only

Enhancing transparency in reporting the synthesis of qualitative research: ENTREQ

ENTREQ Statement: content and rationale

The ENTREQ statement consists of 21 items grouped into five main domains: introduction, methods and methodology, literature search and selection, appraisal, and synthesis of findings (Table 1). For each item, a descriptor and examples are provided. Below we present a rationale for each domain and its associated items.

Table 1

Enhancing transparency in reporting the synthesis of qualitative research: the ENTREQ statement

No	Item	Guide and description	
**1**	Aim	State the research question the synthesis addresses.	See Page 3
**2**	Synthesis methodology	Identify the synthesis methodology or theoretical framework which underpins the synthesis, and describe the rationale for choice of methodology *(e.g. meta-ethnography, thematic synthesis, critical interpretive synthesis, grounded theory synthesis, realist synthesis, meta-aggregation, meta-study, framework synthesis).*	See Pages 3-4 and S1 Box
**3**	Approach to searching	Indicate whether the search was pre-planned (*comprehensive search strategies to seek all available studies)* or iterative (*to seek all available concepts until they theoretical saturation is achieved*).	See Page 3
**4**	Inclusion criteria	Specify the inclusion/exclusion criteria *(e.g. in terms of population, language, year limits, type of publication, study type).*	See Page 3-4
**5**	Data sources	Describe the information sources used (e.g. *electronic databases (MEDLINE, EMBASE, CINAHL, psycINFO, Econlit), grey literature databases (digital thesis, policy reports), relevant organisational websites,*	See Page 3

No	Item	Guide and description	
		experts, information specialists, generic web searches (Google Scholar) hand searching, reference lists) and when the searches conducted; provide the rationale for using the data sources.	
**6**	Electronic Search strategy	Describe the literature search *(e.g. provide electronic search strategies with population terms, clinical or health topic terms, experiential or social phenomena related terms, filters for qualitative research, and search limits)*.	See Page 3 and Appendix 1
**7**	Study screening methods	Describe the process of study screening and sifting *(e.g. title, abstract and full text review, number of independent reviewers who screened studies).*	See Page 3-4
**8**	Study characteristics	Present the characteristics of the included studies *(e.g. year of publication, country, population, number of participants, data collection, methodology, analysis, research questions).*	See Page 4-5
**9**	Study selection results	Identify the number of studies screened and provide reasons for study exclusion *(e,g, for comprehensive searching, provide numbers of studies screened and reasons for exclusion indicated in a figure/flowchart; for iterative searching describe reasons for study exclusion and inclusion based on modifications t the research question and/or contribution to theory development).*	See Page 4 and Figure 1 and 2
**10**	Rationale for appraisal	Describe the rationale and approach used to appraise the included studies or selected findings *(e.g. assessment of conduct (validity and robustness), assessment of reporting (transparency), assessment of content and utility of the findings).*	See Page 3-4

No	Item	Guide and description	
**11**	Appraisal items	State the tools, frameworks and criteria used to appraise the studies or selected findings *(e.g. Existing tools: CASP, QARI, COREQ, Mays and Pope* [25]*; reviewer developed tools; describe the domains assessed: research team, study design, data analysis and interpretations, reporting).*	See Page 4 and S1 CASP checklist
**12**	Appraisal process	Indicate whether the appraisal was conducted independently by more than one reviewer and if consensus was required.	See Page 4. 1 reviewer (the main author) initially assessed quality of included studies using the CASP criteria and noted any critical aspects of quality with the study team. During subsequent group discussions we continued to discuss and reflect on key aspects of quality. Due to the small number of eligible studies we decided to include all (please see discussion section and also S1 CASP checklist)
**13**	Appraisal results	Present results of the quality assessment and indicate which articles, if any, were weighted/excluded based on the assessment and give the rationale.	Please see discussion section and S1 CASP checklist
**14**	Data extraction	Indicate which sections of the primary studies were analysed and how were the data extracted from the primary studies? *(e.g. all text under the headings "results /conclusions" were extracted electronically and entered into a computer software).*	See Page 4 and S1 Box
**15**	Software	State the computer software used, if any.	N/A
**16**	Number of reviewers	Identify who was involved in coding and analysis.	See Pages 4
**17**	Coding	Describe the process for coding of data *(e.g. line by line coding to search for concepts).*	See Page 4

No	Item	Guide and description	
**18**	Study comparison	Describe how were comparisons made within and across studies *(e.g. subsequent studies were coded into pre-existing concepts, and new concepts were created when deemed necessary).*	See Page 4, S1 Box, S3 Table
**19**	Derivation of themes	Explain whether the process of deriving the themes or constructs was inductive or deductive.	See Page 4,S1 Box, S3 Table
**20**	Quotations	Provide quotations from the primary studies to illustrate themes/constructs, and identify whether the quotations were participant quotations of the author's interpretation.	See Results section and S2 Table
**21**	Synthesis output	Present rich, compelling and useful results that go beyond a summary of the primary studies (e.g. *new interpretation, models of evidence, conceptual models, analytical framework, development of a new theory or construct).*	See Results and discussion section. Also see our conceptual model illustrating our 'line of argument' (S3 Figure).

For peer review only - http://bmjopen.bmj.com/site/about/guidelines.xhtml

PRISMA Flow Diagram: Retention Review

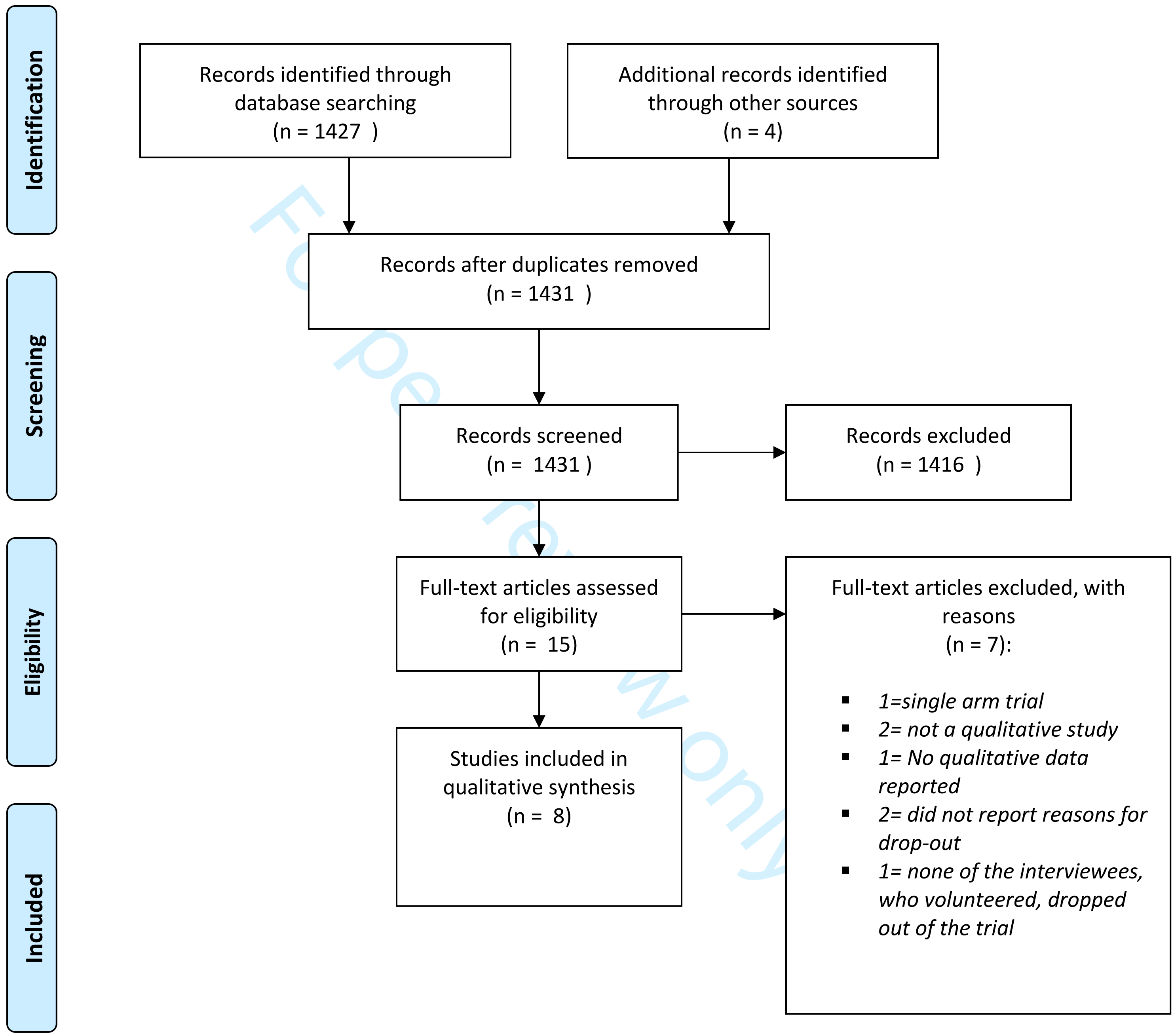

For peer review only - http://bmjopen.bmj.com/site/about/guidelines.xhtml

PRISMA Flow Diagram: Retention Review (search update)

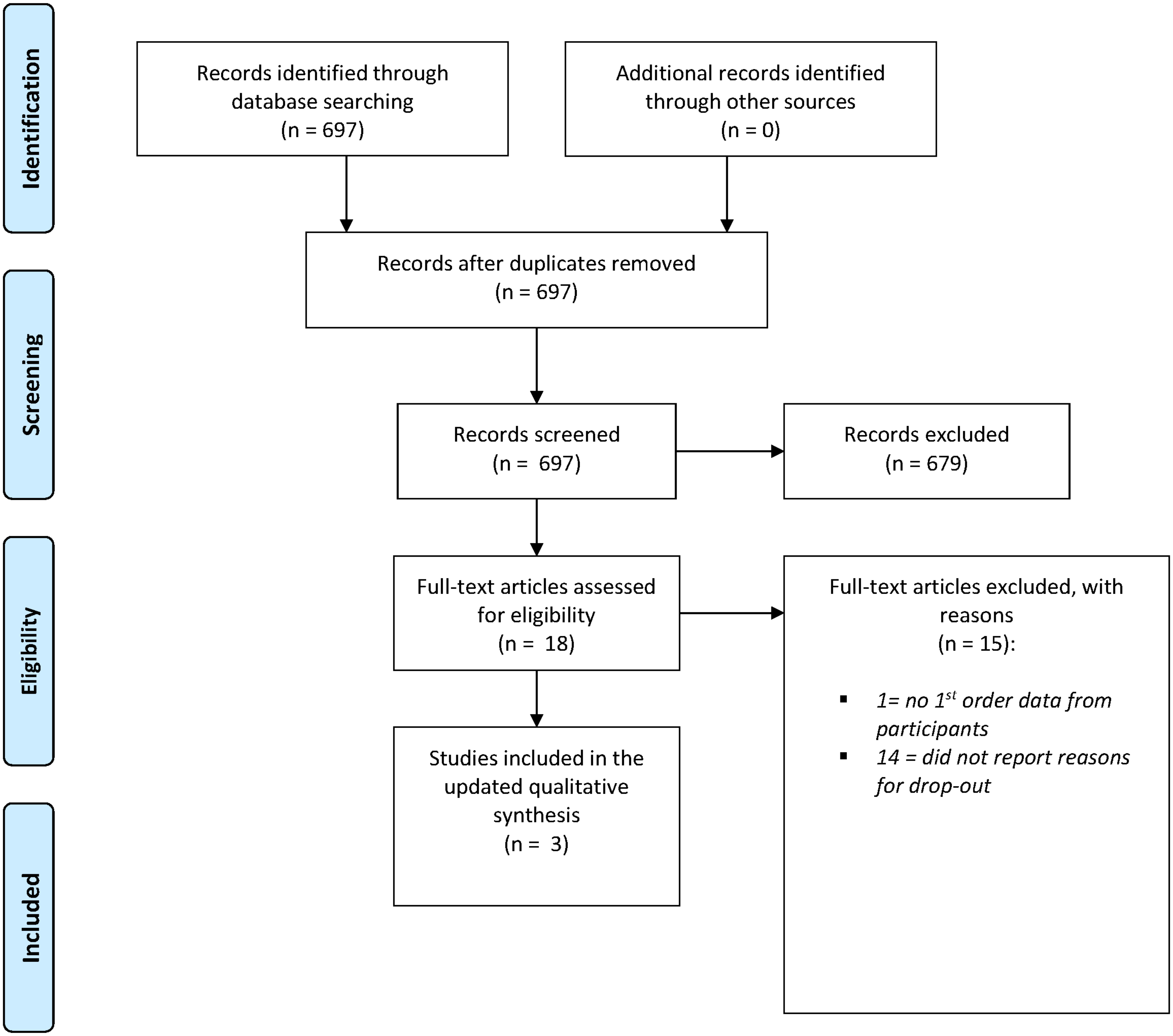

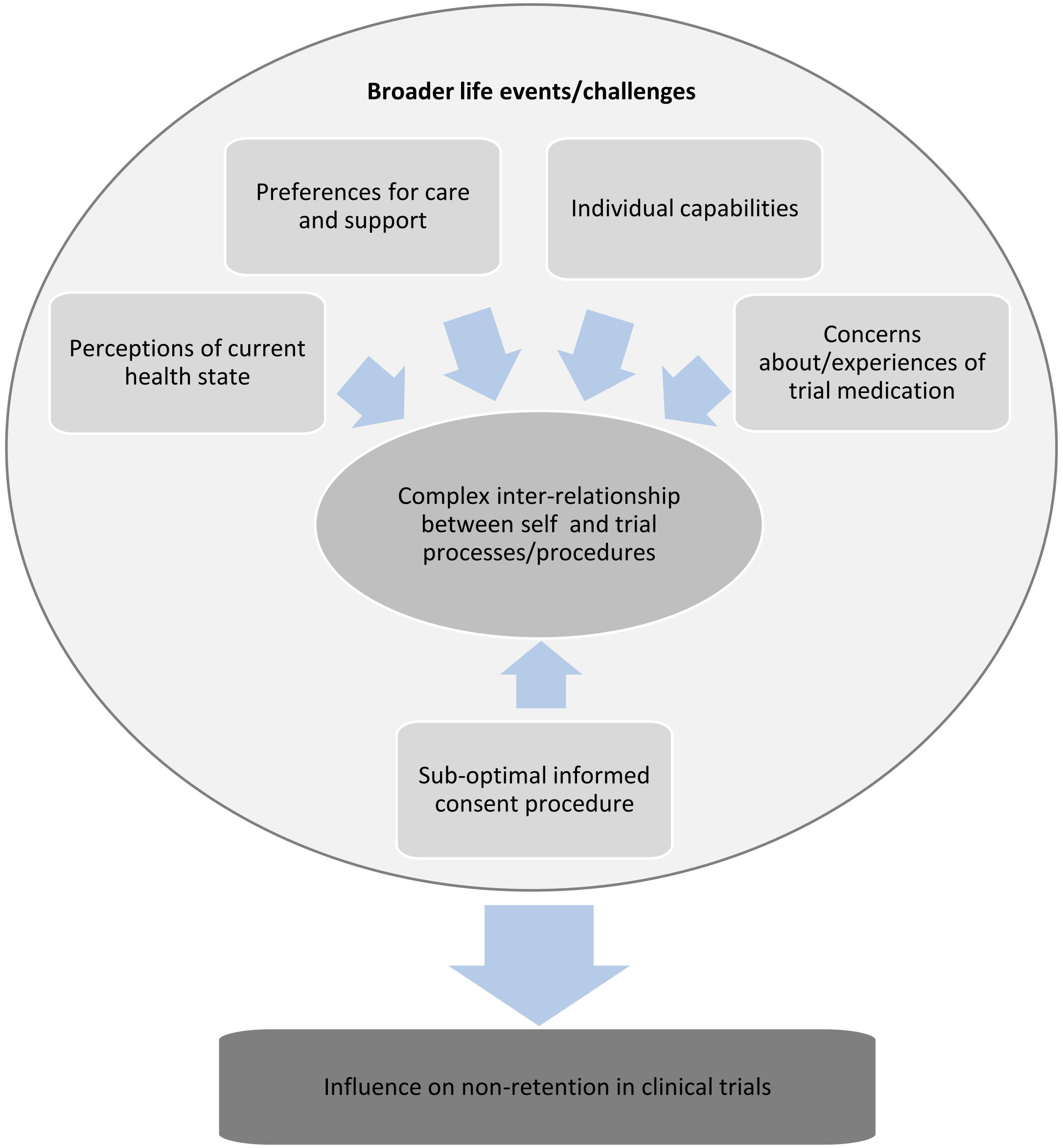
Broader life events/challenges
Preferences for care and support
Individual capabilities
Perceptions of current health state
Concerns about/experiences of trial medication
Complex inter-relationship between self and trial processes/procedures
Sub-optimal informed consent procedure
Influence on non-retention in clinical trials

S1 Table Characteristics of included studies

REF	COUNTRY	AIM	CONDITIONS OF FOCUS	PARTICIPANT CHARACTERISTICS	TRIAL COMPARATORS	ATTRITION BEHAVIOUR	DATA COLLECTION METHODS
Nakash et al 2007 [31]	UK	To examine factors affecting response and non-response from the clinical trial participant's perspective.	Severe ankle sprains	8 non-retainers. Age and gender of non-retainers unclear although of the 22 included in this study, 11 were male; 11 female, aged from 16 to 62 yrs (mean age 34 yrs). A purposive sample was sought to represent the diversity of trial participants in age, sex, level of education, occupation and type of ankle support.	Different mechanical supports	Non-response to postal questionnaire follow-up. 8 had not responded to at least one of their follow up questionnaires.	Semi-structured interviews
Nicholas et al 2010 [21]	Australia	To identify participants' reasons for non-adherence to, and attrition from the online intervention	Newly diagnosed bipolar disorder	39 non-retainers. 22 female; 17 male. 20 were aged less than 30 yrs, 14 were married, 29 were tertiary educated and 24 in full time employment. Participants from all 3 study groups were interviewed, 16 from the unsupported intervention group (BEP), 9 from the supported BEP intervention group (BEP+IS), and 14 from the minimal	1 of 2 active interventions (online psycho-education program either alone or with email support from informed supporters) or an attention control condition (online information about bipolar disorder presented in text as bullet points). Both active interventions and control contained 'workbook' activities.	Cessation of therapy sessions and/or non-completion of some or all intervention workbooks; non-completion of some or all control workbooks.	Semi-structured interviews

For peer review only

S1 Table Characteristics of included studies

				information control group.			
Postel et al 2010 [22]	Netherlands	To evaluate an e-therapy program with active therapeutic involvement for problem drinkers. Reasons for drop out were also investigated via a ‘dropout’ questionnaire consisting mainly of open questions.	Problem drinking	40 non-retainers. No gender or age details provided specifically for non-retainers, although 53.8 % of trial participants were female, mean age 45.3 yrs. Authors also state that in the control group more non-responders than responders were male.	3 month e-therapy programme (consisting of a structured 2 part online treatment programme with asynchronous therapist contact via the internet only) or a waiting list control group (receiving ‘no reply’ email messages once every 2 weeks). Participants completed online self-report questionnaires at baseline and at 3 months follow up (control group) or at posttreatment, which was approximately 3 months (e therapy group). Weekly alcohol consumption was assessed by a 7 day retrospective drinking diary.	Non-completion of treatment sessions and/or follow-up questionnaires. Non-retention was defined as anyone who did not complete the 3 month assessment. Dropouts in the e therapy group did not complete all 12 treatment sessions: 9 assignments and 3 assessments.	A ‘dropout’ questionnaire consisting mainly of open questions.
Eborall et al 2011 [30]	UK	To explore people’s explanations for declining to participate in the trial, or, having begun the trial, stopping the trial medication	Asymptomatic atherosclerosis	17 non-retainers. No gender or age details provided specifically for non-retainers (as opposed to those who declined to consent), but states that total sample (n=28) had a mean age of 65.2 yrs; 19 were female; wide	Aspirin (100mg daily) or placebo for a mean duration of 8.2 yrs.	Cessation of trial medication (unclear whether active medication or placebo). Non-adherence with study medication throughout the trial was 40%; 15% took their medication for less than 6 months.	Semi-structured interviews (n=11) and one focus group (n=6)

For peer review only

S1 Table Characteristics of included studies

				range of socioeconomic backgrounds.			
Wells et al 2011 [23]	USA	To explore low-income, minority cancer patient perspectives about not adhering or dropping out of depression treatment.	Depression and cancer	20 non-retainers. No gender or age details provided specifically for non-retainers included in the qualitative study but trial non-retainers were described as predominantly female, foreign born, unmarried, unemployed, and older than 50 yrs.	Intervention or usual care. Intervention was an individualised stepped care depression programme provided by a cancer depression clinical specialist in collaboration with a study psychiatrist. Patients in the intervention group were offered antidepressant medication and/or problem solving treatment (PST).	Cessation of problem solving treatment sessions and/or anti-depressant medication.<br><br>PST dropouts were defined as patients who had fewer than 4 PST sessions. PST dropouts included those who initially agreed to be randomised to the intervention, but thereafter had either verbally declined treatment or did not show up for the therapy appointments. This included patients who had refused some sessions, but agreed to remain in the study for outcome interviews.<br><br>Patients receiving antidepressant medication were dropouts if they discontinued treatment within 30 days.	Semi-structured interviews
Shilling et al 2011 [27]	UK	To investigate recruitment processes across a range of clinical trials and from the perspective of parents, young people and practitioners to identify strategies to	Trial 1: Neuro-development disorders<br><br>Trial 2: Osteopenia	3 non-retainers from 2 of the 4 included trials. In trial 1, participants were 11-14 yrs; In trial 2, participants were 4-18 yrs (although demographic details of non-retainers unclear).	Trial 1: Melatonin versus placebo (over 12 week treatment period). Families made 3 additional hospital visits, and received 4 home visits by the research nurse and 3 telephone calls.	Cessation of trial medication (unclear whether active medication or placebo)	Semi-structured interviews (with the young people and/or their parents).

For peer review only

S1 Table Characteristics of included studies

		improve recruitment and its conduct across a spectrum of trials of medicines for children.<br><br>NB: This was a monograph that included the reporting of a range of trials, two of which reported data on non-retainers and were included in this meta-ethnography.			Trial 2: bisphosphonate risedronate or vitamin D analogue 1 – alphahydroxychol ecalciferol versus placebo (1 yr treatment period). Young people were seen 7 times over the year. This was timed to coincide with routine clinic visits where possible. Blood samples were also taken (same time as routine visits) and they gave regular urine samples and had 3 x-ray scans and 2 bone radiographs.		
Sanders 2012 [28]	UK	To explore barriers to participation and adoption of tele-health and tele-care from the perspective of people who declined to participate or withdrew from the trial	Diabetes, COPD, heart failure, or social care needs	3 non-retainers (all in the intervention arm), 1 female (diabetes); 2 male (1 x COPD; 1 x diabetes + heart and lung problems. 73-85 yrs.	The RCT was a cluster design with GP practices being randomised to receive access to telehealth or telecare for their populations. Participants randomised to the control arm were offered telehealth or telecare at the end of the 12mth trial.<br><br>Tele-health equipment included a monitor unit via which recordings from peripheral devices were uploaded to a monitoring centre. The monitoring centres prioritised and	Cessation of use of telehealth equipment or tele care devices (defined as withdrawing from the trial after joining the intervention arm).	Semi-structured interviews

For peer review only

S1 Table Characteristics of included studies

					tailored response according to need based on the information received.<br><br>Telecare interventions also varied according to assessed need but included various sensors to detect gas, water overflow, falls and movement around the property. Such sensors would trigger alarms direct to a monitoring centre if anything abnormal was detected, allowing emergency intervention.		
Johansson 2015 [24]	Sweden	To explore participants' experiences of non-adherence to internet-delivered psychological treatment	Generalised anxiety disorder	7 non-retainers, 6 female; 1 male; mean age 39.3 yrs	Intervention consisted of internet delivered psychological treatment, with weekly support from a licensed clinical psychologist. The treatment consisted of 8 weekly self-help modules of text, audio and illustrations, averaging 21 pages per module. All modules contained a homework assignment that needed to be answered and sent to the guiding therapist.	Non-adherence to internet delivered psychological treatment.<br><br>Non-adherence was defined as completing at least 1 and no more than 7 treatment modules (out of 8 in total).	Semi-structured interviews

For peer review only

S1 Table Characteristics of included studies

					No details re. control group given.		
Sari 2017 [25]	Denmark	To investigate perceived barriers to participate in an exercise intervention among alcohol use disorder patients, who dropped out from the Healthy Lifestyle Study	Alcohol Use Disorder	17 non-retainers, 4 female; 13 male; age 30-68 yrs. Nine were allocated to the group intervention, 7 were allocated to the individual intervention and one were allocated to the control group.	Treatment as-usual or one of two 6-month interventions that were selected on the basis of existing evidence-based studies. In the first intervention group, participants exercised individually after receiving basic instructions and a training program for home use. In the second intervention group, several patients exercised together with two instructors in 60-min training sessions twice a week. Running was the specific exercise form for both groups in the study.	Drop out was indicated when participants directly reported to project personnel or when they ceded participation without contact. Their length of participation before dropout varied from 2 days to 12 weeks.	Semi-structured interviews
Fernandez-Alvarez 2017 [26]	Spain	To conduct a qualitative analysis of the subjective experience of a sample of patients who dropped out of a transdiagnostic Internet based treatment for emotional disorders.	Obsessive-compulsive disorder; Agoraphobia;Social Anxiety Disorder; Major Depressive Disorder; Generalized Anxiety Disorder	10 non-retainers, 8 female; 2 men, age 21-59 yrs. Unclear whether control or treatment arm.	The protocol consists of 12 modules, and participants are encouraged to complete one module per week. Two RCTs are being conducted using the protocol. The purpose of one of the RCTs is to analyze the effectiveness of a transdiagnostic IBT compared to treatment as usual as provided in the Spanish public mental	Individuals who dropped out after completing a minimum of 3 modules.	Semi-structured interviews

S1 Table Characteristics of included studies

					health care system. Both treatment protocols are web-based, self-administered treatments with minimum contact/support from a therapist that consists in a weekly phone call lasting 5 to 10 min to each participant. A non-human support is delivered through two weekly mobile phone text messages that are automatically sent and aim to remind the participants of the importance of reviewing the modules as well as doing the homework tasks.		
Henshall 2018 [29]	UK	To determine the overall experiences of newly diagnosed adults with T1D in an exercise study, and to understand issues that influence the retention of trial participants in such studies	Newly diagnosed Type 1 diabetes	4 non-retainers; 2 female; 2 male age 19 - 55 yrs (2 in intervention arm/2 in control arm)	Conventional treatment or exercise, stratified on beta cell function and fitness. The exercise group were encouraged to increase their level of activity to a minimum of 150 minutes of moderate to vigorous intensity exercise per week, aiming for 240 minutes per week of exercise for 12 months.	No specific details given other than 'those who withdrew before the end of the study'	Semi-structured interviews

For peer review only

S1 Table Characteristics of included studies

For peer review only

For peer review only - http://bmjopen.bmj.com/site/about/guidelines.xhtml

S2 Table Key themes of influence on decisions to withdraw from trial participation, with corresponding example data.

Theme	Exemplary quote
**Perceptions of current health state in relation to specific aspects of the trial**	*"A very short while after doing the program I fell into another episode, a depressive episode, and pretty much stopped doing everything, the program included"[21, male, 18-29 yrs, BEP+IS group]*<br><br>*"I found it quite confronting, and reading the information made me feel uncomfortable, thinking that these issues related to me – I preferred the ostrich approach" [21, male, 40-49 yrs, BEP group]*<br><br>**This study demonstrates that reasons…were often explained in terms of potential threats to existing self-care, independence, the majority of respondents in this study depicted themselves as too healthy and too independent for the interventions to be of value [28, telehealth and telecare interventions in aging populations]**
**The 'fit' of aspects of the trial with individual preferences for care and support**	**Two respondents who withdrew from the trial described how the service changes they experienced caused additional stress. For example, one woman said she** *'did not want to be a nurse' …***and she was much happier to have returned to a regular appointment (fortnightly) with the community matron. Another man described the good care he received prior to joining the trial, but how he was subsequently discharged from the specialist professionals who had been involved in his care…..he described his main problems as** *'complex problems with my heart and breathing,'* **and that the faulty recordings and changes in service provision were causing him great stress [28, telehealth and telecare interventions in aging populations]**<br><br>**Some participants changed to other treatment formats after terminating the Internet-delivered therapy. One participant explained that she had never prioritised her own personal development and that an individual therapy consisting of face to face meetings was needed to get away from home and focus on the therapy [24, internet-delivered psychological treatment for people with generalised anxiety disorder]**
**The compatibility of aspects of the trial with individual capabilities**	**Twelve of the 20 patients interviewed acknowledged that they had dropped out of treatment, citing several reasons and circumstances related to dropping out of treatment. These**

For peer review only

	**included…cultural (which included language communication problems) [23, problem solving treatment sessions and/or anti-depressant medication for people with depression and cancer]**<br><br>**Difficulty with literacy [31, Different mechanical supports for people with severe ankle sprains]**<br><br>**One critique was that information was difficult to understand because the content was perceived as complex and abstract. In some cases the participants felt unintelligent for their inability to understand [24, internet-delivered psychological treatment for people with generalised anxiety disorder]**
**Concerns about or experiences of trial medication**	**One mother's discovery, via an internet search done by the child's father, that the trial drug was unlicensed for children had left her concerned about the safety of the trial and she subsequently withdrew her child from MENDS [trial] [27]**<br><br>*"I really felt I'd got gall bladder trouble again because [the pain] was from here right through into me kidneys and really severe. So I went to my GP, and she just checked round and said straight away, 'don't take anymore, and ring [the trial] and tell them'"[30, Aspirin for people with Asymptomatic atherosclerosis]*
**Considerations around extent to which trial participation could be appropriately accommodated into broader life circumstances**	**The participants' statements regarding non-adhering showed an incompatible relationship between the length of the weekly text modules and factors or conditions in the personal life of the participants [24, internet-delivered psychological treatment for people with generalised anxiety disorder]**<br><br>*"…as a student you read so much already. I felt like I couldn't muster more energy or more time to spend by the computer and to read 10 or 20 more pages and also answer questions. It felt as if you were inclined to have a very structured life already to handle that" [24, internet-delivered psychological treatment for people with generalised anxiety disorder]*<br><br>**These were reasons such as pregnancy, exams, or work commitments which participants felt were the cause of their non-response [31 Different mechanical supports for people with severe ankle sprains]**

For peer review only

	**We could establish that in the e-therapy group 11 participants dropped out because of personal reasons unrelated to the e-therapy program or the study (eg, ill family member) [22]**

Primary study participant quotes (1st order constructs) are displayed in italics and primary study author interpretations (2nd order constructs) are presented in bold. Study references and details of participants/interventions where available have been added to the end of exemplar quotes.

For peer review only

S3 Table

	Inter-relationship between self and trial processes/procedures													
**Themes**	**1. Perceptions of current health state in relation to specific aspects of the trial**		**2. The 'fit of aspects of the trial with individual preferences for care and support**						**3. The compatibility of aspects of trial processes with individual capabilities**		**4. Concerns about or experiences of trial medication**			**5. Considerations around the extent to which trial participation could be appropriately accommodated into individuals' broader lives**
14 Sub-themes	Perception of being too well	Perception of being too ill	Not individual /tailored/ personalised enough	Too basic	Inflexible	Too technical	Too intensive	Too stressful	Not tailored to individual capabilities	Cognitively not pitched at the individual	Medication not necessary	Potentially dangerous	Contraindications or side effects	Aspects of life getting in the way
Nakash 2008	*									*				*
Nicholas 2010	*	*	*	*	*									*
Postel 2010	*						*	*						*
Eborall 2011	*										*		*	*
Wells 2011	*	*								*				
Shilling 2011												*	*	
Sanders 2012	*			*	*	*								
Johansson 2015	*	*	*		*				*	*				*
Sari 2017	*						*							*
Fernandez-Alvarez 2017		*	*	*										*
Henshall 2018		*												*

*Denotes presence of themes across papers

For peer review only

For peer review only - http://bmjopen.bmj.com/site/about/guidelines.xhtml

BMJ Open

Exploring non-retention in clinical trials: A meta-ethnographic synthesis of studies reporting participant reasons for drop out

Journal:	*BMJ Open*
Manuscript ID	bmjopen-2018-021959.R3
Article Type:	Research
Date Submitted by the Author:	23-Apr-2019
Complete List of Authors:	Skea, Zoë; Universit of Aberdeen, Health Services Research Unit<br>Newlands, Rumana ; University of Aberdeen College of Life Sciences and Medicine, Health Services Research Unit<br>Gillies, Katie; University of Aberdeen, Health Services Research Unit
**Primary Subject Heading**:	Health services research
Secondary Subject Heading:	Qualitative research
Keywords:	QUALITATIVE RESEARCH, Clinical trials < THERAPEUTICS, Retention

SCHOLARONE™
Manuscripts

Exploring non-retention in clinical trials: A meta-ethnographic synthesis of studies reporting participant reasons for drop out

Author: Dr Zoë Christina Skea,

Corresponding author: Dr Zoë Christina Skea, Health Services Research Unit, 3rd Floor, Health Sciences Building, University of Aberdeen, Aberdeen, AB25 2ZD, Tel: +44 (0) 1224 438153. email:z.skea@abdn.ac.uk. Fax: +44 (0) 1224 438165.

Co-authors: Dr Rumana Newlands, University of Aberdeen, Aberdeen, UK. Dr Katie Gillies, University of Aberdeen, Aberdeen, UK.

Key words: Qualitative research; Clinical Trials; Retention.

Word count: 7328

Abstract

Objectives

To undertake a meta-ethnographic synthesis of findings from primary studies reporting qualitative data that have explored participant reported factors influencing non-retention within a clinical trial context.

Design

A systematic search and meta-ethnography was conducted for published papers (from 1946 –July 2018) that contained qualitative data from trial non-retainers.

Participants

We identified 11 studies reporting qualitative data from 13 trials. The studies were undertaken between 2008 and 2018. Each study included between 3 and 40 people who had dropped out from a trial, with findings from 168 people in total reported across the papers.

Results

Emergent from our synthesis was the significance of trial non-retainers' perceptions around the personal 'fit' of key aspects of the trial with their personal beliefs, preferences, capabilities or life circumstances. These related to their own health state; preferences for receiving trial 'care'; individual capabilities; beliefs about or experiences of trial medication; and considerations whether trial participation could be accommodated into their broader lives. All these factors raise important issues around the extent to which initial decisions to participate were fully informed.

Conclusions

To improve retention in clinical trials, researchers should work to reduce the burden on trial participants both through the design of the intervention itself as well as through simplified data collection processes. Providing more detail on the nature of the trial interventions and what can be expected by 'participation' at the consenting stage may prove helpful in order to manage expectations.

For peer review only - http://bmjopen.bmj.com/site/about/guidelines.xhtml

Strengths and limitations of this study

- Trial retention has recently been identified as one of the top three priorities for methodological research by UK trialists.
- Within the context of clinical trials, issues around retention have not received equal scrutiny compared to methodological questions about trial recruitment despite being arguably just as important for trial validity.
- Understanding the complex reasons why trial participants leave a trial after initially consenting is important if trialists are to be able to design effective intervention strategies to address the problem.
- To our knowledge this is the first synthesis of key qualitative findings from studies exploring participants' perspectives of trial non-retention which provides learning across their collective contributions.
- Our synthesis only included 11 eligible papers reporting findings across 13 trials, 5 of which were set within a mental health context and all of which were conducted in high-income countries. This could have issues for the transferability of findings.

Introduction

Randomised controlled trials are integral for evidenced based clinical decision making. Within the context of clinical trials, the focus of much methodological research in recent years has been on issues specifically relating to trial recruitment, including significant investigation into how to increase the numbers of prospective participants recruited [1,2]. A key focus of much of this research has been on trial participants' perspectives and experiences particularly around why they do or do not choose to consent to participate in clinical trials [3,4,5,6,7,]. Whilst issues relating to trial recruitment are undoubtedly important, issues around retention (i.e. ensuring that trial participants remain in the trial to provide primary outcome data) have not received equal scrutiny in the literature despite being arguably just as important for trials in terms of ensuring that research questions are adequately answered [2].

Trial retention was recently identified in the top three priorities for methodological research by UK trialists [8]. Most trials experience the issue of missing data often referred to as a 'loss to follow-up', 'attrition' or 'drop out' and this can bias the findings of a trial. Some recent quantitative surveys have identified participant characteristics (e.g. age, gender, physical or mental health) or trial

For peer review only - http://bmjopen.bmj.com/site/about/guidelines.xhtml

processes (e.g. study duration or length and relevance of outcome measures) as being potential predictors of trial retention [9,10,11]. However, these studies are small in size, often limited to a particular clinical context, and the items included in the surveys are often identified by researchers rather than asking participants what items should be included. In addition, they lack any in depth exploration of the relevant issues affecting why participants withdraw, as reported by participants.

Understanding the complex reasons why trial participants leave a trial (either actively (e.g. by requesting no further follow up or purposefully not returning data) or passively (e.g. forgetting to return a questionnaire or attend a clinic visit)) after initially consenting to participation is important especially if those reasons are modifiable. This understanding of participant perspectives then becomes crucial if trialists are to be able to design effective intervention strategies to address the problem.

The approach of conducting in-depth qualitative research within the context of clinical trials is considered particularly useful for improving the evidence base for how trialists conduct them [12]. Indeed this approach has been used widely to explore perspectives on trial recruitment both in terms of primary qualitative studies and secondary syntheses. To our knowledge this is the first synthesis of key findings from studies exploring participants' perspectives of trial non-retention which provides learning across their collective contributions. Our aim was to undertake a meta-ethnographic synthesis of findings from such studies and our specific research question was 'what influences non-retention in clinical trials'?

Methods

A systematic literature search and meta-ethnography was conducted (See S1 ENTREQ Checklist). This meta-ethnography was undertaken in two parts. Our original systematic search and synthesis was undertaken in August 2016. To integrate potentially more recent relevant research, we undertook an update in July 2018.

Meta-ethnography essentially involves an 'interpretive and inductive' approach to synthesising studies [13,14]. Essentially meta-ethnography involves the process of 'translating' the findings of individual qualitative studies so that they can be considered in relation to one another with the aim of identifying and building new conceptual knowledge on a particular topic [13,14]. The process of 'translating' findings across studies can be either 'reciprocal' or 'refutational' depending on how individual studies relate to each other [13].

Searching and identification of relevant studies

A systematic search was conducted for published papers that contained qualitative data about trial participants' reasons for not completing some or all of the processes involved in a clinical trial after initially consenting to take part (which we describe as constituting non-retention). Search strategies were informed by previous studies [12] and are provided in Appendix 1. Seven electronic databases were searched by an information specialist: Embase, Ovid MEDLINE, PsycINFO, Cochrane Central Register of Controlled Trials, The Social Sciences Citation Index (SSCI), Cumulative Index of Nursing &

For peer review only - http://bmjopen.bmj.com/site/about/guidelines.xhtml

Allied Health Literature, and Applied Social Sciences Index and Abstracts (ASSIA) and covered papers published from 1946 to August, 2016 (first search) and from August 2016 – July 2018 (updated search). Google Scholar and bibliographies of identified publications were also searched manually for additional potentially eligible papers.

For both searches, one author screened all titles and abstracts (RN for original search; ZS for update) with a second author (KG) screening a random 10% sample. Eligible studies included those that used qualitative methods and contained qualitative data exploring any aspect of non-retention from the perspective of patient participants (recognising that non-retention might cover activities such as cessation of or withdrawal from the intervention(s), non-attendance at clinic visits, through to non-response to some or all follow up questionnaires etc).

Analysis and synthesis

In order to collate and synthesise the available primary research, the seven steps of meta-ethnography as listed in S1 Box were followed. In summary, the three authors (ZS, RN, KG) each read and systematically extracted data from the included papers, shared notes and discussed study findings and interpretations during a series of group meetings. The papers were initially organised in chronological order (but as inductive analysis progressed papers were grouped according to emerging themes) and we focused on the findings, concepts and themes used by the papers' authors generating a list of key categories. We used a standard form which summarised the main themes, information regarding methods, and any other important information relating to the context of the research within each study (some of this data is illustrated in S1 Table). Although we initially organised papers chronologically in this table, we used it to facilitate a series of further group discussions around emerging issues (See Table S2 for examples). As inductive analysis progressed we grouped and discussed our data according to the 5 key emerging themes (See S3 Table). In line with the process of undertaking a meta-ethnography, primary data or 'first order constructs' (quotations from study participants who had not completed any or some of the various trial processes) and authors' interpretations of these data ('second order constructs') were extracted, compared and contrasted between studies (enabling us to produce a 'reciprocal translation'), and organised into themes to facilitate the development of new insights or a 'line of argument' [13].

Study Quality

One author (ZS) undertook a quality assessment of each of the papers included in the synthesis. This was based on the Critical Appraisal Skills Programme (CASP) criteria [15] which was used to appraise the identified primary studies and consider their inclusion into the synthesis (See S1 CASP Checklist). Questions developed by the CASP have been used previously for appraising the quality of studies for inclusion in meta-ethnography [16,17,18,19,20].

Patient and Public Involvement

This research was done without patient involvement. Patients were not invited to comment on the study design and were not consulted to develop patient relevant outcomes or interpret the results. Patients were not invited to contribute to the writing or editing of this document for readability or accuracy.

For peer review only - http://bmjopen.bmj.com/site/about/guidelines.xhtml

Results

Description of Studies

The database search produced 1431 abstracts for the initial search and 697 abstracts for the update (see S1 Figure and S2 Figure for details). We only included studies that provided data about reasons for non-retention from the included study participants and/or in the authors' reflections. In all, 11 papers met our inclusion criteria (8 were identified from the initial search and 3 from the update). The focus and key study characteristics for the 11 included papers are outlined in S1 Table. The identified papers were conducted in 7 countries (UK, USA, Australia, Sweden, The Netherlands, Denmark and Spain) and discussed non-retention in 13 separate trials. Six of the papers focussed solely on reasons for non-retention [21,22,23,24, 25, 26], with the remaining 5 also considering reasons for consenting [27], non-consenting [28,29] and retention [30,31]. The findings in this synthesis relate to the data from non-retainers only. Each study included between 3 and 40 people who had dropped out from a trial, with findings from 168 people in total reported across the papers. As can be seen from S1 Table the setting of the trials in which the qualitative research was embedded included a range of clinical contexts such as: mental health problems [21,24,26]; mental health problems and cancer [23]; problem drinking [22, 25]; Type 1 diabetes [30]; diabetes, Chronic obstructive pulmonary disease, heart failure, or social care needs [28]; severe ankle sprains [31]; asymptomatic atherosclerosis [29]; neurodevelopment disorders [27], and osteopenia [27]. As expected, the clinical context differed as did the interventions under investigation and included: telehealth equipment or tele care devices [28]; web-based psycho-educational/cognitive therapy based support tools [21,22,24, 26]; anti-depressant medication and/or cognitive behavioural therapy [23]; exercise [25,30] various mechanical ankle supports [31]; aspirin [29]; melatonin [27]; and bisphosphonate risedronate or vitamin D analogue 1 – alphahydroxychol ecalciferol [27].

Findings were presented from trial non-retainers both before outcome data had been collected (e.g. those who withdrew from the intervention) and/or during the follow up when outcome data was being collected – in other words, papers included a mix in terms of non-retention behaviour (See S1 Table for a summary of non-retention behaviour i.e. non-adherence to intervention, non-return of questionnaires). For example, 8 studies reported aspects related to non-adherence to trial intervention: 3 of these reported cessation of trial medication [23,27 for both trials,29]; 5 reported cessation of treatment therapy sessions [21,22,23,24,26]; 1 reported cessation of use of telehealth equipment or tele health devices [28] and another reported non-completion of study workbooks [21]. Two studies reported non-return of follow-up questionnaires [22, 31]. For 2 studies, non-retention behaviour was unspecified [25,30] Three of the 11 studies appeared to have included only the views of those who had dropped out of the active intervention arms of the trial [23,24,28]. For 4 studies it was unclear whether data was from intervention or control groups [27 for both trials, 26,29,31,] and only 4 studies specifically stated that they included views of both those in the intervention and control groups [21,22,25,30].

Nine of the 11 studies used semi-structured interviews to collect data from people who had withdrawn from the main trial [21,23,24,25,26, 27,28,30 31]; 1 used a combination of focus groups and interviews[29] and another distributed a questionnaire that contained various open ended response options [23] (NB: only the qualitative data are reported and referred to in this paper). Although some papers provided gender, age and/or demographic details for participants taking part

For peer review only - http://bmjopen.bmj.com/site/about/guidelines.xhtml

in the trial in question, as can be seen from S1 Table, this information was less comprehensive for those who had dropped out of the trial. Where participant characteristic information was provided in the original studies we have included this at the end of the quotes presented to illustrate findings.

Key themes from the synthesis

Our grouping of first and second-order constructs across the 8 initially identified papers resulted in 14 sub-themes. During the process of translating themes from each of the individual studies (i.e. comparing and contrasting across studies) these sub-themes were then grouped and categorised into 5 broad key themes which characterised the main considerations and features that appeared to influence non-retention in the trials under investigation (See S2 Table). For the 3 subsequently identified papers, we repeated the various stages of meta-ethnography - in essence comparing for 'fit' and checking for any additional themes [32,33]. For the update, we attempted to follow the 'extend and renovate the house' approach [33], which involves examining the newly included studies to establish whether they add new concepts or contribute to existing ones. During this process, we were confident that concepts identified in the later 3 papers supported and complemented our originally identified 5 key themes (from the original 8 studies) with no new concepts emerging.

These themes were: 1) Perceptions of current health state in relation to specific aspects of the trial; 2) the 'fit' of aspects of the trial with individual preferences for care and support; 3) the compatibility of aspects of trial processes with individual capabilities; 4) concerns about or experiences of trial medication; and 5) considerations around the extent to which trial participation could be appropriately accommodated into individuals' broader lives.

As these theme labels suggest, within them they accommodate a spectrum of views or experiences.

The 5 broad key themes identified as influencing participants' non-retention in clinical trials are illustrated with example data in S2 Table. In S2 Table, primary study participant quotes illustrating first order constructs are displayed in italics, and primary study author interpretations illustrating second order constructs are presented in bold text. In the rest of this paper, primary study participant quotes are displayed in italics.

Influences on participant non-retention in clinical trials: a line of argument

Expressed below is our 'line of argument' which is organised into themes to facilitate the development of cumulative insights (S3 Figure conceptually illustrates the line of argument developed from the synthesis). These themes appear to be weighed up during the participant's involvement in the trial and set alongside the complex inter-relationship between self and trial process/procedures and ultimately impact on their retention in the trial. Overall, our argument emphasises the significance of trial participants' perceptions around the 'fit' of key aspects of the trial (intervention and trial processes) with their personal beliefs, preferences, capabilities or life circumstances. These factors (which were not necessarily mutually exclusive) related to beliefs about their own health state, preferences for how they wanted to receive care, their individual capabilities, beliefs about or experiences of trial medication, and also considerations around the extent to which trial participation could be appropriately accommodated (or not) into their broader lives. All of these

For peer review only - http://bmjopen.bmj.com/site/about/guidelines.xhtml

were set against the overall backdrop of the balance between their sense of self and the trial processes and procedures – this providing the overarching explanation for the influence on retention in trials Implicit within several of these identified factors is the suggestion that there may have been deficits within the initial trial consenting process which led to participants (who subsequently withdrew) not being fully informed or at least not realising what the trial expected of them and what they could expect of the trial. These findings are discussed in more detail below and arranged across 5 key themes.

1) Perceptions of current health state in relation to specific aspects of the trial.

This theme describes how aspects of the trial might not be right for people as individuals. For example, across 8 of the 11 studies a key influence on decisions to discontinue trial participation appeared to relate to perceptions of either being 'too well' to warrant further engagement with the trial [21,22,23, 25, 28,29,31] or struggling with the compatibility of aspects of the trial, particularly the interventions or ways outcomes were assessed, with their personal sense of self [21,22,25,28,29,30]. Conversely, other participants described periods of feeling too unwell to be able to engage appropriately in trial processes.

a) Being too well to engage further with trial processes

Some participants cited a belief that they had suitably recovered part way through a particular trial as a reason for discontinuing trial medication and/or problem solving treatment exercises [21,22,23, 25]. For some, this was also linked to not wanting to be reminded about health issues that they considered to be over:

"*I just don't want to be reminded of the alcohol thing, because I actually think it's over*" [25; Female, 30-68 years, Alcohol Use Disorder]

"Things really improved for me…I just felt really good and didn't really feel like I had that much to offer in regard to finding out more about it" [21; Female, 30-39 years, Bipolar disorder, control group]

"I have been sufficiently helped" [22; No gender/age details, Problem drinker, Intervention group]

Participants also cited recovery as a reason for not completing and returning all the required follow-up outcome assessment questionnaires [28, Severe ankle sprains] perhaps highlighting here the importance at the consenting stage of making sure participants are fully informed about the value of sustained engagement throughout the duration of the trial (even if they feel they are no longer personally benefiting from that engagement).

b) Lack of compatibility with personal sense of self

Sometimes reasoning around trial withdrawal related to participants' struggle to accommodate aspects of the trial with their personal sense of self at the time [25,28,29], suggesting that the intervention challenged their sense of self somehow. Again perhaps indicating the importance for initial trial recruitment consultations to include adequate discussions about the nature of the study intervention and also what will be expected of participants in terms of engagement with them. For example, a belief that they could self-manage or cope well enough without the need to engage with

For peer review only - http://bmjopen.bmj.com/site/about/guidelines.xhtml

the trial support intervention [28; self –care intervention to facilitate support for self-management in aging populations]; a belief that they were too overweight and unfit to participate in a group exercise intervention [25; exercise intervention for people with alcohol use disorder] a belief that they had adequately managed their condition thus far without the need for any medication [29; aspirin for asymptomatic atherosclerosis] and also non-acceptance of a diagnosis amongst those newly diagnosed [21; with bi-polar disorder; 30; with type 1 diabetes] as a reason for not relating to (or seeing any value in) the study interventions:

[Discussing the need to keep active rather than monitoring his health indoors using tele-health equipment] *"You've got [to have] the will power…if you can't do it I am finished. If I wouldn't have that I'd be, I'd be stuck inside here you know, and looking through the window like…I throw myself in the garden and everything. Everything I do I'm working on, I cook myself dinners and everything.'* [28; Male; 85yers; COPD]

"I think if it had been medication that I needed to take, I would have taken it" [29; Male; 72; stopped taking aspirin medication]

"If you're taking a lot, it knocks the hell out of your stomach…Given the choice, I'd rather not take medication full stop" [29; Male; 55; stopped taking trial medication for asymptomatic atherosclerosis]

"I wasn't ready to accept the illness. At that stage after diagnosis I wasn't willing to change my life according to the program." [21; Male; 18-29 yrs; Bipolar disorder, control group]

"*Don't think it kind of really sank in as to what I'd been diagnosed with … It had kind of hit me and I wasn't really dealing with having it* … [30; Female, 19-55yrs; Type 1 diabetes]

If trial participants believed that the trial did not fit with their personal sense of self this was also linked to an emotional response. For example, feelings of guilt and shame that they were too overweight and unfit to participate in a group exercise intervention [25; exercise intervention for people with alcohol use disorder].

c) *Being 'too ill' to be able to engage appropriately with trial interventions*

Conversely, within all of the papers focussing on interventions for mental health conditions, and in 1 paper focussing on people newly diagnosed with Type 1 diabetes, participants described being 'too ill' to be able to engage appropriately in trial processes [21,23,24, 26,30]. Reasons discussed in this context related to feeling either too fragile, depressed, too manic, or too emotional/stressed at certain times to be able to complete the required intervention tasks (e.g. e-health intervention and associated workbook activities; cognitive behavioural therapy; taking blood samples) and also a concern that engagement with the intervention could act as a 'trigger' in terms of exacerbating anxiety symptoms:

"I was feeling that the therapy wasn't going to help me with my problems. I thought it could lead me to be even more anxious and that it wasn't going to be beneficial for me. So, I felt that I was going to waste my time if I continued" [26; no gender details, 21-59 yrs, people with a range of serious mental health problems]

For peer review only - http://bmjopen.bmj.com/site/about/guidelines.xhtml

"I did not cope with the exercises. I did them at the start but it gradually became more difficult to complete them…..particularly the breathing exercises. I got a bit dizzy and it increased my feelings of anxiety" [24, no gender or age details, generalised anxiety disorder]

"The biggest problem I have with my bipolar disorder is consistency; when I'm down I can't even brush my teeth or get up in the morning. So doing an education program with workbooks was beyond me" [21, Female, 18-29 yrs, Bipolar disorder, BEP group]

"I often go walking when having highs because I have to keep moving, so I didn't want to sit at a computer" [21, Male, 40-49 yrs, Bipolar disorder, BEP+IS group]

As with the earlier sub-themes in this section, emotional influences were also woven through this perception of being 'too ill' to engage with the trial. One study pointed to the 'emotional impact of the cancer diagnosis' as being an influential factor linked to participant drop out [23].

2) The 'fit' of aspects of the trial with individual preferences for care and support

Across 8 of the 11 studies another important influence in decisions to discontinue trial participation appeared to relate to the fit of aspects of the intervention with preferences for how participants wanted to receive care and support [21, 22,23,24,25,26,28,31], implicitly suggesting that the initial trial consenting process may have been sub-optimal in key ways. Participants in these trials discussed how aspects of the design of the interventions were not individualised or tailored enough to be helpful and others commented on interventions being either too technical, too physically demanding, too intensive or conversely too basic:

"I needed a therapy that could better address what I felt. It didn't give me a specific answer to my worries". [26, no gender details, 21-59 yrs, people with a range of serious mental health problems]

"I would have liked to have more of a personal contact, it became a little distant everything, to do on the internet, because it is so heavy stuff, it's nice to meet a real person when you're working with heavy things like this" [24, no gender or age details, generalised anxiety disorder]

"I wanted something more about me specifically, as opposed to talking about general issues" [21, Male, 40-49 yrs, Bipolar disorder, BEP group]

"The information in the modules was too general and too limited" [21, Male, 18-29 yrs, Bipolar disorder, BEP group]

Some other participants simply indicated that they had been unhappy or dissatisfied or "not comfortable" with the treatment they had received although specific reasons were not provided within the included studies [22,23,31].

3) The compatibility of aspects of trial processes with individual capabilities

Across 3 of the 11 studies [23,24,31] the extent to which aspects of the interventions were deemed to be appropriately 'pitched' at the individual emerged as being of importance. For example, participants cited attention problems and limited reading and writing skills as a reason for withdrawing from internet delivered cognitive behavioural therapy [24] or as a reason for non-

For peer review only - http://bmjopen.bmj.com/site/about/guidelines.xhtml

response to follow-up questionnaires [31], with participants in one of these studies stating that they felt unintelligent because of their inability to understand [24].Communication and cultural issues were also cited as reasons for the discontinuation of problem solving treatments [23], suggesting that these issues would benefit from greater consideration and discussion at the consenting stage:

"I thought that it was too much to read, and I cannot read anything at all that I need to remember or learn. It goes in here and out there [pointing at the ears]" [24, no gender or age details, generalised anxiety disorder]

4) Concerns about or experiences of the trial medication

Across 2 of the 8 studies which were set within trials testing drug interventions, [27 – 2 trials; 29] concerns about the study medication were cited as reasons for discontinuing with trial participation. These included concerns that the trial drug(s) were not properly tested/licensed [27], concerns that the trial medication could negatively interact with other prescribed medication [29], through to citing a dislike of taking too much medication [29] or that the trial medication tasted offensive [27]. Constructs within this key theme again suggest potential issues with the informed consent process and highlight the importance of discussions about the purpose of any trial, the nature of trial medications and also the implication for participation of having certain co-morbidities, linking back in to the complex inter-relationship between self and trial process/procedures:

"It just scared me when it said not to be given to children under 20…I didn't understand they weren't licensed for children…and that's what I thought it was, just to see if it worked, not to actually like so then it could be licensed" [27; Mother of child in trial for young people aged 4-18yrs with rheumatic diseases]

"again I found that I had stomach problems with the tablet so I assumed that it must be the aspirin…[29, Female, 63 yrs, stopped taking trial medication for asymptomatic atherosclerosis]

"..and they discovered I had heart fibrillation…After that I'd to go on warfarin you see, so that's why I had to drop out because warfarin and aspirin just don't agree" [29, Female, 77 yrs, stopped taking trial medication for asymptomatic atherosclerosis]

"I didn't think I really wanted to go on at the start but mum and dad persuaded me to. And so…when I was getting really fed up I just said 'No I don't want to' because I didn't like the taste [of the medicine] [27; POP trial; young person 11-14 yrs]

5) Considerations around the extent to which trial participation could be appropriately accommodated into their broader lives

Aside from issues relating to beliefs about current health state, individual capabilities, preferences for care and concerns about side effects, participants also discussed how decisions to discontinue with trial participation related to other life 'events' that tended to take priority over or made it hard for them to engage fully with the various demands of the trial [21, 22,23,24,25,26,30,31]. These factors appeared less directly related to the nature of the trial interventions themselves and more about the challenges of life in general (with one study [31] suggesting that these people could be

For peer review only - http://bmjopen.bmj.com/site/about/guidelines.xhtml

classed more as 'happy' rather than 'unhappy' non-responders, in the sense that non-retention may be related to aspects out-with the trial itself). Reasoning here involved trading off trial participation with competing priorities and ranged from events such as work or family, moving to another country, exams, pregnancies, postal strikes etc and more generally simply daily routines that got in the way. Within one study [25], the importance of existing social networks was highlighted, with some participants citing a lack of support from family members as a reason for discontinuing trial participation. Within this theme participants also sometimes cited 'laziness' or 'forgetfulness' as reasons for why they had either not completed trial interventions or had not responded to follow-up questionnaires with some apparently being unaware that they were being considered as 'drop-outs' by study researchers:

[discussing cessation of therapy sessions/non-completion of study workbooks]*"I didn't have the time, and with everything else, it wasn't a priority"* [21, Female, 18-29 yrs, Bipolar disorder, control group]

[describing why they did not return a follow-up questionnaire] *"Do you know what…laziness I'm just gonna put it down to that"*

Researcher: *"OK and em it wasn't because you were disgruntled about part of the project?"*

"Definitely not no" [31, no gender/age details given, severe ankle sprains)

[discussing cessation of problem solving treatment sessions]"Did I drop out? No, I didn't dropout. I became busy and I figured I started missing calls." [23, Female, no age details, Cancer and depression)

Discussion

Principal findings

Our meta-ethnographic synthesis sought to explore factors that influence non-retention within clinical trial contexts. We identified 11 studies (reporting qualitative data from 13 trials) that explored participant reported reasons for not completing any or some of the various trial processes (after initially consenting to take part). What emerged from our analysis was the importance of trial participants' perceptions about the personal compatibility of key aspects of the trial with their personal beliefs, preferences, capabilities or life circumstances. These factors related to their own health state, preferences for how they wanted to receive care, their individual capabilities, beliefs about or experiences of trial medication, and also considerations around the extent to which trial participation could be appropriately accommodated or not into their broader lives (Conceptually illustrated in S3 Figure). Our synthesis has also highlighted that people's reasoning around dropping out of a trial can be described as being more or less 'active' in nature, with some people in our synthesis not even realising that they were being considered by the researchers as trial 'drop outs' [23]. All these factors raise important issues around the extent to which initial decisions to participate were fully informed and illustrate the importance for trial recruiters of ensuring that prospective participants are made aware of what the trial will entail and also what will be expected of them in terms of full participation.

For peer review only - http://bmjopen.bmj.com/site/about/guidelines.xhtml

Quantitative surveys have tended to investigate non-retention in the context of non-response to follow up questionnaires. These studies have identified either participant characteristics or trial processes as being potential predictors of trial retention [9,10,11].Whilst these studies have a place, it is arguably difficult to influence some of these previously identified factors influencing retention as they may not be modifiable e.g. age or study duration. Our synthesis of more in depth qualitative data has usefully built on these findings and has enabled a more nuanced understanding of key issues of relevance (which are potentially modifiable) relating to non-adherence to interventions and non-return of follow-up questionnaires. Participant characteristics as well as trial processes are of importance but we have also demonstrated that there can be a complex inter-relationship between the two. For example, a perception that the nature of the intervention negatively affects one's mental health can be of importance as can perceptions about the nature of the intervention in relation to perceptions of self or in relation to personal preferences for care and support. Furthermore, the compatibility or otherwise of various trial processes with individual capabilities can have implications for retention. Reasons given for not completing various trial processes were not necessarily mutually exclusive, but were rather a synergistic combination of factors that could apparently work towards trial non-retention. Our findings also highlight that some participants' behaviour around leaving a trial could be described as being more or less 'active' in nature (e.g. stopping trial medication because of a concern around side effects (active) versus simply not remembering or being too busy to return a questionnaire (passive)). This is an important finding and one that has not been given due consideration in previous literature to date. People's views and life situations can change over time, all having the potential to impact on their retention within a trial. Furthermore, different types of trials are likely to present particular challenges in terms of their potential for non-retention. It could also be that certain types of reasoning might be more or less modifiable and easier to address particularly if they can be anticipated upfront during the trial design stage.

A recent study exploring reasons why people declined trial participation at the consent to recruitment stage has found that most declined at the outset because they judged themselves ineligible or not in need of the specific trial therapy in question [34]. The study authors suggest that to improve recruitment to trials the most successful interventions are likely to be the ones that focus on patients' assessments of their own eligibility and their potential to benefit from the trial treatment, rather than reducing trial burden per se. In our synthesis we found that perceptions around eligibility and assessments regarding potential to benefit from the trial treatment were also considerations for people who had initially decided to join but who had subsequently ceased to engage. For example, this included those who felt that they had recovered such they did not need to engage further [21,22,23,25] and those who felt they could manage sufficiently well without engaging with the intervention [21,28,29]. However, in the context of non-retention, it is worth considering issues around trial burden (e.g. interventions that might be perceived to be too technical or too demanding given a person's health state) as well as issues around preference for particular styles of care and support and acknowledging that the specific intervention and, or, the ways outcomes are assessed has to be compatible within the context of trial participants' broader lives. In other words, issues around reducing trial burden is of importance, both in terms of the intervention itself and also the ways that follow up data is collected.

We know from previous syntheses of qualitative studies focusing on trial recruitment that people often choose to enter into trials in the hope of gaining some help for themselves from the

For peer review only - http://bmjopen.bmj.com/site/about/guidelines.xhtml

intervention (even if they also state they are doing so for altruistic reasons – i.e. to benefit research more generally), so called 'conditional altruism' [7]. Some participants in our synthesis described perceptions around feeling too ill to continue taking part or feeling suitably better such that trial engagement was no longer warranted [21,22,23,24,25]. This perception of improvement in health would appear to resonate with the concept of conditional altruism in the sense that people might cease participation if they perceive their condition improves or conversely deteriorates, such that in effect their benefit for self has been realised and their continued participation is no longer warranted. Our finding here is perhaps exaggerated in trials with a mental health context (which applied to 6 of the 13 included trials), where diagnoses can adversely affect people's ability and inclination to initially take part in research [34,35,36]. We have shown that this issue also has relevance for retention in such trials as people's health states can be particularly vulnerable to fluctuation [34]. A recent meta-synthesis of factors affecting recruitment to depression trials [37] indicated that decisions can depend on issues relating to: perceptions of health at the time of invite; attitudes towards the research and trial interventions; and the demands of the trial. Our synthesis has shown that some of this reasoning might also have the potential to impact on non-retention in those who are successfully recruited. Furthermore, previous research has suggested that the therapeutic alliance can have an impact on adherence to treatment [38]. Within the papers included in our synthesis, this was not something that was discussed per se. However, as one of our key themes illustrate, some decisions to discontinue trial participation appeared to relate to the fit of aspects of the intervention with preferences for how participants wanted to receive care and support. Within this, some trial non-retainers stated that they had wanted more face-to-face personal contact with for example, a therapist. This comparable finding could suggest that the underlying beliefs, preferences and expectations about trial participation are not explored and unpacked fully during trial consenting discussions.

Strengths and limitations

We recognise that different review teams may interpret qualitative data in slightly different ways due to pre-existing world views or expertise across research areas. However, a strength of undertaking a meta-ethnographic synthesis of findings from studies providing qualitative data on factors influencing non-retention within clinical trials is that it has allowed us to gain important new shared insights into factors that seem to affect retention across a range of trial contexts - to our knowledge this is the first study to have synthesised these primary studies in this way. Through synthesising, we have been able to pull insights from across studies, providing learning from their collective contributions. However, our systematic search identified only 11 eligible papers reporting findings across 13 trials, 5 of which had a mental health context and all of which were conducted in high-income countries. This in part perhaps reflects the difficulties researchers face in gaining access to the views of those who disengage with research. Furthermore, unlike for example surgical trials, all the included papers incorporated within their trials, interventions that participants could choose to discontinue engaging with (e.g. taking drugs; stopping CBT etc). Whilst qualitative research does not usually intend to be generalizable, it is nevertheless important to consider the transferability of our findings to other clinical trial contexts and settings and one could argue that participants within e.g. mental health trials, surgical trials, or trials that involve surrogate/proxy consent including those involving children [27] might face very different issues and challenges regarding retention. Although we were reassured that the key themes we identified had resonance across the included papers to a greater or lesser extent and so are likely to be important considerations within a range of clinical

For peer review only - http://bmjopen.bmj.com/site/about/guidelines.xhtml

trial contexts, some influences on trial non-retention are likely to be more trial specific than others (e.g. concerns about trial medication).

We carried out a quality assessment of the 11 included papers (see S1 CASP Checklist). Although all papers had study aims that were amenable to investigation via qualitative means and all included qualitative data, some were deemed richer than others in terms of data and insights (i.e. first and second order constructs). Arguably, this made undertaking a meta-ethnography in this context quite challenging as the number of studies and volume and/or quality of available data can affect depth of analysis. For example, 1 paper only reported qualitative data from open ended questionnaire response options [22], and 2 were deemed less useful in terms of presenting only very limited qualitative data (both first and second order constructs) (22,23)). Nevertheless, we did feel that they provided some helpful insights that usefully built on the findings of the other papers. Furthermore, despite some variation in the overall level of quality, due to the small number of included studies we felt it was more important to retain any relevant findings rather than disregard based on study quality. In doing so, we would argue that all 11 papers contributed useful elements to the collective whole and enabled us to develop our line of argument in terms of the issues of importance regarding trial non-retention.

Practice Implications

The way in which a trial is presented to individuals needs to take account of the influencing factors we have identified in this synthesis. Whilst not all the factors we identified are modifiable there influence needs to be recognised. We would argue that trialists need to think carefully about how the design of their trial might contribute to non-retention and that there is potential to modify trial design to improve retention.

To improve retention in clinical trials, researchers should work to reduce the burden on trial participants both through the design of the intervention itself as well as through simplified data collection processes. Providing more detail on the nature of the trial interventions and what can be expected by 'participation' (i.e. when and how data will be collected) at the consenting stage may prove helpful in order to manage expectations.

Some people in our synthesis appeared to be unaware that they were being considered as trial non-retainers by the study researchers. This raises the question of participants' understanding of the importance of remaining in a trial for its duration (i.e. completing the intervention and the outcome assessments) and its implications for the study in question. This finding is supported by a recent study of patient information documentation from UK NIHR funded trials that has highlighted that withdrawal and retention are poorly described and that statements about the value of retention are infrequent [39]. If trialists want to improve retention to clinical trials then there is an argument for giving the importance of completing the trial more prominence in patient information materials (and also during any trial recruitment discussions).

Our synthesis also potentially highlights the issue of people's awareness or lack thereof of what the trial interventions would entail. If trialists want to improve retention then this suggests an argument for also providing more detail on the nature of the trial interventions at the consenting stage in

For peer review only - http://bmjopen.bmj.com/site/about/guidelines.xhtml

order to manage expectations. We know from previous literature that patient/public involvement at the front end of trial design tends to be extremely limited if indeed it happens at all [40,41]. Given some of the key factors we found as being influential for non-retention, one could speculate that some early and meaningful patient/public involvement would be particularly useful (e.g. for ensuring that aspects of the trial are user-friendly and as compatible as possible with the target population's likely preferences and capabilities).

Implications for Research

A Cochrane review investigating interventions to improve retention in trials has highlighted that most strategies to improve retention have focussed on trying to improve follow-up questionnaire response [42]. Of these interventions, only monetary incentives have been shown to have a significant effect on return of questionnaires and the review highlighted that very few studies included trial participants in their design or development [42]. Our synthesis has demonstrated that there may be a range of issues relevant to trial participants that influence non-retention which may not be amenable to modification by 'incentives' or other interventions that fail to consider participants during development.

As mentioned previously, qualitative methods to improve recruitment to trials is now recognised as a well-established methodology built into the design and delivery of large publically funded clinical trials. The Qunitet Recruitment Intervention (QRI) is gathering momentum across a range of trials and Clinical Trials Units as a mechanism to unpack many of the nuances around how participants are recruited to RCTs [43]. Many of the approaches in the QRI are directly transferable to questions about retention. For example, how it is discussed in consultations and trial paperwork, what do stakeholders (trial participants and trial staff) report as the barriers and facilitators to retention, and work in this area could prove fruitful for minimising non-retention in ongoing RCTs. However, despite there being a clear need for more research in the context of trial retention, we also recognise the inherent challenges for researchers in obtaining the necessary ethical approvals for this type of research (particularly as current recruitment materials for trial participants tend to emphasise prospective participants' right to withdraw without given any reasons etc). Therefore, development of shareable resources to facilitate regulatory approvals may be an important contribution for the trials methodology community.

Finally, given that synthesis was based on a sparse data set, with 5 of the 11 included studies focused on qualitative research within mental health trials, there is certainly scope for more good quality, rigorous primary studies exploring the barriers and enablers to trial retention from a participant's perspective across a range of clinical specialties and trial design types. Interestingly, our search did not identify any studies that had explored reasons for trial participants' non-attendance at trial follow-up visits. Ideally, future studies should consider and explore all aspects of trial process relevant for retention, including completion and return of data (and its mode of delivery or collection), and attendance at follow-up visits. A recent prioritisation exercise for research into trial retention has now identified the Top 10 unanswered questions for trial retention [44]. Many of these Top 10 questions lend themselves well to enquiry by qualitative research methods and priorities should be focussed here.

For peer review only - http://bmjopen.bmj.com/site/about/guidelines.xhtml

Conclusions

Our systematic literature search and synthesis has highlighted that there is very little published qualitative literature exploring participant reported reasons for non-retention in clinical trials. Researchers have already called for 'a science of recruitment' in recognition that recruiting for science (e.g. trials) is not currently underpinned by an evidence base around the factors which might have the potential to impact on recruitment [1]. This is undoubtedly important but we would also argue that we need to develop a parallel focus on 'a science of retention' if we are to start to be able to tackle the very real issue of non-retention in clinical trials. Our qualitative synthesis (of albeit a small set of studies) feeds into this relatively undeveloped science and has shed some important light on the factors that might influence non-retention in clinical trials- factors that have implications both for practice and for further research. Taken together, the findings presented here and the subsequent implications for practice and research highlight the critical need to plan for retention as much as for recruitment during trial design and not treat it like the overlooked trial conduct 'Cinderella'.

Supporting Information

S1 ENTREQ Checklist

S1 Appendix. Database search strategies

S1 Box Steps of Meta-ethnography

S1 Figure. PRISMA flow diagram

S2 Figure. PRISMA flow diagram – updated search

S1 Table. Characteristics of included studies

S2 Table. Key themes of influence on decisions to withdraw from trial participation

S3 Table. Spread of themes across included studies

S3 Figure. Conceptual diagram

S1 CASP Checklist

Acknowledgements

We would like to thank Cynthia Fraser for her assistance in helping develop the database searching strategies.

Contributors

For peer review only - http://bmjopen.bmj.com/site/about/guidelines.xhtml

KG conceived the study idea. RN screened all titles and abstracts in initial search; ZCS screened all titles and abstracts in updated search with KG screening a random 10% sample from both searches. ZCS, RN and KG conducted the data analysis and ZCS wrote the initial and subsequent manuscript drafts. All the authors contributed critically to discussions about interpretation of data and revisions of manuscript drafts. All the authors approved the final version.

Funding

ZCS was supported by a core grant from the CSO (reference CZU/3/3) and a Wellcome Trust Institutional Strategic Support Fund award (reference RG12724-18). RN was supported by a Wellcome Trust Institutional Strategic Support Fund award (reference RG12724-18). KG was supported by an MRC Methodology Research Fellowship (MR/L01193X/1).

Data sharing statement

This is a review of published studies which are available to access through the relevant journals.

Competing interests statement

There are no competing interests for any author.

References

1. Bower P, Wallace P, Ward E, Graffy J, Miller J, Delaney B, Kinmonth AL. Improving recruitment to health research in primary care. Fam Pract. 2009;26(5):391–7.
2. Adamson J, Hewitt CE, Torgerson DJ. Producing better evidence on how to improve randomised controlled trials. BMJ. 2015;351:h4923.
3. Blanch DC, Rudd RE, Wright E, et al. Predictors of refusal during a multi-step recruitment process for a randomized controlled trial of arthritis education. Patient Educ Couns 2008;73:280–5.
4. Madsen SM, Holm S, Davidsen B, et al. Ethical aspects of clinical trials: the attitudes of participants in two non-cancer trials. J Intern Med. 2000;248:463–74.
5. Verheggen FW, Nieman F, Jonkers R. Determinants of patient participation in clinical studies requiring informed consent: why patients enter a clinical trial. Patient Educ Couns 1998;35:111–25.
6. Locock L, Smith L. Personal benefit, or benefiting others? Deciding whether to take part in clinical trials. Clin Trials 2011;8:85–93.
7. McCann SK, Campbell MK, Entwistle VA. Reasons for participating in randomised controlled trials: conditional altruism and considerations for self. Trials 2010;11:31.
8. Smith CT, Hickey H, Clarke M, Blazeby J, Williamson P. The trials methodological research agenda: results from a priority setting exercise. Trials. 2014;15(1):32.
9. Applebaum AJ, Lichtenthal WG, Pessin HA, et al. Factors associated with attrition from a randomized controlled trial of meaning-centered group psychotherapy for patients with advanced cancer. Psycho-Oncology. 2012; Nov;21(11):1195-204. doi: 10.1002/pon.2013. Epub 2011 Jul 12.
10. McCambridge J, Kalaitzaki E, White IR, Khadjesari Z, Murray E, Linke S et al. Impact of length or relevance of questionnaires on attrition in online trials: randomized controlled

For peer review only - http://bmjopen.bmj.com/site/about/guidelines.xhtml

trial. Journal of Medical Internet Research. 2011 Oct;13(4):e96. e96. Available from, DOI: 10.2196/jmir.1733.

11. Hui D, Glitza I, Chisholm G et al. Attrition rates, reasons, and predictive factors in supportive care and palliative oncology clinical trials. Cancer. 2013:119;5:1098-1105.
12. O'Cathain A, Thomas K, Drabble Set al. The QUAlitative Research in Trials (QUART) study. Health Technology Assessment. 2014;18: No. 38 DOI: 10.3310/hta18380.
13. Noblit GW, Hare RD. Meta-ethnography: synthesizing qualitative studies. 1988. Newbury Park: Sage.
14. Britten N, Campbell R, Pope C et al. Using meta ethnography to synthesise qualitative research: a worked example. J Health Serv Res Policy. 2002;7:4:209–215.
15. Critical Appraisal Skills Programme (2017). CASP (Qualitative Research) Checklist. [online] Available at: http://docs.wixstatic.com/ugd/dded87_25658615020e427da194a325e7773d42.pdf Accessed: July 2017.
16. [illegible]ell R, Pound [illegible], P[illegible] C et a[illegible]valuating m[illegible]ta-ethnography[illegible]a synthes[illegible] of qualitative [illegible]
17. Pound P, Britten N, Morgan M, et al. Resisting medicines: a synthesis of qualitative studies of medicine taking. Soc Sci Med. 2005. 61: 133-155. 10.1016/j.socscimed.2004.11.063.
18. Atkins S, Lewin S, Smith H, et al. Conducting a meta-ethnography of qualitative literature: Lessons learnt. BMC Medical Research Methodology. 2008, 8:21 doi:10.1186/1471-2288-8-21.
19. Schumm K, Skea Z, McKee L, N'Dow J. 'They're doing surgery on two people': a meta-ethnography of the influences on couples' treatment decision making for prostate cancer. Health Expectations. 2010:13, pp.335–349.
20. Black M, Entwistle VA, Bhattacharya S, et al. Vaginal birth after caesarean section: why is uptake so low? Insights from a meta-ethnographic synthesis of women's accounts of their birth choices. BMJ Open 2016;6: e008881. doi:10.1136/ bmjopen-2015-008881.
21. Nicholas J, Proudfoot J, Parker G, et al. "The Ins and Outs of an Online Bipolar Education Program: A Study of Program Attrition."J Med Internet Res 2010 (Dec 19); 12(5):e57.
22. Postel M, de Haan H, ter Huurne E, et al. Effectiveness of a Web-based Intervention for Problem Drinkers and Reasons for Dropout: Randomized Controlled Trial. J Med Internet Res 2010;12(4):e68.
23. Wells A, Palinkas L, Qiu X, et al. Cancer patients' perspectives on discontinuing depression treatment: the "drop out" phenomenon. Patient preference and Adherence. 2011:5;465-470.
24. Johansson O, Michel, T, Andersson G, et al. Experiences of non-adherence to Internet-delivered cognitive behavior therapy: A qualitative study. Internet Interventions. 2015:2;137-142.
25. Sari S, Muller AE, Roessler KK. Exercising alcohol patients don't lack motivation but struggle with structures, emotions and social context – a qualitative dropout study. BMC Family Practice 2017:18;45.
26. Fernandez-Alvarez J, Diaz-Garcia A, Gonzalez-Robles A et al. Dropping out of a transdiagnostic online intervention: A qualitative analysis of clients' experiences. Internet Interventions. 2017:10; 29-38.

For peer review only - http://bmjopen.bmj.com/site/about/guidelines.xhtml

27. Shilling, V, Williamson, P R, Hickey, H, Sowden, E, Smyth, R L and Young, B (2011) Processes in recruitment to randomised controlled trials of medicines for children (RECRUIT): a qualitative study. Project Report. 2011: 15. NIHR Journals Library.
28. Sanders C, Rogers A, Bowen R, et al. Exploring barriers to participation and adoption of telehealth and telecare within the Whole System Demonstrator trial: a qualitative study. BMC Health Services Research. 2012: **12**:220 https://doi.org/10.1186/1472-6963-12-220.
29. Eborall H, Stewart M, Cunningham-Burley S, et al. et al. Accrual and drop out in a primary prevention randomised controlled trial: qualitative study.Trials. 2011: 12:7 http://www.trialsjournal.com/content/12/1/7.
30. Henshall C, Narendran P, Andrews RC et al. Qualitative study of barriers to clinical trial retention in adults with type 1 diabetes. BMJ Open. 2018;8:e022353.
31. Nakash R, Hutton J, Lamb S, et al. Response and non-response to postal questionnaire follow-up in a clinical trial – a qualitative study of the patient's perspective. Journal of Evaluation in Clinical Practice. 2008:14;226-235.
32. Lang H, France E, Williams B et al. The psychological experience of living with head and neck cancer: a systematic review and synthesis. Psychooncology. 2013:22;12:2648-63.
33. France E, Wells M, Lang H et al. Why, When and how to update a meta-ethnography qualitative synthesis. Systematic Reviews. 2016:5:44.
34. Hughes-Morley A, Young B, Hempel R, Russell I, Waheed W, Bower P. What can we learn from trial decliners about improving recruitment? Qualitative study *Trials.*2016;**17**:494.
35. Halpern SD, Karlawish JHT, Berlin JA. The continuing unethical conduct of underpowered clinical trials. JAMA. 2002;288:358–62.
36. Drueke TB, Descamps-Latscha B, Locatelli F. Stopping a medical research project for financial reasons. Nephrol Dial Transplant. 2003;18(10):1982–3.
37. Hughes-Morley A, Young B, Waheed W, Small N, Bower P. Factors affecting recruitment into depression trials: systematic review, meta-synthesis and conceptual framework. J Affect Disord. 2015;172C:274–90.
38. Thompson L, McCabe R. The effect of clinician-patient alliance and communication on treatment adherence in mental health care: a systematic review. BMC Psychiatry. 2012;12:87. doi:10.1186/1471-244X-12-87.
39. Kearney A, Rosala-Hallas A, Bacon N, et al. Reducing attrition: the communication of retention and withdrawal within patient information sheets. Trials. 2017:18;(Suppl 1):P.489.
40. Gamble C, Dudley L, Allam A, et al. Patient and public involvement in the early stages of clinical trial development: a systematic cohort investigation. BMJ Open 2014;4:e005234. doi:10.1136/bmjopen-2014- 005234.
41. Buck D, Gamble C, Dudley L, et al. From plans to actions in patient and public involvement: qualitative study of documented plans and the accounts of researchers and patients sampled from a cohort of clinical trials. BMJ Open 2014;4:e006400. doi:10.1136/bmjopen-2014- 006400.
42. Brueton VC, Tierney J, Stenning S, Harding S, Meredith S, Nazareth I, Rait G. Strategies to improve retention in randomised trials. Cochrane Database of Systematic Reviews 2013, Issue 12. Art. No.: MR000032. DOI: 10.1002/14651858.MR000032.pub2.
43. Donovan J, Rooshenas L, Jepson M et al. Optimising recruitment and informed consent in randomised controlled trials: the development and implementation of the Quintet Recruitment Intervention (QRI). Trials. 2016; 17:283. DOI 10.1186/s13063-016-1391-4.

Authors: Dan Brunsdon; Linda Biesty; Peter Brocklehurst;

For peer review only - http://bmjopen.bmj.com/site/about/guidelines.xhtml

44. Brunsdon D, Biesty L, Brocklehurst et al. What are the most important unanswered research questions in trial retention? A James Lind Alliance Priority Setting Partnership – The PRioRiTy II (Prioritising Retention in Randomised Trials) Study https://doi.org/10.21203/rs.2.439/v1

For peer review only

For peer review only - http://bmjopen.bmj.com/site/about/guidelines.xhtml

S1 Box. Our analytical approach

1) Getting started

This stage involved us generating a research question specific to our area of interest that we believed could be usefully addressed by referring to qualitative research. In our case this was the question of what influences non-retention within clinical trials from the perspectives of trial withdrawers?

2) Describing what is relevant to initial interest

This stage involved making a series of decisions relating to deciding what was relevant to our initial area of interest, deciding on the searching process, inclusion/exclusion decisions and quality assessment. In our case we were interested in any study that reported the use of qualitative methods (for collection and analysis of data) to explore the reasons why individual participants withdraw from clinical trials. We were interested in any reports made by participants themselves or by trial staff, but this had to be specifically in relation to why participants withdraw. We defined withdrawal or non-retention as covering any aspect of attrition recognising that this might cover activities such as cessation of, or withdrawal from the intervention(s), non-attendance at clinic visits, through to non-response to some or all follow up questionnaires etc. We decided that we would exclude studies that did not use qualitative means to collect or analyse their data and also studies reporting findings from trial withdrawers who were not patient participants e.g. GPs in a primary care cluster trial. Following these decisions, a systematic search across a range of databases was conducted with assistance from an information specialist (See S1 Appendix) and all titles and abstracts were screened for inclusion (see S1 and S2 Figures). Applying quality criteria to qualitative research remains a contentious issue and there is no consensus regarding whether and how this should be done (Mays 2000; McEwan 2004). However, one author (ZS) undertook a quality assessment of each of the 11 papers that were identified as being eligible for inclusion in the synthesis. Whilst authors of some qualitative evidence syntheses have chosen to exclude what they deem to be poor quality papers, we made the decision not to exclude any of the identified papers. Although all papers had study aims that were amenable to investigation via qualitative means and all included qualitative data, as a team we deemed some as being richer than others in terms of data and insights (i.e. first and second order constructs). Despite this variation in the overall level of quality, due to the small number of identified studies we considered it more important to retain any relevant findings than disregard based on study quality. In doing so, we would argue that all 11 papers contributed useful elements to the collective whole and enabled us to develop our line of argument in terms of the issues of importance regarding trial non-retention.

3) Reading the studies

At this stage, we aimed to become as familiar as possible with the content of all the identified papers with each author independently reading through all of the data provided and making detailed notes of their observations including identification of preliminary themes. After sharing notes, we met to discuss our findings as a team, comparing and contrasting our preliminary observations etc. We repeated this process for the 3 papers that we identified in our updated database search.

4) Determining how the studies are related

In describing this phase, Noblit and Hare 1988 state that “In doing a synthesis, the various studies must be ‘put together’. This requires determining the relationships between the studies to be

For peer review only - http://bmjopen.bmj.com/site/about/guidelines.xhtml

synthesized. We think it makes sense to create a list of the key metaphors, phrases, ideas, and/or concepts (and their relations) used in each account and to juxtapose them…" During this next stage (which in practice we found very much related to activities undertaken as part of stage 3), data was extracted initially from all 8 papers (retrieved from the 1st database search) using a standard form which summarised the main phrases, themes and ideas, along with, information regarding methods, and any other important information relating to the context of the research (some of this data is illustrated in S1 Table). During this stage, we focussed on both 1st order constructs within included papers (meaning study participant quotations found in the results section of papers) along with 2nd order constructs (meaning the interpretations made by the papers' authors, usually found in the discussion and conclusion sections of papers but also sometimes within the results). Using the standard form, the papers were initially organised in chronological order (but as inductive analysis progressed papers were grouped according to emerging themes) and we focused on the findings, concepts and themes used by the papers' authors generating a list of key categories. This document (along with our other written notes and observations) facilitated discussions at a series of subsequent team meetings and were very useful for consideration of how identified themes from one paper might relate to the others. We added similar data from the additionally identified 3 papers to this form, to allow us to compare and contrast findings with the earlier 8 papers.

5) Translating the studies into one another

Noblit and Hare 1988 state that "In its simplest form, translation involves treating the accounts as analogies: One program is like another except….It also compares both the metaphors or concepts and their interactions in one account with the metaphors and their interactions in the other accounts."

At this key stage (which again in practice we found inter-related to stage 4), following this process, we sought to consider the extent to which themes and concepts seemed common or distinct across the papers. Our initial grouping of 1st and 2nd order constructs across the 8 papers resulted in 14 sub-themes. These were issues/ideas that we each considered important in terms of things that might make people withdraw from trials. During the process of translating themes/concepts from each of the individual studies into those of the others (i.e. comparing and contrasting across studies), following further team discussion these were then grouped and categorised into 5 broad key themes (as it became apparent that some of sub-themes were related or overlapped). We interpreted our 5 key themes as characterising the main considerations and features that appeared to influence non-retention in the trials under investigation (See S2 Table). For the 3 subsequently identified papers, we repeated this stage by comparing and contrasting concepts and their interactions in these 3 accounts with the concepts identified in the original 8 accounts– in essence comparing for 'fit' and checking for any additional themes (Lang 2013). During this process, we were confident that concepts identified in the later 3 papers supported and complemented our originally identified 5 key themes with no new themes emerging.

6) Synthesizing the translations

Noblit and Hare 1988 state that "Synthesis refers to making a whole into something more than the parts alone imply." For our synthesis, what we were attempting to do at this stage was to move towards an explanatory analysis. We considered and discussed how the various translations compared in an attempt to develop a more nuanced and collective understanding of factors influencing trial non-retention (in doing so, developing our 'line of argument' synthesis). As before, we did this through a process of reflection and team discussions, in an attempt to produce overarching insights into the factors that appear to influence non-retention.

For peer review only - http://bmjopen.bmj.com/site/about/guidelines.xhtml

7) Expressing the synthesis

As is common with other meta-ethnographies we sought to express our collective insights in both textual and diagrammatic format within our paper. In doing so, we expressed our synthesis both within our paper as our 'line of argument' (with supportive illustrative data from across the studies) and also as a conceptual diagram (see S3 Figure).

For peer review only

S1 Table Characteristics of included studies

REF	COUNTRY	AIM	CONDITIONS OF FOCUS	PARTICIPANT CHARACTERISTICS	TRIAL COMPARATORS	ATTRITION BEHAVIOUR	DATA COLLECTION METHODS
Nakash et al 2007 [31]	UK	To examine factors affecting response and non-response from the clinical trial participant's perspective.	Severe ankle sprains	8 non-retainers. Age and gender of non-retainers unclear although of the 22 included in this study, 11 were male; 11 female, aged from 16 to 62 yrs (mean age 34 yrs). A purposive sample was sought to represent the diversity of trial participants in age, sex, level of education, occupation and type of ankle support.	Different mechanical supports	Non-response to postal questionnaire follow-up. 8 had not responded to at least one of their follow up questionnaires.	Semi-structured interviews
Nicholas et al 2010 [21]	Australia	To identify participants' reasons for non-adherence to, and attrition from the online intervention	Newly diagnosed bipolar disorder	39 non-retainers. 22 female; 17 male. 20 were aged less than 30 yrs, 14 were married, 29 were tertiary educated and 24 in full time employment. Participants from all 3 study groups were interviewed, 16 from the unsupported intervention group (BEP), 9 from the supported BEP intervention group (BEP+IS), and 14 from the minimal	1 of 2 active interventions (online psycho-education program either alone or with email support from informed supporters) or an attention control condition (online information about bipolar disorder presented in text as bullet points). Both active interventions and control contained 'workbook' activities.	Cessation of therapy sessions and/or non-completion of some or all intervention workbooks; non-completion of some or all control workbooks.	Semi-structured interviews

For peer review only

S1 Table Characteristics of included studies

				information control group.			
Postel et al 2010 [22]	Netherlands	To evaluate an e-therapy program with active therapeutic involvement for problem drinkers. Reasons for drop out were also investigated via a 'dropout' questionnaire consisting mainly of open questions.	Problem drinking	40 non-retainers. No gender or age details provided specifically for non-retainers, although 53.8 % of trial participants were female, mean age 45.3 yrs. Authors also state that in the control group more non-responders than responders were male.	3 month e-therapy programme (consisting of a structured 2 part online treatment programme with asynchronous therapist contact via the internet only) or a waiting list control group (receiving 'no reply' email messages once every 2 weeks). Participants completed online self-report questionnaires at baseline and at 3 months follow up (control group) or at posttreatment, which was approximately 3 months (e therapy group). Weekly alcohol consumption was assessed by a 7 day retrospective drinking diary.	Non-completion of treatment sessions and/or follow-up questionnaires. Non-retention was defined as anyone who did not complete the 3 month assessment. Dropouts in the e therapy group did not complete all 12 treatment sessions: 9 assignments and 3 assessments.	A 'dropout' questionnaire consisting mainly of open questions.
Eborall et al 2011 [30]	UK	To explore people's explanations for declining to participate in the trial, or, having begun the trial, stopping the trial medication	Asymptomatic atherosclerosis	17 non-retainers. No gender or age details provided specifically for non-retainers (as opposed to those who declined to consent), but states that total sample (n=28) had a mean age of 65.2 yrs; 19 were female; wide	Aspirin (100mg daily) or placebo for a mean duration of 8.2 yrs.	Cessation of trial medication (unclear whether active medication or placebo). Non-adherence with study medication throughout the trial was 40%; 15% took their medication for less than 6 months.	Semi-structured interviews (n=11) and one focus group (n=6)

For peer review only

S1 Table Characteristics of included studies

				range of socioeconomic backgrounds.			
Wells et al 2011 [23]	USA	To explore low-income, minority cancer patient perspectives about not adhering or dropping out of depression treatment.	Depression and cancer	20 non-retainers. No gender or age details provided specifically for non-retainers included in the qualitative study but trial non-retainers were described as predominantly female, foreign born, unmarried, unemployed, and older than 50 yrs.	Intervention or usual care. Intervention was an individualised stepped care depression programme provided by a cancer depression clinical specialist in collaboration with a study psychiatrist. Patients in the intervention group were offered antidepressant medication and/or problem solving treatment (PST).	Cessation of problem solving treatment sessions and/or anti-depressant medication.<br><br>PST dropouts were defined as patients who had fewer than 4 PST sessions. PST dropouts included those who initially agreed to be randomised to the intervention, but thereafter had either verbally declined treatment or did not show up for the therapy appointments. This included patients who had refused some sessions, but agreed to remain in the study for outcome interviews.<br><br>Patients receiving antidepressant medication were dropouts if they discontinued treatment within 30 days.	Semi-structured interviews
Shilling et al 2011 [27]	UK	To investigate recruitment processes across a range of clinical trials and from the perspective of parents, young people and practitioners to identify strategies to	Trial 1: Neuro-development disorders<br><br>Trial 2: Osteopenia	3 non-retainers from 2 of the 4 included trials. In trial 1, participants were 11-14 yrs; In trial 2, participants were 4-18 yrs (although demographic details of non-retainers unclear).	Trial 1: Melatonin versus placebo (over 12 week treatment period). Families made 3 additional hospital visits, and received 4 home visits by the research nurse and 3 telephone calls.	Cessation of trial medication (unclear whether active medication or placebo)	Semi-structured interviews (with the young people and/or their parents).

For peer review only

S1 Table Characteristics of included studies

		improve recruitment and its conduct across a spectrum of trials of medicines for children.<br><br>NB: This was a monograph that included the reporting of a range of trials, two of which reported data on non-retainers and were included in this meta-ethnography.			Trial 2: bisphosphonate risedronate or vitamin D analogue 1 – alphahydroxychol ecalciferol versus placebo (1 yr treatment period). Young people were seen 7 times over the year. This was timed to coincide with routine clinic visits where possible. Blood samples were also taken (same time as routine visits) and they gave regular urine samples and had 3 x-ray scans and 2 bone radiographs.		
Sanders 2012 [28]	UK	To explore barriers to participation and adoption of tele-health and tele-care from the perspective of people who declined to participate or withdrew from the trial	Diabetes, COPD, heart failure, or social care needs	3 non-retainers (all in the intervention arm), 1 female (diabetes); 2 male (1 x COPD; 1 x diabetes + heart and lung problems. 73-85 yrs.	The RCT was a cluster design with GP practices being randomised to receive access to telehealth or telecare for their populations. Participants randomised to the control arm were offered telehealth or telecare at the end of the 12mth trial.<br><br>Tele-health equipment included a monitor unit via which recordings from peripheral devices were uploaded to a monitoring centre. The monitoring centres prioritised and	Cessation of use of telehealth equipment or tele care devices (defined as withdrawing from the trial after joining the intervention arm).	Semi-structured interviews

For peer review only

S1 Table Characteristics of included studies

					tailored response according to need based on the information received.<br><br>Telecare interventions also varied according to assessed need but included various sensors to detect gas, water overflow, falls and movement around the property. Such sensors would trigger alarms direct to a monitoring centre if anything abnormal was detected, allowing emergency intervention.		
Johansson 2015 [24]	Sweden	To explore participants' experiences of non-adherence to internet-delivered psychological treatment	Generalised anxiety disorder	7 non-retainers, 6 female; 1 male; mean age 39.3 yrs	Intervention consisted of internet delivered psychological treatment, with weekly support from a licensed clinical psychologist. The treatment consisted of 8 weekly self-help modules of text, audio and illustrations, averaging 21 pages per module. All modules contained a homework assignment that needed to be answered and sent to the guiding therapist.	Non-adherence to internet delivered psychological treatment.<br><br>Non-adherence was defined as completing at least 1 and no more than 7 treatment modules (out of 8 in total).	Semi-structured interviews

For peer review only

S1 Table Characteristics of included studies

					No details re. control group given.		
Sari 2017 [25]	Denmark	To investigate perceived barriers to participate in an exercise intervention among alcohol use disorder patients, who dropped out from the Healthy Lifestyle Study	Alcohol Use Disorder	17 non-retainers, 4 female; 13 male; age 30-68 yrs. Nine were allocated to the group intervention, 7 were allocated to the individual intervention and one were allocated to the control group.	Treatment as-usual or one of two 6-month interventions that were selected on the basis of existing evidence-based studies. In the first intervention group, participants exercised individually after receiving basic instructions and a training program for home use. In the second intervention group, several patients exercised together with two instructors in 60-min training sessions twice a week. Running was the specific exercise form for both groups in the study.	Drop out was indicated when participants directly reported to project personnel or when they ceded participation without contact. Their length of participation before dropout varied from 2 days to 12 weeks.	Semi-structured interviews
Fernandez-Alvarez 2017 [26]	Spain	To conduct a qualitative analysis of the subjective experience of a sample of patients who dropped out of a transdiagnostic Internet based treatment for emotional disorders.	Obsessive-compulsive disorder; Agoraphobia;Social Anxiety Disorder; Major Depressive Disorder; Generalized Anxiety Disorder	10 non-retainers, 8 female; 2 men, age 21-59 yrs. Unclear whether control or treatment arm.	The protocol consists of 12 modules, and participants are encouraged to complete one module per week. Two RCTs are being conducted using the protocol. The purpose of one of the RCTs is to analyze the effectiveness of a transdiagnostic IBT compared to treatment as usual as provided in the Spanish public mental	Individuals who dropped out after completing a minimum of 3 modules.	Semi-structured interviews

S1 Table Characteristics of included studies

					health care system. Both treatment protocols are web-based, self-administered treatments with minimum contact/support from a therapist that consists in a weekly phone call lasting 5 to 10 min to each participant. A non-human support is delivered through two weekly mobile phone text messages that are automatically sent and aim to remind the participants of the importance of reviewing the modules as well as doing the homework tasks.		
Henshall 2018 [29]	UK	To determine the overall experiences of newly diagnosed adults with T1D in an exercise study, and to understand issues that influence the retention of trial participants in such studies	Newly diagnosed Type 1 diabetes	4 non-retainers; 2 female; 2 male age 19 - 55 yrs (2 in intervention arm/2 in control arm)	Conventional treatment or exercise, stratified on beta cell function and fitness. The exercise group were encouraged to increase their level of activity to a minimum of 150 minutes of moderate to vigorous intensity exercise per week, aiming for 240 minutes per week of exercise for 12 months.	No specific details given other than 'those who withdrew before the end of the study'	Semi-structured interviews

For peer review only

S1 Table Characteristics of included studies

For peer review only

S2 Table Key themes of influence on decisions to withdraw from trial participation, with corresponding example data.

Theme	Exemplary quote
**Perceptions of current health state in relation to specific aspects of the trial**	*"A very short while after doing the program I fell into another episode, a depressive episode, and pretty much stopped doing everything, the program included"[21, male, 18-29 yrs, BEP+IS group]*<br><br>*"I found it quite confronting, and reading the information made me feel uncomfortable, thinking that these issues related to me – I preferred the ostrich approach" [21, male, 40-49 yrs, BEP group]*<br><br>**This study demonstrates that reasons…were often explained in terms of potential threats to existing self-care, independence, the majority of respondents in this study depicted themselves as too healthy and too independent for the interventions to be of value [28, telehealth and telecare interventions in aging populations]**
**The 'fit' of aspects of the trial with individual preferences for care and support**	**Two respondents who withdrew from the trial described how the service changes they experienced caused additional stress. For example, one woman said she** *'did not want to be a nurse' …***and she was much happier to have returned to a regular appointment (fortnightly) with the community matron. Another man described the good care he received prior to joining the trial, but how he was subsequently discharged from the specialist professionals who had been involved in his care…..he described his main problems as** *'complex problems with my heart and breathing,'* **and that the faulty recordings and changes in service provision were causing him great stress [28, telehealth and telecare interventions in aging populations]**<br><br>**Some participants changed to other treatment formats after terminating the Internet-delivered therapy. One participant explained that she had never prioritised her own personal development and that an individual therapy consisting of face to face meetings was needed to get away from home and focus on the therapy [24, internet-delivered psychological treatment for people with generalised anxiety disorder]**
**The compatibility of aspects of the trial with individual capabilities**	**Twelve of the 20 patients interviewed acknowledged that they had dropped out of treatment, citing several reasons and circumstances related to dropping out of treatment. These**

For peer review only

	**included…cultural (which included language communication problems) [23, problem solving treatment sessions and/or anti-depressant medication for people with depression and cancer]**<br><br>**Difficulty with literacy [31, Different mechanical supports for people with severe ankle sprains]**<br><br>**One critique was that information was difficult to understand because the content was perceived as complex and abstract. In some cases the participants felt unintelligent for their inability to understand [24, internet-delivered psychological treatment for people with generalised anxiety disorder]**
**Concerns about or experiences of trial medication**	**One mother's discovery, via an internet search done by the child's father, that the trial drug was unlicensed for children had left her concerned about the safety of the trial and she subsequently withdrew her child from MENDS [trial] [27]**<br><br>*"I really felt I'd got gall bladder trouble again because [the pain] was from here right through into me kidneys and really severe. So I went to my GP, and she just checked round and said straight away, 'don't take anymore, and ring [the trial] and tell them'"[30, Aspirin for people with Asymptomatic atherosclerosis]*
**Considerations around extent to which trial participation could be appropriately accommodated into broader life circumstances**	**The participants' statements regarding non-adhering showed an incompatible relationship between the length of the weekly text modules and factors or conditions in the personal life of the participants [24, internet-delivered psychological treatment for people with generalised anxiety disorder]**<br><br>*"…as a student you read so much already. I felt like I couldn't muster more energy or more time to spend by the computer and to read 10 or 20 more pages and also answer questions. It felt as if you were inclined to have a very structured life already to handle that" [24, internet-delivered psychological treatment for people with generalised anxiety disorder]*<br><br>**These were reasons such as pregnancy, exams, or work commitments which participants felt were the cause of their non-response [31 Different mechanical supports for people with severe ankle sprains]**

For peer review only

	**We could establish that in the e-therapy group 11 participants dropped out because of personal reasons unrelated to the e-therapy program or the study (eg, ill family member) [22]**

Primary study participant quotes (1st order constructs) are displayed in italics and primary study author interpretations (2nd order constructs) are presented in bold. Study references and details of participants/interventions where available have been added to the end of exemplar quotes.

For peer review only

S3 Table

	Inter-relationship between self and trial processes/procedures													
**Themes**	**1. Perceptions of current health state in relation to specific aspects of the trial**		**2. The 'fit of aspects of the trial with individual preferences for care and support**						**3. The compatibility of aspects of trial processes with individual capabilities**		**4. Concerns about or experiences of trial medication**			**5. Considerations around the extent to which trial participation could be appropriately accommodated into individuals' broader lives**
14 Sub-themes	Perception of being too well	Perception of being too ill	Not individual /tailored/ personalised enough	Too basic	Inflexible	Too technical	Too intensive	Too stressful	Not tailored to individual capabilities	Cognitively not pitched at the individual	Medication not necessary	Potentially dangerous	Contraindications or side effects	Aspects of life getting in the way
Nakash 2008	*									*				*
Nicholas 2010	*	*	*	*	*									*
Postel 2010	*						*	*						*
Eborall 2011	*										*		*	*
Wells 2011	*	*								*				
Shilling 2011												*	*	
Sanders 2012	*			*	*	*								
Johansson 2015	*	*	*		*				*	*				*
Sari 2017	*						*							*
Fernandez-Alvarez 2017		*	*	*										*
Henshall 2018		*												*

*Denotes presence of themes across papers

For peer review only

PRISMA Flow Diagram: Retention Review

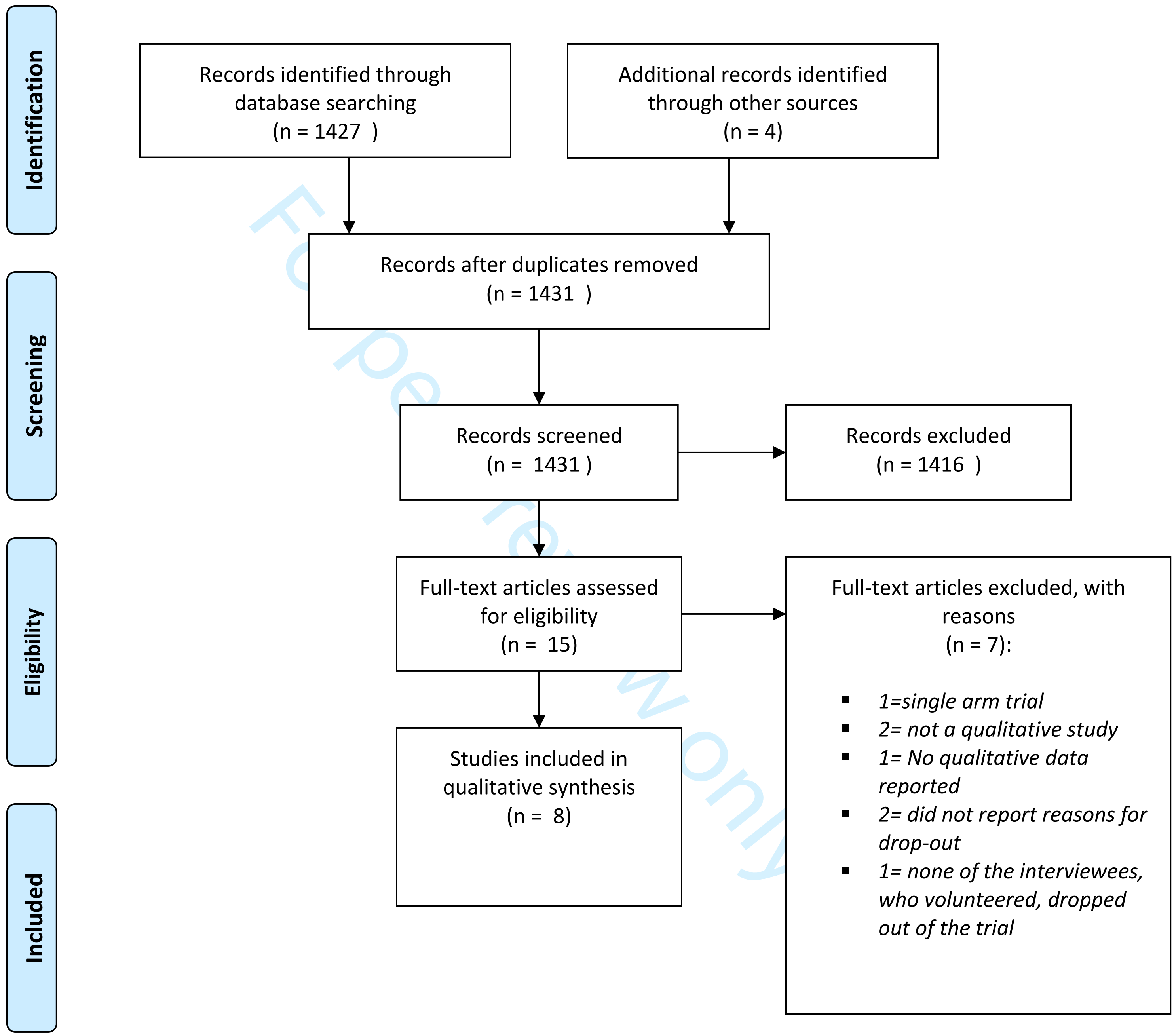

PRISMA Flow Diagram: Retention Review (search update)

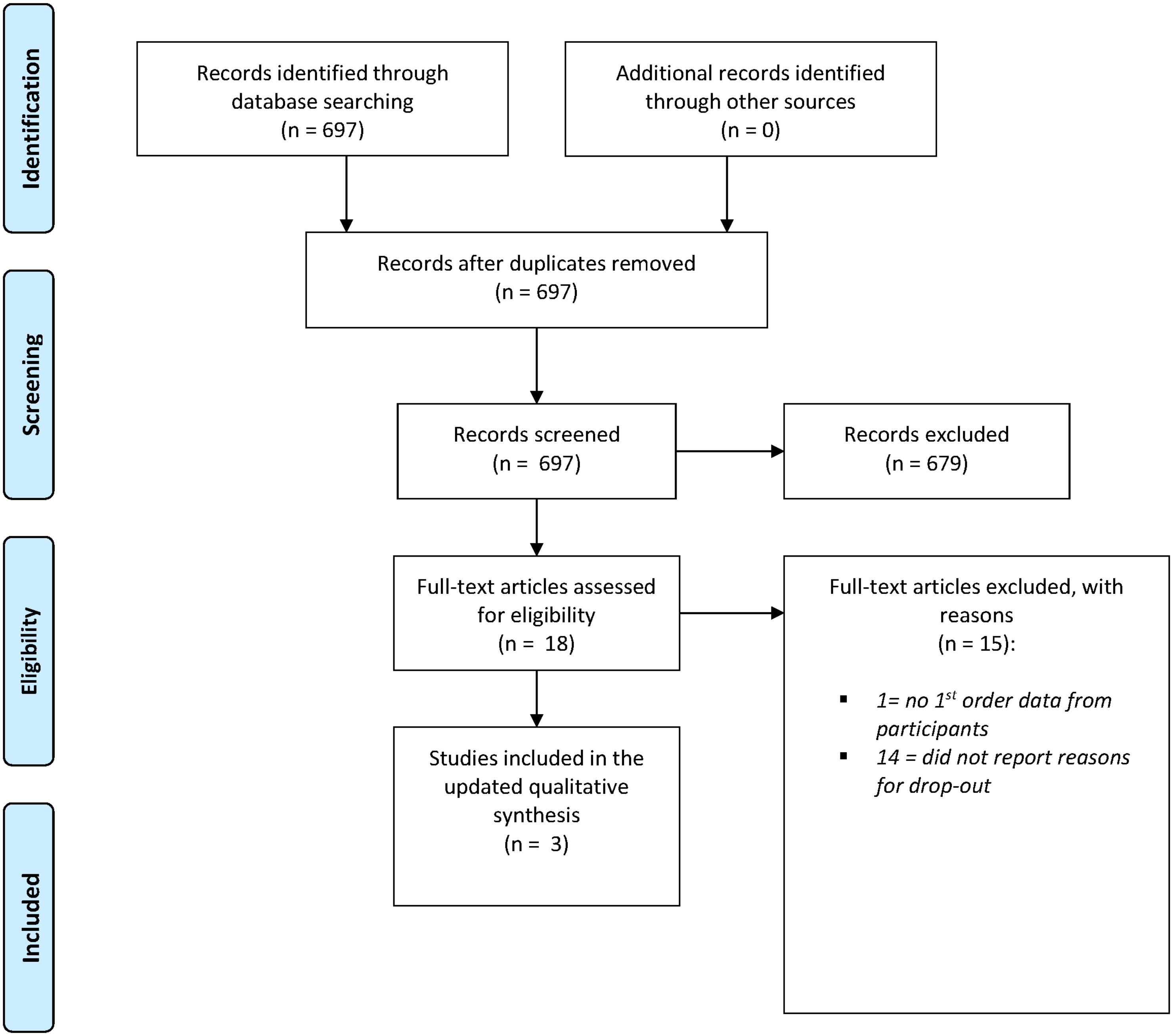

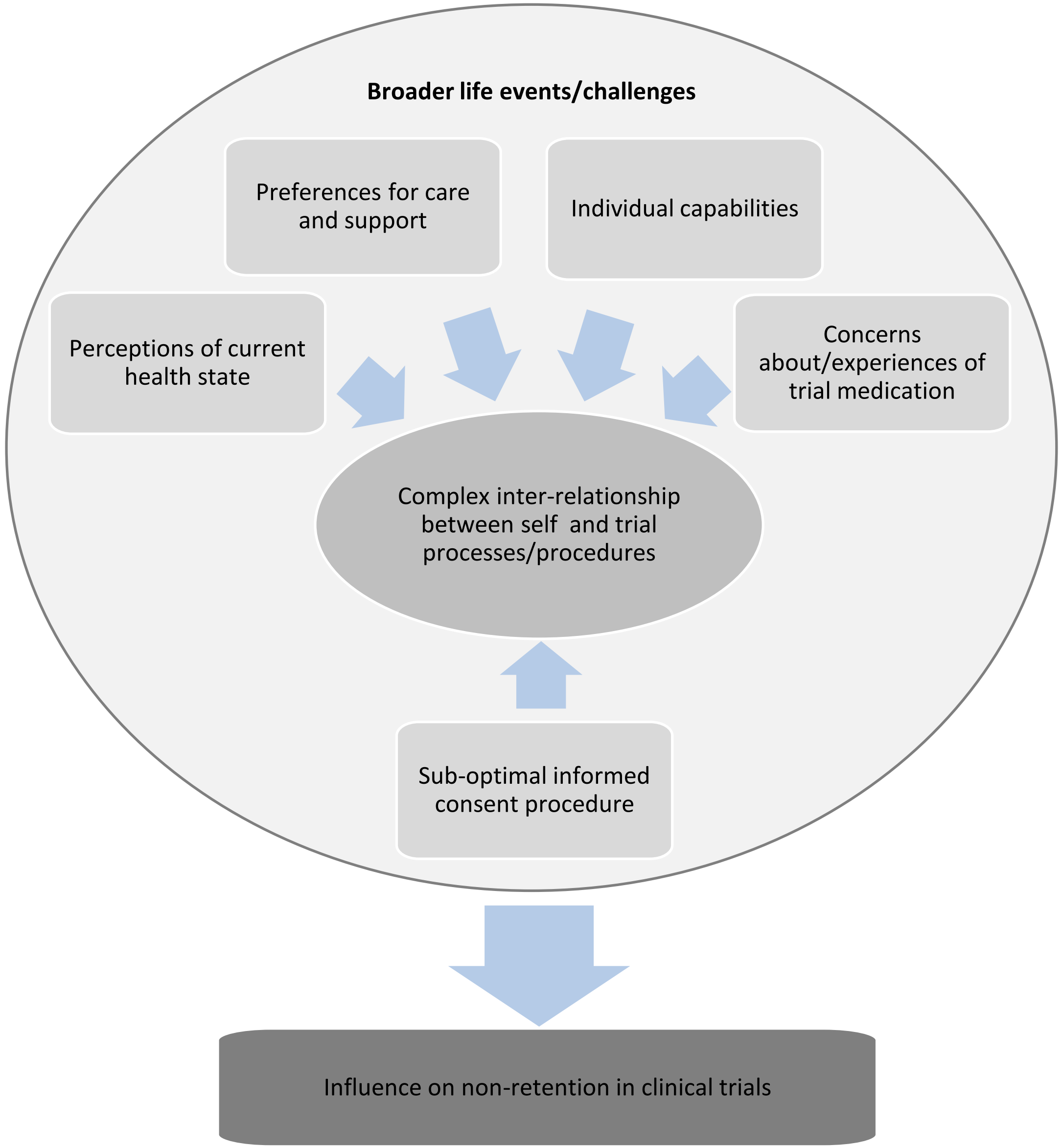
Broader life events/challenges
Preferences for care and support
Individual capabilities
Perceptions of current health state
Concerns about/experiences of trial medication
Complex inter-relationship between self and trial processes/procedures
Sub-optimal informed consent procedure
Influence on non-retention in clinical trials

Search strategies

July 2018 Update

Database: Embase <1996 to 2018 Week 30>, Ovid MEDLINE(R) and Epub Ahead of Print, In-Process & Other Non-Indexed Citations, and Daily <1946 to July 23, 2018>
Search run 24th July 2018
URL: https://ovidsp.ovid.com/

1 exp clinical trial/ (1980088)
2 randomi?ed control$ trial$.tw. (336149)
3 randomi?ed controlled trial?.kw. (23247)
4 clinical trial?.tw,kw. (729051)
5 controlled trial?.tw,kw. (429924)
6 controlled clinical trial?.tw,kw. (50908)
7 pragmatic trial?.tw,kw. (2136)
8 complex intervention?.tw,kw. (4998)
9 or/1-8 (2639576)
10 qualitative research/ (94827)
11 qualitative research.tw,kw. (34500)
12 (qualitative adj3 method$).tw. (53149)
13 (qualitative method? or qualitative methodology).kw. (2740)
14 (qualitative adj3 stud$).tw. (95856)
15 qualitative study.kw. (2597)
16 focus groups/ use ppez (24850)
17 focus group?.tw,kw. (82443)
18 grounded theory/ (6039)
19 grounded theory.tw,kw. (21174)
20 narrative analys?s.tw,kw. (2147)
21 process evaluation.tw,kw. (5815)
22 mixed method?.tw,kw. (30409)
23 mixed method$.mp. (31273)
24 mixed methodology.tw,kw. (711)
25 (in depth adj4 interview$).tw. (41493)
26 in depth interview?.kw. (201)
27 ((semi structured or semistructured) adj5 interview$).tw. (89673)
28 semi structured interview?.kw. (277)

For peer review only

29 qualitative interview$.tw. (18002)
30 qualitative interview?.kw. (432)
31 (interview$ and theme$).tw. (61425)
32 interview?.kw. (6730)
33 (interview$ and audio recorded).tw. (5252)
34 qualitative case stud$.tw. (2011)
35 descriptive case stud$.tw. (475)
36 qualitative case study.kw. (25)
37 descriptive case study.kw. (0)
38 qualitative exploration.tw,kw. (1994)
39 qualitative evaluation.tw,kw. (6296)
40 qualitative intervention.tw,kw. (25)
41 qualitative approach.tw,kw. (7887)
42 qualitative inquiry.tw,kw. (1197)
43 qualitativ$ analys$.tw. (31755)
44 qualitative analysis.kw. (1269)
45 (qualitative adj3 data).tw. (34567)
46 qualitative data.kw. (152)
47 discourse analysis.tw,kw. (3342)
48 discursive.tw,kw. (3245)
49 phenomenological.tw,kw. (29346)
50 thematic analysis.tw,kw. (27166)
51 ethnograph$.tw. (18207)
52 ethnography.kw. (1849)
53 action research.tw,kw. (7525)
54 ethno?methodology.tw,kw. (150)
55 social construction.tw,kw. (1643)
56 or/10-55 (426531)
57 phenomenological characteristics.tw,kw. (242)
58 phenomenological model.tw,kw. (1806)
59 action research arm test.tw,kw. (1065)
60 protocol.ti. (79269)
61 or/57-60 (82341)
62 56 not 61 (418963)
63 9 and 62 (25059)
64 Patient Dropout/ use ppez (7679)
65 Patient Dropouts/ use emef (433)

For peer review only

66 Patient Recruitment/ use ppez (59391)

67 Research Subjects/ use emef (4918)

68 patient recruitment.kw. (179)

69 attrition.kw. (1451)

70 patient retention.kw. (32)

71 ((recruit$ or participat$ or take part or dropout$ or drop$ out$ or withdr?wl$ or barrier$ or retention or response$ or respond$ or attrition) adj10 trial?).tw. (134886)

72 or/64-71 (204550)

73 63 and 72 (3670)

74 limit 73 to english language (3640)

75 74 not abstract.pt. (3020)

76 exp animals/ not human/ (7165523)

77 exp nonhuman/ not humans/ (3518640)

78 75 not (76 or 77) (3011)

79 limit 78 to yr="2010 -Current" (2280)

80 remove duplicates from 79 (1325)

****

Database: PsycINFO <2002 to July Week 3 2018>

Search run 24th July 2018

URL: https://ovidsp.ovid.com/

1 clinical trials/ (10606)

2 randomi?ed control$ trial$.tw. (26600)

3 clinical trial?.tw. (27527)

4 controlled trial?.tw. (31463)

5 controlled clinical trial?.tw. (2319)

6 pragmatic trial?.tw. (191)

7 complex intervention?.tw. (624)

8 or/1-7 (58183)

9 qualitative research/ (7513)

10 qualitative research.tw. (17348)

11 (qualitative adj3 method$).tw. (21172)

12 (qualitative adj3 stud$).tw. (46815)

13 focus group?.tw. (27999)

14 grounded theory/ (3229)

15 grounded theory.tw. (12698)

For peer review only

16 narrative analys?s.tw. (1966)
17 process evaluation.tw. (1167)
18 mixed method?.tw. (18416)
19 mixed methodology.tw. (731)
20 (in depth adj4 interview$).tw. (21003)
21 ((semi structured or semistructured) adj5 interview$).tw. (34163)
22 qualitative interview$.tw. (7708)
23 (interview$ and theme$).tw. (33368)
24 interview?.kw. (0)
25 (interview$ and audio recorded).tw. (1253)
26 qualitative case stud$.tw. (4333)
27 descriptive case stud$.tw. (565)
28 qualitative exploration.tw. (944)
29 qualitative evaluation.tw. (751)
30 qualitative intervention.tw. (9)
31 qualitative approach.tw. (3312)
32 qualitative inquiry.tw. (1457)
33 qualitativ$ analys$.tw. (10100)
34 (qualitative adj3 data).tw. (17288)
35 discourse analysis/ (6373)
36 discursive.tw,kw. (7705)
37 phenomenological.tw. (20981)
38 thematic analysis.tw. (9031)
39 ethnograph$.tw. (21222)
40 action research.tw. (6521)
41 ethno?methodology.tw. (369)
42 social construction.tw. (2816)
43 or/9-42 (211086)
44 phenomenological characteristics.tw. (129)
45 phenomenological model.tw. (123)
46 action research arm test.tw. (110)
47 protocol.ti. (2319)
48 or/44-47 (2678)
49 43 not 48 (210507)
50 experimental attrition/ (307)
51 experimental recruitment/ (96)
52 experimental subjects/ (2407)

For peer review only

53 dropouts/ (348)

54 ((recruit$ or participat$ or take part or dropout$ or drop$ out$ or withdr?wl$ or barrier$ or retention or response$ or respond$ or attrition) adj10 trial?).tw. (13610)

55 or/50-54 (16312)

56 8 and 49 and 55 (484)

57 exp animals/ not human/ (179236)

58 56 not 57 (484)

59 limit 58 to (english language and yr="2010 -Current") (339)

****

Cochrane Central Register of Controlled Trials : Issue 7, 2018

Search run 25th July 2018

URL: http://www.cochranelibrary.com/

#1 MeSH descriptor: [Qualitative Research] this term only

#2 qualitative NEXT research:ti,ab,kw or qualitative NEXT method:ti,ab,kw or qualitative NEXT study:ti,ab,kw (Word variations have been searched)

#3 MeSH descriptor: [Focus Groups] this term only

#4 MeSH descriptor: [Grounded Theory] this term only

#5 mixed NEXT method:ti,ab,kw or narrative NEXT analysis:ti,ab,kw (Word variations have been searched)

#6 interview:ti,ab,kw

#7 qualitative case study:ti,ab,kw or descriptive case study:ti,ab,kw (Word variations have been searched)

#8 qualitative NEXT exploration:ti,ab,kw or qualitative NEXT evaluation:ti,ab,kw or qualitative intervention:ti,ab,kw or qualitative approach:ti,ab,kw or qualitative analysis: ti,ab,kw (Word variations have been searched)

#9 qualitative data:ti,ab,kw (Word variations have been searched)

#10 discourse analysis:ti,ab,kw or discursive:ti,ab,kw (Word variations have been searched)

#11 social construction:ti,ab,kw or action research:ti,ab,kw or ethnography:ti,ab,kw or thematic analysis:ti,ab,kw or phenomenological:ti,ab,kw (Word variations have been searched)

#12 #1 or #2 or #3 or #4 or #5 or #6 or #7 or #8 or #9 or #10 or #11

#13 MeSH descriptor: [Patient Dropouts] this term only

#14 MeSH descriptor: [Patient Selection] this term only

#15 ((recruit$ or participat$ or take part or dropout$ or drop$ out$ or withdr?wl$ or barrier$ or retention or response$ or respond$ or attrition) near/10 trial?):ti,ab,kw (Word variations have been searched)

For peer review only - http://bmjopen.bmj.com/site/about/guidelines.xhtml

For peer review only

#16 #13 or #14 or #15

#17 #12 and #16 Publication Year from 2010 to 2018, in Trials

#18 abstract:pt (Word variations have been searched)

#19 #17 not #18 (385)

Social Sciences Citation Index

Search run: 24th July 2018

Web of Knowledge: URL http://wok.mimas.ac.uk/

For peer review only

#32 382 #21 AND #24 AND #31 Refined by: DOCUMENT TYPES: (ARTICLE OR REVIEW) Indexes=SSCI Timespan=2010-2018

31 12,293 #25 OR #26 OR #27 OR #28 OR #29 OR #30

30 1,646 (TS=patient dropout) AND LANGUAGE: (English)

29 1,311 (TS=patient attrition) AND LANGUAGE: (English)

28 2,040 (TS=patient retention) AND LANGUAGE: (English)

27 4761 (TS=((withdraw$ or barrier$ or retention or response$ or respond$ or attrition) NEAR/10 trial?)) AND LANGUAGE: (English)

26 136 (TS=(("take part" or dropout$ or "drop$ out") NEAR/10 trial?)) AND LANGUAGE: (English)

25 220 (TS=((recruit$ or participat$) NEAR/10 trial?)) AND LANGUAGE: (English)

24 42,063 #23 OR #22

23 42,671 (TS=randomised controlled trial) AND LANGUAGE: (English)

22 42,671 (TS=randomized controlled trial) AND LANGUAGE: (English)

21 148,316 #20 OR #19 OR #18 OR #17 OR #16 OR #15 OR #14 OR #13 OR #12 OR #11 OR #10 OR #9 OR #8 OR #7 OR #6 OR #5 OR #4 OR #3 OR #2 OR #1

20 7,765 (TS=thematic analysis) AND LANGUAGE: (English)

19 16,672 (TS=action research) AND LANGUAGE: (English)

18 7,436 (TS=social construction) AND LANGUAGE: (English)

17 5,371 (TS=discursive) AND LANGUAGE: (English)

1611,763 (TS=discourse analysis) AND LANGUAGE: (English)

15 6,704 (TS=(qualitative near/1 data)) AND LANGUAGE: (English)

14 8,581 (TS=(qualitative near/1 analysis)) AND LANGUAGE: (English)

13 3,122 (TS=(qualitative near/1 approach)) AND LANGUAGE: (English)

12 122 (TS=(qualitative near/1 intervention)) AND LANGUAGE: (English)

11 688 (TS=(qualitative near/1 evaluation)) AND LANGUAGE: (English)

10 529 (TS=(qualitative near/1 exploration)) AND LANGUAGE: (English)

For peer review only - http://bmjopen.bmj.com/site/about/guidelines.xhtml

9 40,423 (TS=(in depth interview* or semi structured interview* or qualitative interview*)) AND LANGUAGE: (English)

8 7,414 (TS=narrative analysis) AND LANGUAGE: (English)

7 19,879 (TS=mixed method*) AND LANGUAGE: (English)

6 8,541 (TS=grounded theory) AND LANGUAGE: (English)

5 40,298 (TS=focus group*) AND LANGUAGE: (English)

4 2,062 (TS= (qualitative NEAR/1 studies)) AND LANGUAGE: (English)

#3 15,953 ((TS= (qualitative NEAR/1 study))) AND LANGUAGE: (English)

2 8,468 ((TS= (qualitative NEAR/1 method*))) AND LANGUAGE: (English)

1 12,460 (TS= (qualitative NEAR/1 research)) AND LANGUAGE: (English)

Cumulative Index of Nursing & Allied Health Literature

Search run 25th July 2018

URL: http://search.ebscohost.com/

S18 S7 AND S16 Limiters - Published Date: 20100101-20181231 Narrow by Language: - English (278)

S17 S7 AND S16

S16 S8 OR S9 OR S10 OR S11 OR S12 OR S13 OR S14 OR S15

S15 TX discourse analysis OR TX discursive OR TX thematic analysis OR TX ethnography OR TX action research OR TX phenomenological

S14 TX qualitative exploration OR TX qualitative evaluation OR TX qualitative intervention* OR TX qualitative approach OR TX qualitative analysis OR TX qualitative data

S13 TX mixed method* OR TX semi structured interview* OR TX in depth interview*

S12 TX focus group* OR TX grounded theory OR TX narrative analysis

S11 TX qualitative n3 research OR TX qualitative n3 method* OR TX qualitative n3 study

S10 (MH "Focus Groups")

S9 (MH "Semi-Structured Interview") OR (MH "Structured Interview") OR (MH "Narratives")

S8 (MH "Qualitative Studies+")

S7 S3 AND S6

S6 S4 OR S5

S5 TX ((recruit$ or participat$ or take part or dropout$ or drop$ out$ or withdr?wl$ or barrier$ or retention or response$ or respond$ or attrition) N10 trial?)

S4 (MH "Research Subjects+")

S3 S1 OR S2

For peer review only - http://bmjopen.bmj.com/site/about/guidelines.xhtml

S2 TX ranndomized or randomised or trial*

S1 (MH "Clinical Trials+")

Applied Social Sciences Index and Abstracts

Search run 25th July 2018

http://search.proquest.com/assia/

S8 S5 and S6Limits applied Language:English PY: 2010-2018 (373)

S7 S5 and S6

S6 (recruit* N/10 trial?) OR (participat* N/10 trial? OR "take part" N/10 trial?) OR (dropout* N/10 trial? OR drop* our* N/10 trial?) OR (withdraw* N/10 trial* OR barrier* N/10 trial?) OR (retention N/10 trial? OR response* N/10 trial?) OR (respond* N/10 trial? OR attrition N/10 trial?)

S5 S3 and S4

S4 qualitative OR (focus group* OR interview*) OR (mixed method* OR ethnography) OR (phenomenological OR discourse analysis) OR discursive

S3 S1 or S2

S2 randomized OR randomised

S1 SU.EXACT("Clustor randomized trials") OR SU.EXACT("Clinical randomized controlled trials") OR SU.EXACT("Single blind randomized controlled trials") OR SU.EXACT("Cluster randomized controlled trials") OR SU.EXACT("Randomized controlled trials") OR SU.EXACT("Double blind randomized trials") OR SU.EXACT("Prospective controlled trials") OR SU.EXACT("Double blind randomized controlled trials") OR SU.EXACT("Clinical trials")

For peer review only

For peer review only - http://bmjopen.bmj.com/site/about/guidelines.xhtml

	Nakash 2008	Nicholas 2010	Postel 2010	Eborall 2011	Wells 2011	Shilling 2011	Sanders 2012	Johannson 2015	Sari 2017	Fernandez-Alvarez 2017	Henshall 2018
Was there a clear statement of the aims of the research?	Yes	Yes	Yes	No – not explicitly in abstract or background	Yes	Yes	Yes	Yes	Yes	Yes	Yes
Is a qualitative methodology appropriate?	Yes	Yes	This reported some qualitative data from open ended questionnaire response options	Yes	Yes	Yes	Yes	Yes	Yes	Yes	Yes
Was the research design appropriate to address the aims of the research?	Yes	Yes	Unclear	Yes	Yes	Yes	Yes	Yes	Yes	Yes	Yes
Was the recruitment strategy appropriate to the aims of the research?	Yes	Yes	Yes	Yes	Yes	Yes	Yes	Yes	Yes	Yes	Yes
Was the data collected in a way that addressed the research issue?	Yes	Yes	Unclear	Yes	Yes	Yes	Yes	Yes	Yes	Yes	Yes
Has the relationship between researcher and participants been adequately considered?	Unclear – not mentioned in paper	Unclear - not mentioned in paper	Unclear	Unclear – status of interviewer mentioned but not discussed further	Unclear - not mentioned in paper	Unclear- not mentioned in paper	Unclear – not mentioned in paper	Yes, this was discussed	Yes, this was discussed	Yes, this was discussed	Yes, this was discussed

For peer review only

Have ethical issues been taken into consideration?	Yes	Yes	Yes	Yes	Unclear	Yes	Unclear	Yes	Unclear	Unclear – informed consent mentioned, but not ethical approvals	Unclear – informed consent mentioned, but not ethical approvals
Was the data analysis sufficiently rigorous?	Yes, apparently so (although not huge detail)	Yes	Unclear – not mentioned other than that the data was systematically analysed – reflects that it was not a qualitative study as such.	Yes	Unclear – very brief details and presented in quantitative manner	Yes	Yes	Yes	Yes	Yes	Yes
Is there a clear statement of findings?	Yes	Yes	Yes, but brief	Yes	Yes	Yes, but focus of report was not on reasons for withdrawing and so this was somewhat buried and limited.	Yes	Yes	Yes	Yes	Yes
How valuable is the research to our review?	Useful findings – NB: Limited 1st order construct data perhaps because paper also included data from trial retainers (and this seemed to be main emphasis)	Useful – perhaps one of the richer papers in terms of insights and data	Less useful than other studies but still provides helpful insights that help to build on the findings of other studies. Qualitative data presented was very limited (both 1st and 2nd	Useful – more 1st order constructs than some of the other papers e.g. Nakash, Sanders	Less useful than other studies but still provides helpful insights that help build on other studies. Qualitative data presented was very limited	Useful – but study focussed on decliners and withdrawers and provided limited data from the latter.	Useful – but only 3 were withdrawers, rest were decliners and so like Nakash the emphasis of the paper (and the bulk of data and reflection provided) was on decliners. Few 1st order	Useful – perhaps one of the richer papers in terms of insights and data	Useful, although limited 1st order constructs	Useful – more 1st order constructs than some of the other papers e.g. Nakash, Sanders	Useful findings – NB: Limited 1st order construct data perhaps because paper also included data from trial retainers – out of 20 participants interviewed,

For peer review only

			order constructs) and was discussed in a more quantitative way.		(both 1st and 2nd order constructs) and was discussed in a more quantitative way.		constructs from withdrawers.				only 4 had dropped out.

For peer review only

Enhancing transparency in reporting the synthesis of qualitative research: ENTREQ

ENTREQ Statement: content and rationale

The ENTREQ statement consists of 21 items grouped into five main domains: introduction, methods and methodology, literature search and selection, appraisal, and synthesis of findings (Table 1). For each item, a descriptor and examples are provided. Below we present a rationale for each domain and its associated items.

Table 1

Enhancing transparency in reporting the synthesis of qualitative research: the ENTREQ statement

No	Item	Guide and description	
**1**	Aim	State the research question the synthesis addresses.	See Page 3
**2**	Synthesis methodology	Identify the synthesis methodology or theoretical framework which underpins the synthesis, and describe the rationale for choice of methodology *(e.g. meta-ethnography, thematic synthesis, critical interpretive synthesis, grounded theory synthesis, realist synthesis, meta-aggregation, meta-study, framework synthesis).*	See Pages 3-4 and S1 Box
**3**	Approach to searching	Indicate whether the search was pre-planned (*comprehensive search strategies to seek all available studies)* or iterative (*to seek all available concepts until they theoretical saturation is achieved)*.	See Page 3
**4**	Inclusion criteria	Specify the inclusion/exclusion criteria *(e.g. in terms of population, language, year limits, type of publication, study type).*	See Page 3-4
**5**	Data sources	Describe the information sources used (e.g. *electronic databases (MEDLINE, EMBASE, CINAHL, psycINFO, Econlit), grey literature databases (digital thesis, policy reports), relevant organisational websites,*	See Page 3

No	Item	Guide and description	
		experts, information specialists, generic web searches (Google Scholar) hand searching, reference lists) and when the searches conducted; provide the rationale for using the data sources.	
**6**	Electronic Search strategy	Describe the literature search *(e.g. provide electronic search strategies with population terms, clinical or health topic terms, experiential or social phenomena related terms, filters for qualitative research, and search limits)*.	See Page 3 and Appendix 1
**7**	Study screening methods	Describe the process of study screening and sifting *(e.g. title, abstract and full text review, number of independent reviewers who screened studies).*	See Page 3-4
**8**	Study characteristics	Present the characteristics of the included studies *(e.g. year of publication, country, population, number of participants, data collection, methodology, analysis, research questions).*	See Page 4-5
**9**	Study selection results	Identify the number of studies screened and provide reasons for study exclusion *(e,g, for comprehensive searching, provide numbers of studies screened and reasons for exclusion indicated in a figure/flowchart; for iterative searching describe reasons for study exclusion and inclusion based on modifications t the research question and/or contribution to theory development).*	See Page 4 and Figure 1 and 2
**10**	Rationale for appraisal	Describe the rationale and approach used to appraise the included studies or selected findings *(e.g. assessment of conduct (validity and robustness), assessment of reporting (transparency), assessment of content and utility of the findings).*	See Page 3-4

No	Item	Guide and description	
**11**	Appraisal items	State the tools, frameworks and criteria used to appraise the studies or selected findings *(e.g. Existing tools: CASP, QARI, COREQ, Mays and Pope* [25]*; reviewer developed tools; describe the domains assessed: research team, study design, data analysis and interpretations, reporting).*	See Page 4 and S1 CASP checklist
**12**	Appraisal process	Indicate whether the appraisal was conducted independently by more than one reviewer and if consensus was required.	See Page 4. 1 reviewer (the main author) initially assessed quality of included studies using the CASP criteria and noted any critical aspects of quality with the study team. During subsequent group discussions we continued to discuss and reflect on key aspects of quality. Due to the small number of eligible studies we decided to include all (please see discussion section and also S1 CASP checklist)
**13**	Appraisal results	Present results of the quality assessment and indicate which articles, if any, were weighted/excluded based on the assessment and give the rationale.	Please see discussion section and S1 CASP checklist
**14**	Data extraction	Indicate which sections of the primary studies were analysed and how were the data extracted from the primary studies? *(e.g. all text under the headings "results /conclusions" were extracted electronically and entered into a computer software).*	See Page 4 and S1 Box
**15**	Software	State the computer software used, if any.	N/A
**16**	Number of reviewers	Identify who was involved in coding and analysis.	See Pages 4
**17**	Coding	Describe the process for coding of data *(e.g. line by line coding to search for concepts).*	See Page 4

No	Item	Guide and description	
**18**	Study comparison	Describe how were comparisons made within and across studies *(e.g. subsequent studies were coded into pre-existing concepts, and new concepts were created when deemed necessary).*	See Page 4, S1 Box, S3 Table
**19**	Derivation of themes	Explain whether the process of deriving the themes or constructs was inductive or deductive.	See Page 4,S1 Box, S3 Table
**20**	Quotations	Provide quotations from the primary studies to illustrate themes/constructs, and identify whether the quotations were participant quotations of the author's interpretation.	See Results section and S2 Table
**21**	Synthesis output	Present rich, compelling and useful results that go beyond a summary of the primary studies (e.g. *new interpretation, models of evidence, conceptual models, analytical framework, development of a new theory or construct).*	See Results and discussion section. Also see our conceptual model illustrating our 'line of argument' (S3 Figure).

For peer review only
